# Effect of Dietary Restriction and Subsequent Re-Alimentation on the Transcriptional Profile of Bovine Skeletal Muscle

**DOI:** 10.1371/journal.pone.0149373

**Published:** 2016-02-12

**Authors:** Kate Keogh, David A. Kenny, Paul Cormican, Matthew S. McCabe, Alan K. Kelly, Sinead M. Waters

**Affiliations:** 1 Animal and Bioscience Research Department, Animal and Grassland Research and Innovation Centre, Teagasc, Dunsany, Co. Meath, Ireland; 2 UCD School of Agriculture and Food Science, Belfield, Dublin 4, Ireland; University of Florida, UNITED STATES

## Abstract

Compensatory growth (CG), an accelerated growth phenomenon which occurs following a period of dietary restriction is exploited worldwide in animal production systems as a method to lower feed costs. However the molecular mechanisms regulated CG expression remain to be elucidated fully. This study aimed to uncover the underlying biology regulating CG in cattle, through an examination of skeletal muscle transcriptional profiles utilising next generation mRNA sequencing technology. Twenty Holstein Friesian bulls were fed either a restricted diet for 125 days, with a target growth rate of 0.6 kg/day (Period 1), following which they were allowed feed *ad libitum* for a further 55 days (Period 2) or fed *ad libitum* for the entirety of the trial. *M*. *longissimus dorsi* biopsies were harvested from all bulls on days 120 and 15 of periods 1 and 2 respectively and RNAseq analysis was performed. During re-alimentation in Period 2, previously restricted animals displayed CG, growing at 1.8 times the rate of the *ad libitum* control animals. Compensating animals were also more feed efficient during re-alimentation and compensated for 48% of their previous dietary restriction. 1,430 and 940 genes were identified as significantly differentially expressed (Benjamini Hochberg adjusted *P* < 0.1) in periods 1 and 2 respectively. Additionally, 2,237 genes were differentially expressed in animals undergoing CG relative to dietary restriction. Dietary restriction in Period 1 was associated with altered expression of genes involved in lipid metabolism and energy production. CG expression in Period 2 occurred in association with greater expression of genes involved in cellular function and organisation. This study highlights some of the molecular mechanisms regulating CG in cattle. Differentially expressed genes identified are potential candidate genes for the identification of biomarkers for CG and feed efficiency, which may be incorporated into future breeding programmes.

## Introduction

An accelerated growth phenomenon known as compensatory growth (CG) is incorporated into many animal production systems worldwide as a method to reduce costs [1]. The CG phenomenon may be defined as a physiological process whereby an animal has the potential, following a period of restricted feed intake, to undergo accelerated growth upon re-alimentation [2]. In addition to its usefulness as a method to reduce costs, exploitation of CG also typically yields more feed efficient animals during re-alimentation [2, 3, 4, 5]. Several studies have investigated the physiological control of CG [3, 4, 5, 6, 7, 8, 9, 10]. However, there is a dearth of information in the literature on the molecular mechanisms underpinning CG in cattle.

The recent advent of next generation sequencing technology allows for an in-depth analysis of the molecular control underlying phenotypes such as CG or greater feed efficiency, however, the analysis of potential transcriptional differences between restricted and re-alimenting animals during early CG using RNAseq has not been performed to date. RNAseq has distinct advantages over previous technologies utilised such as microarrays, including the sensitive detection of all genes without the need to generate an array of probes based on known sequences, little background noise and a much higher dynamic range [11]. Previous research using microarray technology [12] identified alterations in gene expression profiles in skeletal muscle in steers after 114 days of dietary restriction and after subsequent 84 days of re-alimentation. However, by day 84 of re-alimentation, the animals may have entered a normal growth trajectory [12] as the peak of CG has been shown to occur at approximately 60 days into re-alimentation [2]. Therefore, the objective of this study was to examine the molecular control of CG in *M*. *longissimus dorsi* tissue of Holstein Friesian bulls following (i) a period of feed restriction (120 days) and (ii) an initial period of re-alimentation (15 days) using RNAseq. Skeletal muscle was chosen as a target tissue, not only because of its obvious central economic importance to beef production but also because it accounts for approximately 50% of body mass and is in the order of 25% of basal metabolic energy expenditure [13]. A greater understanding of the molecular control regulating CG could be exploited to identify polymorphisms which could contribute to genomically assisted breeding programs in selecting animals with a greater ability to undergo CG and allow for a reduction in feed costs. Furthermore, identification of genes associated with feed efficiency in the current study could also be incorporated into selection programs specifically for the selection of more feed efficient animals.

## Materials and Methods

All procedures involving animals were approved by the University College Dublin, Animal Research Ethics Committee and licensed by the Irish Department of Health and Children in accordance with the European Community Directive 86/609/EC.

### Animal model

This experiment was conducted as part of a larger study designed to examine the physiological and molecular control of CG in growing beef cattle. The animal model and management has previously been described by Keogh et al. [9] and is briefly outlined here. Twenty purebred Holstein Friesian bulls (mean live weight 369 ± 31 kg; mean age 485 ± 14 days) which had been reared under the same management conditions from calf-hood were selected for inclusion in this study. Purebred Holstein Friesian bulls were used in order to prevent any potential confounding effects on RNAseq results of using crossbred animals. The animals were blocked on the basis of live weight, age and sire and were subsequently assigned within block to one of two dietary regimens (i) restricted feed allowance for 125 days (RES; n = 10) followed by *ad libitum* access to feed for a further 55 days or (ii) *ad libitum* access to feed throughout the 180 day trial (ADLIB; n = 10). The first 125 days of the trial were denoted as Period 1 and the subsequent 55 days, Period 2. During Period 1 RES animals were managed to achieve a target mean daily growth rate of 0.6 kg/day, whilst ADLIB animals were expected to gain between 1.5 and 2 kg/day. All animals were offered a total mixed ration diet consisting of 70% concentrate and 30% grass silage on a dry matter basis. Diets were fed individually, with the proportion of feed required based on each animal’s own individual bodyweight. Animals were weighed on two consecutive days at the start of the study, at the end of Period 1 and again at the end of Period 2. Additionally, throughout the experiment, animals were weighed every two weeks during Period 1 and once every week during Period 2. Weighing took place at the same time each morning prior to animals receiving fresh feed.

### Biopsy sample collection

*M*. *longissimus dorsi* biopsies were harvested from all bulls under local anaesthetic (5 mL Adrenacaine, Norbrook Laboratories (Ireland) Ltd.) on days 120 and 15 of periods 1 and 2 respectively. A 6 mm diameter, modified Bergstrom biopsy needle (Jørgen KRUUSE, Veterinary Supplies, Lyon, France) was used. All surgical instruments used for tissue collection were sterilised and treated with RNase Zap (Ambion, Applera Ireland, Dublin, Ireland). Biopsy samples were consistently taken at a depth of ~2.5 cm into the muscle tissue. Biopsies were washed in sterile DPBS, snap frozen in liquid nitrogen and subsequently stored at -80°C for long term storage pending further processing.

### RNA isolation and purification

Total RNA was isolated from the entire muscle biopsy harvested using TRIzol reagent (Sigma-Aldrich Ireland, Dublin, Ireland). Tissue samples were homogenised in 3 mL of TRIzol reagent using a rotor-strator tissue lyser (Qiagen, UK) and chloroform (Sigma-Aldrich Ireland, Dublin, Ireland). RNA was subsequently precipitated from all samples using isopropanol (Sigma-Aldrich Ireland, Dublin, Ireland). RNA samples were then purified using the RNeasy Plus Mini Kit (Qiagen, UK) according to the manufacturer’s instructions, which included a step to remove any contaminating genomic DNA. The quantity of the RNA isolated was determined by measuring the absorbance at 260 nm using a Nanodrop spectrophotometer ND-1000 (Nanodrop Technologies, DE, USA). RNA quality was assessed on the Agilent Bioanalyser 2100 using the RNA 6000 Nano Lab Chip kit (Agilent Technologies Ireland Ltd., Dublin, Ireland). RNA quality was also verified by ensuring all RNA samples had an absorbance (A260/280) of between 1.8 and 2. RNA samples with 28S/18S ratios ranging from 1.8 to 2.0 and RIN (RNA integrity number) values of between 8 and 10 were deemed to be of high quality.

### cDNA library preparation and sequencing

DNA libraries were prepared from high quality RNA using the Illumina TruSeq RNA sample prep kit following the manufacturer’s instructions (Illumina, San Diego, CA, USA). For each sample, 3 μg of RNA was used for DNA library preparation. Briefly, mRNA was purified from total RNA and then fragmented. First strand cDNA synthesis was performed using SuperScript II Reverse Transcriptase (Applied Biosystems Ltd., LifeTechnologies) subsequently synthesising the second strand using components of the Illumina TruSeq RNA sample prep kit. Adaptors were ligated to the cDNA which was then enriched by PCR. Final individual cDNA libraries were validated on the Agilent Bioanalyser 2100 using the DNA 1000 Nano Lab Chip kit, ensuring that library fragment size was ~260 bp and library concentration was >30 ng/μl. After quality control procedures, individual RNAseq libraries were pooled based on their respective sample-specific-6 bp adaptors and sequenced at 100 bp/sequence single-end read using an Illumina HiSeq 2000 sequencer. Approximately 37.5 million sequences per sample (Mean ± SD = 37,541,426 ± 4,204,042) were generated.

### RNAseq data analyses

Raw sequence reads were first checked for quality using FASTQC software (version 0.10.0). Input reads were then aligned to the bovine reference genome (UMD3.1) using TopHat (v2.0.9). The software package HTSeq (v0.5.4p5) (http://pypi.python.org/pypi/HTSeq) was employed to calculate the number of sequenced fragments overlapping all protein-coding genes from the ENSEMBL v74 annotation of the bovine genome. The number of counts of reads mapping to each annotated gene from HTSeq was then collated into a single file and used for subsequent differential gene expression. Differentially expressed genes were identified using the R (v2.14.1) Bioconductor package, EdgeR (v3.4.1). Genes with low read counts across all libraries were excluded from subsequent analysis. Remaining gene counts were normalised using the RLE method as implemented in EdgeR [14] to account for varying sequencing depth between samples. Transcript counts were modelled by fitting the data to a negative binomial distribution using moderated tagwise estimates of dispersion and differentially expressed genes were identified using a generalised linear model likelihood ratio test using a paired design and Benjamini Hochberg corrected *P* value of 0.1. Data analysis was undertaken to determine genes differentially expressed in RES animals relative to ADLIB animals at each time-point (Period 1 and Period 2). Additionally, data pertaining to the RES group at each time-point were analysed within treatment group, differentially expressed genes were identified in RES Period 2, relative to RES Period 1.

### Pathway analysis

Biological pathways that were significantly over-represented among differentially expressed genes were identified using the GOseq software (v.1.14.0) and Kyoto Encyclopaedia of Genes and Genomes (KEGG) pathway annotations. In RNAseq experiments the differences in transcript length can yield differing levels of total reads, even if transcripts are expressed at the same level. GOseq is an application for performing gene ontology analysis on RNAseq data while appropriately incorporating the effect of this selection bias [15]. Pathways are deemed over-represented when there are more differentially expressed genes in the pathway than would be expected given the size and gene length distribution. Due to the incomplete functional annotation of the bovine genome, to facilitate GOseq analysis, the online tool BioMart (www.ensembl.org/biomart/martview) was used to convert bovine gene IDs to their human orthologs. The set of differentially expressed genes was then applied to test KEGG pathways (http://www.genome.jp/kegg/pathway.html) for over- or under-representation. The significant KEGG pathway maps were examined for significant differentially expressed genes. To examine the molecular functions and biochemical pathways, the RNAseq data was further analysed using Ingenuity pathway analysis (IPA) (Ingenuity Systems, Redwood City, CA; http://www.ingenuity.com), a web-based software application that enables identification of over-represented biological mechanisms, pathways and functions most relevant to experimental datasets or genes of interest [16, 17, 18, 19].

## Results

### Animal performance

Differences in live weight gain, feed intake, and animal performance are outlined in detail by Keogh et al. [9]. Briefly, at the end of 125 days of differential feeding during Period 1, there was a 157 kg difference in bodyweight recorded between RES (444 kg ± 36 kg) and ADLIB (601 kg ± 47 kg) animals. Following 55 days of *ad libitum* feeding for both groups in Period 2, the difference in body weight had reduced to 86 kg (RES: 587 kg ± 36 kg; ADLIB: 673 kg ± 52 kg). Animals subjected to the restricted feeding regime during Period 1, managed to achieve their target growth rate of 0.6 kg/day during this period, with *ad libitum* control animals displaying a live weight gain of 1.9 kg/day during the same period. During Period 2, previously restricted animals, undergoing CG, exhibited a live weight gain of 2.6 kg/day, whilst *ad libitum* animals grew at 1.3 kg/day. These results indicate that the bulls in the present study showed accelerated growth upon re-alimentation and displayed a degree of CG as a consequence. A CG index [2] provides a method of quantifying the extent of the CG achieved; in the current study RES animals displayed a CG index of 45% after only 55 days of re-alimentation. Additionally, ultrasonic scanning of the *M*. *longissimus dorsi* revealed accelerated growth of this tissue in RES compared with ADLIB animals undergoing CG, during re-alimentation in Period 2 [9].

### mRNASeq read alignment and differential gene expression

The average (SD) number of raw reads across all samples was 37.5 million (mean ± SD = 37,541,426 ± 4,204,042). Approximately 85% of reads aligned to the bovine genome and 84% of those that aligned were mapped to the gene space. The bovine reference genome (UMD3.1) contains 26,740 gene constructs. At the end of dietary restriction in Period 1, the number of genes that had mapped reads was 10,882, whereas following 15 days of re-alimentation in Period 2, 10,977 genes had reads mapped to them. A total of 1,430 and 940 genes were identified as differentially expressed between RES and ADLIB in Period 1 and Period 2, respectively. These were manifested as 621 genes with increased expression and 809 genes with decreased expression in RES relative to ADLIB animals in Period 1. During CG, 548 and 392 genes had increased and decreased expression respectively in RES compared to ADLIB animals. Differentially expressed genes in both periods 1 and 2 are presented in S1 and S2 Tables, respectively. Between the two time points selected, 171 genes were differentially expressed following dietary restriction and subsequent re-alimentation. Of these 171 differentially expressed genes, 98 displayed different directions of their fold changes between the two sampling time points. Further details of these genes are provided in S3 Table. Additionally, 2,201 genes were identified as differentially expressed in the RES group during re-alimentation relative to the dietary restriction period (RES Period 2 compared with RES Period 1). From this analysis, 1,166 genes had greater expression and 1,035 genes had lower expression during re-alimentation relative to dietary restriction. Further details of these genes are provided in S4 Table. The RNAseq data have been deposited in NCBI’s Gene Expression Omnibus [20] and are accessible through GEO Series accession number GSE64289.

### Pathway analysis

**Fig 1:**
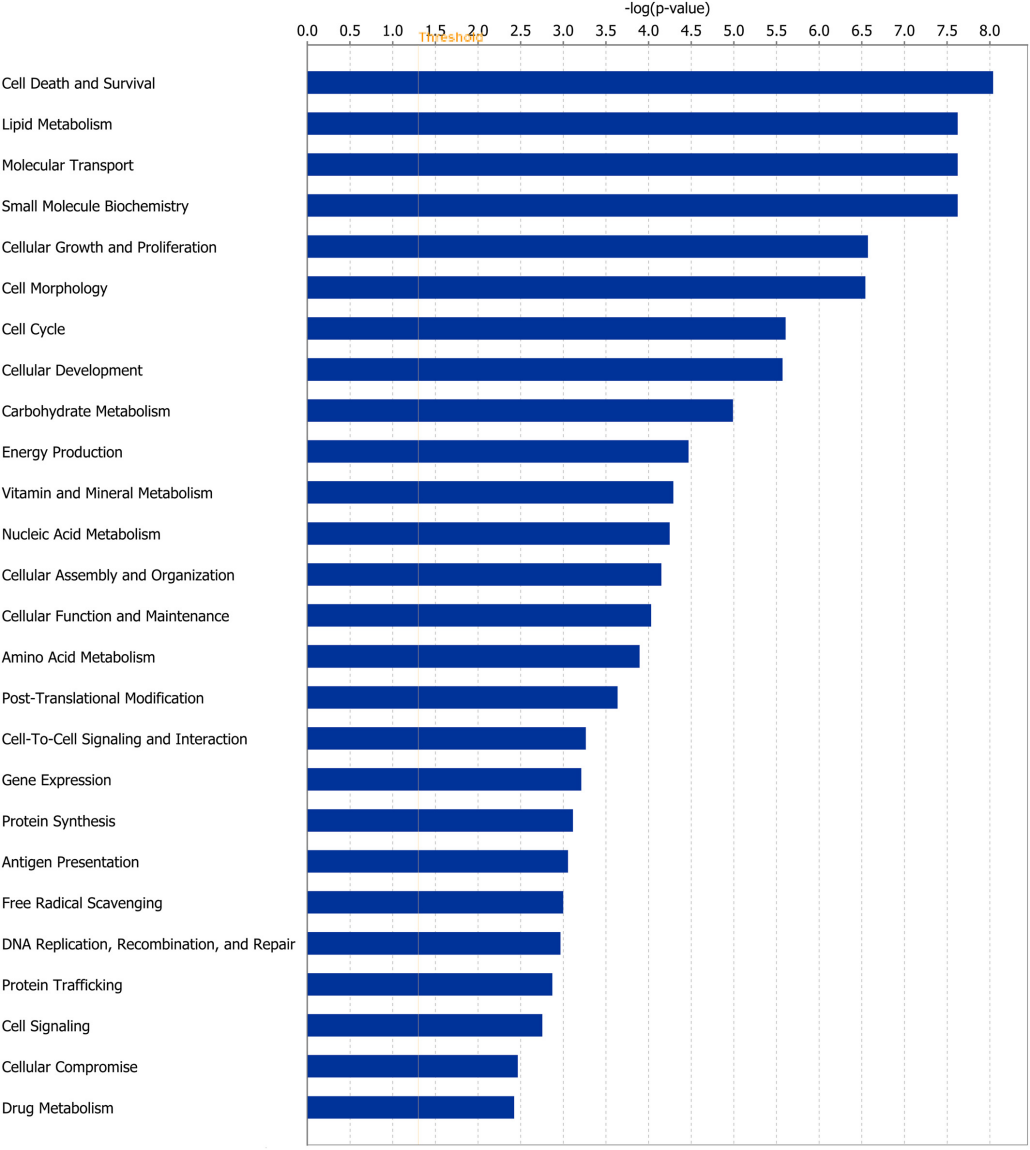
Molecular and cellular functions of differentially expressed genes affected by dietary restriction. Genes differentially expressed in bovine *M*. *longissimus dorsi* classified by molecular and cellular function in response to a period of dietary restriction (125 days) compared with *ad libitum*-fed cattle. The bars indicate the likelihood [-log (P-value)] that the specific molecular and cellular function was affected by restricted feeding compared with others represented in the list of differentially expressed genes. The threshold line in the bar chart represents a p-value of 0.05.

**Fig 2:**
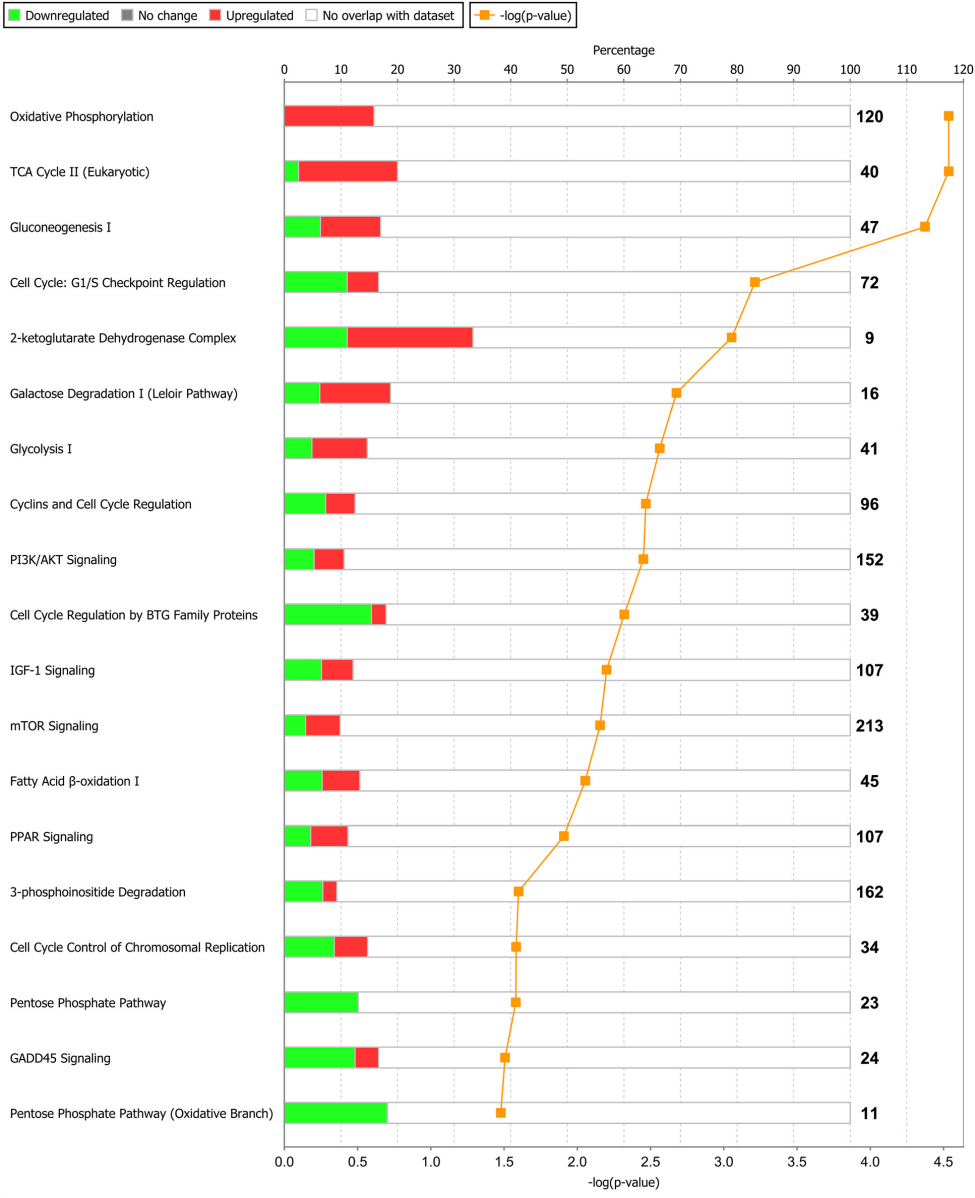
Biochemical pathways significantly enriched in bovine *M*. *longissimus dorsi* in response to a period of dietary restriction (120 days) compared with *ad libitum*-fed cattle. Green bars represent genes down-regulated and red bars up-regulated genes as percentages of the overall number of genes in each pathway. The significance of each pathway is represented by the yellow line describing—log(p-value). The p-value is calculated by the number of genes from our data-set of differentially expressed genes that participate in a particular pathway and dividing it by the total number of genes in that Canonical Pathway in IPA analysis.

**Fig 3:**
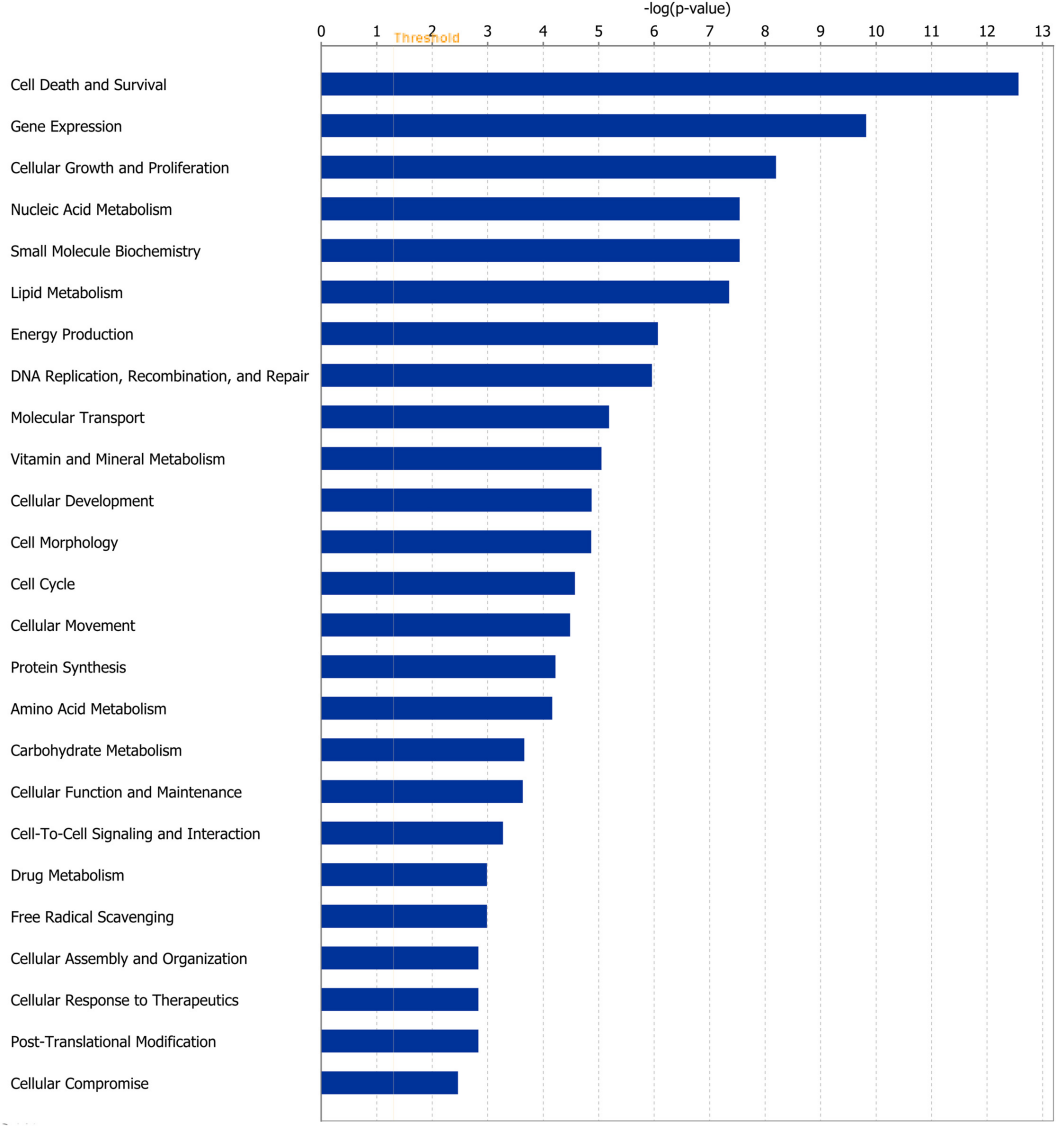
Molecular and cellular function of differentially expressed genes affected by re-alimentation and compensatory growth. Genes differentially expressed in bovine *M*. *longissimus dorsi* classified by molecular and cellular function in response to a period of re-alimentation (15 days) following a prior dietary restriction (120 days) compared with *ad libitum*-fed cattle. The bars indicate the likelihood [-log (P-value)] that the specific molecular and cellular function was affected by restricted feeding compared with others represented in the list of differentially expressed genes. The threshold line in the bar chart represents a p-value of 0.05.

**Fig 4:**
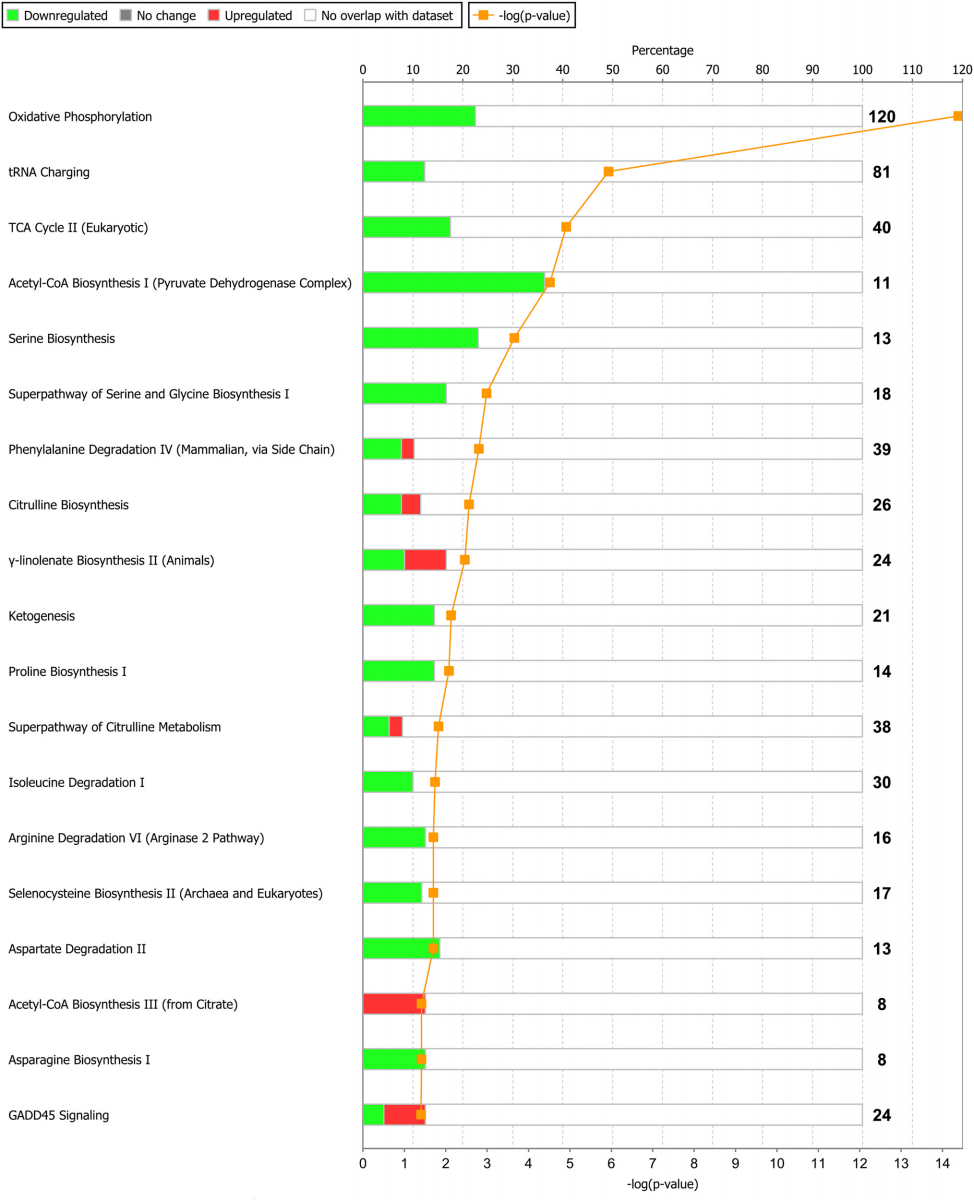
Biochemical pathways significantly enriched in bovine *M*. *longissimus dorsi* in response to a period of re-alimentation (15 days) and compensatory growth following a prior period of dietary restriction (120 days) compared with *ad libitum*-fed cattle. Green bars represent genes down-regulated and red bars up-regulated genes as percentages of the overall number of genes in each pathway. The significance of each pathway is represented by the yellow line describing—log(p-value). The p-value is calculated by the number of genes from our data-set of differentially expressed genes that participate in a particular pathway and dividing it by the total number of genes in that Canonical Pathway in IPA analysis.

**Fig 5:**
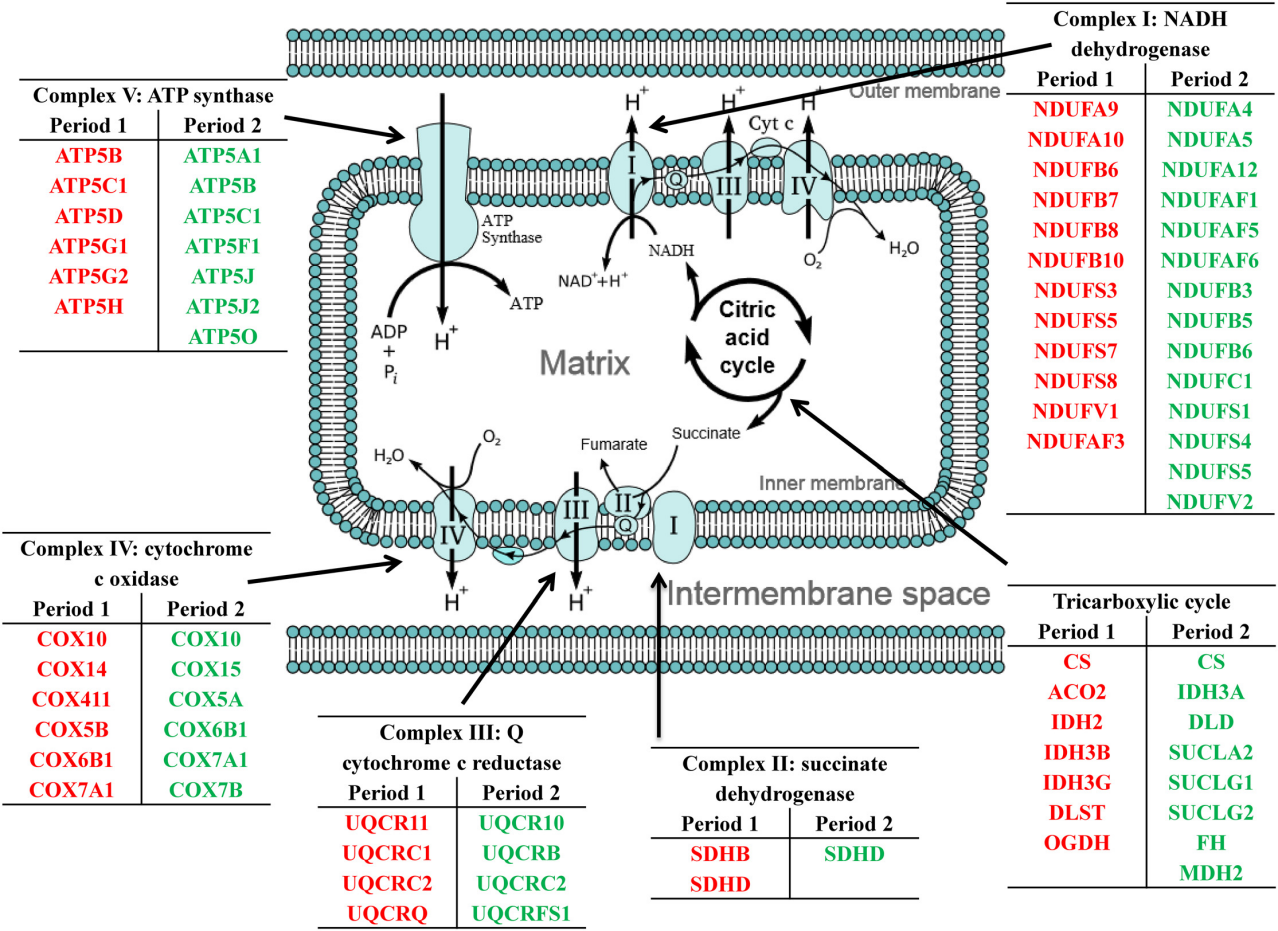
Differentially expressed genes of bovine *M*. *longissimus dorsi* associated with mitochondrial energy production during dietary restriction and compensatory growth. Up- (red) and down-regulated (green) genes associated with energy production in the TCA cycle and the electron transport chain following a period of dietary restriction (Period 1; 120 days) and also following a subsequent re-alimentation period (Period 2; 15 days) compared to *ad libitum*-fed cattle.

Of the 1,430 differentially expressed genes at the end of Period 1, and 940 differentially expressed genes at the end of Period 2, 1,299 and 812 genes were successfully mapped to a molecular or biological pathway and/or category in the IPA database in periods 1 and 2, respectively. Differentially expressed genes were analysed and separated according to their biological functions within IPA. Details of functional processes and pathways affected by dietary restriction are presented in Figs 1 and 2 respectively. Functional processes and pathways affected by re-alimentation and CG in skeletal muscle are outlined in Figs 3 and 4 respectively. From pathway analysis in IPA, a pathway or functional process was found to be significant based on the number of genes identified as differentially expressed in the current dataset relative to the number of genes involved in particular pathways or functional processes in the IPA database. However, further evaluation of the direction of change of these genes meant that in some cases although a pathway or functional process was significant the pathway or process was not up- or down-regulated overall in response to dietary restriction or subsequent CG. As a consequence the most affected pathways/functional processes have been presented and discussed in the current study. Upon further examination of biochemical pathways and functional processes pertaining to IPA analysis, the following were identified as being the most affected processes in skeletal muscle following a period of dietary restriction and also subsequent re-alimentation induced CG: beta-oxidation and lipid metabolism and energy production through alterations in the expression of genes of the tricarboxylic acid cycle (TCA) and oxidative phosphorylation. Direction of change of the differentially expressed genes pertaining to these processes suggested an up-regulation of beta-oxidation, TCA and oxidative phosphorylation in response to dietary restriction. The inversion of these results was apparent following 15 days of re-alimentation. Further details of genes involved in beta oxidation, are presented in Tables 1 and 2. Additionally, genes associated with TCA and oxidative phosphorylation differentially expressed at each time-point are outlined in Fig 5. Functional analysis of genes differentially expressed in the skeletal muscle of cattle undergoing CG (RES, Period 2) relative to those at the end of a period of dietary restriction (RES, Period 1) revealed alterations in a number of processes involved in cellular function and organisation, including cytoskeleton, cellular transporters and protein folding (Tables 3 and 4). Overall fold changes of these genes suggested up-regulation of these processes during re-alimentation.

**Table 1:** Fatty acid degradation and synthesis genes, differentially expressed in *M*. *longissimus dorsi* of Holstein Friesian bulls following a 120 day period of restricted feeding at the end of Period 1 and after 15 days of subsequent re-alimentation in Period 2.

Gene symbol	Gene name	Fold change^1^	P value
Period 1			
*ACACA*	Acetyl-CoA carboxylase alpha	-2.021	0.006808
*ACADL*	Acyl-CoA dehydrogenase, long chain	-1.317	0.004043
*ACADM*	Acyl-CoA dehydrogenase, C-4 to C-12 straight chain	1.335	0.00035
*CPT1B*	Carnitine palmitoyltransferase 1B (muscle)	1.465	8.59E-05
*ECI2*	Enoyl-CoA delta isomerase 2	1.386	4.10E-05
*ELOVL5*	ELOVL fatty acid elongase 5	-2.425	0.000534
*ELOVL6*	ELOVL fatty acid elongase 6	-9.547	1.70E-07
Period 2			
*ACADM*	Acyl-CoA dehydrogenase, C-4 to C-12 straight chain	-1.278	0.00436
*CPT1B*	Carnitine palmitoyltransferase 1B (muscle)	-1.358	0.001416
*DGAT1*	Diacylglycerol O-acyltransferase 1	1.402	0.00369
*SLC27A6*	Solute carrier family 27 (fatty acid transporter), member 6	2.765	6.04E-05

^1^ Fold changes are up or down in restricted fed animals compared to *ad libitum* control animals

**Table 2:** Beta oxidation and fatty acid synthesis genes, differentially expressed in *M*. *longissimus dorsi* of Holstein Friesian bulls following a 15 day re-alimentation period in Period 2, relative to a 120 day period of dietary restriction (Period 1).

Gene symbol	Gene name	Fold change^1^	P value
**Beta-oxidation**			
*ACAA1*	Acetyl-CoA acyltransferase 1	-1.267	1.52E-03
*ACAD10*	Acyl-CoA dehydrogenase family, member 10	-1.771	1.62E-11
*ACADM*	Acyl-CoA dehydrogenase, C-4 to C-12 straight chain	-1.252	7.85E-03
*ECHS1*	Enoyl CoA hydratase, short chain, 1, mitochondrial	-1.289	4.78E-04
*ECI2*	Enoyl-CoA delta isomerase 2	-1.257	3.26E-03
**Fatty acid synthesis**			
*ACSF2*	Acyl-CoA synthetase family member 2	1.426	2.80E-03
*ELOVL6*	ELOVL fatty acid elongase 6	2.976	2.41E-04
*FABP3*	Fatty acid binding protein 3, muscle and heart	1.889	7.36E-10
*FADS3*	Fatty acid desaturase 3	2.349	5.96E-11
*HSD17B12*	Hydroxysteroid (17-beta) dehydrogenase 12	1.586	5.22E-05
*HSD17B7*	Hydroxysteroid (17-beta) dehydrogenase 7	1.531	1.53E-03
*HSD17B8*	Hydroxysteroid (17-beta) dehydrogenase 8	1.528	3.58E-08
*SQLE*	Squalene epoxidase	2.521	4.21E-06

^1^ Fold changes are up or down in compensating animals compared to restricted fed animals

**Table 3:** Genes involved in cellular function and organisation differentially expressed in *M*. *longissimus dorsi* of Holstein Friesian bulls following a 15 day compensatory growth and re-alimentation period in Period 2, relative to a 120 day period of dietary restriction (Period 1)^1^.

**Cellular interactions**
**Adhesion:** *ADGRE5 ADGRL1 ARSB C1QTNF5 CCDC80 CD44 CD93 CDH11 CDH13 CDH23 CRELD1 DCHS1 FBLN1 FBN1 GJA1 ICAM3 ITGB2 LAMA4 LAMB1 LIMS2 MICALL2 NCAM1 NEXN NPNT PARVA PCDH12 RASIP1 RBBP8 SPON1 SPON2 TNFRSF12A VCAN*
**Cell migration:** *ACTA2 ACTB ACTC1 ACTG1 ACTN1 MARCKS MIEN1 ROBO1 SH2D3C SLIT3*
**Cell-cell interactions:** *CLIP2 CPXM1 EMP3 ITGA7 LGALS1 NLGN2 THBS1*
**Matrix:** *ADAMTSL2 ANTXR2 CHST15 CRISPLD2 DPT ECM2 EMILIN1 HSPG2 LMNB1 LOXL1 LOXL2 MMP15 MMP16 MMP19 MRC2 MXRA5 NOV PRELP SH3PXD2B TIMP1*
**Cytoskeleton**
**Cytoskeleton:** *ABI3 ACTR1A ACTR3 AFAP1L1 ANK1 ARAP3 ARL6IP5 ARPC3 ARPC5 B9D1 B9D2 CCDC13 CDC42EP1 CDC42EP3 CDC42SE1 CFL1 CFL2 CKAP4 COL15A1 COL1A1 COL1A2 COL4A1 COL4A2 COL5A3 COL6A1 COL6A3 COL6A6 COL8A1 COL8A2 COLGALT1 DYNC1LI2 DYNLL1 EMD FAM101B FHOD3 FLNA FSCN1 IFT140 IFT172 IFT20 KPTN LASP1 MACF1 MARCKSL1 MFAP2 MSN MYOM3 MYOT OBSL1 P4HA1 P4HA2 PCOLCE PLD2 PLOD1 PLOD2 PLOD3 RND2 SEPT5 SEPT6 SEPT8 SERPINH1 SGCG* ***SSH2*** *STMN1 TCTN1 TGM2 TMOD1 TUBA1A TUBA1B TUBA1C TUBB TUBB6 VIM WIPF1*
**Myosin: *MYBPC1 MYH14 MYH4 MYLIP MYLK2 MYLK3 MYO9A MTMR12 MTMR14 MTMR3 MTMR7***
**Kinesin: *KIF13A KIF1B KIF3A KLC4***
**Cellular transport**
**Channels and transporters: *ATP11A ATP13A3 ATP1A4 ATP1B2 ATP1B4 ATP2A2 ATP2C2 ATP7A ATP8A1*** *KCNA5 KCNB1 KCNJ8 KCNMA1 KCNQ4 KCNQ5 KCTD11 KCTD15 KCTD21 KCTD3 LRRC8D P2RX5 PIEZO1* ***SCN4A*** *SLC10A3 SLC15A4 SLC16A13 SLC16A6 SLC1A4 SLC22A17 SLC22A23 SLC22A5 SLC25A11 SLC25A15 SLC25A20 SLC25A30 SLC25A39 SLC25A5 SLC27A6 SLC31A1 SLC35E3 SLC35E4 SLC37A4 SLC38A2 SLC38A4 SLC39A1 SLC39A14 SLC39A6 SLC3A2 SLC9A2 SLC9A5 SLC9A9 SLCO2A1 TPCN1 TTYH2 ZACN*
**Vesicles and endocytosis:** *AGFG1* ***AKTIP*** *AP4B1* ***AP4S1*** *AP5S1 AP1G2 AP1S1 AP1S2 CAST C9orf72 DAB2 EHD3 EXOC2 FAM109A* ***KXD1*** *NAPG NIPSNAP3A PICALM* ***PIKFYVE*** *PRAF2* ***RAB11FIP2*** *RAB7B SH3GL1 SMAP2 STX6 SYT7* ***TBC1D17*** *TMED1 TMED3 TMED4 TMED5 TRAPPC6A* ***TRIM23*** *UNC13B* ***VPS33A*** *VPS45*

^1^Genes highlighted in bold are down-regulated in compensating animals compared with restricted fed animals

**Table 4:** Genes involved in protein folding and the ribosome differentially expressed in *M*. *longissimus dorsi* of Holstein Friesian bulls following a 15 day compensatory growth and re-alimentation period in Period 2, relative a 120 day period of dietary restriction (Period 1)^1^.

Protein synthesis
**Ribosome:** *ABT1* ***AFF4*** *BYSL* ***CTDSP2 ELL2*** *GID4 GPN3 GRWD1 NOL6 NOL9 NOP14 NOP16 NOP58 RPL22 RPL3 RPL32 RPL6 RPL7 RPL8 RPP40 RPS6KA1 RRP15*
**tRNA:** *CARS DARS2 DUS1L GARS MARS MARS2 NARS2 TARS VARS WARS YARS YARS2 IARS*
**Endoplasmic reticulum:** *ADAM17 ADAM19 ARMC5* ***ART3 ART5 CNST CREBRF*** *CALU DERL1* ***EDEM3*** *KDELC1 KDELC2 KDELR3 NUTF2 REEP1 Rrbp1 SEC61A2 SRPR SYS1 TTC1*
**Protein folding:** *AHSA1* ***BAG4*** *BCS1L CALR CANX CCT2 CCT3 CCT4 CCT5 CCT6A CCT7 CCT8 CHCHD4 CHORDC1 DNAJA1 DNAJA4 DNAJB1 DNAJB11* ***DNAJB12*** *DNAJB4 DNAJC12 DNAJC21 DNAJC24* ***DNAJC3*** *FKBP10 FKBP14 FKBP1A FKBP3 FKBP7 FKBP9 HSP90AA1 HSP90AB1 HSP90B1 HSPA4 HSPA5 HSPA8 HSPB1 HSPB8 HSPBP1 HSPD1 HSPE1 HSPH1 HYOU1 PPIC PPID PPIF PPIL1 PPIL3 PPWD1 STIP1 TMEM126B*

^1^Genes highlighted in bold are down-regulated in compensating animals compared with restricted fed animals

## Discussion

Feed accounts for up to 80% of the production costs of cattle and thus producers have exploited the CG phenomenon for many years in order to reduce the lifetime feed costs of the animals they produce. Many studies have examined various aspects of the biological control of CG in cattle including energy partitioning and tissue growth [3, 4, 5, 9, 21, 22] and endocrinology [6, 7, 8, 10, 23]. While understanding the biological processes underpinning CG is important, elucidation of the genetic basis for this trait, should it exist, would facilitate the future identification of animals with a greater genetic potential to undergo moderate feed restriction and exhibit CG. To date few studies have examined the molecular control of CG in cattle, indeed in these studies a microarray platform was employed [12, 24]. This is the first study to employ next generation sequencing technology to the examination of CG in cattle. *Longissimus dorsi* tissue was targeted, firstly due to its high economic value but also due to its large metabolic energy cost [13].

### Fatty acid degradation and synthesis

It is well established that variation in plane of nutrition induces alterations in tissue metabolism [25], including changes in the rate of fatty acid synthesis and degradation [26]. Mitochondrial fatty acid beta oxidation is the principal pathway for the oxidation of fat [27]. This degradation pathway brings about the oxidation of long-chain fatty acids to produce energy in the form of acetyl coenzyme A. Acetyl coenzyme A (acetyl CoA) can then be subsequently fed into the citrate cycle, with the ultimate end point being the production of ATP. Additionally reducing powers; FADH2 and NADH produced during beta oxidation can feed directly into oxidative phosphorylation, further increasing the production of energy from fatty acid stores. Following a period of restricted feeding in Period 1, the expression of genes associated with the beta oxidation of fatty acids was significantly altered between restricted and *ad libitum* fed animals. Selman et al. [28] also observed in both liver and muscle tissues, a switch away from energetically expensive biosynthetic processes, towards fatty acid metabolism, beta-oxidation and gluconeogenesis as the dominant energy pathways during calorie restriction. Up-regulation of a number of genes of this pathway in the restricted fed animals in the current study implies greater energy production from lipid stores in the body during dietary restriction. In eukaryotes, fatty acid degradation occurs in the mitochondrial matrix. However, the inner mitochondrial matrix is not permeable to long-chain acyl CoA derivatives and so these are transported into the mitochondria as carnitine derivatives by carnitine/acyl carnitine translocase enzymes such as the protein encoded by the muscle specific *CPT1B* transcript [29, 30]. As this gene codes for the rate-controlling enzyme of the beta-oxidation pathway [25], the up-regulation of this gene that we observed in this study, following a period of restricted feeding, may suggest that a greater quantity of fatty acids were being oxidised to produce energy in feed restricted animals. The increased utilisation of fatty acid reserves to meet energy needs by restricted animals, was further supported by the observed up-regulation of a number of other enzymes in this pathway. Beta oxidation involves a repeating sequence of four enzymatic steps in which an acyl-coenzyme A ester (acyl-CoA) undergoes subsequent steps of dehydrogenation, hydration, another dehydrogenation and finally thiolytic cleavage [27]. Acyl-CoA dehydrogenases are a class of enzymes that function to catalyse the initial step in each cycle of mitochondrial beta oxidation. Whilst different dehydrogenases target fatty acids of varying chain length, all types of acyl-CoA dehydrogenases are mechanistically similar. In the current restricted fed animal model, *ACADL* (long chain fatty acids) and *ACADM* (medium chain fatty acids) which are component genes coding for acyl-CoA dehydrogenases were identified to be differentially expressed in Period 1. During Period 1, expression of *ACADM* was greater in RES animals, while *ACADL* expression was less in the same treatment group, indicating that breakdown of medium and long chain fatty acids were being oxidised preferentially over long chain fatty acyl-CoAs. In addition to the set of enzymes involved in this beta oxidation cycle, the degradation of unsaturated fatty acids requires the obligatory participation of a set of 3 auxiliary enzymes including enoyl-CoA isomerase (ECI) [31]. In Period 1, expression of *ECI2*, which codes for enoyl-CoA isomerase [32] was greater in RES animals, indicating oxidation of both saturated and unsaturated fatty acids. Alterations in lipid metabolism gene expression, specifically an increased expression of genes involved in lipid degradation has also been observed in hepatic tissue in mice when subjected to a calorie restriction regime [33, 34]. The genes identified as differentially expressed in the fatty acid beta oxidation pathway in Period 1, were subsequently not different in Period 2, with the exception of *CPT1B* and *ACADM*. An inverse relationship was evident between the expression of *CPT1B* and nutrition treatment, whereby expression of this gene was lower in re-alimented animals during Period 2. As this gene encodes the rate-controlling enzyme of beta oxidation in muscle mitochondria, down-regulation suggests down regulation of the pathway overall and a potential for triglyceride synthesis over degradation during CG in Period 2. This was further established though up-regulation of fatty acid synthesis genes and down-regulation of beta-oxidation genes in RES animals during re-alimentation relative to dietary restriction (Table 2).

As well as an increase in the expression of genes associated with fatty acid beta oxidation in Period 1, a decrease in the expression of genes associated with fatty acid synthesis was also evident in RES animals in the same period. This mirrored a reduction in adipose tissue deposition in these animals during dietary restriction [9]. An increase in fatty acid oxidation and a decrease in fatty acid synthesis have also been implicated previously as the main metabolic consequences of food restriction in other studies [35, 36, 37]. Fatty acid synthesis involves a separate series of reactions to beta oxidation including the build-up of long chain hydrocarbons from acetyl CoA units, requiring ATP. Genes including *ACACA* which codes for the enzyme that catalyses the first committed step in fatty acid biosynthesis [38], and two enzymes involved in fatty acid elongation *ELOVL5* and *ELOVL6* were all down regulated in restricted animals. Down regulation of *ACACA* was also observed in both liver and adipose tissues in calorie restriction studies in pigs [34]. Additionally, *ELOVL6* has previously been shown to be regulated by fasting and subsequent re-feeding [39]. Turyn et al. [40] also noted a reduction in *ELOVL6* expression during feed restriction in adipose tissue, with the authors of that study noting that re-feeding after fasting caused a significant increase of *ELOVL6* mRNA levels in adipose tissue. Up-regulation of *DGAT1* during re-alimentation indicates that this gene may have been involved in fatty acid biosynthesis in RES animals. This gene codes for an enzyme that catalyses the terminal, and only committed step, in triacylglycerol synthesis by using diacylglycerol and fatty acyl CoA as substrates [25]. Consistent with the up-regulation of genes involved in fatty acid synthesis, *SLC27A6*, a fatty acid transporter was also up-regulated in RES animals in Period 2. Consistent with gene expression in these animals greater deposition of subcutaneous fat was also recorded in these animals during Period 2 [9].

### Energy production

Mitochondria are the site of energy production within the cell, with the tricarboxylic acid (TCA) cycle, coupled with the electron transport chain, generating approximately 90% of cellular ATP within this organelle [41, 42]. The majority of the body's catabolic pathways converge on the TCA cycle. For example, the intermediates formed as part of fatty acid beta oxidation are channelled towards the TCA cycle. The cycle has eight stages, catalysed by eight different enzymes. In period 1, a number of genes coding for these enzymes, or subunits of the enzymes, were up regulated in RES animals (Fig 5), suggesting a greater capacity for the efficient use of substrates in cellular energy production in these animals. Specifically, genes coding for isocitrate dehydrogenase (*IDH2*, *IDH3B* and *IDH3G*), alpha-ketoglutarate dehydrogenase (*OGDH* and *DLST*) and succinate dehydrogenase (*SDHB*) which produce both NADH and FADH_2_ as a result of their catalytic reactions, were found to be up-regulated in RES compared with ADLIB. Intermediates formed as part of the actions of these genes may then progress further towards energy production through incorporation into the electron transport chain. For example, the step catalysed by succinate dehydrogenase, the oxidation of succinate to fumarate with the reduction of ubiquinone to ubiquinol, occurs in the inner mitochondrial membrane thus coupling the two reactions of the citric acid cycle and the electron transport chain together. Oxidative phosphorylation, the final stage in aerobic cell respiration, produces ATP through the transfer of electrons through the electron transport chain on the inner mitochondrial membrane. Electrons are transferred between four enzyme complexes on the inner mitochondrial membrane, with the final step of ATP production occurring at complex five. In each period of the current study, genes associated with each of the five complexes of the electron transport chain were differentially expressed between the two dietary regimens, namely: complex 1: NADH reductase; complex II: succinate reductase; complex III Q-cytochrome c reductase; complex IV: cytochrome c oxidase and complex V: ATPsynthase. Electron movement down the respiratory chain to the terminal electron acceptor, O_2_, is coupled to proton pumping from the matrix to the inter-membrane space. The resulting proton motive force consisting of a membrane potential and a pH gradient, provides energy for ATP synthesis as protons flow into the matrix through ATPsynthase [43]. Differential expression of genes regulating oxidative phosphorylation was not evident after 114 days of feed restriction, nor following 84 days of subsequent re-alimentation in bovine skeletal muscle, following different levels of feed restriction [12]. Down regulation of two genes coding for subunits of cytochrome c oxidase (subunits 1 and III) was observed in *longissimus dorsi* in Brahman steers after 27 days of a more harsh restricted feeding regime when compared to that utilised in the present study [44]. However, the larger number of oxidative phosphorylation genes identified as differentially expressed in the present dataset suggests that feed restriction was associated with greater mitochondrial function. However as mitochondrial functional assays were not performed in the current study such a conclusion cannot be drawn, alternatively greater expression of mitochondrial genes may be the consequence of a greater abundance of mitochondria during dietary restriction, however we did not identify any mitochondrial biogenesis genes to be differentially expressed following dietary restriction. Moreover, calorie restriction has previously been shown to cause increased expression of genes involved in mitochondrial energy metabolism [28, 45]. Data from our own group [46] and others [43] have shown that feed efficiency affects the coupling of the enzyme reactions of the electron transport chain. Bottje et al. [47] reported differences in mitochondrial proton leak kinetics between low and high feed efficient broilers, whereby inefficient animals exhibit leakage of protons out of the inner mitochondrial membrane, reducing the amount of ATP produced, with the reverse effect recorded in efficient mitochondria. Additionally, in beef cattle, Kolath et al. [42] observed higher muscle mitochondrial activity in Angus steers exhibiting higher feed efficiency compared to steers with lower feed efficiency. During Period 1, restricted feed intake in RES animals caused greater expression of genes of each complex. This result may be indicative of a greater mitochondrial efficiency in these animals over ADLIB animals. Indeed, Lopez-Lluch et al. [48] observed that calorie restriction produces very efficient electron transport through the respiratory chain. However although greater mitochondrial efficiency has been reported in other cases of dietary restriction, such a result cannot be concluded upon in the current study as mitochondrial functional assays were not performed. Selman et al. [28] suggested that shifts in cellular metabolic pathways observed during calorie restriction, such as those towards gluconeogenesis and beta-oxidation are energetically expensive and require enhanced mitochondrial function to provide ATP. Alternatively it may be due to a greater abundance of mitochondria in animals undergoing feed restriction as a method to produce ATP as previous work has reported that calorie restriction is capable of inducing the enzyme endothelial nitric oxide synthase which can result in an increase in mitochondrial biogenesis [49, 50]. Lopex-Lluch et al. [48] reported that increased mitochondrial efficiency as a consequence of calorie restriction was the result of an increase in the number of mitochondria.

Following 15 days of re-alimentation in Period 2, the direction of the changes of a number of these genes had reversed. Genes that were subsequently down regulated in Period 2 included *CS* and *SDHD*. Additionally, a number of genes that had not been affected by feed restriction were altered as a result of CG during re-alimentation (Fig 5). Down regulation of all differentially expressed genes of the citric acid cycle in Period 2, indicates a clear lack of potential energy efficiency in animals undergoing CG during re-alimentation. This would have resulted in less energy production, which is further evidenced through down regulation of genes coding for the enzyme succinyl CoA synthetase which produces ATP as a consequence of catalysis. Furthermore, after 15 days of re-alimentation in Period 2, all differentially expressed genes of the oxidative phosphorylation pathway were down-regulated. The transposition of previously up-regulated genes, in addition to other down-regulated genes in Period 2, may potentially suggest a lack of coupling of the electron transport chain during re-alimentation, however again having not performed mitochondrial functional assays, such a conclusion cannot be drawn upon in the current study. In their study of hepatic gene expression in steers during dietary restriction and subsequent CG, Connor et al. [24], identified greater expression of genes associated with each complex on the first day of re-alimentation after a period of restricted feeding, with the authors suggesting that greater mitochondrial efficiency occurred in re-alimenting animals, undergoing CG. This has also been demonstrated in previous research where significant increases in mitochondrial abundance and mitochondrial efficiency in hepatic cells in response to caloric restriction of rodents has been observed [48]. However, the initial up-regulation of oxidative phosphorylation genes observed by Connor et al. [24] were subsequently found to be down-regulated or not detectable by day fourteen of their study. In our study, which examined molecular response in skeletal muscle tissue, the data suggest that greater mitochondrial efficiency during dietary restriction may sustain into re-alimentation as observed by Connor et al. [24]. However, down regulation of these genes by day 15 of re-alimentation in CG in Period 2, indicate that improved energy production is not a major biochemical process regulating the compensation of skeletal muscle tissue and does not contribute wholly to the overall occurrence of CG.

### Cellular function and organisation

Following a period of dietary restriction, processes associated with cellular function and organisation, for example, cellular transportation and cellular interactions may be down-regulated in order to cope with reduced feed intake and increase cellular survival [51]. This has previously been observed in cattle following a period of dietary restriction, where genes involved in the cytoskeleton and extracellular matrix were down-regulated in skeletal muscle [12, 44] as well as in hepatic tissue [24]. Additionally, with lower nutrient intake and subsequent availability for use in the animal, the need for cellular transport of molecules may be lessened. Indeed, in the work of Keogh et al. [52] following a period of dietary restriction lower expression of nutrient transporter genes was evident in bovine hepatic tissue. Conversely though when feed supply becomes plentiful again there may be an increase in these cellular functions. A similar finding was observed in the current study, whereby through an evaluation of genes that were differentially expressed in muscle tissue of cattle undergoing CG relative to dietary restriction (RES group, Period 2 relative to Period 1) up-regulation of genes involved in processes involved in cellular organisation and function was evident. More specifically these included cellular interactions, the cytoskeleton, cellular transporters, the ribosome and protein folding. Up-regulation of these processes during early CG may have reflected a necessary adaptive requirement for cells to be able to cope with increased activity upon re-alimentation. Additionally, greater expression of genes associated with cellular function and organisation may be required to allow myocytes to build up the capacity to be prepared for subsequent accelerated growth of skeletal muscle tissue, which has previously been observed in cattle during re-alimentation [9]. During re-alimentation we observed up-regulation of genes involved in cellular interactions, the cytoskeleton, adhesion, attachment, migration, and also genes coding for protein components of the cellular matrix (Table 3). These processes play important roles in the normal functioning of cells and contribute to cellular organisation and structure, up-regulation of these genes during re-alimentation suggests greater cellular organisation and structure in myocytes. The cytoskeleton and extracellular matrix have previously been shown to be responsive to feeding level in adipose tissue [53]. Connor et al. [24] also observed up-regulation of genes involved in cellular organisation in bovine hepatic tissue upon re-alimentation following a prior dietary restriction, indeed a number of these genes including: *ACTA2*, *ACTB*, *ACTG1*, *ACTN1*, *COL4A1*, *ARPC3* and *VIM* were identified as commonly expressed in animals undergoing CG in the current study. Furthermore, alterations in the expression of genes coding for proteins involved in membrane channels and transporter proteins was also apparent. Overall there was an up-regulation of genes involved in intracellular transport as well as membrane channels including voltage gated channels and solute like carriers. Three genes coding for solute like carrier transporting proteins (*SLC25A11*, *SLC25A39* and *SLC27A6*) have previously been identified as differentially expressed in response to re-alimentation following a prior dietary restriction [24]. Additionally, differentially expressed genes showed an up-regulation of genes involved in vesicle trafficking and docking as well as endocytosis, indicating greater transport of cellular constituents during re-alimentation. However although genes coding for proteins involved in passive or facilitated transport were up-regulated in re-alimenting animals relative to dietary restriction, we did observe down-regulation of active transporter proteins which require input of ATP. Down-regulated ATPase transporter genes included: *ATP11A*, *ATP13A3*, *ATP1A4*, *ATP1B2*, *ATP1B4*, *ATP2A2*, *ATP2C2*, *ATP7A* and *ATP8A1*. Likewise genes coding for cytoskeletal motor proteins including kinesins and myosins which also require input of ATP for functionality were also down-regulated during re-alimentation (Table 3), suggesting that cellular ATP was being used for functions or processes other than cellular organisation during early re-alimentation.

The data of both Connor et al. [24] and Keogh et al. [52] identified differential expression of ribosomal genes in response to dietary restriction and subsequent re-alimentation induced CG. Likewise in the current study, during re-alimentation we observed up-regulation of translational associated genes, including those coding for ribosomal biogenesis proteins, ribosomal subunits and tRNAs, which function to transfer mRNA sequence code into a peptide code (Table 4). Eleven ribosomal associated genes up-regulated in the current study in animals undergoing re-alimentation relative to dietary restriction were also up-regulated in hepatic tissue at the beginning of re-alimentation [24]. These included six genes coding for the ribosome (*RPL22*, *RPL3*, *RPL32*, *RPL6*, *RPS6KA1*, *RRP15*) and five tRNA genes: *CARS*, *DUS1L*, *NARS2*, *TARS* and *SARS*. Up-regulation of genes associated with the endoplasmic reticulum was also apparent during re-alimentation. As a cellular organelle involved in protein synthesis, up-regulation of endoplasmic reticulum associated genes further establishes greater protein synthesis during re-alimentation. Indeed, greater protein synthesis and tissue deposition has previously been reported in cattle during CG [54, 55, 56]. Furthermore, the increase in the expression of genes associated with cellular translational machinery and protein synthesis in animals undergoing CG in the current study was paralleled by an increase in the expression of genes involved in protein folding. Following peptide synthesis a polypeptide chain may acquire its biologically functional conformation through protein folding by for example chaperones or heat shock proteins [57]. At the same time that there was up-regulation of cellular translational machinery, a large number of genes with chaperone functionality involved in protein folding were also up-regulated (Table 4), suggesting a greater amount of cellular peptide synthesis during re-alimentation compared with at the end of dietary restriction in Period 1. Connor et al. [24] also reported greater expression of genes coding for proteins involved in protein folding. Protein folding genes commonly expressed between the current study and that of Connor et al. [24] included: *CCT5*, *CCT6A*, *DNAJB11*, *FKBP3*, *HSP90AA1*, *HSP90AB1*, *HSP90B1*, *HSPA5*, *HSPA8* and *HSPH1*. Overall genes differentially expressed in animals undergoing re-alimentation and CG compared to dietary restriction at the end of Period 1, suggest greater expression of genes involved in controlling cellular function and organisation as well as in protein synthesis. Greater organisation and functional capacity of cells during re-alimentation may allow for subsequent greater growth of this tissue, as previously described by Keogh et al. [9] during re-alimentation following a previous period of dietary restriction.

### Potential molecular biomarkers

The exploitation of the CG phenomenon is a common practise in beef production systems worldwide [1, 58]. However, although information on the physiological control of CG has been examined previously [3, 4, 5, 6, 7, 8, 21, 22], knowledge of the underlying molecular control regulating the expression of CG is lacking. Additionally, due to variation in animal response to both dietary restriction and subsequent CG, knowledge of the genetic basis for this trait is critical to the future effective exploitation of the trait. A greater understanding of the molecular mechanisms regulating the expression of CG may provide essential information to the discovery of DNA-based biomarkers which could be incorporated into genomic selection breeding programmes to select animals that display enhanced genetic potential for CG following prior dietary restriction. Furthermore, as CG is associated with an improvement in feed efficiency, differentially expressed genes identified in this study may contribute to breeding protocols for the selection of animals with improved feed efficiency. The large number of differentially expressed genes identified in response to CG in the current study may hold potential for subsequent identification of DNA-based biomarkers for the selection of CG and feed efficiency. From an evaluation of genes identified as differentially expressed within the current study (presented in S1, S2 and S3 Tables) and that of the hepatic based CG data of Connor et al. [24] and Keogh et al. [52] and the feed efficiency based data of Chen et al. [59], a number of genes were identified to be commonly expressed and followed the same direction of change between studies and may hold potential for further investigation in relation to their use as biomarkers for the selection of CG, these genes are listed in Table 5. The following genes: *DHRS3*, *COL1A1*, *SLC27A6*, *SPARC*, *TCEA3* and *VIM* are of particular interest as they were expressed during CG in the data presented in the current study as well as in two of the following datasets mentioned above [24, 52, 59]. Differential expression of these genes in different cohorts of animals and under different experimental conditions may imply a greater importance of these genes to these traits. Further investigation is warranted to determine potential polymorphisms in the DNA of these genes which may be associated with differences in potential to display CG.

**Table 5:** Genes identified as potential molecular biomarkers for use for the selection of compensatory growth.

Reference	Genes^1^
Connor et al. [24]	*ACSL5 ACSM1 ACTA2 ACTB ACTG1 ACTN1 ADH5 AHCY ALDH18A1 ALDH1A1 ARPC3 BLVRB BMP2K C1QB CALR CALU CC2D1A CCND1 CCT5 CCT6A CDC34 CEBPG CIB1 CKB CMBL COL4A1 COQ7 CPEB4 CPT2 CTSF CYP51A1 DAAM1 DCN DHRS3 DNAJB11 DUS1L DUSP6 EDC4 EDEM3 EEF1B2 ENO1 EXTL1 FAM131A FANCM FBXL4 FKBP3 FNBP4 GADD45A GNL3 Gtf2ird2 HINT1 HMGCR HMGCS1 HPDL HSP90AA1 HSP90AB1 HSP90B1 HSPA5 HSPA8 HSPB1 HSPH1 IL27RA KLF15 MGA MRPL17 MYL6 MYO1B MYO1E NAMPT NANS NDRG1 NDRG2 NDUFA4L2 NRIP1 NSDHL NUDCD2 ODC1 OTUD4 PDE7B PFKFB1 PGK1 PGRMC1 PHF21A PHGDH PLCB3 POLR21 POLR2L PON1 PPA1 PRKD2 PSAP PSAT1 RPL3 RPL32 RRP15 SARS SCUBE2 SEMA6B SLC12A7 SLC25A39 SLC27A6 SNRPD1 SOCS2 SP4 SPARC STAP2 TARS TCEA3 TIMELESS TRIM33 TTC9 TXN UBE4A VIM VRK2 XPC YWHAB ZC3H6 ZNHIT3*
Keogh et al. [52]	*ARL4A BCORL1 C10orf10 COL1A1 COL1A2 FBLN1 LNP1 MAST3 MGP NOV SELK SPARC TCEA3*
Chen et al. [59]	*CACNA1H CLEC3B CNN1 COL1A1 DHRS3 ETS2 FMOD IMPA2 KITLG LOXL1 MAOA MRPL12 MTCH2 N4BP1 NFATC2IP PSPH PYCR1 SH3PXD2A SLC27A6 SOD3 SPRY4 THBS1 TIMP1 TNKS1BP1 VIM*

^1^Genes were commonly expressed and showed the same direction of change in the current dataset and those of Connor et al. [24] and Keogh et al. [52] in relation to compensatory growth and Chen et al. [59] in relation to feed efficiency

## Conclusions

Following a period of dietary restriction animals were utilising body lipid stores, as evidenced through alterations in the expression of genes of the mitochondrial beta oxidation system. This was potentially necessary in order to meet basal energy requirements. Additionally a greater capacity for mitochondrial energy production efficiency was also apparent at the end of the restricted feeding regime. This emphasised a requirement to utilise energy more efficiently in times of restricted calorie intake. The findings of the current study indicate that the expression of compensatory growth during early re-alimentation may be due to greater cellular organisation and function which may be necessary to build up capacity within the tissue for subsequent accelerated growth and protein accretion. The greater capacity for mitochondrial efficiency observed during restricted feeding was not observed during CG, with an overall reduction in the expression of genes controlling mitochondrial energy production reported during CG. This indicates that mitochondrial efficiency does not contribute wholly to the occurrence of accelerated growth in CG in the current study. These data contribute to an improved understanding of the molecular control of the response to dietary restriction and subsequent re-alimentation and provide an insight into dietary nutrient efficiency at a cellular level. Furthermore, these differential gene expression profiles provide data which can be further integrated to form the basis of genomic biomarkers to aid the selection of cattle which are economically and environmentally more sustainable to produce.

## Supporting Information

S1 Table Genes differentially expressed in *M*. *longissimus dorsi* of Holstein Friesian bulls (n = 10) following a 120-day period of restricted feeding at the end of Period 1 relative to *ad libitum*-fed controls (n = 10) (DOCX)

S2 Table Genes differentially expressed in *M*. *longissimus dorsi* of Holstein Friesian bulls (n = 10) following a 15-day period of re-alimentation and compensatory growth in Period 2 relative to *ad libitum*-fed controls (n = 10) (DOCX)

S3 Table Genes commonly differentially expressed in *M*. *longissimus dorsi* of Holstein Friesian bulls following a 120-day period of dietary restriction at the end of Period 1 and during a subsequent re-alimentation and compensatory growth period of 15-days in Period 2 relative to *ad libitum*-fed controls (DOCX)

S4 Table Genes differentially expressed in *M*. *longissimus dorsi* of Holstein Friesian bulls (n = 10) following a 15-day period of re-alimentation and compensatory growth in Period 2 relative to animals fed a restricted diet for 120 days at the end of Period 1 (n = 10) (DOCX)
