# Supplementary material for: Effect of Dietary Restriction and Subsequent Re-Alimentation on the Transcriptional Profile of Bovine Skeletal Muscle
Source: PLoS One. 2016 Feb 12;11(2):e0149373. doi: 10.1371/journal.pone.0149373 (PMC4752344; doi:10.1371/journal.pone.0149373)
Supplement: S1 Table — (DOCX) [file pone.0149373.s001.docx]

**S1 Table.** Genes differentially expressed in *M. longissimus dorsi* of Holstein Friesian bulls (n = 10) following a 120-day period of restricted feeding at the end of Period 1 relative to *ad libitum*-fed controls (n = 10)

| Gene symbol | Gene name | Fold change^1^ | P value |
| --- | --- | --- | --- |
| *AAAS* | Achalasia, adrenocortical insufficiency, alacrimia | -1.603 | 2.02E-05 |
| *AASS* | Aminoadipate-semialdehyde synthase | 1.325 | 0.00896 |
| *ABCA1* | ATP-binding cassette, sub-family A (ABC1), member 1 | 1.705 | 0.0004 |
| *ABCA3* | ATP-binding cassette, sub-family A (ABC1), member 3 | -1.929 | 4.56E-05 |
| *ABCA5* | ATP-binding cassette, sub-family A (ABC1), member 5 | -1.284 | 0.01077 |
| *ABCB8* | ATP-binding cassette, sub-family B (MDR/TAP), member 8 | -1.455 | 0.00024 |
| *ABCC8* | ATP-binding cassette, sub-family C (CFTR/MRP), member 8 | -1.669 | 0.00068 |
| *ABCG1* | ATP-binding cassette, sub-family G (WHITE), member 1 | 3.015 | 1.37E-10 |
| *ABHD11* | Abhydrolase domain containing 11 | -1.661 | 9.74E-06 |
| *ABHD14B* | Abhydrolase domain containing 14B | -1.34 | 0.01001 |
| *ABLIM2* | Actin binding LIM protein family, member 2 | 1.342 | 0.00373 |
| *ABR* | Active BCR-related | -1.266 | 0.00316 |
| *ACAA1* | Acetyl-CoA acyltransferase 1 | 1.281 | 0.00225 |
| *ACACA* | Acetyl-CoA carboxylase alpha | -2.021 | 0.00681 |
| *ACAD10* | Acyl-CoA dehydrogenase family, member 10 | 1.365 | 4.11E-05 |
| *ACADL* | Acyl-CoA dehydrogenase, long chain | -1.317 | 0.00404 |
| *ACADM* | Acyl-CoA dehydrogenase, C-4 to C-12 straight chain | 1.335 | 0.00035 |
| *ACBD5* | Acyl-CoA binding domain containing 5 | -1.362 | 0.00084 |
| *ACCS* | 1-aminocyclopropane-1-carboxylate synthase homolog (Arabidopsis)(non-functional) | -1.647 | 0.00037 |
| *ACHE* | Acetylcholinesterase (Yt blood group) | 1.759 | 0.00544 |
| *ACKR3* | Atypical chemokine receptor 3 | -1.357 | 0.00818 |
| *ACLY* | ATP citrate lyase | -3.676 | 1.95E-06 |
| *ACN9* | ACN9 homolog (S. cerevisiae) | 1.474 | 0.00101 |
| *ACO2* | Aconitase 2, mitochondrial | 1.38 | 0.00014 |
| *ACOX1* | Acyl-CoA oxidase 1, palmitoyl | 1.428 | 0.00489 |
| *ACOX2* | Acyl-CoA oxidase 2, branched chain | 1.495 | 8.12E-07 |
| *ACSL1* | Acyl-CoA synthetase long-chain family member 1 | -1.284 | 0.0012 |
| *ACSM1* | Acyl-CoA synthetase medium-chain family member 1 | -3.262 | 0.01295 |
| *ACTC1* | Actin, alpha, cardiac muscle 1 | -1.98 | 0.00015 |
| *ACTN2* | Actinin, alpha 2 | 1.382 | 0.00022 |
| *ACTR3* | ARP3 actin-related protein 3 homolog (yeast) | -1.312 | 0.00108 |
| *ADA* | Adenosine deaminase | -1.32 | 0.00484 |
| *ADAL* | Adenosine deaminase-like | -1.347 | 0.00282 |
| *ADAM19* | ADAM metallopeptidase domain 19 | -1.327 | 0.01198 |
| *ADAMTS2* | ADAM metallopeptidase with thrombospondin type 1 motif, 2 | -1.388 | 0.01021 |
| *ADAMTS9* | ADAM metallopeptidase with thrombospondin type 1 motif, 9 | -1.338 | 0.00675 |
| *ADAMTSL2* | ADAMTS-like 2 | -1.726 | 0.00011 |
| *ADCY2* | Adenylate cyclase 2 (brain) | 1.387 | 6.54E-07 |
| *ADCY6* | Adenylate cyclase 6 | -1.383 | 0.01168 |
| *ADD1* | Adducin 1 (alpha) | 1.291 | 0.00014 |
| *ADH5* | Alcohol dehydrogenase 5 (class III), chi polypeptide | -1.289 | 0.00183 |
| *ADHFE1* | Alcohol dehydrogenase, iron containing, 1 | 1.544 | 1.13E-05 |
| *ADRM1* | Adhesion regulating molecule 1 | 1.279 | 0.00893 |
| *ADSSL1* | Adenylosuccinate synthase like 1 | -1.25 | 0.00942 |
| *AEBP1* | AE binding protein 1 | -2.192 | 0.00327 |
| *AFF1* | AF4/FMR2 family, member 1 | 1.576 | 0.0002 |
| *AGMAT* | Agmatine ureohydrolase (agmatinase) | 1.488 | 0.00408 |
| *AGPAT6* | 1-acylglycerol-3-phosphate O-acyltransferase 6 | 1.383 | 1.20E-05 |
| *AGRN* | Agrin | -1.52 | 0.00034 |
| *AGTPBP1* | ATP/GTP binding protein 1 | -2.225 | 0.00068 |
| *AHCY* | Adenosylhomocysteinase | -1.255 | 0.00523 |
| *AHDC1* | AT hook, DNA binding motif, containing 1 | -1.304 | 0.00567 |
| *AIFM1* | Apoptosis-inducing factor, mitochondrion-associated, 1 | 1.26 | 0.00087 |
| *AIP* | Aryl hydrocarbon receptor interacting protein | -1.322 | 0.00223 |
| *AKAP1* | A kinase (PRKA) anchor protein 1 | 1.266 | 0.00237 |
| *AKAP5* | A kinase (PRKA) anchor protein 5 | -1.636 | 0.00664 |
| *AKAP7* | A kinase (PRKA) anchor protein 7 | 1.627 | 0.00078 |
| *AKTIP* | AKT interacting protein | 1.297 | 0.00017 |
| *ALDH1A1* | Aldehyde dehydrogenase 1 family, member A1 | -1.387 | 0.00171 |
| *ALDOA* | Aldolase A, fructose-bisphosphate | 1.387 | 0.00025 |
| *ALKBH3* | AlkB, alkylation repair homolog 3 (E. coli) | 1.299 | 7.15E-05 |
| *ALYREF* | Aly/REF export factor | 1.252 | 0.00274 |
| *AMOT* | Angiomotin | 1.45 | 5.42E-06 |
| *AMOTL1* | Angiomotin like 1 | 1.253 | 0.00315 |
| *AMPD2* | Adenosine monophosphate deaminase 2 | -1.494 | 0.01247 |
| *ANAPC4* | Anaphase promoting complex subunit 4 | -1.473 | 0.00011 |
| *ANGPT1* | Angiopoietin 1 | -1.699 | 0.00129 |
| *ANGPTL4* | Angiopoietin-like 4 | -2.384 | 0.00815 |
| *ANKRA2* | Ankyrin repeat, family A (RFXANK-like), 2 | 1.396 | 0.00014 |
| *ANKRD13B* | Ankyrin repeat domain 13B | -1.749 | 4.61E-06 |
| *ANKRD2* | Ankyrin repeat domain 2 (stretch responsive muscle) | 1.429 | 0.01201 |
| *ANKRD50* | Ankyrin repeat domain 50 | -1.458 | 0.01428 |
| *ANKRD9* | Ankyrin repeat domain 9 | 1.321 | 0.01205 |
| *ANTXR2* | Anthrax toxin receptor 2 | -1.281 | 0.00759 |
| *ANXA1* | Annexin A1 | -1.572 | 0.01197 |
| *ANXA7* | Annexin A7 | 1.303 | 0.01264 |
| *AP2A1* | Adaptor-related protein complex 2, alpha 1 subunit | 1.266 | 0.00273 |
| *AP3B1* | Adaptor-related protein complex 3, beta 1 subunit | -1.265 | 0.00501 |
| *AP3M2* | Adaptor-related protein complex 3, mu 2 subunit | 1.338 | 0.00072 |
| *AP4B1* | Adaptor-related protein complex 4, beta 1 subunit | -1.289 | 0.00373 |
| *AP4S1* | Adaptor-related protein complex 4, sigma 1 subunit | 1.301 | 0.00258 |
| *APCDD1* | Adenomatosis polyposis coli down-regulated 1 | 1.752 | 8.89E-07 |
| *APITD1* | Apoptosis-inducing, TAF9-like domain 1 | -1.394 | 0.00203 |
| *APMAP* | Adipocyte plasma membrane associated protein | 1.627 | 5.09E-06 |
| *APOD* | Apolipoprotein D | 1.655 | 1.68E-05 |
| *AQP3* | Aquaporin 3 (Gill blood group) | 1.525 | 0.00515 |
| *AQP4* | Aquaporin 4 | 1.409 | 0.00754 |
| *ARF1* | ADP-ribosylation factor 1 | -1.283 | 0.00185 |
| *ARFGAP2* | ADP-ribosylation factor GTPase activating protein 2 | 1.413 | 6.17E-05 |
| *ARFGEF1* | ADP-ribosylation factor guanine nucleotide-exchange factor 1 (brefeldin A-inhibited) | -1.401 | 0.00288 |
| *ARHGAP18* | Rho GTPase activating protein 18 | -1.651 | 1.05E-05 |
| *ARHGEF40* | Rho guanine nucleotide exchange factor (GEF) 40 | -1.444 | 0.00197 |
| *ARID5B* | AT rich interactive domain 5B (MRF1-like) | 1.446 | 0.01231 |
| *ARL13B* | ADP-ribosylation factor-like 13B | -1.426 | 0.00771 |
| *ARL6* | ADP-ribosylation factor-like 6 | -1.906 | 1.69E-05 |
| *ARRDC2* | Arrestin domain containing 2 | 1.836 | 0.01098 |
| *ARSB* | Arylsulfatase B | -1.717 | 0.00034 |
| *ART5* | ADP-ribosyltransferase 5 | 2.166 | 0.00339 |
| *ASB12* | Ankyrin repeat and SOCS box containing 12 | -1.357 | 0.00012 |
| *ASB14* | Ankyrin repeat and SOCS box containing 14 | -1.314 | 0.00094 |
| *ASB8* | Ankyrin repeat and SOCS box containing 8 | 1.26 | 0.00236 |
| *ASPA* | Aspartoacylase | -1.952 | 0.00545 |
| *ASPN* | Asporin | -2.255 | 0.00013 |
| *ASS1* | Argininosuccinate synthase 1 | 2.302 | 6.69E-12 |
| *ATG4A* | Autophagy related 4A, cysteine peptidase | -1.318 | 0.00029 |
| *ATG9A* | Autophagy related 9A | 1.346 | 2.77E-05 |
| *ATL2* | Atlastin GTPase 2 | -1.386 | 0.0002 |
| *ATMIN* | ATM interactor | 1.355 | 0.00797 |
| *ATP1A4* | ATPase, Na+/K+ transporting, alpha 4 polypeptide | 2.09 | 2.56E-06 |
| *ATP1B3* | ATPase, Na+/K+ transporting, beta 3 polypeptide | -1.391 | 0.00285 |
| *ATP1B4* | ATPase, Na+/K+ transporting, beta 4 polypeptide | 1.365 | 0.01461 |
| *ATP2B2* | ATPase, Ca++ transporting, plasma membrane 2 | -2.109 | 1.32E-07 |
| *ATP5B* | ATP synthase, H+ transporting, mitochondrial F1 complex, beta polypeptide | 1.359 | 1.72E-05 |
| *ATP5C1* | ATP synthase, H+ transporting, mitochondrial F1 complex, gamma polypeptide 1 | 1.366 | 6.09E-06 |
| *ATP5D* | ATP synthase, H+ transporting, mitochondrial F1 complex, delta subunit | 1.389 | 5.82E-06 |
| *ATP5G1* | ATP synthase, H+ transporting, mitochondrial Fo complex, subunit C1 (subunit 9) | 1.359 | 0.00255 |
| *ATP5G2* | ATP synthase, H+ transporting, mitochondrial Fo complex, subunit C2 (subunit 9) | 1.296 | 7.54E-05 |
| *Atp5h* | ATP synthase, H+ transporting, mitochondrial F0 complex, subunit D | 1.342 | 9.81E-05 |
| *ATP7A* | ATPase, Cu++ transporting, alpha polypeptide | 2.264 | 0.00456 |
| *ATPIF1* | ATPase inhibitory factor 1 | 1.278 | 0.00964 |
| *ATXN10* | Ataxin 10 | -1.391 | 0.00012 |
| *AVEN* | Apoptosis, caspase activation inhibitor | -1.471 | 2.09E-06 |
| *B3GALT4* | UDP-Gal:betaGlcNAc beta 1,3-galactosyltransferase, polypeptide 4 | 1.275 | 0.0073 |
| *B9D2* | B9 protein domain 2 | -1.683 | 6.25E-07 |
| *BAG6* | BCL2-associated athanogene 6 | 1.356 | 0.00011 |
| *BAIAP2L1* | BAI1-associated protein 2-like 1 | 1.762 | 0.00027 |
| *BANK1* | B-cell scaffold protein with ankyrin repeats 1 | 2.699 | 0.00118 |
| *BBS12* | Bardet-Biedl syndrome 12 | -1.361 | 0.00545 |
| *BBS4* | Bardet-Biedl syndrome 4 | -1.295 | 0.00303 |
| *BCAT2* | Branched chain amino-acid transaminase 2, mitochondrial | -1.458 | 0.01174 |
| *BCKDHA* | Branched chain keto acid dehydrogenase E1, alpha polypeptide | 1.279 | 0.00293 |
| *BCL2L1* | BCL2-like 1 | 1.402 | 0.00101 |
| *BCL6* | B-cell CLL/lymphoma 6 | -2.108 | 1.05E-05 |
| *BCORL1* | BCL6 corepressor-like 1 | -1.489 | 0.00025 |
| *BEND5* | BEN domain containing 5 | 1.477 | 0.00045 |
| *BGN* | Biglycan | -1.403 | 0.00591 |
| *BIRC5* | Baculoviral IAP repeat containing 5 | -2.074 | 1.77E-07 |
| *BLCAP* | Bladder cancer associated protein | 1.54 | 1.14E-07 |
| *BLOC1S2* | Biogenesis of lysosomal organelles complex-1, subunit 2 | -1.339 | 0.00477 |
| *BLVRA* | Biliverdin reductase A | 1.302 | 0.00097 |
| *BOC* | BOC cell adhesion associated, oncogene regulated | 1.473 | 0.00546 |
| *BOLA1* | BolA family member 1 | 1.307 | 0.00063 |
| *BOLL* | Boule-like RNA-binding protein | -1.723 | 0.00014 |
| *BPHL* | Biphenyl hydrolase-like (serine hydrolase) | 1.415 | 0.00358 |
| *BRAP* | BRCA1 associated protein | -1.254 | 0.0056 |
| *BRCA1* | Breast cancer 1, early onset | -1.618 | 0.00049 |
| *BRMS1L* | Breast cancer metastasis-suppressor 1-like | -1.393 | 0.00607 |
| *BSCL2* | Berardinelli-Seip congenital lipodystrophy 2 (seipin) | 1.292 | 0.00446 |
| *BTBD11* | BTB (POZ) domain containing 11 | -2.085 | 4.43E-05 |
| *BTD* | Biotinidase | 1.49 | 0.00592 |
| *BTG2* | BTG family, member 2 | -3.317 | 5.22E-10 |
| *BTNL9* | Butyrophilin-like 9 | 1.672 | 0.00146 |
| *C10orf10* | Chromosome 10 open reading frame 10 | 1.616 | 2.05E-05 |
| *C10orf32* | Chromosome 10 open reading frame 32 | -1.354 | 0.00828 |
| *C10orf71* | Chromosome 10 open reading frame 71 | 1.26 | 0.00438 |
| *C11orf54* | Chromosome 11 open reading frame 54 | -1.481 | 0.00257 |
| *C11orf71* | Chromosome 11 open reading frame 71 | 1.696 | 1.30E-05 |
| *C15orf27* | Chromosome 15 open reading frame 27 | 1.298 | 0.00247 |
| *C18orf21* | Chromosome 18 open reading frame 21 | 1.575 | 0.00073 |
| *C1qtnf6* | C1q and tumor necrosis factor related protein 6 | -2.476 | 4.56E-05 |
| *C2orf76* | Chromosome 2 open reading frame 76 | -1.409 | 0.01118 |
| *C3orf18* | Chromosome 3 open reading frame 18 | 1.702 | 3.19E-07 |
| *C3orf62* | Chromosome 3 open reading frame 62 | 1.29 | 0.01135 |
| *C4orf27* | Chromosome 4 open reading frame 27 | 1.251 | 0.00743 |
| *C9orf40* | Chromosome 9 open reading frame 40 | -1.343 | 0.00121 |
| *C9orf41* | Chromosome 9 open reading frame 41 | -1.59 | 0.00015 |
| *C9orf50* | Chromosome 9 open reading frame 50 | 1.318 | 0.00538 |
| *C9orf72* | Chromosome 9 open reading frame 72 | -1.609 | 8.42E-05 |
| *CA11* | Carbonic anhydrase XI | -1.737 | 0.00025 |
| *CA14* | Carbonic anhydrase XIV | 1.334 | 0.00086 |
| *CA2* | Carbonic anhydrase II | -1.523 | 0.00545 |
| *CA3* | Carbonic anhydrase III, muscle specific | -1.363 | 0.00036 |
| *CACNA1S* | Calcium channel, voltage-dependent, L type, alpha 1S subunit | 1.356 | 0.0017 |
| *CALCOCO1* | Calcium binding and coiled-coil domain 1 | 1.301 | 0.00268 |
| *Cald1* | Caldesmon 1 | -1.267 | 0.00685 |
| *CAMK1* | Calcium/calmodulin-dependent protein kinase I | -1.498 | 0.00913 |
| *CAMK2A* | Calcium/calmodulin-dependent protein kinase II alpha | 1.267 | 0.00924 |
| *CARD11* | Caspase recruitment domain family, member 11 | -1.983 | 0.00282 |
| *CARNS1* | Carnosine synthase 1 | -1.301 | 0.0011 |
| *CASP4* | Caspase 4, apoptosis-related cysteine peptidase | -1.605 | 0.00073 |
| *CASP8AP2* | Caspase 8 associated protein 2 | -1.596 | 0.00015 |
| *CAV3* | Caveolin 3 | 1.361 | 0.00017 |
| *CBR4* | Carbonyl reductase 4 | 1.341 | 0.01044 |
| *CBWD1* | COBW domain containing 1 | -1.46 | 3.79E-06 |
| *CBX7* | Chromobox homolog 7 | 1.406 | 0.00232 |
| *CCDC107* | Coiled-coil domain containing 107 | 1.47 | 7.49E-05 |
| *CCDC117* | Coiled-coil domain containing 117 | -1.352 | 0.0036 |
| *CCDC3* | Coiled-coil domain containing 3 | -2.53 | 0.00508 |
| *CCDC41* | Coiled-coil domain containing 41 | -1.693 | 0.00408 |
| *CCDC43* | Coiled-coil domain containing 43 | 1.259 | 0.00427 |
| *CCDC6* | Coiled-coil domain containing 6 | -1.271 | 0.01373 |
| *CCDC69* | Coiled-coil domain containing 69 | -1.454 | 2.09E-05 |
| *CCDC8* | Coiled-coil domain containing 8 | 1.421 | 0.00019 |
| *CCDC80* | Coiled-coil domain containing 80 | -1.787 | 2.32E-08 |
| *CCDC86* | Coiled-coil domain containing 86 | -1.513 | 2.87E-07 |
| *CCDC88A* | Coiled-coil domain containing 88A | -1.593 | 0.0024 |
| *CCNE1* | Cyclin E1 | -1.502 | 0.00284 |
| *CCNE2* | Cyclin E2 | -1.812 | 0.00234 |
| *CCP110* | Centriolar coiled coil protein 110kDa | -1.637 | 0.00235 |
| *CCRL2* | Chemokine (C-C motif) receptor-like 2 | 1.994 | 1.72E-08 |
| *CD109* | CD109 molecule | 1.697 | 0.00512 |
| *CD44* | CD44 molecule (Indian blood group) | -1.652 | 0.00291 |
| *CDC14A* | Cell division cycle 14A | -1.443 | 0.01137 |
| *CDC23* | Cell division cycle 23 | -1.261 | 0.00316 |
| *CDC26* | Cell division cycle 26 | -1.83 | 2.41E-08 |
| *CDC42EP2* | CDC42 effector protein (Rho GTPase binding) 2 | -1.492 | 0.00061 |
| *CDC7* | Cell division cycle 7 | -1.678 | 0.00115 |
| *CDH22* | Cadherin 22, type 2 | 2.397 | 2.27E-07 |
| *CDIP1* | Cell death-inducing p53 target 1 | 1.383 | 8.99E-05 |
| *CDK20* | Cyclin-dependent kinase 20 | 1.358 | 0.00407 |
| *CDK4* | Cyclin-dependent kinase 4 | 1.279 | 0.00132 |
| *CDK7* | Cyclin-dependent kinase 7 | -1.319 | 0.00586 |
| *CDKN2C* | Cyclin-dependent kinase inhibitor 2C (p18, inhibits CDK4) | -1.253 | 0.00552 |
| *CEP104* | Centrosomal protein 104kDa | -1.276 | 0.00533 |
| *CEP250* | Centrosomal protein 250kDa | -1.394 | 0.00391 |
| *CERS1* | Ceramide synthase 1 | 1.405 | 8.55E-05 |
| *CHCHD10* | Coiled-coil-helix-coiled-coil-helix domain containing 10 | 1.385 | 0.00137 |
| *CHCHD3* | Coiled-coil-helix-coiled-coil-helix domain containing 3 | 1.297 | 4.18E-05 |
| *CHD3* | Chromodomain helicase DNA binding protein 3 | -1.978 | 2.52E-16 |
| *CHKA* | Choline kinase alpha | -1.583 | 0.0097 |
| *CHODL* | Chondrolectin | -1.585 | 0.00027 |
| *CHORDC1* | Cysteine and histidine-rich domain (CHORD) containing 1 | -1.643 | 5.19E-09 |
| *CHP1* | Calcineurin-like EF-hand protein 1 | -1.365 | 0.00026 |
| *CHPT1* | Choline phosphotransferase 1 | -1.418 | 0.00266 |
| *CHRDL2* | Chordin-like 2 | -2.182 | 0.00023 |
| *CHST15* | Carbohydrate (N-acetylgalactosamine 4-sulfate 6-O) sulfotransferase 15 | -1.488 | 0.00365 |
| *CHST2* | Carbohydrate (N-acetylglucosamine-6-O) sulfotransferase 2 | -1.388 | 0.00408 |
| *CIDEC* | Cell death-inducing DFFA-like effector c | -3.852 | 0.00616 |
| *CIITA* | Class II, major histocompatibility complex, transactivator | 1.552 | 0.00692 |
| *CIRBP* | Cold inducible RNA binding protein | 2.362 | 1.49E-13 |
| *CITED1* | Cbp/p300-interacting transactivator, with Glu/Asp-rich carboxy-terminal domain, 1 | 2.031 | 6.90E-06 |
| *CITED2* | Cbp/p300-interacting transactivator, with Glu/Asp-rich carboxy-terminal domain, 2 | 1.336 | 0.00756 |
| *CKB* | Creatine kinase, brain | -2.724 | 0.00029 |
| *CKM* | Creatine kinase, muscle | 1.295 | 0.00291 |
| *CLEC3B* | C-type lectin domain family 3, member B | 1.267 | 0.00178 |
| *CLIC4* | Chloride intracellular channel 4 | -1.343 | 0.00054 |
| *CLIP1* | CAP-GLY domain containing linker protein 1 | -1.255 | 0.01004 |
| *CLPTM1* | Cleft lip and palate associated transmembrane protein 1 | 1.372 | 3.32E-05 |
| *Clu* | Clusterin | -1.845 | 0.00025 |
| *CLUH* | Clustered mitochondria (cluA/CLU1) homolog | 1.387 | 8.32E-05 |
| *CMC4* | C-x(9)-C motif containing 4 | 1.6 | 4.64E-07 |
| *CNBP* | CCHC-type zinc finger, nucleic acid binding protein | 1.363 | 1.63E-06 |
| *CNKSR3* | CNKSR family member 3 | -1.647 | 3.99E-06 |
| *CNN1* | Ccalponin 1, basic, smooth muscle | -1.709 | 8.02E-05 |
| *CNP* | 2',3'-cyclic nucleotide 3' phosphodiesterase | -1.598 | 7.64E-06 |
| *CNPY4* | Canopy FGF signaling regulator 4 | -1.253 | 0.00527 |
| *COL15A1* | Collagen, type XV, alpha 1 | -1.267 | 0.00586 |
| *COL1A2* | Collagen, type I, alpha 2 | -1.835 | 0.00049 |
| *COL3A1* | Collagen, type III, alpha 1 | -1.599 | 0.00026 |
| *COL4A1* | Collagen, type IV, alpha 1 | -1.571 | 1.60E-05 |
| *COL4A2* | Collagen, type IV, alpha 2 | -1.337 | 0.00689 |
| *COL4A5* | Collagen, type IV, alpha 5 | -1.486 | 0.00434 |
| *COL5A2* | Collagen, type V, alpha 2 | -1.811 | 3.74E-09 |
| *COL5A3* | Collagen, type V, alpha 3 | -1.647 | 5.76E-05 |
| *COMMD7* | COMM domain containing 7 | -1.332 | 0.00054 |
| *COPRS* | Coordinator of PRMT5, differentiation stimulator | 1.518 | 1.59E-05 |
| *COQ10B* | Coenzyme Q10 homolog B (S. cerevisiae) | -1.777 | 0.00085 |
| *COX10* | COX10 heme A:farnesyltransferase cytochrome c oxidase assembly factor | 1.254 | 0.00341 |
| *COX14* | Cytochrome c oxidase assembly homolog 14 (S. cerevisiae) | 1.28 | 0.0005 |
| *COX4I1* | Cytochrome c oxidase subunit IV isoform 1 | 1.321 | 0.00161 |
| *COX5B* | Cytochrome c oxidase subunit Vb | 1.476 | 4.73E-07 |
| *COX6B1* | Cytochrome c oxidase subunit VIb polypeptide 1 (ubiquitous) | 1.294 | 0.00434 |
| *COX7A1* | Cytochrome c oxidase subunit VIIa polypeptide 1 (muscle) | 1.383 | 6.62E-06 |
| *CPED1* | Cadherin-like and PC-esterase domain containing 1 | -2.008 | 7.52E-09 |
| *CPSF6* | Cleavage and polyadenylation specific factor 6, 68kDa | 1.446 | 1.55E-06 |
| *CPT1A* | Carnitine palmitoyltransferase 1A (liver) | -2.391 | 1.85E-05 |
| *CPT1B* | Carnitine palmitoyltransferase 1B (muscle) | 1.465 | 8.59E-05 |
| *CPXM1* | Carboxypeptidase X (M14 family), member 1 | -1.503 | 0.00861 |
| *CRISPLD2* | Cysteine-rich secretory protein LCCL domain containing 2 | -1.414 | 1.57E-05 |
| *CRYAB* | Crystallin, alpha B | -1.299 | 0.0008 |
| *CS* | Citrate synthase | 1.315 | 0.00032 |
| *CSRP3* | Cysteine and glycine-rich protein 3 (cardiac LIM protein) | 2.657 | 2.50E-08 |
| *CSTF2* | Cleavage stimulation factor, 3' pre-RNA, subunit 2, 64kDa | -1.421 | 0.00122 |
| *CSTF3* | Cleavage stimulation factor, 3' pre-RNA, subunit 3, 77kDa | 1.282 | 0.00561 |
| *CTGF* | Connective tissue growth factor | -1.782 | 0.00088 |
| *CTNNBIP1* | Catenin, beta interacting protein 1 | 1.902 | 1.29E-06 |
| *CTNNBL1* | Catenin, beta like 1 | 1.267 | 0.00181 |
| *CTPS1* | CTP synthase 1 | -1.75 | 3.04E-06 |
| *CTSC* | Cathepsin C | 1.348 | 0.00333 |
| *CTSF* | Cathepsin F | 1.569 | 2.09E-09 |
| *CTSS* | Cathepsin S | -1.368 | 0.01444 |
| *CXCR4* | Chemokine (C-X-C motif) receptor 4 | -1.473 | 0.00162 |
| *CYB5R1* | Cytochrome b5 reductase 1 | 1.424 | 1.06E-05 |
| *CYB5R2* | Cytochrome b5 reductase 2 | -1.617 | 0.00925 |
| *CYB5R4* | Cytochrome b5 reductase 4 | -1.332 | 0.0103 |
| *CYC1* | Cytochrome c-1 | 1.466 | 1.52E-07 |
| *CYP1A1* | Cytochrome P450, family 1, subfamily A, polypeptide 1 | 2.017 | 5.96E-11 |
| *D2HGDH* | D-2-hydroxyglutarate dehydrogenase | 1.377 | 0.00011 |
| *DAB2* | Dab, mitogen-responsive phosphoprotein, homolog 2 (Drosophila) | -1.354 | 0.01147 |
| *DAG1* | Dystroglycan 1 (dystrophin-associated glycoprotein 1) | 1.267 | 0.0086 |
| *DAPK3* | Death-associated protein kinase 3 | 1.266 | 0.00884 |
| *DARS2* | Aspartyl-tRNA synthetase 2, mitochondrial | -1.332 | 0.01349 |
| *DBI* | Diazepam binding inhibitor (GABA receptor modulator, acyl-CoA binding protein) | -1.506 | 4.44E-07 |
| *DCAF15* | DDB1 and CUL4 associated factor 15 | 1.379 | 0.00239 |
| *DDC* | Dopa decarboxylase (aromatic L-amino acid decarboxylase) | -1.775 | 0.00054 |
| *DDHD2* | DDHD domain containing 2 | -1.369 | 0.00025 |
| *DDIT3* | DNA-damage-inducible transcript 3 | -1.364 | 0.00914 |
| *DDIT4L* | DNA-damage-inducible transcript 4-like | 1.385 | 0.01 |
| *DDO* | D-aspartate oxidase | 1.44 | 0.00178 |
| *DDX25* | DEAD (Asp-Glu-Ala-Asp) box helicase 25 | -2.588 | 6.40E-08 |
| *DDX28* | DEAD (Asp-Glu-Ala-Asp) box polypeptide 28 | 1.299 | 0.00185 |
| *DDX5* | DEAD (Asp-Glu-Ala-Asp) box helicase 5 | -1.335 | 0.00643 |
| *DDX50* | DEAD (Asp-Glu-Ala-Asp) box polypeptide 50 | -1.266 | 0.01118 |
| *DEDD* | Death effector domain containing | 1.301 | 0.00134 |
| *DES* | Desmin | 1.57 | 6.29E-07 |
| *DFFA* | DNA fragmentation factor, 45kDa, alpha polypeptide | -1.35 | 0.0019 |
| *DHRS3* | Dehydrogenase/reductase (SDR family) member 3 | -1.389 | 0.00954 |
| *DHTKD1* | Dehydrogenase E1 and transketolase domain containing 1 | -1.467 | 5.48E-06 |
| *DIRAS1* | DIRAS family, GTP-binding RAS-like 1 | 1.715 | 0.00061 |
| *DIXDC1* | DIX domain containing 1 | 1.312 | 0.01132 |
| *DKK2* | Dickkopf WNT signaling pathway inhibitor 2 | -2.639 | 0.0002 |
| *DLST* | Dihydrolipoamide S-succinyltransferase (E2 component of 2-oxo-glutarate complex) | 1.273 | 0.00041 |
| *DNAJA1* | DnaJ (Hsp40) homolog, subfamily A, member 1 | -1.637 | 3.55E-05 |
| *DNAJA4* | DnaJ (Hsp40) homolog, subfamily A, member 4 | -1.986 | 8.31E-14 |
| *DNAJC12* | DnaJ (Hsp40) homolog, subfamily C, member 12 | -1.672 | 0.00084 |
| *DNAJC21* | DnaJ (Hsp40) homolog, subfamily C, member 21 | -1.316 | 0.0009 |
| *DNAJC25* | DnaJ (Hsp40) homolog, subfamily C , member 25 | -1.394 | 0.01165 |
| *DNAJC4* | DnaJ (Hsp40) homolog, subfamily C, member 4 | 1.341 | 0.00124 |
| *DNM1L* | Dynamin 1-like | -1.368 | 0.00083 |
| *DNPEP* | Aspartyl aminopeptidase | 1.285 | 0.00063 |
| *DOCK1* | Dedicator of cytokinesis 1 | -1.256 | 0.00241 |
| *DOK4* | Docking protein 4 | 1.702 | 1.27E-05 |
| *DPAGT1* | Dolichyl-phosphate (UDP-N-acetylglucosamine) N-acetylglucosaminephosphotransferase 1 | -1.25 | 0.00947 |
| *DPT* | Dermatopontin | -1.346 | 0.00859 |
| *DPY19L1* | Dpy-19-like 1 (C. elegans) | -1.688 | 0.00071 |
| *DPYSL3* | Dihydropyrimidinase-like 3 | -2.328 | 2.52E-08 |
| *DSG4* | Desmoglein 4 | 1.572 | 0.01039 |
| *DSTN* | Destrin (actin depolymerizing factor) | -1.303 | 0.00272 |
| *DTD1* | D-tyrosyl-tRNA deacylase 1 | 1.353 | 0.00058 |
| *DTNBP1* | Dystrobrevin binding protein 1 | 1.293 | 0.0003 |
| *DUSP1* | Dual specificity phosphatase 1 | -1.788 | 5.88E-09 |
| *DUSP10* | Dual specificity phosphatase 10 | -1.331 | 0.00197 |
| *DUSP14* | Dual specificity phosphatase 14 | 1.289 | 0.00862 |
| *DUSP16* | Dual specificity phosphatase 16 | -1.371 | 0.0029 |
| *DUSP26* | Dual specificity phosphatase 26 (putative) | 1.679 | 2.64E-08 |
| *DYNC1LI2* | Dynein, cytoplasmic 1, light intermediate chain 2 | -1.353 | 0.0004 |
| *DYNLL1* | Dynein, light chain, LC8-type 1 | -2.097 | 2.80E-08 |
| *E2F3* | E2F transcription factor 3 | -1.583 | 0.00074 |
| *EBNA1BP2* | EBNA1 binding protein 2 | -1.324 | 0.00029 |
| *ECHDC1* | Enoyl CoA hydratase domain containing 1 | -1.318 | 0.0002 |
| *ECHDC2* | Enoyl CoA hydratase domain containing 2 | 1.39 | 0.01052 |
| *ECHDC3* | Enoyl CoA hydratase domain containing 3 | 1.46 | 0.00072 |
| *ECI2* | Enoyl-CoA delta isomerase 2 | 1.386 | 4.10E-05 |
| *ECM2* | Extracellular matrix protein 2, female organ and adipocyte specific | -1.566 | 0.00063 |
| *ECSIT* | ECSIT signalling integrator | 1.354 | 0.00374 |
| *EEFSEC* | Eukaryotic elongation factor, selenocysteine-tRNA-specific | 1.395 | 0.00208 |
| *EGLN3* | Egl-9 family hypoxia-inducible factor 3 | -1.665 | 0.00069 |
| *EGR1* | Early growth response 1 | -2.135 | 0.00415 |
| *EHBP1L1* | EH domain binding protein 1-like 1 | -1.271 | 0.00041 |
| *EIF3B* | Eukaryotic translation initiation factor 3, subunit B | 1.279 | 0.00035 |
| *EIF3F* | Eukaryotic translation initiation factor 3, subunit F | 1.262 | 0.00017 |
| *EIF4B* | Eukaryotic translation initiation factor 4B | 1.432 | 5.18E-08 |
| *EIF4E3* | Eukaryotic translation initiation factor 4E family member 3 | 1.494 | 8.39E-07 |
| *EIF4EBP1* | Eukaryotic translation initiation factor 4E binding protein 1 | 2.175 | 5.87E-10 |
| *EIF4G3* | Eukaryotic translation initiation factor 4 gamma, 3 | -1.355 | 0.00011 |
| *ELL2* | Elongation factor, RNA polymerase II, 2 | -1.486 | 0.00021 |
| *ELOVL5* | ELOVL fatty acid elongase 5 | -2.425 | 0.00053 |
| *ELOVL6* | ELOVL fatty acid elongase 6 | -9.547 | 1.70E-07 |
| *EMC3* | ER membrane protein complex subunit 3 | 1.256 | 0.00062 |
| *ENO1* | Enolase 1, (alpha) | -1.36 | 5.21E-05 |
| *ENO3* | Enolase 3 (beta, muscle) | 1.32 | 0.00102 |
| *ENTPD5* | Ectonucleoside triphosphate diphosphohydrolase 5 | -1.423 | 0.00676 |
| *ERAP2* | Endoplasmic reticulum aminopeptidase 2 | -2.069 | 0.00161 |
| *ESCO1* | Establishment of sister chromatid cohesion N-acetyltransferase 1 | -2.014 | 6.62E-05 |
| *ESRRB* | Estrogen-related receptor beta | 1.51 | 0.00536 |
| *ETFB* | Electron-transfer-flavoprotein, beta polypeptide | 1.307 | 0.0126 |
| *ETS2* | V-ets avian erythroblastosis virus E26 oncogene homolog 2 | -1.501 | 0.00023 |
| *EVC2* | Ellis van Creveld syndrome 2 | -1.518 | 0.00796 |
| *EXOC1* | Exocyst complex component 1 | -1.26 | 0.00452 |
| *EXTL1* | Exostosin-like glycosyltransferase 1 | -2.28 | 1.17E-10 |
| *EYA1* | Eyes absent homolog 1 (Drosophila) | -1.312 | 0.00242 |
| *EZH1* | Enhancer of zeste homolog 1 (Drosophila) | 1.263 | 0.00038 |
| *FABP3* | Fatty acid binding protein 3, muscle and heart (mammary-derived growth inhibitor) | -1.457 | 0.0002 |
| *FADS3* | Fatty acid desaturase 3 | -1.414 | 0.00456 |
| *FAH* | Fumarylacetoacetate hydrolase (fumarylacetoacetase) | 2.648 | 7.94E-13 |
| *FAM101B* | Family with sequence similarity 101, member B | -1.777 | 0.00017 |
| *FAM109A* | Family with sequence similarity 109, member A | -1.993 | 4.50E-06 |
| *FAM134B* | Family with sequence similarity 134, member B | 1.562 | 0.00159 |
| *FAM171A2* | Family with sequence similarity 171, member A2 | 1.592 | 3.61E-05 |
| *FAM173A* | Family with sequence similarity 173, member A | -1.667 | 0.00017 |
| *FAM174B* | Family with sequence similarity 174, member B | -2.023 | 4.11E-07 |
| *FAM177A1* | Family with sequence similarity 177, member A1 | -1.375 | 0.00519 |
| *FAM188A* | Family with sequence similarity 188, member A | -1.45 | 0.00053 |
| *FAM189B* | Family with sequence similarity 189, member B | -1.261 | 0.00522 |
| *FAM195B* | Family with sequence similarity 195, member B | 1.306 | 0.01002 |
| *FAM214A* | Family with sequence similarity 214, member A | -1.464 | 0.00212 |
| *FAM46C* | Family with sequence similarity 46, member C | -1.932 | 4.40E-06 |
| *FAM49A* | Family with sequence similarity 49, member A | -1.699 | 0.00024 |
| *FAM53C* | Family with sequence similarity 53, member C | 1.264 | 0.00037 |
| *FAM63A* | Family with sequence similarity 63, member A | 1.36 | 3.91E-05 |
| *FAM69B* | Family with sequence similarity 69, member B | 1.545 | 0.00492 |
| *FAM83H* | Family with sequence similarity 83, member H | 1.578 | 0.00018 |
| *FANCC* | Fanconi anemia, complementation group C | -1.377 | 0.00607 |
| *FANCM* | Fanconi anemia, complementation group M | -1.669 | 0.00181 |
| *FBP2* | Fructose-1,6-bisphosphatase 2 | 1.269 | 0.01035 |
| *FBXL5* | F-box and leucine-rich repeat protein 5 | 1.344 | 0.00268 |
| *FBXO10* | F-box protein 10 | 1.797 | 3.19E-06 |
| *FCHSD1* | FCH and double SH3 domains 1 | 1.406 | 0.00027 |
| *FDFT1* | Farnesyl-diphosphate farnesyltransferase 1 | 1.252 | 0.00231 |
| *FECH* | Ferrochelatase | 1.291 | 0.00053 |
| *FERMT2* | Fermitin family member 2 | -1.356 | 0.0003 |
| *FGFR4* | Fibroblast growth factor receptor 4 | -1.644 | 4.42E-05 |
| *FGL2* | Fibrinogen-like 2 | -1.486 | 0.00019 |
| *FIBIN* | Fin bud initiation factor homolog (zebrafish) | 1.747 | 0.00789 |
| *FIS1* | Fission 1 (mitochondrial outer membrane) homolog (S. cerevisiae) | 1.344 | 7.75E-05 |
| *FKBP10* | FK506 binding protein 10, 65 kDa | -1.448 | 0.0117 |
| *FKBP14* | FK506 binding protein 14, 22 kDa | -1.796 | 8.23E-06 |
| *FMNL1* | Formin-like 1 | -1.574 | 0.00288 |
| *FMO5* | Flavin containing monooxygenase 5 | -1.589 | 0.00898 |
| *FMOD* | Fibromodulin | -1.73 | 0.00962 |
| *FNTB* | Farnesyltransferase, CAAX box, beta | 1.447 | 0.0136 |
| *FOS* | FBJ murine osteosarcoma viral oncogene homolog | -6.206 | 6.83E-06 |
| *FOXP1* | Forkhead box P1 | 1.296 | 0.00488 |
| *FOXS1* | Forkhead box S1 | 1.668 | 0.00014 |
| *FRMD4A* | FERM domain containing 4A | -1.376 | 1.99E-05 |
| *FRRS1* | Ferric-chelate reductase 1 | 1.559 | 0.00153 |
| *FTSJ3* | FtsJ homolog 3 (E. coli) | -1.305 | 0.00277 |
| *FXYD1* | FXYD domain containing ion transport regulator 1 | 1.313 | 0.00215 |
| *G3BP2* | GTPase activating protein (SH3 domain) binding protein 2 | -1.357 | 0.00209 |
| *G6PD* | Glucose-6-phosphate dehydrogenase | -2.284 | 0.0032 |
| *GAB2* | GRB2-associated binding protein 2 | 1.33 | 0.00629 |
| *GALK1* | Galactokinase 1 | 1.742 | 2.36E-05 |
| *GALM* | Galactose mutarotase (aldose 1-epimerase) | -1.636 | 3.81E-05 |
| *GALNT16* | UDP-N-acetyl-alpha-D-galactosamine:polypeptide N-acetylgalactosaminyltransferase 16 | -1.56 | 5.84E-05 |
| *GALT* | Galactose-1-phosphate uridylyltransferase | 1.422 | 0.00944 |
| *GAMT* | Guanidinoacetate N-methyltransferase | -1.264 | 0.00521 |
| *GANAB* | Glucosidase, alpha; neutral AB | -1.253 | 0.00651 |
| *GAPDH* | Glyceraldehyde-3-phosphate dehydrogenase | 1.364 | 8.79E-05 |
| *GAR1* | GAR1 ribonucleoprotein | -1.37 | 0.0025 |
| *GAS1* | Growth arrest-specific 1 | 1.68 | 0.00067 |
| *GAS6* | Growth arrest-specific 6 | 1.295 | 0.00808 |
| *GATSL2* | GATS protein-like 2 | 1.318 | 0.00208 |
| *GBAS* | Glioblastoma amplified sequence | 1.315 | 0.00017 |
| *GBP1* | Guanylate binding protein 1, interferon-inducible | -1.934 | 0.00676 |
| *GCGR* | Glucagon receptor | 2.446 | 1.58E-06 |
| *GCNT1* | Glucosaminyl (N-acetyl) transferase 1, core 2 | 1.628 | 0.00139 |
| *GID4* | GID complex subunit 4 | -1.287 | 0.00039 |
| *GJA1* | Gap junction protein, alpha 1, 43kDa | -1.473 | 0.00819 |
| *GLRX* | Glutaredoxin (thioltransferase) | -1.359 | 0.00062 |
| *GLYCTK* | Glycerate kinase | -1.33 | 0.01247 |
| *GMCL1* | Germ cell-less, spermatogenesis associated 1 | -1.337 | 0.01491 |
| *GMNN* | Geminin, DNA replication inhibitor | -1.555 | 0.00095 |
| *GNB2* | Guanine nucleotide binding protein (G protein), beta polypeptide 2 | 1.273 | 0.00399 |
| *GNL3* | Guanine nucleotide binding protein-like 3 (nucleolar) | -1.312 | 0.00058 |
| *GOLGA4* | Golgin A4 | -1.572 | 0.01467 |
| *GOLGB1* | Golgin B1 | -1.331 | 0.01334 |
| *GOLIM4* | Golgi integral membrane protein 4 | -1.296 | 0.01434 |
| *GOT1* | Glutamic-oxaloacetic transaminase 1, soluble | 1.292 | 0.00274 |
| *GPAA1* | Glycosylphosphatidylinositol anchor attachment 1 | 1.251 | 0.00824 |
| *GPAM* | Glycerol-3-phosphate acyltransferase, mitochondrial | -2.534 | 3.34E-06 |
| *GPC1* | Glypican 1 | -1.402 | 2.65E-05 |
| *GPC3* | Glypican 3 | 1.438 | 0.0096 |
| *GPCPD1* | Glycerophosphocholine phosphodiesterase GDE1 homolog (S. cerevisiae) | -2.179 | 5.93E-11 |
| *GPR146* | G protein-coupled receptor 146 | -1.391 | 0.00161 |
| *GPRC5B* | G protein-coupled receptor, family C, group 5, member B | 1.4 | 0.00156 |
| *GPT2* | Glutamic pyruvate transaminase (alanine aminotransferase) 2 | 1.397 | 0.00138 |
| *GRAMD1B* | GRAM domain containing 1B | 2.111 | 7.75E-07 |
| *GRB10* | Growth factor receptor-bound protein 10 | 1.322 | 0.00019 |
| *GREB1* | Growth regulation by estrogen in breast cancer 1 | -2.035 | 0.00263 |
| *GRK4* | G protein-coupled receptor kinase 4 | -1.73 | 5.72E-08 |
| *GSTK1* | Glutathione S-transferase kappa 1 | -1.432 | 0.00011 |
| *GSTM1* | Glutathione S-transferase mu 1 | -1.483 | 0.01017 |
| *GTDC1* | Glycosyltransferase-like domain containing 1 | -1.366 | 0.00528 |
| *GTF2H1* | General transcription factor IIH, polypeptide 1, 62kDa | -1.28 | 0.01358 |
| *GTF3C5* | General transcription factor IIIC, polypeptide 5, 63kDa | 1.265 | 0.00653 |
| *H1FX* | H1 histone family, member X | 1.421 | 6.69E-06 |
| *H2AFJ* | H2A histone family, member J | 1.378 | 0.00128 |
| *HABP4* | Hyaluronan binding protein 4 | -1.375 | 0.00044 |
| *HAGHL* | Hydroxyacylglutathione hydrolase-like | -1.42 | 0.00022 |
| *HDAC11* | Histone deacetylase 11 | 1.356 | 0.01394 |
| *Hdac9* | Histone deacetylase 9 | 1.295 | 0.01053 |
| *Hddc3* | HD domain containing 3 | 1.421 | 0.00322 |
| *HECTD4* | HECT domain containing E3 ubiquitin protein ligase 4 | -1.376 | 0.00137 |
| *HERC2* | HECT and RLD domain containing E3 ubiquitin protein ligase 2 | -1.55 | 5.57E-09 |
| *HERC4* | HECT and RLD domain containing E3 ubiquitin protein ligase 4 | -1.54 | 0.00047 |
| *HES1* | Hes family bHLH transcription factor 1 | -1.622 | 0.00104 |
| *HEXDC* | Hexosaminidase (glycosyl hydrolase family 20, catalytic domain) containing | -1.455 | 0.00313 |
| *HFE2* | Hemochromatosis type 2 (juvenile) | 1.271 | 0.00081 |
| *HIST1H2AC* | Histone cluster 1, H2ac | -2.466 | 5.34E-08 |
| *HIST1H2BD* | Histone cluster 1, H2bd | -1.397 | 4.96E-05 |
| *HLF* | Hepatic leukemia factor | -1.297 | 0.01015 |
| *HMGA1* | High mobility group AT-hook 1 | 1.251 | 0.00987 |
| *HMGCR* | 3-hydroxy-3-methylglutaryl-CoA reductase | -1.461 | 0.00139 |
| *HOMER1* | Homer homolog 1 (Drosophila) | -1.339 | 0.00453 |
| *HOXC5* | Homeobox C5 | -1.494 | 0.0052 |
| *HPCAL4* | Hippocalcin like 4 | 1.531 | 0.00918 |
| *HR* | Hair growth associated | -2.747 | 8.61E-08 |
| *HRSP12* | Heat-responsive protein 12 | -1.41 | 0.00528 |
| *HSD17B12* | Hydroxysteroid (17-beta) dehydrogenase 12 | -1.736 | 0.0001 |
| *HSD17B7* | Hydroxysteroid (17-beta) dehydrogenase 7 | -1.581 | 0.00017 |
| *HSD17B8* | Hydroxysteroid (17-beta) dehydrogenase 8 | -1.408 | 3.75E-05 |
| *HSPH1* | Heat shock 105kDa/110kDa protein 1 | -1.544 | 0.00048 |
| *IARS* | Isoleucyl-tRNA synthetase | -1.26 | 0.01088 |
| *ICA1* | Islet cell autoantigen 1, 69kDa | -1.885 | 3.48E-05 |
| *IDH1* | Isocitrate dehydrogenase 1 (NADP+), soluble | -1.48 | 0.00345 |
| *IDH2* | Isocitrate dehydrogenase 2 (NADP+), mitochondrial | 1.423 | 6.13E-05 |
| *IDH3B* | Isocitrate dehydrogenase 3 (NAD+) beta | 1.489 | 3.36E-09 |
| *IDH3G* | Isocitrate dehydrogenase 3 (NAD+) gamma | 1.269 | 0.0052 |
| *IDI1* | Isopentenyl-diphosphate delta isomerase 1 | -1.348 | 0.00021 |
| *IER2* | Immediate early response 2 | -1.748 | 1.43E-05 |
| *IFFO2* | Intermediate filament family orphan 2 | -1.929 | 0.00021 |
| *IFI35* | Interferon-induced protein 35 | 1.262 | 0.00344 |
| *IFT122* | Intraflagellar transport 122 homolog (Chlamydomonas) | -1.32 | 0.00789 |
| *IFT140* | Intraflagellar transport 140 homolog (Chlamydomonas) | -1.884 | 1.68E-06 |
| *IFT172* | Intraflagellar transport 172 homolog (Chlamydomonas) | -1.48 | 0.00923 |
| *IFT88* | Intraflagellar transport 88 homolog (Chlamydomonas) | -1.419 | 0.00369 |
| *IGF1R* | Insulin-like growth factor 1 receptor | 1.673 | 3.81E-09 |
| *IGFBP4* | Insulin-like growth factor binding protein 4 | -1.476 | 3.20E-05 |
| *IGFBP5* | Insulin-like growth factor binding protein 5 | -1.429 | 0.00119 |
| *IGFBP7* | Insulin-like growth factor binding protein 7 | -1.285 | 0.00104 |
| *IGJ* | Immunoglobulin J polypeptide, linker protein for immunoglobulin alpha and mu polypeptides | -1.588 | 0.00074 |
| *IGSF8* | Immunoglobulin superfamily, member 8 | -1.513 | 0.00113 |
| *IKBKG* | Inhibitor of kappa light polypeptide gene enhancer in B-cells, kinase gamma | -1.308 | 0.00954 |
| *IL11RA* | Interleukin 11 receptor, alpha | 1.331 | 0.00137 |
| *IL4R* | Interleukin 4 receptor | 1.308 | 0.00107 |
| *ILVBL* | IlvB (bacterial acetolactate synthase)-like | 1.358 | 0.00239 |
| *IMPA1* | Inositol(myo)-1(or 4)-monophosphatase 1 | -1.367 | 0.00135 |
| *IMPA2* | Inositol(myo)-1(or 4)-monophosphatase 2 | 1.54 | 0.01117 |
| *ING2* | Inhibitor of growth family, member 2 | -1.295 | 0.0093 |
| *INHA* | Inhibin, alpha | -2.65 | 2.38E-06 |
| *INO80B* | INO80 complex subunit B | 1.414 | 0.00057 |
| *INPP5K* | Inositol polyphosphate-5-phosphatase K | 1.293 | 0.00023 |
| *INSIG1* | Insulin induced gene 1 | -1.867 | 0.0042 |
| *INTS6* | Integrator complex subunit 6 | -1.292 | 0.01416 |
| *INVS* | Inversin | -1.388 | 0.00015 |
| *IRAK1* | Interleukin-1 receptor-associated kinase 1 | -1.588 | 9.42E-05 |
| *IRAK2* | Interleukin-1 receptor-associated kinase 2 | -1.381 | 0.00178 |
| *ISOC2* | Isochorismatase domain containing 2 | 1.599 | 4.79E-06 |
| *ITGA5* | Integrin, alpha 5 (fibronectin receptor, alpha polypeptide) | -1.356 | 0.0009 |
| *ITGB1BP2* | Integrin beta 1 binding protein (melusin) 2 | -1.291 | 0.0032 |
| *ITGB5* | Integrin, beta 5 | -1.301 | 0.00209 |
| *ITPK1* | Inositol-tetrakisphosphate 1-kinase | -1.446 | 0.00099 |
| *ITPRIP* | Inositol 1,4,5-trisphosphate receptor interacting protein | 1.374 | 0.00056 |
| *ITPRIPL1* | Inositol 1,4,5-trisphosphate receptor interacting protein-like 1 | 1.302 | 0.00697 |
| *ITSN2* | Intersectin 2 | -1.414 | 0.0012 |
| *JADE2* | Jade family PHD finger 2 | 1.725 | 4.09E-06 |
| *JARID2* | Jumonji, AT rich interactive domain 2 | -1.339 | 0.00212 |
| *JKAMP* | JNK1/MAPK8-associated membrane protein | -1.472 | 0.00341 |
| *JMJD1C* | Jumonji domain containing 1C | -1.546 | 9.22E-05 |
| *JUN* | Jun proto-oncogene | -1.397 | 0.0004 |
| *JUNB* | Jun B proto-oncogene | -1.874 | 0.001 |
| *KANK4* | KN motif and ankyrin repeat domains 4 | 1.421 | 0.00327 |
| *KAT8* | K(lysine) acetyltransferase 8 | 1.324 | 8.12E-05 |
| *KBTBD13* | Kelch repeat and BTB (POZ) domain containing 13 | 1.508 | 0.00053 |
| *KBTBD2* | Kelch repeat and BTB (POZ) domain containing 2 | -1.557 | 1.20E-06 |
| *KCNA5* | Potassium voltage-gated channel, shaker-related subfamily, member 5 | -1.717 | 9.70E-06 |
| *KCNB1* | Potassium voltage-gated channel, Shab-related subfamily, member 1 | -1.689 | 7.28E-05 |
| *KCNC4* | Potassium voltage-gated channel, Shaw-related subfamily, member 4 | 2.184 | 1.82E-08 |
| *KCNE1L* | KCNE1-like | 2.563 | 2.34E-05 |
| *KCNG2* | Potassium voltage-gated channel, subfamily G, member 2 | 1.997 | 9.41E-05 |
| *KCNJ8* | Potassium inwardly-rectifying channel, subfamily J, member 8 | -1.399 | 0.00368 |
| *KCNMB1* | Potassium large conductance calcium-activated channel, subfamily M, beta member 1 | -2.026 | 1.05E-05 |
| *KCNS3* | Potassium voltage-gated channel, delayed-rectifier, subfamily S, member 3 | 2.079 | 3.46E-10 |
| *KCP* | Kielin/chordin-like protein | -1.75 | 0.00114 |
| *KCTD15* | Potassium channel tetramerization domain containing 15 | -1.434 | 0.01242 |
| *KCTD2* | Potassium channel tetramerization domain containing 2 | 1.345 | 1.47E-05 |
| *KCTD7* | Potassium channel tetramerization domain containing 7 | -1.485 | 0.00335 |
| *KDELC1* | KDEL (Lys-Asp-Glu-Leu) containing 1 | -1.395 | 0.00908 |
| *KDELC2* | KDEL (Lys-Asp-Glu-Leu) containing 2 | -1.326 | 0.01033 |
| *KDM4C* | Lysine (K)-specific demethylase 4C | -1.263 | 0.00885 |
| *KDM5C* | Lysine (K)-specific demethylase 5C | -1.437 | 1.57E-05 |
| *KHNYN* | KH and NYN domain containing | 1.324 | 0.00036 |
| *KIAA0020* | KIAA0020 | -1.449 | 0.00299 |
| *KIAA0100* | KIAA0100 | -1.299 | 0.00017 |
| *KIAA0141* | KIAA0141 | 1.275 | 4.48E-05 |
| *KIAA0408* | KIAA0408 | 1.483 | 0.00488 |
| *KIAA0430* | KIAA0430 | 1.252 | 0.0015 |
| *KIAA0753* | KIAA0753 | 2.19 | 0.00032 |
| *KIAA1191* | KIAA1191 | -1.354 | 0.00107 |
| *KIF21A* | Kinesin family member 21A | -1.529 | 9.36E-05 |
| *KLF10* | Kruppel-like factor 10 | -1.648 | 0.00012 |
| *KLF15* | Kruppel-like factor 15 | 1.413 | 0.00657 |
| *KLF5* | Kruppel-like factor 5 (intestinal) | 1.467 | 0.00146 |
| *KLHL17* | Kelch-like family member 17 | -1.986 | 0.00133 |
| *KLHL2* | Kelch-like family member 2 | -1.705 | 9.93E-05 |
| *KLHL22* | Kelch-like family member 22 | 1.334 | 0.00284 |
| *KLHL41* | Kelch-like family member 41 | 1.297 | 0.00551 |
| *KPNA2* | Karyopherin alpha 2 (RAG cohort 1, importin alpha 1) | -1.437 | 0.00019 |
| *KREMEN1* | Kringle containing transmembrane protein 1 | 1.329 | 0.00104 |
| *LAMA3* | Laminin, alpha 3 | -2.087 | 0.00018 |
| *LANCL1* | LanC lantibiotic synthetase component C-like 1 (bacterial) | -1.373 | 0.00598 |
| *Larp1b* | La ribonucleoprotein domain family, member 1B | -1.626 | 4.39E-06 |
| *LASP1* | LIM and SH3 protein 1 | -1.397 | 6.36E-05 |
| *LBR* | Lamin B receptor | 1.258 | 0.0146 |
| *LDLRAD4* | Low density lipoprotein receptor class A domain containing 4 | -1.879 | 2.74E-06 |
| *LDLRAP1* | Low density lipoprotein receptor adaptor protein 1 | -1.395 | 0.00353 |
| *LEO1* | Leo1, Paf1/RNA polymerase II complex component, homolog (S. cerevisiae) | -1.276 | 0.00809 |
| *LGALS1* | Lectin, galactoside-binding, soluble, 1 | -1.266 | 0.00056 |
| *LHFPL2* | Lipoma HMGIC fusion partner-like 2 | -1.374 | 0.01368 |
| *LHPP* | Phospholysine phosphohistidine inorganic pyrophosphate phosphatase | -1.329 | 0.00242 |
| *LIMA1* | LIM domain and actin binding 1 | -1.51 | 0.00217 |
| *LIMK2* | LIM domain kinase 2 | 1.534 | 3.67E-05 |
| *LIN37* | Lin-37 homolog (C. elegans) | 1.353 | 0.0039 |
| *LIN9* | Lin-9 homolog (C. elegans) | -1.603 | 0.00314 |
| *LIX1* | Lix1 homolog (chicken) | -1.587 | 0.00011 |
| *LLGL2* | Lethal giant larvae homolog 2 (Drosophila) | 1.588 | 0.01023 |
| *LMNB1* | Lamin B1 | -1.494 | 0.00212 |
| *LMOD1* | Leiomodin 1 (smooth muscle) | 1.655 | 1.53E-05 |
| *LMOD3* | Leiomodin 3 (fetal) | -1.393 | 0.00124 |
| *LOC81691* | Exonuclease NEF-sp | -1.38 | 0.00959 |
| *LONP2* | Lon peptidase 2, peroxisomal | 1.373 | 2.90E-07 |
| *LOXL2* | Lysyl oxidase-like 2 | -1.655 | 0.00055 |
| *LPHN1* | Latrophilin 1 | -1.65 | 2.14E-05 |
| *LPIN2* | Lipin 2 | -1.493 | 0.01241 |
| *LPPR2* | Lipid phosphate phosphatase-related protein type 2 | -1.891 | 0.01126 |
| *LRP11* | Low density lipoprotein receptor-related protein 11 | -1.299 | 0.00256 |
| *LRRC17* | Leucine rich repeat containing 17 | -1.554 | 0.00842 |
| *LRRN1* | Leucine rich repeat neuronal 1 | -1.354 | 0.01227 |
| *LSM6* | LSM6 homolog, U6 small nuclear RNA associated (S. cerevisiae) | 1.365 | 0.0006 |
| *LSMD1* | LSM domain containing 1 | -1.35 | 0.00231 |
| *LTBP2* | Latent transforming growth factor beta binding protein 2 | 1.532 | 0.00559 |
| *LTV1* | LTV1 homolog (S. cerevisiae) | -1.54 | 3.99E-05 |
| *LUM* | Lumican | -1.593 | 0.00113 |
| *LYNX1* | Ly6/neurotoxin 1 | 1.712 | 0.00029 |
| *MACROD1* | MACRO domain containing 1 | 1.288 | 0.00966 |
| *MAF1* | MAF1 homolog (S. cerevisiae) | 1.253 | 0.00497 |
| *MAGED1* | Melanoma antigen family D, 1 | 1.323 | 0.00084 |
| *MAGIX* | MAGI family member, X-linked | -1.807 | 0.00144 |
| *MAL* | Mal, T-cell differentiation protein | 1.936 | 2.21E-05 |
| *MAMSTR* | MEF2 activating motif and SAP domain containing transcriptional regulator | -1.401 | 0.00416 |
| *MANBA* | Mannosidase, beta A, lysosomal | 1.707 | 0.0003 |
| *MAOA* | Monoamine oxidase A | 1.616 | 0.00017 |
| *MAOB* | Monoamine oxidase B | -1.574 | 0.00183 |
| *MAP2K3* | Mitogen-activated protein kinase kinase 3 | 1.298 | 0.0051 |
| *Map4k4* | Mitogen-activated protein kinase kinase kinase kinase 4 | -1.251 | 0.00413 |
| *MAPK12* | Mitogen-activated protein kinase 12 | 1.436 | 0.00027 |
| *MAPT* | Microtubule-associated protein tau | -1.285 | 0.00398 |
| *Mar-02* | Mitochondrial amidoxime reducing component 2 | -1.366 | 0.00185 |
| *MARCKSL1* | MARCKS-like 1 | -1.596 | 0.00192 |
| *MARS* | Methionyl-tRNA synthetase | -1.274 | 0.00056 |
| *MASP1* | Mannan-binding lectin serine peptidase 1 (C4/C2 activating component of Ra-reactive factor) | 1.368 | 0.00065 |
| *MAT2A* | Methionine adenosyltransferase II, alpha | -1.316 | 0.01096 |
| *MBD3* | Methyl-CpG binding domain protein 3 | -1.336 | 0.0035 |
| *MCHR1* | Melanin-concentrating hormone receptor 1 | -3.54 | 4.45E-09 |
| *MCM2* | Minichromosome maintenance complex component 2 | 1.404 | 0.00134 |
| *MCOLN1* | Mucolipin 1 | 1.298 | 0.00211 |
| *MDM2* | MDM2 oncogene, E3 ubiquitin protein ligase | -1.439 | 0.00033 |
| *MDN1* | MDN1, midasin homolog (yeast) | -1.287 | 0.0028 |
| *ME1* | Malic enzyme 1, NADP(+)-dependent, cytosolic | -1.489 | 0.00017 |
| *ME3* | Malic enzyme 3, NADP(+)-dependent, mitochondrial | 1.742 | 8.30E-05 |
| *MED10* | Mediator complex subunit 10 | 1.26 | 0.01421 |
| *MED12* | Mediator complex subunit 12 | -1.533 | 5.01E-05 |
| *MED20* | Mediator complex subunit 20 | -1.372 | 0.01115 |
| *MEGF10* | Multiple EGF-like-domains 10 | -1.435 | 0.00511 |
| *MEST* | Mesoderm specific transcript | -1.958 | 2.37E-05 |
| *METTL18* | Methyltransferase like 18 | -1.35 | 0.00419 |
| *METTL21C* | Methyltransferase like 21C | 1.763 | 4.82E-05 |
| *METTL23* | Methyltransferase like 23 | -1.315 | 0.00657 |
| *METTL8* | Methyltransferase like 8 | -1.47 | 0.0071 |
| *MEX3D* | Mex-3 RNA binding family member D | 1.461 | 0.00069 |
| *MFN1* | Mitofusin 1 | -1.298 | 0.00971 |
| *MFSD4* | Major facilitator superfamily domain containing 4 | -1.956 | 0.00401 |
| *MFSD8* | Major facilitator superfamily domain containing 8 | -1.486 | 0.00389 |
| *MGARP* | Mitochondria-localized glutamic acid-rich protein | 1.431 | 0.00504 |
| *MGP* | Matrix Gla protein | -1.45 | 0.0014 |
| *MGST1* | Microsomal glutathione S-transferase 1 | -2.677 | 0.00061 |
| *MICAL2* | Microtubule associated monooxygenase, calponin and LIM domain containing 2 | -2.098 | 1.51E-06 |
| *MICAL3* | Microtubule associated monooxygenase, calponin and LIM domain containing 3 | -1.256 | 0.01376 |
| *MIEN1* | Migration and invasion enhancer 1 | -1.694 | 1.30E-08 |
| *MINA* | MYC induced nuclear antigen | 1.514 | 2.37E-07 |
| *MKLN1* | Muskelin 1, intracellular mediator containing kelch motifs | -1.527 | 0.00213 |
| *MLEC* | Malectin | -1.412 | 0.00054 |
| *MLF1* | Myeloid leukemia factor 1 | -1.411 | 0.00048 |
| *MLLT11* | Myeloid/lymphoid or mixed-lineage leukemia (trithorax homolog, Drosophila); 11 | -2.246 | 0.0007 |
| *MLLT3* | Myeloid/lymphoid or mixed-lineage leukemia (trithorax homolog, Drosophila); 3 | -1.399 | 0.00193 |
| *MLLT6* | Myeloid/lymphoid or mixed-lineage leukemia (trithorax homolog, Drosophila); 6 | 1.334 | 0.00143 |
| *MLST8* | MTOR associated protein, LST8 homolog (S. cerevisiae) | 1.328 | 0.00079 |
| *MNS1* | Meiosis-specific nuclear structural 1 | 1.824 | 0.00451 |
| *MOB3A* | MOB kinase activator 3A | 1.307 | 0.00174 |
| *MOB3B* | MOB kinase activator 3B | 1.707 | 0.00207 |
| *MPI* | Mannose phosphate isomerase | 1.456 | 5.14E-05 |
| *MPND* | MPN domain containing | -1.557 | 4.14E-05 |
| *MPP5* | Membrane protein, palmitoylated 5 (MAGUK p55 subfamily member 5) | -1.318 | 0.00876 |
| *MPPE1* | Metallophosphoesterase 1 | -1.514 | 0.00745 |
| *MPZL1* | Myelin protein zero-like 1 | -1.367 | 0.01349 |
| *MRO* | Maestro | -1.392 | 0.01022 |
| *MRPL19* | Mitochondrial ribosomal protein L19 | -1.287 | 0.01466 |
| *MRPL2* | Mitochondrial ribosomal protein L2 | 1.355 | 4.85E-05 |
| *MRPL4* | Mitochondrial ribosomal protein L4 | 1.276 | 0.00515 |
| *MRPL41* | Mitochondrial ribosomal protein L41 | 1.397 | 0.00284 |
| *MRPS23* | Mitochondrial ribosomal protein S23 | 1.338 | 6.05E-06 |
| *MRPS6* | Mitochondrial ribosomal protein S6 | 1.277 | 0.0095 |
| *MRPS9* | Mitochondrial ribosomal protein S9 | 1.351 | 3.23E-05 |
| *MSH3* | MutS homolog 3 | -1.321 | 0.00536 |
| *MSMO1* | Methylsterol monooxygenase 1 | -1.423 | 0.0014 |
| *MST4* | Serine/threonine protein kinase MST4 | -1.48 | 0.00047 |
| *MSTO1* | Misato 1, mitochondrial distribution and morphology regulator | 1.43 | 0.00028 |
| *MTERFD3* | MTERF domain containing 3 | -1.463 | 0.00061 |
| *MTHFR* | Methylenetetrahydrofolate reductase (NAD(P)H) | 1.306 | 0.00087 |
| *MTIF3* | Mitochondrial translational initiation factor 3 | 1.291 | 6.13E-05 |
| *MTMR1* | Myotubularin related protein 1 | -1.481 | 7.26E-06 |
| *MTX1* | Metaxin 1 | 1.312 | 0.00047 |
| *MUL1* | Mitochondrial E3 ubiquitin protein ligase 1 | 1.452 | 3.51E-06 |
| *MUM1L1* | Melanoma associated antigen (mutated) 1-like 1 | 1.885 | 0.00059 |
| *MVK* | Mevalonate kinase | 1.26 | 0.00464 |
| *MXRA5* | Matrix-remodelling associated 5 | -1.766 | 0.00131 |
| *MYADM* | Myeloid-associated differentiation marker | -1.253 | 0.0072 |
| *MYC* | V-myc avian myelocytomatosis viral oncogene homolog | -1.594 | 0.00043 |
| *MYF5* | Myogenic factor 5 | -1.575 | 0.00076 |
| *MYH14* | Myosin, heavy chain 14, non-muscle | 1.497 | 6.88E-05 |
| *MYL6* | Myosin, light chain 6, alkali, smooth muscle and non-muscle | -1.428 | 0.00021 |
| *MYL6B* | Myosin, light chain 6B, alkali, smooth muscle and non-muscle | -1.995 | 0.00935 |
| *MYL9* | Myosin, light chain 9, regulatory | -1.393 | 0.00359 |
| *MYLIP* | Myosin regulatory light chain interacting protein | 1.32 | 0.00139 |
| *MYLK3* | Myosin light chain kinase 3 | 2.026 | 1.52E-05 |
| *MYLK4* | Myosin light chain kinase family, member 4 | -1.979 | 0.00014 |
| *MYO10* | Myosin X | -1.526 | 3.67E-05 |
| *MYO1B* | Myosin IB | -1.324 | 0.00749 |
| *MYO1E* | Myosin IE | -1.614 | 5.38E-08 |
| *MYOZ1* | Myozenin 1 | 1.323 | 9.91E-05 |
| *N6AMT1* | N-6 adenine-specific DNA methyltransferase 1 (putative) | -1.493 | 0.00422 |
| *NAALADL1* | N-acetylated alpha-linked acidic dipeptidase-like 1 | -1.494 | 0.00118 |
| *NAGK* | N-acetylglucosamine kinase | -1.388 | 0.0041 |
| *NAGLU* | N-acetylglucosaminidase, alpha | -1.378 | 0.00356 |
| *NAT14* | N-acetyltransferase 14 (GCN5-related, putative) | 1.378 | 0.00028 |
| *NCALD* | Neurocalcin delta | -1.299 | 0.01116 |
| *NCAM1* | Neural cell adhesion molecule 1 | -1.644 | 0.01461 |
| *NCAPD2* | Non-SMC condensin I complex, subunit D2 | -1.498 | 0.00127 |
| *NDRG2* | NDRG family member 2 | -1.921 | 1.20E-15 |
| *NDUFA9* | NADH dehydrogenase (ubiquinone) 1 alpha subcomplex, 9, 39kDa | 1.361 | 1.62E-06 |
| *NDUFAF3* | NADH dehydrogenase (ubiquinone) complex I, assembly factor 3 | 1.297 | 0.00134 |
| *NDUFA10* | NADH dehydrogenase (ubiquinone) 1 alpha subcomplex, 10, 42kDa | 1.253 | 0.00798 |
| *NDUFB6* | NADH dehydrogenase (ubiquinone) 1 beta subcomplex, 6, 17kDa | 1.273 | 0.00343 |
| *NDUFB7* | NADH dehydrogenase (ubiquinone) 1 beta subcomplex, 7, 18kDa | 1.262 | 0.00553 |
| *NDUFB8* | NADH dehydrogenase [ubiquinone] 1 beta subcomplex subunit 8, mitochondrial | 1.252 | 0.00368 |
| *NDUFB10* | NADH dehydrogenase (ubiquinone) 1 beta subcomplex, 10, 22kDa | 1.504 | 6.47E-07 |
| *NDUFS3* | NADH dehydrogenase | 1.261 | 0.00809 |
| *NDUFS5* | NADH dehydrogenase (ubiquinone) Fe-S protein 5, 15kDa (NADH-coenzyme Q reductase) | 1.273 | 0.01045 |
| *NDUFS7* | NADH dehydrogenase (ubiquinone) Fe-S protein 7, 20kDa (NADH-coenzyme Q reductase) | 1.508 | 1.81E-05 |
| *NDUFS8* | NADH dehydrogenase (ubiquinone) Fe-S protein 8, 23kDa (NADH-coenzyme Q reductase) | 1.334 | 0.00516 |
| *NDUFV1* | NADH dehydrogenase (ubiquinone) flavoprotein 1, 51kDa | 1.452 | 0.00028 |
| *NEMF* | Nuclear export mediator factor | -1.689 | 0.0034 |
| *NFIC* | Nuclear factor I/C (CCAAT-binding transcription factor) | 1.336 | 0.00063 |
| *NFIL3* | Nuclear factor, interleukin 3 regulated | -2.094 | 2.42E-05 |
| *NFKB2* | Nuclear factor of kappa light polypeptide gene enhancer in B-cells 2 (p49/p100) | -1.367 | 0.00057 |
| *NFKBIA* | Nuclear factor of kappa light polypeptide gene enhancer in B-cells inhibitor, alpha | 1.671 | 0.00066 |
| *NIM1K* | NIM1 serine/threonine protein kinase | -1.45 | 0.00314 |
| *NIPSNAP1* | Nipsnap homolog 1 (C. elegans) | -1.444 | 0.00175 |
| *NISCH* | Nischarin | -1.262 | 0.00101 |
| *NME3* | NME/NM23 nucleoside diphosphate kinase 3 | 1.327 | 0.01264 |
| *NOC3L* | Nucleolar complex associated 3 homolog (S. cerevisiae) | -1.37 | 0.00106 |
| *NOP16* | NOP16 nucleolar protein | -1.434 | 8.90E-06 |
| *NOV* | Nephroblastoma overexpressed | -1.719 | 0.00208 |
| *NPEPPS* | Aminopeptidase puromycin sensitive | -1.303 | 0.00178 |
| *NPM3* | Nucleophosmin/nucleoplasmin 3 | -1.447 | 0.00649 |
| *NPNT* | Nephronectin | -4.839 | 1.88E-40 |
| *NQO1* | NAD(P)H dehydrogenase, quinone 1 | -1.309 | 0.00104 |
| *NQO2* | NAD(P)H dehydrogenase, quinone 2 | 1.446 | 0.00132 |
| *NR1H3* | Nuclear receptor subfamily 1, group H, member 3 | 1.255 | 0.00492 |
| *NRAP* | Nebulin-related anchoring protein | 2.643 | 3.04E-25 |
| *NREP* | Neuronal regeneration related protein | -1.326 | 0.01105 |
| *NSRP1* | Nuclear speckle splicing regulatory protein 1 | -1.723 | 0.01086 |
| *NSUN4* | NOP2/Sun domain family, member 4 | 1.322 | 0.00014 |
| *NUDT2* | Nudix (nucleoside diphosphate linked moiety X)-type motif 2 | 1.319 | 0.00041 |
| *NUP155* | Nucleoporin 155kDa | -1.268 | 0.01292 |
| *OAT* | Ornithine aminotransferase | -1.736 | 1.78E-07 |
| *OAZ2* | Ornithine decarboxylase antizyme 2 | 1.358 | 3.61E-05 |
| *OCRL* | Oculocerebrorenal syndrome of Lowe | -1.293 | 0.0008 |
| *ODC1* | Ornithine decarboxylase 1 | -1.526 | 0.00086 |
| *ODF2L* | Outer dense fiber of sperm tails 2-like | -2.871 | 0.00017 |
| *OGDH* | Oxoglutarate (alpha-ketoglutarate) dehydrogenase (lipoamide) | 1.339 | 0.00012 |
| *ORC5* | Origin recognition complex, subunit 5 | -1.548 | 0.01 |
| *OSBP2* | Oxysterol binding protein 2 | -2.018 | 8.94E-05 |
| *OSBPL10* | Oxysterol binding protein-like 10 | 1.686 | 0.0002 |
| *OSBPL6* | Oxysterol binding protein-like 6 | -1.266 | 0.00563 |
| *OTUD1* | OTU domain containing 1 | -3.355 | 3.77E-05 |
| *OTUD3* | OTU domain containing 3 | -1.257 | 0.0024 |
| *OTUD6B* | OTU domain containing 6B | -1.326 | 0.01031 |
| *OXA1L* | Oxidase (cytochrome c) assembly 1-like | 1.265 | 0.00037 |
| *OXSM* | 3-oxoacyl-ACP synthase, mitochondrial | 1.567 | 6.43E-08 |
| *P2RY6* | Pyrimidinergic receptor P2Y, G-protein coupled, 6 | 1.594 | 0.01351 |
| *P4HA1* | Prolyl 4-hydroxylase, alpha polypeptide I | -1.583 | 4.16E-06 |
| *P4HA2* | Prolyl 4-hydroxylase, alpha polypeptide II | -1.439 | 0.00028 |
| *P4HB* | Prolyl 4-hydroxylase, beta polypeptide | 1.292 | 0.00035 |
| *PAFAH2* | Platelet-activating factor acetylhydrolase 2, 40kDa | -1.315 | 0.01156 |
| *PAIP2B* | Poly(A) binding protein interacting protein 2B | 1.312 | 0.00018 |
| *PALMD* | Palmdelphin | 1.379 | 0.00039 |
| *PAQR3* | Progestin and adipoQ receptor family member III | 1.351 | 0.00103 |
| *PARL* | Presenilin associated, rhomboid-like | 1.362 | 7.07E-06 |
| *PARM1* | Prostate androgen-regulated mucin-like protein 1 | -1.573 | 0.0011 |
| *PARS2* | Prolyl-tRNA synthetase 2, mitochondrial (putative) | 1.447 | 0.00244 |
| *PARVA* | Parvin, alpha | -1.302 | 0.0029 |
| *PATZ1* | POZ (BTB) and AT hook containing zinc finger 1 | 1.515 | 9.09E-06 |
| *PAXBP1* | PAX3 and PAX7 binding protein 1 | -1.438 | 0.00756 |
| *PCBD1* | Pterin-4 alpha-carbinolamine dehydratase/dimerization cofactor of hepatocyte nuclear factor 1 alpha | 3.309 | 0.00113 |
| *PCDH12* | Protocadherin 12 | -1.825 | 1.61E-05 |
| *PCID2* | PCI domain containing 2 | 1.269 | 0.00497 |
| *PCIF1* | PDX1 C-terminal inhibiting factor 1 | 1.386 | 0.00042 |
| *PCK2* | Phosphoenolpyruvate carboxykinase 2 (mitochondrial) | -5.033 | 0.0004 |
| *PCSK4* | Proprotein convertase subtilisin/kexin type 4 | 2.208 | 8.72E-07 |
| *PDE7A* | Phosphodiesterase 7A | 1.394 | 0.00646 |
| *PDE7B* | Phosphodiesterase 7B | 1.419 | 0.00061 |
| *PDHB* | Pyruvate dehydrogenase (lipoamide) beta | 1.312 | 7.68E-05 |
| *PDK2* | Pyruvate dehydrogenase kinase, isozyme 2 | 1.33 | 0.00062 |
| *PDLIM7* | PDZ and LIM domain 7 (enigma) | -1.468 | 8.10E-05 |
| *PDPR* | Pyruvate dehydrogenase phosphatase regulatory subunit | 1.486 | 8.02E-09 |
| *PDXDC1* | Pyridoxal-dependent decarboxylase domain containing 1 | 1.512 | 1.51E-05 |
| *PEMT* | Phosphatidylethanolamine N-methyltransferase | -1.606 | 0.00264 |
| *PEX2* | Peroxisomal biogenesis factor 2 | -1.256 | 0.01408 |
| *PEX7* | Peroxisomal biogenesis factor 7 | -1.324 | 0.00586 |
| *PFKFB1* | 6-phosphofructo-2-kinase/fructose-2,6-biphosphatase 1 | -1.356 | 0.00104 |
| *PFKFB4* | 6-phosphofructo-2-kinase/fructose-2,6-biphosphatase 4 | 1.65 | 8.83E-07 |
| *PGAP3* | Post-GPI attachment to proteins 3 | 1.423 | 0.00032 |
| *PGD* | Phosphogluconate dehydrogenase | -1.46 | 0.01036 |
| *PGK1* | Phosphoglycerate kinase 1 | -1.342 | 0.00202 |
| *PGM1* | Phosphoglucomutase 1 | 1.306 | 0.00024 |
| *PGPEP1L* | Pyroglutamyl-peptidase I-like | 1.924 | 4.48E-07 |
| *PHB2* | Prohibitin 2 | 1.384 | 0.0023 |
| *PHF14* | PHD finger protein 14 | -1.29 | 0.00774 |
| *PHF21A* | PHD finger protein 21A | -1.456 | 0.00019 |
| *PHOSPHO2* | Phosphatase, orphan 2 | -1.593 | 1.68E-06 |
| *PHTF1* | Putative homeodomain transcription factor 1 | -1.534 | 0.00955 |
| *PHTF2* | Putative homeodomain transcription factor 2 | -1.39 | 0.01416 |
| *PICALM* | Phosphatidylinositol binding clathrin assembly protein | -1.256 | 0.00128 |
| *PID1* | Phosphotyrosine interaction domain containing 1 | 1.382 | 0.00256 |
| *PIK3AP1* | Phosphoinositide-3-kinase adaptor protein 1 | 1.644 | 0.00088 |
| *PIK3IP1* | Phosphoinositide-3-kinase interacting protein 1 | 1.352 | 0.00742 |
| *PITHD1* | PITH (C-terminal proteasome-interacting domain of thioredoxin-like) domain containing 1 | 1.279 | 0.00091 |
| *PITPNA* | Phosphatidylinositol transfer protein, alpha | 1.646 | 4.92E-08 |
| *PKD2* | Polycystic kidney disease 2 (autosomal dominant) | -1.438 | 0.00153 |
| *PKIG* | Protein kinase (cAMP-dependent, catalytic) inhibitor gamma | 1.404 | 0.00836 |
| *PKNOX2* | PBX/knotted 1 homeobox 2 | 1.442 | 3.64E-05 |
| *PLA2R1* | Phospholipase A2 receptor 1, 180kDa | -2.577 | 4.77E-07 |
| *PLCL2* | Phospholipase C-like 2 | 1.349 | 1.97E-05 |
| *PLD1* | Phospholipase D1, phosphatidylcholine-specific | 1.535 | 0.0089 |
| *PLD2* | Phospholipase D2 | -1.948 | 0.00218 |
| *PLEKHF1* | Pleckstrin homology domain containing, family F (with FYVE domain) member 1 | 1.317 | 0.00832 |
| *PLIN2* | Perilipin 2 | 1.805 | 1.38E-06 |
| *PLIN3* | Perilipin 3 | 1.344 | 0.0005 |
| *PLIN5* | Perilipin 5 | 2.048 | 1.51E-08 |
| *PLK1S1* | Polo-like kinase 1 substrate 1 | 1.377 | 0.0036 |
| *PLOD2* | Procollagen-lysine, 2-oxoglutarate 5-dioxygenase 2 | -1.898 | 0.00013 |
| *PMEPA1* | Prostate transmembrane protein, androgen induced 1 | -1.63 | 0.00609 |
| *PMF1* | Polyamine-modulated factor 1 | 1.861 | 1.57E-08 |
| *PMS1* | PMS1 postmeiotic segregation increased 1 (S. cerevisiae) | -1.541 | 0.00694 |
| *PNISR* | PNN-interacting serine/arginine-rich protein | -1.87 | 0.00405 |
| *PNPLA2* | Patatin-like phospholipase domain containing 2 | 1.767 | 5.16E-08 |
| *PNPO* | Pyridoxamine 5'-phosphate oxidase | -1.317 | 0.00145 |
| *POC1A* | POC1 centriolar protein A | 1.65 | 0.00045 |
| *POLB* | Polymerase (DNA directed), beta | 1.311 | 0.0112 |
| *POLD4* | Polymerase (DNA-directed), delta 4, accessory subunit | 1.297 | 0.00431 |
| *POLDIP2* | Polymerase (DNA-directed), delta interacting protein 2 | 1.318 | 0.0014 |
| *POLR3B* | Polymerase (RNA) III (DNA directed) polypeptide B | -1.362 | 0.00053 |
| *POLR3G* | Polymerase (RNA) III (DNA directed) polypeptide G (32kD) | -1.543 | 0.00173 |
| *POMC* | Proopiomelanocortin | 1.399 | 0.00737 |
| *PON2* | Paraoxonase 2 | -1.337 | 0.00636 |
| *PON3* | Paraoxonase 3 | 1.399 | 0.01043 |
| *PPA1* | Pyrophosphatase (inorganic) 1 | -1.548 | 6.45E-07 |
| *PPARD* | Peroxisome proliferator-activated receptor delta | 1.375 | 0.01 |
| *PPIC* | Peptidylprolyl isomerase C (cyclophilin C) | -1.492 | 0.00206 |
| *PPIE* | Peptidylprolyl isomerase E (cyclophilin E) | 1.264 | 0.00411 |
| *PPIP5K1* | Diphosphoinositol pentakisphosphate kinase 1 | -1.481 | 4.32E-07 |
| *PPM1J* | Protein phosphatase, Mg2+/Mn2+ dependent, 1J | -1.635 | 6.31E-05 |
| *PPM1K* | Protein phosphatase, Mg2+/Mn2+ dependent, 1K | 1.613 | 0.00035 |
| *PPP1R15A* | Protein phosphatase 1, regulatory subunit 15A | -1.436 | 0.00518 |
| *PPP1R3A* | Protein phosphatase 1, regulatory subunit 3A | -1.466 | 0.00516 |
| *PPP2R5B* | Protein phosphatase 2, regulatory subunit B', beta | -1.51 | 6.42E-05 |
| *PPWD1* | Peptidylprolyl isomerase domain and WD repeat containing 1 | -1.274 | 0.00157 |
| *PRADC1* | Protease-associated domain containing 1 | 1.282 | 0.00464 |
| *PRAF2* | PRA1 domain family, member 2 | -1.322 | 0.00062 |
| *PRC1* | Protein regulator of cytokinesis 1 | -1.649 | 6.85E-05 |
| *PRCP* | Prolylcarboxypeptidase (angiotensinase C) | -1.431 | 0.00101 |
| *PRICKLE1* | Prickle homolog 1 (Drosophila) | 1.306 | 0.00805 |
| *PRIMPOL* | Primase and polymerase (DNA-directed) | -1.635 | 0.00024 |
| *PRKAG3* | Protein kinase, AMP-activated, gamma 3 non-catalytic subunit | -1.32 | 0.00021 |
| *PRKCZ* | Protein kinase C, zeta | 1.33 | 0.00289 |
| *PRKD1* | Protein kinase D1 | -1.493 | 0.0003 |
| *PRKRA* | Protein kinase, interferon-inducible double stranded RNA dependent activator | -1.485 | 0.00936 |
| *PRMT3* | Protein arginine methyltransferase 3 | -1.467 | 0.00074 |
| *PRNP* | Prion protein | -1.308 | 0.00819 |
| *PRPF19* | Pre-mRNA processing factor 19 | -1.281 | 0.00024 |
| *PRPSAP2* | Phosphoribosyl pyrophosphate synthetase-associated protein 2 | 1.34 | 0.00098 |
| *PRR5* | Proline rich 5 (renal) | 1.251 | 0.01476 |
| *PRR5L* | Proline rich 5 like | -2.622 | 0.00418 |
| *PSMB3* | Proteasome (prosome, macropain) subunit, beta type, 3 | 1.265 | 0.0002 |
| *PSMB4* | Proteasome (prosome, macropain) subunit, beta type, 4 | 1.313 | 8.15E-06 |
| *PSMC1* | Proteasome (prosome, macropain) 26S subunit, ATPase, 1 | 1.385 | 1.03E-05 |
| *PSMC3* | Proteasome (prosome, macropain) 26S subunit, ATPase, 3 | 1.367 | 0.00042 |
| *PSMF1* | Proteasome (prosome, macropain) inhibitor subunit 1 (PI31) | 1.293 | 0.00074 |
| *PSMG4* | Proteasome (prosome, macropain) assembly chaperone 4 | 1.327 | 0.00097 |
| *PSPH* | Phosphoserine phosphatase | -2.462 | 2.79E-08 |
| *PTEN* | Phosphatase and tensin homolog | -1.469 | 0.00171 |
| *PTGER4* | Prostaglandin E receptor 4 (subtype EP4) | -1.542 | 0.00012 |
| *PTGS1* | Prostaglandin-endoperoxide synthase 1 (prostaglandin G/H synthase and cyclooxygenase) | 1.431 | 0.00613 |
| *PTMS* | Parathymosin | -1.508 | 0.0026 |
| *PTP4A3* | Protein tyrosine phosphatase type IVA, member 3 | 1.945 | 3.27E-12 |
| *PTPLA* | Protein tyrosine phosphatase-like (proline instead of catalytic arginine), member A | -1.311 | 0.00113 |
| *PTPLB* | Protein tyrosine phosphatase-like (proline instead of catalytic arginine), member b | -1.897 | 0.00025 |
| *PTPN21* | Protein tyrosine phosphatase, non-receptor type 21 | -1.266 | 0.00175 |
| *PTRHD1* | Peptidyl-tRNA hydrolase domain containing 1 | -1.559 | 0.00014 |
| *PXMP2* | Peroxisomal membrane protein 2, 22kDa | 1.273 | 0.01347 |
| *PYGM* | Phosphorylase, glycogen, muscle | 1.269 | 0.00249 |
| *RAB13* | RAB13, member RAS oncogene family | -1.302 | 0.00803 |
| *RAB26* | RAB26, member RAS oncogene family | -1.578 | 0.00416 |
| *RAB2A* | RAB2A, member RAS oncogene family | 1.494 | 0.002 |
| *RAB3GAP1* | RAB3 GTPase activating protein subunit 1 (catalytic) | -1.284 | 0.00083 |
| *RABGEF1* | RAB guanine nucleotide exchange factor (GEF) 1 | -1.466 | 1.07E-07 |
| *RABGGTA* | Rab geranylgeranyltransferase, alpha subunit | 1.309 | 0.00022 |
| *RACGAP1* | Rac GTPase activating protein 1 | -1.502 | 1.18E-05 |
| *RAD1* | RAD1 homolog (S. pombe) | -1.523 | 0.01005 |
| *RAD17* | RAD17 homolog (S. pombe) | -1.284 | 0.01255 |
| *RAD23A* | RAD23 homolog A (S. cerevisiae) | 1.294 | 0.00224 |
| *RADIL* | Ras association and DIL domains | 1.463 | 0.00568 |
| *RAF1* | V-raf-1 murine leukemia viral oncogene homolog 1 | 1.258 | 0.00052 |
| *RAMP1* | Receptor (G protein-coupled) activity modifying protein 1 | 1.588 | 3.63E-07 |
| *RANGRF* | RAN guanine nucleotide release factor | 1.36 | 0.00553 |
| *RASA4* | RAS p21 protein activator 4 | 1.556 | 8.06E-06 |
| *RASIP1* | Ras interacting protein 1 | -1.48 | 1.48E-06 |
| *RASSF3* | Ras association (RalGDS/AF-6) domain family member 3 | -1.318 | 0.01489 |
| *RAVER2* | Ribonucleoprotein, PTB-binding 2 | 1.338 | 0.00785 |
| *RBBP8* | Retinoblastoma binding protein 8 | -2.011 | 1.84E-07 |
| *RBL1* | Retinoblastoma-like 1 (p107) | -1.583 | 0.0035 |
| *RBM12* | RNA binding motif protein 12 | -1.3 | 0.00698 |
| *RBM20* | RNA binding motif protein 20 | 1.361 | 5.12E-05 |
| *RBMS1* | RNA binding motif, single stranded interacting protein 1 | -1.287 | 0.01378 |
| *RCAN2* | Regulator of calcineurin 2 | -1.884 | 0.00052 |
| *RCC2* | Regulator of chromosome condensation 2 | -1.485 | 0.00046 |
| *RCN1* | Reticulocalbin 1, EF-hand calcium binding domain | -1.389 | 0.00082 |
| *RECQL* | RecQ protein-like (DNA helicase Q1-like) | -1.459 | 0.0039 |
| *REEP1* | Receptor accessory protein 1 | -1.639 | 5.17E-09 |
| *REPS1* | RALBP1 associated Eps domain containing 1 | 1.374 | 0.00027 |
| *RET* | Ret proto-oncogene | -1.458 | 0.01054 |
| *RFX5* | Regulatory factor X, 5 (influences HLA class II expression) | -1.478 | 0.00112 |
| *RFXANK* | Regulatory factor X-associated ankyrin-containing protein | -1.869 | 1.09E-05 |
| *RGL2* | Ral guanine nucleotide dissociation stimulator-like 2 | 1.344 | 0.00011 |
| *RHBDF2* | Rhomboid 5 homolog 2 (Drosophila) | -1.802 | 0.00074 |
| *RHOBTB1* | Rho-related BTB domain containing 1 | -1.358 | 7.50E-06 |
| *RIBC1* | RIB43A domain with coiled-coils 1 | 1.39 | 0.00573 |
| *RIPK2* | Receptor-interacting serine-threonine kinase 2 | -1.7 | 0.0001 |
| *RMND5A* | Required for meiotic nuclear division 5 homolog A (S. cerevisiae) | 1.409 | 0.00675 |
| *RNASEH2A* | Ribonuclease H2, subunit A | 1.313 | 0.00635 |
| *RND2* | Rho family GTPase 2 | -2.654 | 3.48E-06 |
| *RNF130* | Ring finger protein 130 | -1.4 | 2.70E-05 |
| *RNF19B* | Ring finger protein 19B | -1.29 | 0.00094 |
| *RNF217* | Ring finger protein 217 | 1.38 | 0.00048 |
| *RNF7* | Ring finger protein 7 | 1.291 | 0.00032 |
| *RNPC3* | RNA-binding region (RNP1, RRM) containing 3 | -2.156 | 0.00039 |
| *ROCK1* | Rho-associated, coiled-coil containing protein kinase 1 | -1.772 | 0.00116 |
| *RORC* | RAR-related orphan receptor C | 2.001 | 2.04E-05 |
| *RPF2* | Ribosome production factor 2 homolog (S. cerevisiae) | -1.363 | 0.00062 |
| *RPL28* | Ribosomal protein L28 | 1.28 | 0.00725 |
| *RPL3* | Ribosomal protein L3 | -1.309 | 0.00292 |
| *RPL3L* | Ribosomal protein L3-like | 1.745 | 1.08E-12 |
| *RPS19* | Ribosomal protein S19 | 1.275 | 0.00111 |
| *RPS2* | Ribosomal protein S2 | 1.341 | 7.56E-05 |
| *RPS20* | Ribosomal protein S20 | 1.323 | 8.83E-05 |
| *RPS6KA1* | Ribosomal protein S6 kinase, 90kDa, polypeptide 1 | -1.644 | 0.00012 |
| *RPSA* | Ribosomal protein SA | 1.313 | 0.00072 |
| *RRAGB* | Ras-related GTP binding B | 1.608 | 0.00573 |
| *Rrbp1* | Ribosome binding protein 1 | -1.427 | 0.00037 |
| *RRP15* | Ribosomal RNA processing 15 homolog (S. cerevisiae) | -1.272 | 0.00564 |
| *RSBN1L* | Round spermatid basic protein 1-like | -1.299 | 0.01084 |
| *RSU1* | Ras suppressor protein 1 | -1.359 | 0.00014 |
| *RTN2* | Reticulon 2 | 1.268 | 0.00037 |
| *RUSC2* | RUN and SH3 domain containing 2 | 1.363 | 0.00037 |
| *S100A10* | S100 calcium binding protein A10 | -1.392 | 0.01494 |
| *S100A11* | S100 calcium binding protein A11 | -1.412 | 0.00578 |
| *S100A14* | S100 calcium binding protein A14 | -1.356 | 0.00703 |
| *S1PR1* | Sphingosine-1-phosphate receptor 1 | 1.251 | 0.00173 |
| *S1PR2* | Sphingosine-1-phosphate receptor 2 | 1.491 | 0.00035 |
| *SAAL1* | Serum amyloid A-like 1 | 1.61 | 0.00035 |
| *SACS* | Spastic ataxia of Charlevoix-Saguenay (sacsin) | -1.267 | 0.00959 |
| *SCMH1* | Sex comb on midleg homolog 1 (Drosophila) | 1.265 | 0.00151 |
| *SCNM1* | Sodium channel modifier 1 | 1.259 | 0.00233 |
| *SDHB* | Succinate dehydrogenase complex, subunit B, iron sulfur (Ip) | 1.405 | 1.19E-05 |
| *SDHD* | Succinate dehydrogenase [ubiquinone] cytochrome b small subunit, mitochondrial | 1.284 | 0.0037 |
| *SDPR* | Serum deprivation response | -1.381 | 0.00908 |
| *SDS* | Serine dehydratase | -2.191 | 0.01356 |
| *SEC61A2* | Sec61 alpha 2 subunit (S. cerevisiae) | -1.252 | 0.00082 |
| *SECTM1* | Secreted and transmembrane 1 | 1.515 | 0.00728 |
| *SELENBP1* | Selenium binding protein 1 | 1.539 | 2.96E-05 |
| *SELT* | Selenoprotein T | -1.44 | 0.00207 |
| *SEMA7A* | Semaphorin 7A, GPI membrane anchor (John Milton Hagen blood group) | 1.326 | 0.00146 |
| *SENP6* | SUMO1/sentrin specific peptidase 6 | -1.584 | 0.00095 |
| *Sep-11* | Septin 11 | 1.484 | 5.21E-05 |
| *Sep-05* | Septin 5 | -2.201 | 1.11E-05 |
| *SERF2* | Small EDRK-rich factor 2 | -1.331 | 0.00019 |
| *SERINC2* | Serine incorporator 2 | 1.487 | 0.00024 |
| *SERINC5* | Serine incorporator 5 | -1.581 | 0.0013 |
| *SERPINB6* | Serpin peptidase inhibitor, clade B (ovalbumin), member 6 | 1.3 | 5.52E-05 |
| *SERPINE1* | Serpin peptidase inhibitor, clade E (nexin, plasminogen activator inhibitor type 1), member 1 | -2.324 | 0.00011 |
| *SERPINF1* | Serpin peptidase inhibitor, clade F (alpha-2 antiplasmin, pigment epithelium derived factor), 1 | -1.505 | 0.00011 |
| *SERPINH1* | Serpin peptidase inhibitor, clade H (HSP 47), member 1, (collagen binding protein 1) | -1.43 | 0.00014 |
| *SERTAD3* | SERTA domain containing 3 | -1.304 | 0.01161 |
| *SETD4* | SET domain containing 4 | -1.637 | 1.19E-05 |
| *SETD8* | SET domain containing (lysine methyltransferase) 8 | -1.323 | 7.73E-05 |
| *SF3B5* | Splicing factor 3b, subunit 5, 10kDa | -1.409 | 0.00015 |
| *SFRP1* | Secreted frizzled-related protein 1 | 1.882 | 0.00043 |
| *SGSM3* | Small G protein signaling modulator 3 | 1.273 | 0.00311 |
| *SH3RF3* | SH3 domain containing ring finger 3 | 1.267 | 0.01056 |
| *SHB* | Src homology 2 domain containing adaptor protein B | -1.619 | 0.00254 |
| *SHQ1* | SHQ1, H/ACA ribonucleoprotein assembly factor | -1.44 | 0.00277 |
| *SHROOM2* | Shroom family member 2 | -1.391 | 0.00597 |
| *SIPA1* | Signal-induced proliferation-associated 1 | -1.265 | 0.00704 |
| *SIPA1L2* | Signal-induced proliferation-associated 1 like 2 | -1.414 | 0.00878 |
| *SIRPA* | Signal-regulatory protein alpha | -1.61 | 0.00254 |
| *SIX1* | SIX homeobox 1 | -1.324 | 2.19E-05 |
| *SIX2* | SIX homeobox 2 | -2.814 | 7.44E-14 |
| *SKAP2* | Src kinase associated phosphoprotein 2 | -1.354 | 0.0049 |
| *SKIL* | SKI-like oncogene | -1.732 | 0.00017 |
| *SLC12A2* | Solute carrier family 12 (sodium/potassium/chloride transporter), member 2 | -1.596 | 0.00074 |
| *SLC12A4* | Solute carrier family 12 (potassium/chloride transporter), member 4 | 1.385 | 0.00113 |
| *SLC16A6* | Solute carrier family 16, member 6 | -3.2 | 7.01E-08 |
| *SLC20A2* | Solute carrier family 20 (phosphate transporter), member 2 | 1.413 | 2.94E-05 |
| *SLC22A17* | Solute carrier family 22, member 17 | -1.555 | 0.00808 |
| *SLC22A23* | Solute carrier family 22, member 23 | -1.637 | 4.20E-06 |
| *SLC22A4* | Solute carrier family 22 (organic cation/zwitterion transporter), member 4 | 1.812 | 0.00042 |
| *SLC25A11* | Solute carrier family 25 (mitochondrial carrier; oxoglutarate carrier), member 11 | 1.331 | 0.00036 |
| *SLC25A12* | Solute carrier family 25 (aspartate/glutamate carrier), member 12 | 1.329 | 0.00029 |
| *SLC25A17* | Solute carrier family 25 (mitochondrial carrier; peroxisomal membrane protein, 34kDa), 17 | -1.302 | 0.01075 |
| *SLC25A28* | Solute carrier family 25 (mitochondrial iron transporter), member 28 | 1.357 | 0.00069 |
| *SLC25A6* | Solute carrier family 25 (mitochondrial carrier; adenine nucleotide translocator), member 6 | 1.293 | 0.01328 |
| *SLC26A6* | Solute carrier family 26 (anion exchanger), member 6 | 1.317 | 0.00267 |
| *SLC2A3* | Solute carrier family 2 (facilitated glucose transporter), member 3 | -1.377 | 0.01375 |
| *SLC2A4* | Solute carrier family 2 (facilitated glucose transporter), member 4 | 1.611 | 9.89E-08 |
| *SLC2A4RG* | SLC2A4 regulator | 1.399 | 0.00099 |
| *SLC2A8* | Solute carrier family 2 (facilitated glucose transporter), member 8 | 1.398 | 0.00547 |
| *SLC35E3* | Solute carrier family 35, member E3 | -1.717 | 1.29E-06 |
| *SLC37A4* | Solute carrier family 37 (glucose-6-phosphate transporter), member 4 | -1.354 | 4.67E-05 |
| *SLC38A6* | Solute carrier family 38, member 6 | -1.501 | 0.00449 |
| *SLC4A4* | Solute carrier family 4 (sodium bicarbonate cotransporter), member 4 | -2.457 | 3.63E-12 |
| *SLC6A8* | Solute carrier family 6 (neurotransmitter transporter), member 8 | 1.29 | 0.00243 |
| *SLC7A1* | Solute carrier family 7 (cationic amino acid transporter, y+ system), member 1 | 1.418 | 0.0003 |
| *SLC7A4* | Solute carrier family 7, member 4 | -2.098 | 0.00248 |
| *SLC7A8* | Solute carrier family 7 (amino acid transporter light chain, L system), member 8 | -2.007 | 4.33E-06 |
| *SLC9A2* | Solute carrier family 9, subfamily A (NHE2, cation proton antiporter 2), member 2 | -1.359 | 0.01368 |
| *SLC9A5* | Solute carrier family 9, subfamily A (NHE5, cation proton antiporter 5), member 5 | -1.785 | 0.00012 |
| *SLIT3* | Slit homolog 3 (Drosophila) | -1.441 | 0.0137 |
| *SMARCAD1* | SWI/SNF-related, matrix-associated actin-dependent regulator of chromatin, subfamily a, containing DEAD/H box 1 | -1.483 | 0.01468 |
| *SMC1A* | Structural maintenance of chromosomes 1A | -1.384 | 0.00044 |
| *SMIM11* | Small integral membrane protein 11 | -1.251 | 0.01185 |
| *SMIM12* | Small integral membrane protein 12 | 1.336 | 0.00028 |
| *SMTN* | Smoothelin | 1.283 | 0.00716 |
| *SMTNL1* | Smoothelin-like 1 | 1.673 | 0.00049 |
| *SNAI3* | Snail family zinc finger 3 | 2.022 | 9.65E-09 |
| *SNAPC5* | Small nuclear RNA activating complex, polypeptide 5, 19kDa | -1.533 | 0.00027 |
| *SNAPIN* | SNAP-associated protein | 1.352 | 1.97E-06 |
| *SNTB2* | Syntrophin, beta 2 (dystrophin-associated protein A1, 59kDa, basic component 2) | 1.489 | 0.01396 |
| *SNX1* | Sorting nexin 1 | 1.687 | 3.23E-09 |
| *SNX25* | Sorting nexin 25 | -1.393 | 0.01038 |
| *SNX8* | Sorting nexin 8 | 1.447 | 0.00241 |
| *SOCS2* | Suppressor of cytokine signaling 2 | 2.746 | 5.98E-16 |
| *SOD3* | Superoxide dismutase 3, extracellular | 1.404 | 0.00129 |
| *SOGA2* | SOGA family member 2 | -1.584 | 0.00016 |
| *SORD* | Sorbitol dehydrogenase | -1.377 | 0.00891 |
| *SORL1* | Sortilin-related receptor, L(DLR class) A repeats containing | -1.564 | 0.00472 |
| *SPAG8* | Sperm associated antigen 8 | -2.093 | 3.68E-08 |
| *SPARC* | Secreted protein, acidic, cysteine-rich (osteonectin) | -1.474 | 0.00119 |
| *SPATS2* | Spermatogenesis associated, serine-rich 2 | -2.744 | 2.72E-13 |
| *SPG21* | Spastic paraplegia 21 (autosomal recessive, Mast syndrome) | -1.341 | 0.00363 |
| *SPICE1* | Spindle and centriole associated protein 1 | -1.375 | 0.01268 |
| *SPINT2* | Serine peptidase inhibitor, Kunitz type, 2 | 1.58 | 3.29E-05 |
| *SPOCK2* | Sparc/osteonectin, cwcv and kazal-like domains proteoglycan (testican) 2 | -1.757 | 0.00317 |
| *SPON1* | Spondin 1, extracellular matrix protein | -1.495 | 0.00226 |
| *SPSB3* | SplA/ryanodine receptor domain and SOCS box containing 3 | 1.367 | 0.00257 |
| *SRD5A1* | Steroid-5-alpha-reductase, alpha polypeptide 1 (3-oxo-5 alpha-steroid delta 4-dehydrogenase alpha 1) | -1.377 | 0.00083 |
| *SREBF1* | Sterol regulatory element binding transcription factor 1 | 1.343 | 0.01024 |
| *SRSF11* | Serine/arginine-rich splicing factor 11 | -1.352 | 0.00222 |
| *SRXN1* | Sulfiredoxin 1 | -1.903 | 0.00066 |
| *SSFA2* | Sperm specific antigen 2 | -1.384 | 0.00022 |
| *ST6GALNAC4* | ST6 (alpha-N-acetyl-neuraminyl-2,3-beta-galactosyl-1,3)-N-acetylgalactosaminide alpha-2,6-sialyltransferase 4 | -1.66 | 2.10E-06 |
| *ST8SIA2* | ST8 alpha-N-acetyl-neuraminide alpha-2,8-sialyltransferase 2 | -2.053 | 1.32E-05 |
| *STAT5A* | Signal transducer and activator of transcription 5A | 1.276 | 0.00295 |
| *STAT5B* | Signal transducer and activator of transcription 5B | 1.543 | 4.31E-08 |
| *STK38* | Serine/threonine kinase 38 | -1.332 | 0.00223 |
| *STMN2* | Stathmin-like 2 | 1.591 | 0.00214 |
| *STRADA* | STE20-related kinase adaptor alpha | 1.416 | 0.00094 |
| *STRBP* | Spermatid perinuclear RNA binding protein | -1.483 | 2.87E-05 |
| *STXBP1* | Syntaxin binding protein 1 | -1.424 | 0.00664 |
| *SUOX* | Sulfite oxidase | -1.27 | 0.00401 |
| *SUPT3H* | Suppressor of Ty 3 homolog (S. cerevisiae) | -1.464 | 0.00469 |
| *SUV39H1* | Suppressor of variegation 3-9 homolog 1 (Drosophila) | 2.126 | 9.22E-09 |
| *SUZ12* | SUZ12 polycomb repressive complex 2 subunit | -1.365 | 0.01254 |
| *SWI5* | SWI5 recombination repair homolog (yeast) | 1.387 | 3.57E-05 |
| *SYCP3* | Synaptonemal complex protein 3 | -1.428 | 0.00679 |
| *SYNGR1* | Synaptogyrin 1 | 1.462 | 1.07E-05 |
| *SYNPO* | Synaptopodin | 1.292 | 0.01137 |
| *SYT4* | Synaptotagmin IV | -1.508 | 0.00702 |
| *SYT7* | Synaptotagmin VII | -1.575 | 0.01109 |
| *SYTL4* | Synaptotagmin-like 4 | -1.473 | 0.0001 |
| *SZT2* | Seizure threshold 2 homolog (mouse) | -1.293 | 0.01394 |
| *TACO1* | Translational activator of mitochondrially encoded cytochrome c oxidase I | 1.327 | 0.00334 |
| *TAF1* | TAF1 RNA polymerase II, TATA box binding protein (TBP)-associated factor, 250kDa | -1.581 | 4.35E-06 |
| *TAF13* | TAF13 RNA polymerase II, TATA box binding protein (TBP)-associated factor, 18kDa | -1.487 | 0.00118 |
| *TAF4* | TAF4 RNA polymerase II, TATA box binding protein (TBP)-associated factor, 135kDa | 1.252 | 0.00417 |
| *TARBP1* | TAR (HIV-1) RNA binding protein 1 | -1.344 | 0.0128 |
| *TATDN3* | TatD DNase domain containing 3 | -1.275 | 0.00335 |
| *TBC1D12* | TBC1 domain family, member 12 | -1.372 | 0.01285 |
| *TBC1D17* | TBC1 domain family, member 17 | 1.295 | 0.00493 |
| *TBRG4* | Transforming growth factor beta regulator 4 | 1.254 | 0.00628 |
| *TCAP* | Titin-cap | 1.63 | 2.56E-08 |
| *TCEA3* | Transcription elongation factor A (SII), 3 | 1.54 | 1.82E-09 |
| *TCP11L1* | T-complex 11, testis-specific-like 1 | -1.368 | 0.00059 |
| *TCTN1* | Tectonic family member 1 | -1.355 | 0.00138 |
| *TEAD4* | TEA domain family member 4 | -1.316 | 0.00222 |
| *TECR* | Trans-2,3-enoyl-CoA reductase | 1.282 | 0.00226 |
| *TFPI2* | Tissue factor pathway inhibitor 2 | 1.394 | 6.16E-05 |
| *TGFBI* | Transforming growth factor, beta-induced, 68kDa | 1.348 | 0.0103 |
| *TGM2* | Transglutaminase 2 | -1.327 | 0.00683 |
| *THADA* | Thyroid adenoma associated | -1.651 | 3.94E-07 |
| *THBS1* | Thrombospondin 1 | -1.987 | 3.58E-06 |
| *THRA* | Thyroid hormone receptor, alpha | 1.263 | 0.00125 |
| *THRSP* | Thyroid hormone responsive | -5.817 | 0.00284 |
| *TIMM17A* | Translocase of inner mitochondrial membrane 17 homolog A (yeast) | 1.312 | 0.00367 |
| *TIPARP* | TCDD-inducible poly(ADP-ribose) polymerase | -1.295 | 0.00935 |
| *TIPRL* | TIP41, TOR signaling pathway regulator-like (S. cerevisiae) | -1.402 | 6.79E-05 |
| *TKT* | Transketolase | -1.814 | 0.01169 |
| *TLE2* | Transducin-like enhancer of split 2 (E(sp1) homolog, Drosophila) | -1.43 | 0.00244 |
| *TLK1* | Tousled-like kinase 1 | -1.454 | 0.00076 |
| *TLN2* | Talin 2 | 1.361 | 0.00066 |
| *TM2D1* | TM2 domain containing 1 | -1.314 | 0.0015 |
| *TM7SF2* | Transmembrane 7 superfamily member 2 | 1.376 | 0.00047 |
| *TMBIM1* | Transmembrane BAX inhibitor motif containing 1 | 1.266 | 0.00742 |
| *TMED3* | Transmembrane emp24 protein transport domain containing 3 | -1.963 | 7.73E-05 |
| *TMEFF2* | Transmembrane protein with EGF-like and two follistatin-like domains 2 | 1.524 | 0.00802 |
| *TMEM120A* | Transmembrane protein 120A | 1.447 | 0.00188 |
| *TMEM126B* | Transmembrane protein 126B | -1.368 | 0.01246 |
| *TMEM135* | Transmembrane protein 135 | -1.433 | 0.00438 |
| *TMEM182* | Transmembrane protein 182 | 1.266 | 0.00337 |
| *TMEM223* | Transmembrane protein 223 | 1.291 | 0.00051 |
| *TMEM255A* | Transmembrane protein 255A | 2.323 | 5.33E-06 |
| *TMEM259* | Transmembrane protein 259 | 1.293 | 0.00173 |
| *TMEM33* | Transmembrane protein 33 | -1.421 | 0.00168 |
| *TMEM35* | Transmembrane protein 35 | -1.422 | 0.013 |
| *TMEM51* | Transmembrane protein 51 | -1.994 | 2.25E-07 |
| *TMEM63A* | Transmembrane protein 63A | 1.279 | 0.00291 |
| *TMEM8B* | Transmembrane protein 8B | 1.364 | 0.00164 |
| *TNNC1* | Troponin C type 1 (slow) | 1.41 | 3.16E-05 |
| *TNRC6A* | Trinucleotide repeat containing 6A | -1.289 | 0.0082 |
| *TNS1* | Tensin 1 | 1.452 | 7.09E-06 |
| *TOMM34* | Translocase of outer mitochondrial membrane 34 | -1.506 | 0.00209 |
| *TOMM40* | Translocase of outer mitochondrial membrane 40 homolog (yeast) | 1.36 | 0.00221 |
| *TOMM40L* | Translocase of outer mitochondrial membrane 40 homolog (yeast)-like | 1.304 | 0.00313 |
| *TOP1* | Topoisomerase (DNA) I | -1.326 | 0.01097 |
| *TP53INP2* | Tumor protein p53 inducible nuclear protein 2 | -1.482 | 0.00963 |
| *TPD52* | Tumor protein D52 | -1.469 | 0.00148 |
| *TPK1* | Thiamin pyrophosphokinase 1 | -1.348 | 0.01185 |
| *TPM4* | Tropomyosin 4 | -1.288 | 0.0027 |
| *TPMT* | Thiopurine S-methyltransferase | -1.41 | 0.00348 |
| *TPP1* | Tripeptidyl peptidase I | -1.378 | 1.17E-05 |
| *TPPP2* | Tubulin polymerization-promoting protein family member 2 | -3.331 | 1.94E-07 |
| *TPPP3* | Tubulin polymerization-promoting protein family member 3 | -1.6 | 2.38E-05 |
| *TPST2* | Tyrosylprotein sulfotransferase 2 | 1.292 | 0.00221 |
| *TPX2* | TPX2, microtubule-associated | -1.952 | 4.10E-08 |
| *TRAF3* | TNF receptor-associated factor 3 | -1.598 | 0.0012 |
| *TRAF5* | TNF receptor-associated factor 5 | 1.461 | 0.01048 |
| *TRIM44* | Tripartite motif containing 44 | -1.607 | 0.00024 |
| *TRIM45* | Tripartite motif containing 45 | 1.363 | 0.0008 |
| *TSPAN4* | Tetraspanin 4 | 1.365 | 0.00943 |
| *TSPAN6* | Tetraspanin 6 | -1.378 | 0.00398 |
| *TSPYL2* | TSPY-like 2 | 1.655 | 2.86E-08 |
| *TSPYL4* | TSPY-like 4 | 1.395 | 0.01331 |
| *TTC14* | Tetratricopeptide repeat domain 14 | -1.551 | 0.00963 |
| *TTC9* | Tetratricopeptide repeat domain 9 | 1.533 | 1.23E-05 |
| *TTF2* | Transcription termination factor, RNA polymerase II | -1.508 | 0.00066 |
| *TTI2* | TELO2 interacting protein 2 | -1.579 | 4.43E-05 |
| *TTLL1* | Tubulin tyrosine ligase-like family, member 1 | -1.45 | 0.00058 |
| *TTLL4* | Tubulin tyrosine ligase-like family, member 4 | -1.303 | 0.01154 |
| *TUFM* | Tu translation elongation factor, mitochondrial | 1.34 | 0.00106 |
| *TXLNB* | Taxilin beta | 1.282 | 0.0014 |
| *UACA* | Uveal autoantigen with coiled-coil domains and ankyrin repeats | -1.604 | 0.00578 |
| *UAP1L1* | UDP-N-acteylglucosamine pyrophosphorylase 1-like 1 | 1.565 | 5.47E-05 |
| *UBA52* | Ubiquitin A-52 residue ribosomal protein fusion product 1 | 1.356 | 6.44E-05 |
| *UBC* | Ubiquitin C | 1.276 | 0.00545 |
| *UBE2L6* | Ubiquitin-conjugating enzyme E2L 6 | 1.388 | 0.00142 |
| *UBE2M* | Ubiquitin-conjugating enzyme E2M | 1.278 | 0.00114 |
| *UBE2T* | Ubiquitin-conjugating enzyme E2T (putative) | 1.337 | 0.0003 |
| *UBIAD1* | UbiA prenyltransferase domain containing 1 | 1.288 | 0.00942 |
| *UBXN2B* | UBX domain protein 2B | -1.352 | 0.01314 |
| *UCK2* | Uridine-cytidine kinase 2 | -1.928 | 4.42E-06 |
| *UCP2* | Uncoupling protein 2 (mitochondrial, proton carrier) | 1.717 | 3.53E-07 |
| *UCP3* | Uncoupling protein 3 (mitochondrial, proton carrier) | -1.85 | 0.00089 |
| *UEVLD* | UEV and lactate/malate dehyrogenase domains | -1.503 | 0.00061 |
| *UFSP1* | UFM1-specific peptidase 1 (non-functional) | 1.441 | 0.00016 |
| *UFSP2* | UFM1-specific peptidase 2 | -1.358 | 0.00969 |
| *UNC13B* | Unc-13 homolog B (C. elegans) | -1.63 | 1.14E-05 |
| *UPF3A* | UPF3 regulator of nonsense transcripts homolog A (yeast) | -1.689 | 1.23E-10 |
| *UQCR11* | Cytochrome b-c1 complex subunit 10 | 1.297 | 0.01163 |
| *UQCRC1* | Ubiquinol-cytochrome c reductase core protein I | 1.329 | 0.00266 |
| *UQCRC2* | Ubiquinol-cytochrome c reductase core protein II | 1.285 | 0.00669 |
| *UQCRQ* | Ubiquinol-cytochrome c reductase, complex III subunit VII, 9.5kDa | 1.283 | 0.00328 |
| *USP18* | Ubiquitin specific peptidase 18 | 1.991 | 0.00011 |
| *USP48* | Ubiquitin specific peptidase 48 | -1.61 | 1.47E-07 |
| *VAMP1* | Vesicle-associated membrane protein 1 (synaptobrevin 1) | 1.633 | 0.0002 |
| *VASH1* | Vasohibin 1 | -1.767 | 0.00027 |
| *VDAC2* | Voltage-dependent anion channel 2 | 1.331 | 1.20E-05 |
| *Vegfb* | Vascular endothelial growth factor B | 1.619 | 5.77E-08 |
| *VIT* | Vitrin | 1.443 | 0.00123 |
| *VMP1* | Vacuole membrane protein 1 | -1.302 | 0.00957 |
| *VNN1* | Vanin 1 | 1.501 | 0.00016 |
| *VOPP1* | Vesicular, overexpressed in cancer, prosurvival protein 1 | 1.542 | 0.00031 |
| *VPS41* | Vacuolar protein sorting 41 homolog (S. cerevisiae) | -1.252 | 0.0024 |
| *VPS51* | Vacuolar protein sorting 51 homolog (S. cerevisiae) | -1.268 | 0.00785 |
| *VPS52* | Vacuolar protein sorting 52 homolog (S. cerevisiae) | -1.49 | 0.00233 |
| *VRK2* | Vaccinia related kinase 2 | -1.5 | 0.00253 |
| *VSTM2L* | V-set and transmembrane domain containing 2 like | 1.519 | 0.00096 |
| *WBSCR17* | Williams-Beuren syndrome chromosome region 17 | -2.107 | 6.26E-09 |
| *WDR12* | WD repeat domain 12 | -1.277 | 0.00902 |
| *WDR62* | WD repeat domain 62 | -1.53 | 0.00559 |
| *WIBG* | Within bgcn homolog (Drosophila) | 1.325 | 0.0036 |
| *WNK2* | WNK lysine deficient protein kinase 2 | 1.424 | 1.18E-05 |
| *XIRP1* | Xin actin-binding repeat containing 1 | 1.395 | 0.00622 |
| *XIRP2* | Xin actin-binding repeat containing 2 | -1.809 | 0.00036 |
| *XPNPEP3* | X-prolyl aminopeptidase (aminopeptidase P) 3, putative | -1.398 | 0.00119 |
| *XRCC6BP1* | XRCC6 binding protein 1 | -1.266 | 0.00487 |
| *YBEY* | YbeY metallopeptidase (putative) | 1.489 | 4.42E-05 |
| *YBX3* | Y box binding protein 3 | 1.539 | 6.86E-08 |
| *YEATS2* | YEATS domain containing 2 | -1.498 | 1.04E-06 |
| *YPEL3* | Yippee-like 3 (Drosophila) | 1.322 | 0.00232 |
| *YWHAH* | Tyrosine 3-monooxygenase/tryptophan 5-monooxygenase activation protein, eta | 1.272 | 0.01194 |
| *ZBTB1* | Zinc finger and BTB domain containing 1 | -1.413 | 0.00148 |
| *ZBTB40* | Zinc finger and BTB domain containing 40 | -1.32 | 0.00796 |
| *ZC3H15* | Zinc finger CCCH-type containing 15 | -1.345 | 0.00096 |
| *ZC3H3* | Zinc finger CCCH-type containing 3 | 1.265 | 0.01083 |
| *ZC3H6* | Zinc finger CCCH-type containing 6 | 1.577 | 3.98E-05 |
| *ZCCHC12* | Zinc finger, CCHC domain containing 12 | -1.45 | 0.01401 |
| *ZCCHC17* | Zinc finger, CCHC domain containing 17 | 1.328 | 2.28E-05 |
| *ZFC3H1* | Zinc finger, C3H1-type containing | -1.487 | 0.00677 |
| *ZFHX2* | Zinc finger homeobox 2 | -1.705 | 0.00061 |
| *ZFP90* | ZFP90 zinc finger protein | -1.337 | 0.00604 |
| *ZFYVE19* | Zinc finger, FYVE domain containing 19 | 1.617 | 0.00023 |
| *ZFYVE26* | Zinc finger, FYVE domain containing 26 | -1.273 | 0.00017 |
| *ZHX1* | Zinc fingers and homeoboxes 1 | -1.415 | 0.00179 |
| *ZMAT2* | Zinc finger, matrin-type 2 | -1.258 | 0.00069 |
| *ZMIZ1* | Zinc finger, MIZ-type containing 1 | -1.355 | 0.00561 |
| *ZMIZ2* | Zinc finger, MIZ-type containing 2 | 1.348 | 0.0002 |
| *ZNF106* | Zinc finger protein 106 | -1.559 | 0.01199 |
| *ZNF143* | Zinc finger protein 143 | 1.27 | 0.00413 |
| *ZNF274* | Zinc finger protein 274 | 1.336 | 0.00734 |
| *ZNF382* | Zinc finger protein 382 | -1.886 | 0.00084 |
| *ZNF419* | Zinc finger protein 419 | 1.328 | 0.00181 |
| *ZNF45* | Zinc finger protein 45 | 1.309 | 0.00072 |
| *ZNF608* | Zinc finger protein 608 | -1.762 | 9.01E-05 |
| *ZNF641* | Zinc finger protein 641 | -1.268 | 0.00353 |
| *ZNF672* | Zinc finger protein 672 | 1.359 | 0.00055 |
| *ZNF710* | Zinc finger protein 710 | 1.931 | 2.40E-06 |
| *ZNRF2* | zinc and ring finger 2 | -1.388 | 0.00061 |
| *ZSCAN29* | Zinc finger and SCAN domain containing 29 | -1.445 | 0.00084 |
| *ZUFSP* | Zinc finger with UFM1-specific peptidase domain | -1.386 | 0.01392 |

^1^ Fold changes are up or down in restricted fed animals compared to *ad libitum* control animals
