# Supplementary material for: Effect of Dietary Restriction and Subsequent Re-Alimentation on the Transcriptional Profile of Bovine Skeletal Muscle
Source: PLoS One. 2016 Feb 12;11(2):e0149373. doi: 10.1371/journal.pone.0149373 (PMC4752344; doi:10.1371/journal.pone.0149373)
Supplement: S2 Table — (DOCX) [file pone.0149373.s002.docx]

**S2 Table.** Genes differentially expressed in *M. longissimus dorsi* of Holstein Friesian bulls (n = 10) following a 15-day period of re-alimentation and compensatory growth in Period 2 relative to *ad libitum*-fed controls (n = 10)

| Gene symbol | Gene name | Fold change^1^ | P value |
| --- | --- | --- | --- |
| *A4GALT* | Alpha 1,4-galactosyltransferase | 1.413 | 0.0053 |
| *AASDH* | Aminoadipate-semialdehyde dehydrogenase | -1.357 | 0.00719 |
| *ABCA1* | ATP-binding cassette, sub-family A (ABC1), member 1 | 2.501 | 8.99E-06 |
| *ABCA2* | ATP-binding cassette, sub-family A (ABC1), member 2 | 1.535 | 0.00494 |
| *ABCB7* | ATP-binding cassette, sub-family B (MDR/TAP), member 7 | -1.296 | 0.00557 |
| *ABCC1* | ATP-binding cassette, sub-family C (CFTR/MRP), member 1 | 1.322 | 0.00196 |
| *ABCC8* | ATP-binding cassette, sub-family C (CFTR/MRP), member 8 | 1.537 | 0.00055 |
| *ABCF2* | ATP-binding cassette, sub-family F (GCN20), member 2 | 1.4 | 0.00011 |
| *ABI2* | Abl-interactor 2 | 1.585 | 0.00664 |
| *ABR* | Active BCR-related | 1.303 | 0.00306 |
| *ABTB1* | Ankyrin repeat and BTB (POZ) domain containing 1 | 1.373 | 0.00225 |
| *ACAD10* | Acyl-CoA dehydrogenase family, member 10 | 1.296 | 0.005 |
| *ACADM* | Acyl-CoA dehydrogenase, C-4 to C-12 straight chain | -1.278 | 0.00436 |
| *ACAT1* | Acetyl-CoA acetyltransferase 1 | -1.626 | 2.59E-06 |
| *ACE* | Angiotensin I converting enzyme | 1.569 | 0.00014 |
| *ACLY* | ATP citrate lyase | 1.846 | 0.00776 |
| *ACSM1* | Acyl-CoA synthetase medium-chain family member 1 | 3.229 | 0.00155 |
| *ADAM33* | ADAM metallopeptidase domain 33 | 1.635 | 0.00818 |
| *ADAM9* | ADAM metallopeptidase domain 9 | -1.317 | 0.00483 |
| *ADAMTS10* | ADAM metallopeptidase with thrombospondin type 1 motif, 10 | 1.818 | 0.00037 |
| *ADAMTS9* | ADAM metallopeptidase with thrombospondin type 1 motif, 9 | -1.512 | 0.00074 |
| *ADCY5* | Adenylate cyclase 5 | 1.492 | 0.00192 |
| *ADCY6* | Adenylate cyclase 6 | 1.316 | 0.0023 |
| *ADK* | Adenosine kinase | -1.527 | 8.13E-06 |
| *ADPRHL1* | ADP-ribosylhydrolase like 1 | 1.34 | 0.00574 |
| *AEBP1* | AE binding protein 1 | 2.545 | 0.00068 |
| *AGPAT2* | 1-acylglycerol-3-phosphate O-acyltransferase 2 | 1.602 | 0.00021 |
| *AGRN* | Agrin | 1.718 | 1.52E-08 |
| *ALDH18A1* | Aldehyde dehydrogenase 18 family, member A1 | -1.334 | 0.00013 |
| *ALS2CL* | ALS2 C-terminal like | 1.425 | 0.00112 |
| *AMD1* | Adenosylmethionine decarboxylase 1 | -1.589 | 0.00034 |
| *AMPD2* | Adenosine monophosphate deaminase 2 | 1.531 | 0.00314 |
| *ANGPT1* | Angiopoietin 1 | -1.61 | 0.00228 |
| *ANGPT4* | Angiopoietin 4 | 1.4 | 0.00797 |
| *ANKRD11* | Ankyrin repeat domain 11 | 1.325 | 0.00183 |
| *ANKRD13B* | Ankyrin repeat domain 13B | 1.444 | 0.00106 |
| *ANXA3* | Annexin A3 | -1.341 | 0.00127 |
| *ANXA9* | Annexin A9 | -1.56 | 0.00176 |
| *AP5B1* | Adaptor-related protein complex 5, beta 1 subunit | 1.48 | 0.00035 |
| *AP5Z1* | Adaptor-related protein complex 5, zeta 1 subunit | 1.443 | 0.00024 |
| *APBB3* | Amyloid beta (A4) precursor protein-binding, family B, member 3 | 1.385 | 0.00641 |
| *APLP1* | Amyloid beta (A4) precursor-like protein 1 | 1.321 | 0.00095 |
| *APLP2* | Amyloid beta (A4) precursor-like protein 2 | 1.343 | 0.00046 |
| *APOO* | Apolipoprotein O | -1.489 | 0.00483 |
| *AQP4* | Aquaporin 4 | -1.655 | 0.00069 |
| *AQPEP* | Laeverin | -1.648 | 0.00276 |
| *ARG2* | Arginase 2 | -2.741 | 2.61E-06 |
| *ARHGAP1* | Rho GTPase activating protein 1 | 1.34 | 0.00797 |
| *ARHGEF37* | Rho guanine nucleotide exchange factor (GEF) 37 | 1.378 | 0.00023 |
| *ARID5B* | AT rich interactive domain 5B (MRF1-like) | 1.952 | 0.00063 |
| *ARL10* | ADP-ribosylation factor-like 10 | 1.801 | 0.0022 |
| *ARMCX3* | Armadillo repeat containing, X-linked 3 | -1.402 | 0.00699 |
| *ARRDC1* | Arrestin domain containing 1 | 1.336 | 0.00361 |
| *ARRDC2* | Arrestin domain containing 2 | 2.512 | 3.42E-11 |
| *ASB1* | Ankyrin repeat and SOCS box containing 1 | 1.344 | 0.00115 |
| *ASCC1* | Activating signal cointegrator 1 complex subunit 1 | 1.376 | 0.00888 |
| *ASF1B* | Anti-silencing function 1B histone chaperone | -1.945 | 2.17E-06 |
| *ASNS* | Asparagine synthetase (glutamine-hydrolyzing) | -3.261 | 0.00141 |
| *ASXL1* | Additional sex combs like 1 (Drosophila) | 1.277 | 0.00324 |
| *ATG13* | Autophagy related 13 | 1.26 | 0.00229 |
| *ATN1* | Atrophin 1 | 1.389 | 0.00244 |
| *ATP1B1* | ATPase, Na+/K+ transporting, beta 1 polypeptide | -1.316 | 0.00137 |
| *ATP2B2* | ATPase, Ca++ transporting, plasma membrane 2 | 1.719 | 0.00075 |
| *ATP2C2* | ATPase, Ca++ transporting, type 2C, member 2 | 2.12 | 7.89E-06 |
| *ATP5A1* | ATP synthase, H+ transporting, mitochondrial F1 complex, alpha subunit 1, cardiac muscle | -1.289 | 0.0032 |
| *ATP5B* | ATP synthase, H+ transporting, mitochondrial F1 complex, beta polypeptide | -1.322 | 0.00058 |
| *ATP5C1* | ATP synthase, H+ transporting, mitochondrial F1 complex, gamma polypeptide 1 | -1.273 | 0.00165 |
| *ATP5F1* | ATP synthase, H+ transporting, mitochondrial Fo complex, subunit B1 | -1.335 | 0.00018 |
| *ATP5J* | ATP synthase, H+ transporting, mitochondrial Fo complex, subunit F6 | -1.308 | 0.00051 |
| *ATP5J2* | ATP synthase, H+ transporting, mitochondrial Fo complex, subunit F2 | -1.27 | 0.00263 |
| *ATP5O* | ATP synthase, H+ transporting, mitochondrial F1 complex, O subunit | -1.314 | 0.00413 |
| *AZIN1* | Antizyme inhibitor 1 | -1.724 | 5.05E-05 |
| *B3GAT1* | Beta-1,3-glucuronyltransferase 1 (glucuronosyltransferase P) | 1.438 | 0.00108 |
| *B4GALT5* | UDP-Gal:betaGlcNAc beta 1,4- galactosyltransferase, polypeptide 5 | 1.396 | 0.00197 |
| *BAHD1* | Bromo adjacent homology domain containing 1 | 1.567 | 2.58E-05 |
| *BAIAP2* | BAI1-associated protein 2 | 1.512 | 0.00015 |
| *BCAM* | Basal cell adhesion molecule (Lutheran blood group) | 1.369 | 0.00031 |
| *BCAS3* | Breast carcinoma amplified sequence 3 | 1.294 | 0.00208 |
| *BGN* | Biglycan | 2.097 | 0.00825 |
| *BMP2K* | BMP2 inducible kinase | 2.317 | 0.00913 |
| *BOLA3* | BolA family member 3 | -1.314 | 0.00405 |
| *BRD3* | Bromodomain containing 3 | 1.387 | 0.00148 |
| *BRPF3* | Bromodomain and PHD finger containing, 3 | 1.325 | 0.00055 |
| *BTG1* | B-cell translocation gene 1, anti-proliferative | 1.939 | 0.00422 |
| *C10orf10* | Chromosome 10 open reading frame 10 | 1.566 | 0.00107 |
| *C11orf52* | Chromosome 11 open reading frame 52 | 1.503 | 0.00671 |
| *C15orf52* | Chromosome 15 open reading frame 52 | 1.382 | 0.00065 |
| *C18orf8* | Chromosome 18 open reading frame 8 | 1.289 | 0.00369 |
| *C1orf35* | Chromosome 1 open reading frame 35 | 1.429 | 0.00514 |
| *C1QBP* | Complement component 1, q subcomponent binding protein | -1.305 | 6.62E-05 |
| *C8orf59* | Chromosome 8 open reading frame 59 | -1.602 | 0.00134 |
| *CABIN1* | Calcineurin binding protein 1 | 1.353 | 0.00097 |
| *CACNA1C* | Calcium channel, voltage-dependent, L type, alpha 1C subunit | 1.789 | 7.12E-05 |
| *CACNA1H* | Calcium channel, voltage-dependent, T type, alpha 1H subunit | 1.72 | 1.83E-06 |
| *CALCOCO1* | Calcium binding and coiled-coil domain 1 | 1.767 | 6.38E-08 |
| *CAMSAP1* | Calmodulin regulated spectrin-associated protein 1 | 1.427 | 3.30E-05 |
| *CARD11* | Caspase recruitment domain family, member 11 | 2.068 | 3.92E-05 |
| *CARS* | Cysteinyl-tRNA synthetase | -1.588 | 0.0007 |
| *CASP8* | Caspase 8, apoptosis-related cysteine peptidase | -1.611 | 0.00784 |
| *CASQ2* | Calsequestrin 2 (cardiac muscle) | -2.174 | 9.12E-08 |
| *CBFA2T3* | Core-binding factor, runt domain, alpha subunit 2; translocated to, 3 | 1.538 | 0.00044 |
| *CBR4* | Carbonyl reductase 4 | -1.696 | 6.46E-09 |
| *CBS* | Cystathionine-beta-synthase | -2.268 | 0.00145 |
| *CBX5* | Chromobox homolog 5 | -1.262 | 0.00865 |
| *CBX7* | Chromobox homolog 7 | 1.566 | 0.00094 |
| *CC2D1A* | Coiled-coil and C2 domain containing 1A | 1.329 | 0.00262 |
| *CCDC3* | Coiled-coil domain containing 3 | 2.823 | 1.12E-06 |
| *CCDC43* | Coiled-coil domain containing 43 | -1.26 | 0.00789 |
| *CCDC57* | Coiled-coil domain containing 57 | 1.602 | 0.00373 |
| *CCDC58* | Coiled-coil domain containing 58 | -1.285 | 0.00158 |
| *CCDC86* | Coiled-coil domain containing 86 | -1.32 | 0.00358 |
| *CCND1* | Cyclin D1 | -1.885 | 0.00225 |
| *CCNG2* | Cyclin G2 | 1.535 | 0.00654 |
| *CD93* | CD93 molecule | -1.345 | 0.00624 |
| *CDC42EP3* | CDC42 effector protein (Rho GTPase binding) 3 | -1.272 | 0.00333 |
| *CDH4* | Cadherin 4, type 1, R-cadherin (retinal) | 1.811 | 3.94E-05 |
| *CDK3* | Cyclin-dependent kinase 3 | 1.55 | 0.00856 |
| *CDKN2D* | Cyclin-dependent kinase inhibitor 2D (p19, inhibits CDK4) | 1.264 | 0.00823 |
| *CDNF* | Cerebral dopamine neurotrophic factor | -1.389 | 0.00122 |
| *CEBPG* | CCAAT/enhancer binding protein (C/EBP), gamma | -1.601 | 5.12E-06 |
| *CENPQ* | Centromere protein Q | -1.888 | 0.00215 |
| *CEP250* | Centrosomal protein 250kDa | 1.396 | 0.00342 |
| *CETN3* | Centrin, EF-hand protein, 3 | -1.557 | 0.00197 |
| *CGREF1* | Cell growth regulator with EF-hand domain 1 | 1.396 | 0.00831 |
| *CHCHD3* | Coiled-coil-helix-coiled-coil-helix domain containing 3 | -1.314 | 0.00036 |
| *CHD3* | Chromodomain helicase DNA binding protein 3 | 1.552 | 9.36E-08 |
| *CHD4* | Chromodomain helicase DNA binding protein 4 | 1.285 | 0.00077 |
| *CHD7* | Chromodomain helicase DNA binding protein 7 | 1.313 | 0.00254 |
| *CHFR* | Checkpoint with forkhead and ring finger domains, E3 ubiquitin protein ligase | 1.34 | 0.00165 |
| *CHI3L1* | Chitinase 3-like 1 (cartilage glycoprotein-39) | 8.92 | 7.45E-05 |
| *CHMP4A* | Charged multivesicular body protein 4A | 1.359 | 0.00276 |
| *CHPF* | Chondroitin polymerizing factor | 1.445 | 0.00604 |
| *CHRD* | Chordin | 1.92 | 0.00852 |
| *CHRNE* | Cholinergic receptor, nicotinic, epsilon (muscle) | 1.84 | 5.94E-05 |
| *CHURC1* | Churchill domain containing 1 | -1.47 | 0.00238 |
| *CIDEA* | Cell death-inducing DFFA-like effector a | 1.955 | 0.00805 |
| *CIDEC* | Cell death-inducing DFFA-like effector c | 2.637 | 0.00692 |
| *CILP* | Cartilage intermediate layer protein, nucleotide pyrophosphohydrolase | -1.455 | 0.00693 |
| *CISD1* | CDGSH iron sulfur domain 1 | -1.582 | 0.00027 |
| *CIZ1* | CDKN1A interacting zinc finger protein 1 | 1.412 | 0.00437 |
| *CKS2* | CDC28 protein kinase regulatory subunit 2 | -1.953 | 8.85E-05 |
| *CLASRP* | CLK4-associating serine/arginine rich protein | 1.391 | 0.00754 |
| *CLCN7* | Chloride channel, voltage-sensitive 7 | 1.343 | 0.00015 |
| *CLIC4* | Chloride intracellular channel 4 | -1.483 | 0.0005 |
| *CLYBL* | Citrate lyase beta like | -1.387 | 9.84E-05 |
| *CMSS1* | Cms1 ribosomal small subunit homolog (yeast) | -1.343 | 0.00173 |
| *CMTM8* | CKLF-like MARVEL transmembrane domain containing 8 | -1.624 | 0.00268 |
| *CNNM3* | Cyclin M3 | 1.48 | 4.68E-05 |
| *COA3* | Cytochrome c oxidase assembly factor 3 | -1.253 | 0.00229 |
| *COA5* | Cytochrome c oxidase assembly factor 5 | -1.31 | 0.00064 |
| *COL15A1* | Collagen, type XV, alpha 1 | -1.354 | 0.00109 |
| *COQ3* | Coenzyme Q3 methyltransferase | -1.329 | 0.00114 |
| *COQ5* | Coenzyme Q5 homolog, methyltransferase (S. cerevisiae) | -1.346 | 7.37E-05 |
| *COQ6* | Coenzyme Q6 monooxygenase | -1.458 | 0.00172 |
| *COX10* | COX10 heme A:farnesyltransferase cytochrome c oxidase assembly factor | -1.257 | 0.00578 |
| *COX15* | Cytochrome c oxidase assembly homolog 15 (yeast) | -1.372 | 0.00192 |
| *COX5A* | Cytochrome c oxidase subunit Va | -1.455 | 1.47E-05 |
| *COX6B1* | Cytochrome c oxidase subunit VIb polypeptide 1 (ubiquitous) | -1.412 | 0.0002 |
| *COX7A1* | Cytochrome c oxidase subunit VIIa polypeptide 1 (muscle) | -1.253 | 0.00509 |
| *COX7B* | Cytochrome c oxidase subunit VIIb | -1.432 | 0.00676 |
| *CPSF1* | Cleavage and polyadenylation specific factor 1, 160kDa | 1.336 | 0.00326 |
| *CPT1B* | Carnitine palmitoyltransferase 1B (muscle) | -1.358 | 0.00142 |
| *CRABP2* | Cellular retinoic acid binding protein 2 | 4.134 | 0.00085 |
| *CRIM1* | Cysteine rich transmembrane BMP regulator 1 (chordin-like) | 1.456 | 0.00045 |
| *CROCC* | Ciliary rootlet coiled-coil, rootletin | 1.718 | 0.00043 |
| *CRTC2* | CREB regulated transcription coactivator 2 | 1.308 | 0.00697 |
| *CRYL1* | Crystallin, lambda 1 | 1.442 | 0.00151 |
| *CS* | Citrate synthase | -1.657 | 8.75E-09 |
| *CSAD* | Cysteine sulfinic acid decarboxylase | 1.36 | 0.00221 |
| *CSRNP1* | Cysteine-serine-rich nuclear protein 1 | 1.516 | 0.00573 |
| *CSRP1* | Cysteine and glycine-rich protein 1 | 1.296 | 0.00625 |
| *CTSD* | Cathepsin D | 1.46 | 0.00045 |
| *CTSF* | Cathepsin F | 1.342 | 0.00137 |
| *CTXN3* | Cortexin 3 | 2.419 | 0.00354 |
| *CUL9* | Cullin 9 | 1.333 | 0.00386 |
| *CYB5R1* | Cytochrome b5 reductase 1 | 1.305 | 0.00502 |
| *CYB5R3* | Cytochrome b5 reductase 3 | 1.409 | 0.00021 |
| *CYB5R4* | Cytochrome b5 reductase 4 | -1.363 | 0.00444 |
| *CYCS* | Cytochrome c, somatic | -1.547 | 3.76E-05 |
| *CYP26B1* | Cytochrome P450, family 26, subfamily B, polypeptide 1 | 2.65 | 4.18E-06 |
| *CYP51A1* | Cytochrome P450, family 51, subfamily A, polypeptide 1 | -1.943 | 7.19E-06 |
| *CYR61* | Cysteine-rich, angiogenic inducer, 61 | 1.759 | 0.00036 |
| *DARS2* | Aspartyl-tRNA synthetase 2, mitochondrial | -1.341 | 0.00394 |
| *DBI* | Diazepam binding inhibitor (GABA receptor modulator, acyl-CoA binding protein) | -1.327 | 0.00566 |
| *DBNDD1* | Dysbindin (dystrobrevin binding protein 1) domain containing 1 | 1.311 | 0.00593 |
| *DBP* | D site of albumin promoter (albumin D-box) binding protein | 1.544 | 0.00036 |
| *DCTN5* | Dynactin 5 (p25) | -1.306 | 0.00162 |
| *DDI2* | DNA-damage inducible 1 homolog 2 (S. cerevisiae) | 1.641 | 0.00131 |
| *DDO* | D-aspartate oxidase | -1.27 | 0.00734 |
| *DDR1* | Discoidin domain receptor tyrosine kinase 1 | 1.382 | 0.00159 |
| *DDX25* | DEAD (Asp-Glu-Ala-Asp) box helicase 25 | 1.575 | 0.00211 |
| *DGAT1* | Diacylglycerol O-acyltransferase 1 | 1.402 | 0.00369 |
| *DGKD* | Diacylglycerol kinase, delta 130kDa | 1.349 | 0.00044 |
| *DGKQ* | Diacylglycerol kinase, theta 110kDa | 1.556 | 9.57E-05 |
| *DHCR24* | 24-dehydrocholesterol reductase | -1.519 | 0.0017 |
| *DHDH* | Dihydrodiol dehydrogenase (dimeric) | -1.754 | 1.06E-07 |
| *DHRS3* | Dehydrogenase/reductase (SDR family) member 3 | 1.485 | 0.00151 |
| *DIMT1* | DIM1 dimethyladenosine transferase 1 homolog (S. cerevisiae) | -1.273 | 0.0088 |
| *DLAT* | Dihydrolipoamide S-acetyltransferase | -1.297 | 0.00124 |
| *DLD* | Dihydrolipoamide dehydrogenase | -1.426 | 0.00126 |
| *DLG5* | Discs, large homolog 5 (Drosophila) | 1.338 | 0.00035 |
| *DNAJB4* | DnaJ (Hsp40) homolog, subfamily B, member 4 | -1.611 | 4.70E-05 |
| *DNAJB5* | DnaJ (Hsp40) homolog, subfamily B, member 5 | -1.332 | 0.00808 |
| *DOK4* | Docking protein 4 | -1.673 | 0.00019 |
| *DST* | Dystonin | 1.363 | 8.06E-05 |
| *DTNB* | Dystrobrevin, beta | 1.459 | 0.00319 |
| *DUSP26* | Dual specificity phosphatase 26 (putative) | 1.422 | 0.00905 |
| *DYNC1H1* | Dynein, cytoplasmic 1, heavy chain 1 | 1.255 | 0.00243 |
| *EBAG9* | Estrogen receptor binding site associated, antigen, 9 | -1.333 | 0.00522 |
| *EEF1B2* | Eukaryotic translation elongation factor 1 beta 2 | -1.252 | 0.00307 |
| *EGFLAM* | EGF-like, fibronectin type III and laminin G domains | 1.295 | 0.00808 |
| *EGFR* | Epidermal growth factor receptor | 1.611 | 0.0072 |
| *EGLN3* | Egl-9 family hypoxia-inducible factor 3 | 1.512 | 1.53E-05 |
| *EGR1* | Early growth response 1 | 1.861 | 0.00402 |
| *EHMT1* | Euchromatic histone-lysine N-methyltransferase 1 | 1.309 | 0.00287 |
| *EIF2AK2* | Eukaryotic translation initiation factor 2-alpha kinase 2 | -1.443 | 0.00768 |
| *ELMSAN1* | ELM2 and Myb/SANT-like domain containing 1 | 1.292 | 0.00613 |
| *ELN* | Elastin | 1.815 | 0.00357 |
| *EMILIN2* | Elastin microfibril interfacer 2 | 1.989 | 0.00043 |
| *EPRS* | Glutamyl-prolyl-tRNA synthetase | -1.288 | 0.00111 |
| *ERC1* | ELKS/RAB6-interacting/CAST family member 1 | 1.338 | 0.00077 |
| *ETFA* | Electron-transfer-flavoprotein, alpha polypeptide | -1.38 | 3.09E-05 |
| *ETHE1* | Ethylmalonic encephalopathy 1 | -1.267 | 0.00388 |
| *ETS2* | V-ets avian erythroblastosis virus E26 oncogene homolog 2 | 1.743 | 0.00015 |
| *EXTL1* | Exostosin-like glycosyltransferase 1 | -1.609 | 0.00072 |
| *FABP3* | Fatty acid binding protein 3, muscle and heart (mammary-derived growth inhibitor) | -1.307 | 0.00718 |
| *FADS1* | Fatty acid desaturase 1 | -1.625 | 0.00028 |
| *FADS2* | Fatty acid desaturase 2 | -1.758 | 2.52E-07 |
| *FAHD1* | Fumarylacetoacetate hydrolase domain containing 1 | -1.27 | 0.00207 |
| *FAM173A* | Family with sequence similarity 173, member A | 1.429 | 0.00852 |
| *FAM184B* | Family with sequence similarity 184, member B | 2.085 | 1.28E-09 |
| *FAM189A2* | Family with sequence similarity 189, member A2 | 1.465 | 0.00359 |
| *FAM193B* | Family with sequence similarity 193, member B | 1.353 | 0.00525 |
| *FAM210A* | Family with sequence similarity 210, member A | -1.368 | 0.00022 |
| *FAM78A* | Family with sequence similarity 78, member A | 1.422 | 0.00261 |
| *FAR2* | Fatty acyl CoA reductase 2 | -1.878 | 0.00118 |
| *FASN* | Fatty acid synthase | 1.911 | 0.00713 |
| *FASTKD3* | FAST kinase domains 3 | -1.296 | 0.00918 |
| *FBXO32* | F-box protein 32 | 2.08 | 1.61E-06 |
| *FBXW7* | F-box and WD repeat domain containing 7, E3 ubiquitin protein ligase | -1.29 | 0.00328 |
| *FCGRT* | Fc fragment of IgG, receptor, transporter, alpha | -1.831 | 5.39E-10 |
| *FDFT1* | Farnesyl-diphosphate farnesyltransferase 1 | 1.367 | 0.0077 |
| *FGFR4* | Fibroblast growth factor receptor 4 | 1.458 | 0.00289 |
| *FH* | Fumarate hydratase | -1.322 | 0.00083 |
| *FHOD1* | Formin homology 2 domain containing 1 | 1.46 | 9.42E-05 |
| *FKBP1A* | FK506 binding protein 1A, 12kDa | -1.283 | 0.00088 |
| *FKBP3* | FK506 binding protein 3, 25kDa | -1.261 | 0.00231 |
| *FKBP5* | FK506 binding protein 5 | -1.39 | 0.0073 |
| *FNBP4* | Formin binding protein 4 | 1.363 | 0.00044 |
| *FOSB* | FBJ murine osteosarcoma viral oncogene homolog B | 2.683 | 0.00605 |
| *FOSL2* | FOS-like antigen 2 | 1.992 | 0.00691 |
| *FOXO1* | Forkhead box O1 | 2.008 | 0.00036 |
| *FOXO3* | Forkhead box O3 | 1.385 | 0.00854 |
| *FOXO4* | Forkhead box O4 | 1.391 | 0.00023 |
| *FRMD4A* | FERM domain containing 4A | 1.338 | 0.00045 |
| *FST* | Follistatin | 2.477 | 0.00351 |
| *FUBP3* | Far upstream element (FUSE) binding protein 3 | 1.266 | 0.00472 |
| *G6PD* | Glucose-6-phosphate dehydrogenase | 1.735 | 0.00185 |
| *GAB2* | GRB2-associated binding protein 2 | 1.484 | 0.00193 |
| *GABBR1* | Gamma-aminobutyric acid (GABA) B receptor, 1 | 1.363 | 0.0049 |
| *GADD45A* | Growth arrest and DNA-damage-inducible, alpha | 1.699 | 0.00269 |
| *GADD45B* | Growth arrest and DNA-damage-inducible, beta | 1.527 | 0.00264 |
| *GARS* | Glycyl-tRNA synthetase | -1.9 | 3.22E-07 |
| *GATA2* | GATA binding protein 2 | 1.613 | 0.00112 |
| *GATM* | Glycine amidinotransferase (L-arginine:glycine amidinotransferase) | -1.459 | 0.00297 |
| *GATSL2* | GATS protein-like 2 | 1.581 | 0.00092 |
| *GBAS* | Glioblastoma amplified sequence | -1.276 | 0.00309 |
| *GDPD5* | Glycerophosphodiester phosphodiesterase domain containing 5 | 1.696 | 0.00134 |
| *GEMIN2* | Gem (nuclear organelle) associated protein 2 | -1.308 | 0.00893 |
| *GFM1* | G elongation factor, mitochondrial 1 | -1.336 | 0.00141 |
| *GFRA1* | GDNF family receptor alpha 1 | -1.459 | 0.00043 |
| *GGCT* | Gamma-glutamylcyclotransferase | -1.515 | 0.00447 |
| *GHITM* | Growth hormone inducible transmembrane protein | -1.292 | 0.00346 |
| *GIGYF1* | GRB10 interacting GYF protein 1 | 1.355 | 0.00053 |
| *GIT1* | G protein-coupled receptor kinase interacting ArfGAP 1 | 1.316 | 0.00603 |
| *GMEB2* | Glucocorticoid modulatory element binding protein 2 | 1.302 | 0.00896 |
| *GOT1* | Glutamic-oxaloacetic transaminase 1, soluble | -1.284 | 0.00843 |
| *GPATCH8* | G patch domain containing 8 | 1.449 | 3.26E-05 |
| *GPR146* | G protein-coupled receptor 146 | 1.463 | 0.00054 |
| *GPX8* | Glutathione peroxidase 8 (putative) | -1.49 | 0.0072 |
| *GRB10* | Growth factor receptor-bound protein 10 | 1.456 | 9.04E-05 |
| *GRK4* | G protein-coupled receptor kinase 4 | 1.67 | 6.03E-05 |
| *GRN* | Granulin | 1.394 | 0.00219 |
| *GSE1* | Gse1 coiled-coil protein | 1.57 | 0.00029 |
| *GSTZ1* | Glutathione S-transferase zeta 1 | -1.383 | 0.00304 |
| *GTF2H3* | General transcription factor IIH, polypeptide 3, 34kDa | -1.338 | 0.00725 |
| *H2AFZ* | H2A histone family, member Z | -1.444 | 0.0018 |
| *HADHA* | Hydroxyacyl-CoA dehydrogenase/3-ketoacyl-CoA thiolase/enoyl-CoA hydratase, alpha subunit | -1.263 | 0.00235 |
| *HAUS5* | HAUS augmin-like complex, subunit 5 | 1.317 | 0.00543 |
| *HBB* | Hemoglobin, beta | -8.123 | 6.94E-05 |
| *HDAC4* | Histone deacetylase 4 | 1.862 | 5.92E-07 |
| *HECTD3* | HECT domain containing E3 ubiquitin protein ligase 3 | 1.266 | 0.00469 |
| *HECTD4* | HECT domain containing E3 ubiquitin protein ligase 4 | 1.272 | 0.00905 |
| *HID1* | HID1 domain containing | 1.498 | 0.00227 |
| *Hils1* | Histone H1-like protein in spermatids 1 | 1.567 | 0.00051 |
| *HINT1* | Histidine triad nucleotide binding protein 1 | -1.252 | 0.00209 |
| *HINT2* | Histidine triad nucleotide binding protein 2 | -1.541 | 0.00103 |
| *HIP1* | Huntingtin interacting protein 1 | 1.487 | 0.00166 |
| *HIP1R* | Huntingtin interacting protein 1 related | 1.487 | 0.00066 |
| *HMCN2* | Hemicentin 2 | 1.369 | 0.00421 |
| *HMGCR* | 3-hydroxy-3-methylglutaryl-CoA reductase | -1.642 | 0.00043 |
| *HMGCS1* | 3-hydroxy-3-methylglutaryl-CoA synthase 1 (soluble) | -1.43 | 0.00165 |
| *HOMER3* | Homer homolog 3 (Drosophila) | 1.396 | 0.00106 |
| *HPCAL4* | Hippocalcin like 4 | -2.606 | 3.92E-10 |
| *HSF4* | Heat shock transcription factor 4 | 1.431 | 0.00218 |
| *HSPA5* | Heat shock 70kDa protein 5 (glucose-regulated protein, 78kDa) | -1.43 | 6.29E-05 |
| *HSPA8* | Heat shock 70kDa protein 8 | -1.306 | 0.00508 |
| *HSPA9* | Heat shock 70kDa protein 9 (mortalin) | -1.382 | 3.60E-05 |
| *HSPB6* | Heat shock protein, alpha-crystallin-related, B6 | 1.425 | 0.00054 |
| *HSPD1* | Heat shock 60kDa protein 1 (chaperonin) | -1.388 | 0.0007 |
| *HTRA1* | HtrA serine peptidase 1 | 1.376 | 0.00269 |
| *IARS* | Isoleucyl-tRNA synthetase | -1.444 | 0.00177 |
| *IDH3A* | Isocitrate dehydrogenase 3 (NAD+) alpha | -1.28 | 0.00258 |
| *IFRD1* | Interferon-related developmental regulator 1 | -1.614 | 5.14E-05 |
| *IFT122* | Intraflagellar transport 122 homolog (Chlamydomonas) | 1.492 | 0.003 |
| *IFT140* | Intraflagellar transport 140 homolog (Chlamydomonas) | 1.738 | 0.00049 |
| *IFT27* | Intraflagellar transport 27 homolog (Chlamydomonas) | -1.49 | 0.00498 |
| *IGHMBP2* | Immunoglobulin mu binding protein 2 | 1.413 | 0.00084 |
| *IL17RA* | Interleukin 17 receptor A | 1.549 | 0.0002 |
| *IMPA2* | Inositol(myo)-1(or 4)-monophosphatase 2 | 1.807 | 0.00267 |
| *INPPL1* | Inositol polyphosphate phosphatase-like 1 | 1.306 | 0.00188 |
| *IPP* | Intracisternal A particle-promoted polypeptide | -1.294 | 0.00235 |
| *IRAK1* | Interleukin-1 receptor-associated kinase 1 | 1.35 | 0.00271 |
| *IRF5* | Interferon regulatory factor 5 | 1.811 | 0.0057 |
| *IRF9* | Interferon regulatory factor 9 | 1.32 | 0.00749 |
| *ITGA2B* | Integrin, alpha 2b (platelet glycoprotein IIb of IIb/IIIa complex, antigen CD41) | 1.493 | 0.00044 |
| *ITGB5* | Integrin, beta 5 | 1.46 | 9.33E-06 |
| *ITIH4* | Inter-alpha-trypsin inhibitor heavy chain family, member 4 | 1.481 | 0.00109 |
| *ITPRIP* | Inositol 1,4,5-trisphosphate receptor interacting protein | 1.276 | 0.00917 |
| *IYD* | Iodotyrosine deiodinase | -2.667 | 0.00015 |
| *JADE2* | Jade family PHD finger 2 | 1.957 | 7.91E-06 |
| *JUNB* | Jun B proto-oncogene | 2.041 | 0.00065 |
| *KANK2* | KN motif and ankyrin repeat domains 2 | 1.527 | 6.71E-05 |
| *KAT2B* | K(lysine) acetyltransferase 2B | 1.763 | 5.42E-09 |
| *KBTBD8* | Kelch repeat and BTB (POZ) domain containing 8 | -1.615 | 0.00421 |
| *KCNMA1* | Potassium large conductance calcium-activated channel, subfamily M, alpha member 1 | -1.405 | 0.00141 |
| *KCNMB1* | Potassium large conductance calcium-activated channel, subfamily M, beta member 1 | 1.628 | 0.00666 |
| *KCP* | Kielin/chordin-like protein | 1.469 | 0.00329 |
| *KDM2B* | Lysine (K)-specific demethylase 2B | 1.3 | 0.00366 |
| *KDM4B* | Lysine (K)-specific demethylase 4B | 1.361 | 0.00037 |
| *KDM5C* | Lysine (K)-specific demethylase 5C | 1.26 | 0.00362 |
| *KDM6B* | Lysine (K)-specific demethylase 6B | 1.394 | 0.00069 |
| *KDR* | Kinase insert domain receptor (a type III receptor tyrosine kinase) | -1.349 | 0.00276 |
| *KIAA0754* | KIAA0754 | -1.629 | 0.00023 |
| *KIAA0922* | KIAA0922 | 1.266 | 0.00886 |
| *KLF15* | Kruppel-like factor 15 | 1.533 | 0.00121 |
| *KLF4* | Kruppel-like factor 4 (gut) | 1.545 | 0.00047 |
| *KLF6* | Kruppel-like factor 6 | 1.337 | 0.0015 |
| *KLHL22* | Kelch-like family member 22 | 1.426 | 0.0054 |
| *KLHL25* | Kelch-like family member 25 | 1.569 | 0.0057 |
| *KLHL26* | Kelch-like family member 26 | 1.293 | 0.00557 |
| *KLHL38* | Kelch-like family member 38 | 1.688 | 0.00029 |
| *KMT2B* | Lysine (K)-specific methyltransferase 2B | 1.276 | 0.00814 |
| *L2HGDH* | L-2-hydroxyglutarate dehydrogenase | -1.49 | 0.00627 |
| *LAMA3* | Laminin, alpha 3 | 1.906 | 0.00037 |
| *LAMA5* | Laminin, alpha 5 | 1.29 | 0.0015 |
| *LAMB1* | Laminin, beta 1 | -1.584 | 0.0002 |
| *LDLR* | Low density lipoprotein receptor | -1.973 | 0.0002 |
| *LENG8* | Leukocyte receptor cluster (LRC) member 8 | 1.458 | 0.00144 |
| *LIMD1* | LIM domains containing 1 | 1.357 | 0.00336 |
| *LIN37* | Lin-37 homolog (C. elegans) | 1.334 | 0.00618 |
| *LMNB1* | Lamin B1 | -2.17 | 4.16E-06 |
| *LMOD3* | Leiomodin 3 (fetal) | 1.37 | 0.00088 |
| *LNX1* | Ligand of numb-protein X 1, E3 ubiquitin protein ligase | 1.988 | 0.00193 |
| *LRCH4* | Leucine-rich repeats and calponin homology (CH) domain containing 4 | 1.379 | 0.00203 |
| *LRP1* | Low density lipoprotein receptor-related protein 1 | 1.394 | 0.00106 |
| *LRWD1* | Leucine-rich repeats and WD repeat domain containing 1 | 1.421 | 0.00072 |
| *LTBP2* | Latent transforming growth factor beta binding protein 2 | 1.755 | 0.003 |
| *LTBP3* | Latent transforming growth factor beta binding protein 3 | 1.607 | 0.00053 |
| *LZTS3* | Leucine zipper, putative tumor suppressor family member 3 | 1.448 | 0.00295 |
| *MAFF* | V-maf avian musculoaponeurotic fibrosarcoma oncogene homolog F | 1.469 | 0.00165 |
| *MAFK* | V-maf avian musculoaponeurotic fibrosarcoma oncogene homolog K | 1.598 | 0.00433 |
| *MAK16* | MAK16 homolog (S. cerevisiae) | -1.332 | 0.00014 |
| *MAN2B1* | Mannosidase, alpha, class 2B, member 1 | 1.446 | 2.11E-05 |
| *MANF* | Mesencephalic astrocyte-derived neurotrophic factor | -1.42 | 3.79E-06 |
| *MAOA* | Monoamine oxidase A | -1.57 | 0.00282 |
| *MAOB* | Monoamine oxidase B | 1.593 | 0.0012 |
| *MAP2K7* | Mitogen-activated protein kinase kinase 7 | 1.284 | 0.00606 |
| *MAP4K2* | Mitogen-activated protein kinase kinase kinase kinase 2 | 1.416 | 0.003 |
| *MAPK8IP3* | Mitogen-activated protein kinase 8 interacting protein 3 | 1.374 | 0.0073 |
| *Mar-02* | Mitochondrial amidoxime reducing component 2 | 1.33 | 0.00173 |
| *Mar-08* | Membrane-associated ring finger (C3HC4) 8, E3 ubiquitin protein ligase | 1.3 | 0.0067 |
| *MB* | Myoglobin | 1.409 | 0.00075 |
| *MBOAT7* | Membrane bound O-acyltransferase domain containing 7 | 1.378 | 0.00853 |
| *MCCC1* | Methylcrotonoyl-CoA carboxylase 1 (alpha) | 1.401 | 0.00056 |
| *MCM7* | Minichromosome maintenance complex component 7 | 1.269 | 0.009 |
| *MCTS1* | Malignant T cell amplified sequence 1 | -1.628 | 6.85E-05 |
| *MDH1* | Malate dehydrogenase 1, NAD (soluble) | -1.495 | 0.00553 |
| *ME3* | Malic enzyme 3, NADP(+)-dependent, mitochondrial | 1.769 | 0.00209 |
| *MED12* | Mediator complex subunit 12 | 1.295 | 0.00154 |
| *MED25* | Mediator complex subunit 25 | 1.392 | 0.00072 |
| *METTL1* | Methyltransferase like 1 | -1.464 | 0.00039 |
| *METTL15* | Methyltransferase like 15 | -1.375 | 0.00068 |
| *MFAP4* | Microfibrillar-associated protein 4 | 1.845 | 0.00025 |
| *MFSD4* | Major facilitator superfamily domain containing 4 | 1.874 | 0.00211 |
| *MGLL* | Monoglyceride lipase | 1.35 | 0.00774 |
| *MINK1* | Misshapen-like kinase 1 | 1.3 | 0.00715 |
| *MKI67* | Marker of proliferation Ki-67 | -3.212 | 7.77E-06 |
| *MLLT6* | Myeloid/lymphoid or mixed-lineage leukemia (trithorax homolog, Drosophila); translocated to, 6 | 1.645 | 5.65E-06 |
| *MLXIP* | MLX interacting protein | 1.457 | 0.0043 |
| *MLXIPL* | MLX interacting protein-like | 1.648 | 0.0069 |
| *MMACHC* | Methylmalonic aciduria (cobalamin deficiency) cblC type, with homocystinuria | -1.25 | 0.00839 |
| *MME* | Membrane metallo-endopeptidase | -1.525 | 0.00094 |
| *MPC2* | Mitochondrial pyruvate carrier 2 | -1.316 | 0.0003 |
| *MPHOSPH6* | M-phase phosphoprotein 6 | -1.541 | 6.03E-05 |
| *MROH1* | Maestro heat-like repeat family member 1 | 1.43 | 0.00062 |
| *MRPL1* | Mitochondrial ribosomal protein L1 | -1.294 | 0.00278 |
| *MRPL15* | Mitochondrial ribosomal protein L15 | -1.41 | 1.34E-05 |
| *MRPL42* | Mitochondrial ribosomal protein L42 | -1.251 | 0.0056 |
| *MRPL48* | Mitochondrial ribosomal protein L48 | -1.284 | 0.00306 |
| *MRPS28* | Mitochondrial ribosomal protein S28 | -1.26 | 0.00313 |
| *MRPS31* | Mitochondrial ribosomal protein S31 | -1.317 | 0.00114 |
| *MRPS33* | Mitochondrial ribosomal protein S33 | -1.394 | 0.00247 |
| *MRPS36* | Mitochondrial ribosomal protein S36 | -1.332 | 0.00074 |
| *MSH2* | MutS homolog 2 | -1.429 | 0.00056 |
| *MSMO1* | Methylsterol monooxygenase 1 | -1.971 | 2.33E-06 |
| *MT-CO2* | Cytochrome c oxidase subunit II | -1.357 | 0.00315 |
| *MTHFD1L* | Methylenetetrahydrofolate dehydrogenase (NADP+ dependent) 1-like | -1.845 | 0.0045 |
| *MTHFR* | Methylenetetrahydrofolate reductase (NAD(P)H) | 1.313 | 0.00405 |
| *MTSS1L* | Metastasis suppressor 1-like | 1.913 | 2.97E-05 |
| *MUTYH* | MutY homolog | 1.482 | 0.00416 |
| *MXD1* | MAX dimerization protein 1 | 1.389 | 0.00093 |
| *MXRA8* | Matrix-remodelling associated 8 | 1.757 | 0.00387 |
| *MYF5* | Myogenic factor 5 | 1.503 | 0.00905 |
| *MYH11* | Myosin, heavy chain 11, smooth muscle | 1.9 | 9.07E-05 |
| *MYH14* | Myosin, heavy chain 14, non-muscle | 1.477 | 0.00049 |
| *MYL1* | Myosin, light chain 1, alkali; skeletal, fast | -1.329 | 0.00139 |
| *MYL12A* | Myosin, light chain 12A, regulatory, non-sarcomeric | -1.313 | 0.00261 |
| *MYLIP* | Myosin regulatory light chain interacting protein | 1.28 | 0.00729 |
| *MYO10* | Myosin X | 1.698 | 0.004 |
| *MYOT* | Myotilin | -1.283 | 0.00305 |
| *MZF1* | Myeloid zinc finger 1 | 1.41 | 0.0031 |
| *NADSYN1* | NAD synthetase 1 | 1.457 | 0.001 |
| *NAPRT1* | Nicotinate phosphoribosyltransferase domain containing 1 | 1.702 | 1.14E-06 |
| *NAV2* | Neuron navigator 2 | -1.754 | 0.00228 |
| *NCBP2* | Nuclear cap binding protein subunit 2, 20kDa | -1.292 | 0.00197 |
| *NDRG1* | N-myc downstream regulated 1 | 1.399 | 0.00537 |
| *NDRG2* | NDRG family member 2 | 1.314 | 0.00835 |
| *NDRG4* | NDRG family member 4 | 1.564 | 0.0079 |
| *NDUFA12* | NADH dehydrogenase (ubiquinone) 1 alpha subcomplex, 12 | -1.539 | 2.82E-06 |
| *NDUFA4* | NADH dehydrogenase (ubiquinone) 1 alpha subcomplex, 4, 9kDa | -1.537 | 0.00206 |
| *NDUFA5* | NADH dehydrogenase (ubiquinone) 1 alpha subcomplex, 5 | -1.319 | 0.00034 |
| *NDUFAF1* | NADH dehydrogenase (ubiquinone) complex I, assembly factor 1 | -1.256 | 0.00276 |
| *NDUFAF5* | NADH dehydrogenase (ubiquinone) complex I, assembly factor 5 | -1.359 | 0.00458 |
| *NDUFAF6* | NADH dehydrogenase (ubiquinone) complex I, assembly factor 6 | -1.258 | 0.00605 |
| *NDUFB3* | NADH dehydrogenase (ubiquinone) 1 beta subcomplex, 3, 12kDa | -1.271 | 0.0052 |
| *NDUFB5* | NADH dehydrogenase (ubiquinone) 1 beta subcomplex, 5, 16kDa | -1.511 | 0.00723 |
| *NDUFB6* | NADH dehydrogenase (ubiquinone) 1 beta subcomplex, 6, 17kDa | -1.337 | 7.32E-05 |
| *NDUFC1* | NADH dehydrogenase (ubiquinone) 1, subcomplex unknown, 1, 6kDa | -1.374 | 0.00575 |
| *NDUFS1* | NADH dehydrogenase (ubiquinone) Fe-S protein 1, 75kDa (NADH-coenzyme Q reductase) | -1.414 | 0.00119 |
| *NDUFS4* | NADH dehydrogenase (ubiquinone) Fe-S protein 4, 18kDa (NADH-coenzyme Q reductase) | -1.376 | 0.00039 |
| NDUFS5 | NADH dehydrogenase (ubiquinone) Fe-S protein 5, 15kDa (NADH-coenzyme Q reductase) | -1.254 | 0.00604 |
| *NDUFV2* | NADH dehydrogenase (ubiquinone) flavoprotein 2, 24kDa | -1.333 | 0.00152 |
| *NEDD4L* | Neural precursor cell expressed, developmentally down-regulated 4-like, E3 ubiquitin protein ligase | 1.513 | 2.20E-05 |
| *NFATC1* | Nuclear factor of activated T-cells, cytoplasmic, calcineurin-dependent 1 | 1.318 | 0.00624 |
| *NFE2L1* | Nuclear factor, erythroid 2-like 1 | 1.298 | 0.00115 |
| *NFRKB* | Nuclear factor related to kappaB binding protein | 1.373 | 0.00326 |
| *NME3* | NME/NM23 nucleoside diphosphate kinase 3 | 1.315 | 0.0088 |
| *NOB1* | NIN1/RPN12 binding protein 1 homolog (S. cerevisiae) | -1.282 | 0.00612 |
| *NOTCH1* | Notch 1 | 1.29 | 0.00532 |
| *NOTCH3* | Notch 3 | 1.326 | 0.00091 |
| *NPNT* | Nephronectin | 2.188 | 7.79E-15 |
| *NQO1* | NAD(P)H dehydrogenase, quinone 1 | -1.271 | 0.00171 |
| *NR1D1* | Nuclear receptor subfamily 1, group D, member 1 | 1.636 | 0.00237 |
| *NRDE2* | NRDE-2, necessary for RNA interference, domain containing | 1.312 | 0.00159 |
| *NREP* | Neuronal regeneration related protein | -1.395 | 0.00591 |
| *NRIP2* | Nuclear receptor interacting protein 2 | 1.486 | 0.00664 |
| *NSUN4* | NOP2/Sun domain family, member 4 | -1.321 | 0.00087 |
| *NUAK1* | NUAK family, SNF1-like kinase, 1 | 1.405 | 0.0018 |
| *NUMA1* | Nuclear mitotic apparatus protein 1 | 1.463 | 6.61E-06 |
| *NUMBL* | Numb homolog (Drosophila)-like | 1.477 | 0.00598 |
| *NUP43* | Nucleoporin 43kDa | -1.441 | 0.00165 |
| *NUPR1* | Nuclear protein, transcriptional regulator, 1 | 2.536 | 0.00023 |
| *NUTF2* | Nuclear transport factor 2 | -1.275 | 0.00064 |
| *OBSL1* | Obscurin-like 1 | 1.35 | 0.00386 |
| *OCEL1* | Occludin/ELL domain containing 1 | 1.424 | 0.00152 |
| *OLA1* | Obg-like ATPase 1 | -1.309 | 0.00191 |
| *OLFML2A* | Olfactomedin-like 2A | 1.481 | 0.00014 |
| *OS9* | Osteosarcoma amplified 9, endoplasmic reticulum lectin | 1.316 | 0.00016 |
| *OSBP* | Oxysterol binding protein | 1.299 | 0.00343 |
| *OSBP2* | Oxysterol binding protein 2 | 1.961 | 5.54E-05 |
| *OSBPL5* | Oxysterol binding protein-like 5 | 1.493 | 0.00079 |
| *OSTC* | Oligosaccharyltransferase complex subunit (non-catalytic) | -1.266 | 0.00427 |
| *OTUD3* | OTU domain containing 3 | 1.396 | 0.00079 |
| *PACS1* | Phosphofurin acidic cluster sorting protein 1 | 1.29 | 0.00209 |
| *PARK2* | Parkin RBR E3 ubiquitin protein ligase | 1.416 | 0.00511 |
| *PARM1* | Prostate androgen-regulated mucin-like protein 1 | 2.437 | 7.65E-10 |
| *PBXIP1* | Pre-B-cell leukemia homeobox interacting protein 1 | 1.343 | 0.00039 |
| *PCBD1* | Pterin-4 alpha-carbinolamine dehydratase/dimerization cofactor of hepatocyte nuclear factor 1 alpha | -4.002 | 3.63E-05 |
| *PCCB* | Propionyl CoA carboxylase, beta polypeptide | -1.292 | 0.0026 |
| *PCDH12* | Protocadherin 12 | -1.756 | 0.00075 |
| *PCED1A* | PC-esterase domain containing 1A | 1.362 | 0.00382 |
| *PCNP* | PEST proteolytic signal containing nuclear protein | -1.322 | 0.00667 |
| *PCNT* | Pericentrin | 1.295 | 0.00169 |
| *PDCL3* | Phosducin-like 3 | -1.336 | 0.00097 |
| *PDHA1* | Pyruvate dehydrogenase (lipoamide) alpha 1 | -1.322 | 0.00121 |
| *PDHB* | Pyruvate dehydrogenase (lipoamide) beta | -1.283 | 0.00091 |
| *Pdlim3* | PDZ and LIM domain 3 | 1.415 | 0.00019 |
| *PDSS1* | Prenyl (decaprenyl) diphosphate synthase, subunit 1 | -1.397 | 0.00062 |
| *PDSS2* | Prenyl (decaprenyl) diphosphate synthase, subunit 2 | -1.312 | 0.0084 |
| *PDZRN3* | PDZ domain containing ring finger 3 | 1.427 | 0.00034 |
| *PECR* | Peroxisomal trans-2-enoyl-CoA reductase | -1.37 | 2.99E-05 |
| *PELI3* | Pellino E3 ubiquitin protein ligase family member 3 | 1.817 | 0.00015 |
| *PER2* | Period circadian clock 2 | 1.456 | 0.00093 |
| *Pfn2* | Profilin 2 | -1.347 | 0.0022 |
| *PGGT1B* | Protein geranylgeranyltransferase type I, beta subunit | -1.27 | 0.00726 |
| *PGK1* | Phosphoglycerate kinase 1 | -1.351 | 0.00227 |
| *PGPEP1L* | Pyroglutamyl-peptidase I-like | 1.818 | 0.00119 |
| *PGRMC1* | Progesterone receptor membrane component 1 | -1.635 | 0.00064 |
| *PHC1* | Polyhomeotic homolog 1 (Drosophila) | 1.386 | 0.00564 |
| *PHGDH* | Phosphoglycerate dehydrogenase | -8.936 | 6.78E-10 |
| *PHLDB1* | Pleckstrin homology-like domain, family B, member 1 | 1.407 | 0.00127 |
| *PHLPP1* | PH domain and leucine rich repeat protein phosphatase 1 | 1.26 | 0.00481 |
| *PI4KA* | Phosphatidylinositol 4-kinase, catalytic, alpha | 1.272 | 0.00509 |
| *PIAS3* | Protein inhibitor of activated STAT, 3 | 1.336 | 0.00067 |
| *PIGH* | Phosphatidylinositol glycan anchor biosynthesis, class H | -1.437 | 0.00075 |
| *PIK3IP1* | Phosphoinositide-3-kinase interacting protein 1 | 1.945 | 2.20E-07 |
| *PIM3* | Pim-3 oncogene | 1.365 | 0.00846 |
| *PIP5K1C* | Phosphatidylinositol-4-phosphate 5-kinase, type I, gamma | 1.351 | 0.00316 |
| *PITPNM1* | Phosphatidylinositol transfer protein, membrane-associated 1 | 1.425 | 0.00161 |
| *PKD1* | Polycystic kidney disease 1 (autosomal dominant) | 1.293 | 0.00648 |
| *PLA2G6* | Phospholipase A2, group VI (cytosolic, calcium-independent) | 1.336 | 0.00182 |
| *PLA2R1* | Phospholipase A2 receptor 1, 180kDa | 1.678 | 0.00585 |
| *PLCB3* | Phospholipase C, beta 3 (phosphatidylinositol-specific) | 1.392 | 6.18E-05 |
| *PLEKHM1* | Pleckstrin homology domain containing, family M (with RUN domain) member 1 | 1.376 | 0.00094 |
| *PLIN2* | Perilipin 2 | 1.731 | 0.00037 |
| *PLXDC2* | Plexin domain containing 2 | 2.537 | 0.00323 |
| *PLXNB1* | Plexin B1 | 1.592 | 0.00452 |
| *PMEPA1* | Prostate transmembrane protein, androgen induced 1 | 1.711 | 0.00035 |
| *PMP22* | Peripheral myelin protein 22 | -1.361 | 0.00036 |
| *PNP* | Purine nucleoside phosphorylase | -1.301 | 0.00203 |
| *PNPLA2* | Patatin-like phospholipase domain containing 2 | 1.394 | 0.00264 |
| *PNPO* | Pyridoxamine 5'-phosphate oxidase | -1.286 | 0.00315 |
| *POLD4* | Polymerase (DNA-directed), delta 4, accessory subunit | 1.308 | 0.00437 |
| *POLR2G* | Polymerase (RNA) II (DNA directed) polypeptide G | -1.255 | 0.00587 |
| *POLR2I* | Polymerase (RNA) II (DNA directed) polypeptide I, 14.5kDa | 1.301 | 0.00603 |
| *POLRMT* | Polymerase (RNA) mitochondrial (DNA directed) | 1.344 | 0.0034 |
| *PON1* | Paraoxonase 1 | 2.292 | 0.00672 |
| *PPA1* | Pyrophosphatase (inorganic) 1 | -1.368 | 0.00169 |
| *PPA2* | Pyrophosphatase (inorganic) 2 | -1.508 | 0.00225 |
| *PPID* | Peptidylprolyl isomerase D | -1.278 | 0.00169 |
| *PPIF* | Peptidylprolyl isomerase F | -1.387 | 0.00209 |
| *PPP1R13L* | Protein phosphatase 1, regulatory subunit 13 like | 1.482 | 0.00307 |
| *PQLC3* | PQ loop repeat containing 3 | -1.666 | 0.00767 |
| *PRKAG3* | Protein kinase, AMP-activated, gamma 3 non-catalytic subunit | 1.35 | 0.00698 |
| *PRODH* | Proline dehydrogenase (oxidase) 1 | 1.744 | 0.00139 |
| *PRSS23* | Protease, serine, 23 | 1.791 | 8.18E-06 |
| *PSAP* | Prosaposin | 1.262 | 0.00445 |
| *PSAT1* | Phosphoserine aminotransferase 1 | -3.804 | 2.87E-08 |
| *PSMG2* | Proteasome (prosome, macropain) assembly chaperone 2 | -1.455 | 0.00713 |
| *PSPH* | Phosphoserine phosphatase | -4.508 | 6.60E-08 |
| *PSTK* | Phosphoseryl-tRNA kinase | -1.392 | 0.00391 |
| *PTGIS* | Prostaglandin I2 (prostacyclin) synthase | 1.91 | 0.0059 |
| *PTPLA* | Protein tyrosine phosphatase-like (proline instead of catalytic arginine), member A | -1.375 | 0.00023 |
| *PTPN23* | Protein tyrosine phosphatase, non-receptor type 23 | 1.358 | 0.00315 |
| *PTPRS* | Protein tyrosine phosphatase, receptor type, S | 1.446 | 2.91E-05 |
| *PTPRU* | Protein tyrosine phosphatase, receptor type, U | 1.584 | 0.00045 |
| *PXMP2* | Peroxisomal membrane protein 2, 22kDa | 1.444 | 0.00204 |
| *PYCR1* | Pyrroline-5-carboxylate reductase 1 | -2.504 | 6.22E-05 |
| *RABGGTB* | Rab geranylgeranyltransferase, beta subunit | -1.352 | 0.0004 |
| *RAD1* | RAD1 homolog (S. pombe) | -1.516 | 0.00486 |
| *RAET1L* | Retinoic acid early transcript 1L | -1.737 | 0.00167 |
| *RALA* | V-ral simian leukemia viral oncogene homolog A (ras related) | -1.386 | 0.00038 |
| *RASA3* | RAS p21 protein activator 3 | 1.297 | 0.00301 |
| *RASA4* | RAS p21 protein activator 4 | 1.44 | 0.00048 |
| *RASD1* | RAS, dexamethasone-induced 1 | 1.461 | 0.00341 |
| *RASSF4* | Ras association (RalGDS/AF-6) domain family member 4 | -2.28 | 0.0006 |
| *RBCK1* | RanBP-type and C3HC4-type zinc finger containing 1 | 1.321 | 0.00232 |
| *RBM33* | RNA binding motif protein 33 | 1.324 | 0.0007 |
| *RBM38* | RNA binding motif protein 38 | 1.728 | 0.00012 |
| *Rcan1* | Regulator of calcineurin 1 | -1.772 | 0.00119 |
| *RCC1* | Regulator of chromosome condensation 1 | 1.28 | 0.00901 |
| *RDH11* | Retinol dehydrogenase 11 (all-trans/9-cis/11-cis) | -1.418 | 0.00907 |
| *RELL1* | RELT-like 1 | 1.458 | 0.00321 |
| *RERE* | Arginine-glutamic acid dipeptide (RE) repeats | 1.522 | 0.00035 |
| *RFXANK* | Regulatory factor X-associated ankyrin-containing protein | 1.479 | 0.00099 |
| *RGS14* | Regulator of G-protein signaling 14 | 1.554 | 0.00039 |
| *RGS3* | Regulator of G-protein signaling 3 | -1.315 | 0.00434 |
| *RHOD* | Ras homolog family member D | -1.647 | 0.00648 |
| *RNF123* | Ring finger protein 123 | 1.438 | 0.00012 |
| *Rpl10* | Ribosomal protein L10 | -1.289 | 0.00258 |
| *RPL21* | Ribosomal protein L21 | -1.443 | 0.00082 |
| *RPL3* | Ribosomal protein L3 | -1.621 | 2.45E-05 |
| *RPL5* | Ribosomal protein L5 | -1.396 | 0.00147 |
| *RPL7* | Ribosomal protein L7 | -1.327 | 0.0028 |
| *RPS12* | Ribosomal protein S12 | -1.291 | 0.00174 |
| *RPS15A* | Ribosomal protein S15a | -1.493 | 0.00058 |
| *RPS24* | Ribosomal protein S24 | -1.337 | 0.00469 |
| *RPS27L* | Ribosomal protein S27-like | -1.362 | 0.00545 |
| *RPS3A* | Ribosomal protein S3A | -1.394 | 0.00481 |
| *RREB1* | Ras responsive element binding protein 1 | 1.438 | 0.00025 |
| *RRM2* | Ribonucleotide reductase M2 | -2.956 | 1.92E-05 |
| *RSPO3* | R-spondin 3 | -2.105 | 0.00029 |
| *RTN4IP1* | Reticulon 4 interacting protein 1 | -1.408 | 0.00088 |
| *RXRA* | Retinoid X receptor, alpha | 1.483 | 0.00429 |
| *RYR1* | Ryanodine receptor 1 (skeletal) | 1.419 | 0.00266 |
| *SARS* | Seryl-tRNA synthetase | -1.982 | 1.11E-05 |
| *SART3* | Squamous cell carcinoma antigen recognized by T cells 3 | 1.267 | 0.00414 |
| *SBNO2* | Strawberry notch homolog 2 (Drosophila) | 1.535 | 6.46E-05 |
| *SCRN3* | Secernin 3 | -1.609 | 0.00038 |
| *SDHD* | Succinate dehydrogenase [ubiquinone] cytochrome b small subunit, mitochondrial | -1.27 | 0.00496 |
| *SDK2* | Sidekick cell adhesion molecule 2 | 1.721 | 0.001 |
| *SEC16A* | SEC16 homolog A (S. cerevisiae) | 1.277 | 0.00072 |
| *SELM* | Selenoprotein M | 1.801 | 7.68E-07 |
| *SELT* | Selenoprotein T | -1.372 | 0.00344 |
| *Sep-05* | Septin 5 | 1.714 | 0.00358 |
| *SERINC5* | Serine incorporator 5 | 1.549 | 0.00135 |
| *SETD7* | SET domain containing (lysine methyltransferase) 7 | 1.484 | 0.00792 |
| *SFRP2* | Secreted frizzled-related protein 2 | 7.809 | 3.68E-05 |
| *SFRP4* | Secreted frizzled-related protein 4 | 3.251 | 0.00745 |
| *SH3BP5* | SH3-domain binding protein 5 (BTK-associated) | 1.412 | 0.00632 |
| *SH3PXD2A* | SH3 and PX domains 2A | 1.313 | 0.00317 |
| *SIPA1L2* | Signal-induced proliferation-associated 1 like 2 | 1.421 | 0.00576 |
| *SIX5* | SIX homeobox 5 | 1.765 | 0.00043 |
| *SKA2* | Spindle and kinetochore associated complex subunit 2 | -1.36 | 0.0003 |
| *SKI* | V-ski avian sarcoma viral oncogene homolog | 1.348 | 0.00652 |
| *SKIV2L* | Superkiller viralicidic activity 2-like (S. cerevisiae) | 1.329 | 0.00042 |
| *SLC12A7* | Solute carrier family 12 (potassium/chloride transporter), member 7 | 1.349 | 0.00605 |
| *SLC1A4* | Solute carrier family 1 (glutamate/neutral amino acid transporter), member 4 | -1.373 | 0.00365 |
| *SLC25A12* | Solute carrier family 25 (aspartate/glutamate carrier), member 12 | -1.304 | 0.00543 |
| *SLC25A3* | Solute carrier family 25 (mitochondrial carrier; phosphate carrier), member 3 | -1.372 | 0.00223 |
| *SLC26A11* | Solute carrier family 26 (anion exchanger), member 11 | 1.471 | 8.92E-06 |
| *SLC27A6* | Solute carrier family 27 (fatty acid transporter), member 6 | 2.765 | 6.04E-05 |
| *SLC2A4RG* | SLC2A4 regulator | 1.464 | 0.00304 |
| *SLC31A1* | Solute carrier family 31 (copper transporter), member 1 | -1.358 | 0.00211 |
| *SLC33A1* | Solute carrier family 33 (acetyl-CoA transporter), member 1 | -1.259 | 0.00579 |
| *SLC38A4* | Solute carrier family 38, member 4 | -1.969 | 0.00121 |
| *SLC38A7* | Solute carrier family 38, member 7 | 1.58 | 2.91E-05 |
| *SLC43A2* | Solute carrier family 43 (amino acid system L transporter), member 2 | 1.906 | 0.00052 |
| *SLC7A1* | Solute carrier family 7 (cationic amino acid transporter, y+ system), member 1 | -1.574 | 0.00013 |
| *SLC7A4* | Solute carrier family 7, member 4 | 2.214 | 2.73E-06 |
| *SLC7A8* | Solute carrier family 7 (amino acid transporter light chain, L system), member 8 | 1.738 | 0.00039 |
| *SLMAP* | Sarcolemma associated protein | -1.665 | 4.66E-05 |
| *SMAP2* | Small ArfGAP2 | -1.354 | 0.00405 |
| *SMARCC2* | SWI/SNF related, matrix associated, actin dependent regulator of chromatin, subfamily c, member 2 | 1.369 | 2.35E-05 |
| *SMIM10* | Small integral membrane protein 10 | -1.497 | 0.00625 |
| *SMOC1* | SPARC related modular calcium binding 1 | 1.55 | 0.00192 |
| *SMPDL3A* | Sphingomyelin phosphodiesterase, acid-like 3A | -1.62 | 0.00246 |
| *SMTNL1* | Smoothelin-like 1 | 1.48 | 0.00262 |
| *SNAI2* | Snail family zinc finger 2 | -1.533 | 0.00468 |
| *SNRPD1* | Small nuclear ribonucleoprotein D1 polypeptide 16kDa | -1.377 | 0.00032 |
| *SNRPF* | Small nuclear ribonucleoprotein polypeptide F | -1.389 | 0.00018 |
| *SNX3* | Sorting nexin 3 | -1.356 | 0.00079 |
| *SOCS2* | Suppressor of cytokine signaling 2 | -2.033 | 0.00051 |
| *SOGA2* | SOGA family member 2 | 1.648 | 0.00033 |
| *SORBS1* | Sorbin and SH3 domain containing 1 | 1.464 | 0.00014 |
| *SORL1* | Sortilin-related receptor, L(DLR class) A repeats containing | 1.914 | 0.00016 |
| *SOX13* | SRY (sex determining region Y)-box 13 | 1.477 | 0.00032 |
| *SPCS2* | Signal peptidase complex subunit 2 homolog (S. cerevisiae) | -1.368 | 0.00205 |
| *SPRY4* | Sprouty homolog 4 (Drosophila) | -1.713 | 0.00014 |
| *SPSB1* | SplA/ryanodine receptor domain and SOCS box containing 1 | 1.44 | 0.00075 |
| *SPSB3* | SplA/ryanodine receptor domain and SOCS box containing 3 | 1.435 | 0.00475 |
| *SQLE* | Squalene epoxidase | -3.233 | 8.40E-09 |
| *SREBF1* | Sterol regulatory element binding transcription factor 1 | 1.44 | 0.0011 |
| *SRSF5* | Serine/arginine-rich splicing factor 5 | 1.36 | 0.00182 |
| *SSH3* | Slingshot protein phosphatase 3 | 1.437 | 0.00051 |
| *ST3GAL2* | ST3 beta-galactoside alpha-2,3-sialyltransferase 2 | 1.389 | 0.00226 |
| *ST7* | Suppression of tumorigenicity 7 | -1.304 | 0.00202 |
| *ST8SIA5* | ST8 alpha-N-acetyl-neuraminide alpha-2,8-sialyltransferase 5 | -1.834 | 0.00035 |
| *STAT5A* | Signal transducer and activator of transcription 5A | 1.505 | 4.78E-05 |
| *STAT5B* | Signal transducer and activator of transcription 5B | 1.395 | 0.00185 |
| *STK36* | Serine/threonine kinase 36 | 1.39 | 0.00165 |
| *SUCLA2* | Succinate-CoA ligase, ADP-forming, beta subunit | -1.438 | 0.0003 |
| *SUCLG1* | Succinate-CoA ligase, alpha subunit | -1.274 | 0.00154 |
| *SUCLG2* | Succinate-CoA ligase, GDP-forming, beta subunit | -1.279 | 0.0058 |
| *SULF2* | Sulfatase 2 | 1.387 | 0.00144 |
| *SUPT5H* | Suppressor of Ty 5 homolog (S. cerevisiae) | 1.293 | 0.00052 |
| *SYNM* | Synemin, intermediate filament protein | 1.345 | 0.0062 |
| *SZT2* | Seizure threshold 2 homolog (mouse) | 1.296 | 0.00316 |
| *TAF12* | TAF12 RNA polymerase II, TATA box binding protein (TBP)-associated factor, 20kDa | -1.321 | 7.48E-05 |
| *TAF1C* | TATA box binding protein (TBP)-associated factor, RNA polymerase I, C, 110kDa | 1.418 | 0.00339 |
| *TAGLN* | Transgelin | 1.388 | 0.00288 |
| *TARS* | Threonyl-tRNA synthetase | -1.409 | 0.00011 |
| *TBC1D17* | TBC1 domain family, member 17 | 1.389 | 0.00036 |
| *TBKBP1* | TBK1 binding protein 1 | 1.489 | 0.00479 |
| *TCEA2* | Transcription elongation factor A (SII), 2 | 1.331 | 0.00747 |
| *TCHP* | Trichoplein, keratin filament binding | 1.366 | 0.00264 |
| *TEAD4* | TEA domain family member 4 | 1.434 | 0.00197 |
| *TEF* | Thyrotrophic embryonic factor | 1.292 | 0.00261 |
| *TEFM* | Transcription elongation factor, mitochondrial | -1.381 | 0.00449 |
| *TENC1* | Tensin like C1 domain containing phosphatase (tensin 2) | 1.331 | 0.0021 |
| *TEP1* | Telomerase-associated protein 1 | 1.515 | 6.90E-06 |
| *TFB2M* | Transcription factor B2, mitochondrial | -1.317 | 0.00323 |
| *THBS1* | Thrombospondin 1 | 1.85 | 0.00455 |
| *THBS4* | Thrombospondin 4 | 2.006 | 0.00316 |
| *THRB* | Thyroid hormone receptor, beta | 1.294 | 0.007 |
| *THY1* | Thy-1 cell surface antigen | 2.322 | 0.00408 |
| *TIMM8A* | Translocase of inner mitochondrial membrane 8 homolog A (yeast) | -1.418 | 0.00049 |
| *TM2D1* | TM2 domain containing 1 | -1.253 | 0.00426 |
| *TM4SF18* | Transmembrane 4 L six family member 18 | -2.312 | 0.00052 |
| *TMCO3* | Transmembrane and coiled-coil domains 3 | 1.485 | 0.00167 |
| *TMEM120A* | Transmembrane protein 120A | -1.425 | 0.00066 |
| *TMEM140* | Transmembrane protein 140 | 1.538 | 0.00096 |
| *TMEM145* | Transmembrane protein 145 | 1.454 | 0.00234 |
| *TMEM182* | Transmembrane protein 182 | -1.295 | 0.00201 |
| *TMEM51* | Transmembrane protein 51 | 1.74 | 2.98E-05 |
| *TMEM63B* | Transmembrane protein 63B | 1.278 | 0.00288 |
| *TNFAIP8L1* | Tumor necrosis factor, alpha-induced protein 8-like 1 | -1.474 | 0.00493 |
| *TNFRSF21* | Tumor necrosis factor receptor superfamily, member 21 | 1.874 | 3.65E-06 |
| *TNKS1BP1* | Tankyrase 1 binding protein 1, 182kDa | 1.379 | 0.00089 |
| *TNS1* | Tensin 1 | 1.398 | 0.00135 |
| *TOMM20* | Translocase of outer mitochondrial membrane 20 homolog (yeast) | -1.485 | 0.00036 |
| *TOMM40L* | Translocase of outer mitochondrial membrane 40 homolog (yeast)-like | -1.424 | 7.44E-05 |
| *TP53BP1* | Tumor protein p53 binding protein 1 | 1.286 | 0.00389 |
| *TP53INP1* | Tumor protein p53 inducible nuclear protein 1 | 1.86 | 0.00135 |
| *TP63* | Tumor protein p63 | 1.441 | 0.00653 |
| *TPPP2* | Tubulin polymerization-promoting protein family member 2 | 1.959 | 0.00568 |
| *TRAF3IP1* | TNF receptor-associated factor 3 interacting protein 1 | 1.368 | 0.00117 |
| *TRANK1* | Tetratricopeptide repeat and ankyrin repeat containing 1 | 1.692 | 0.00088 |
| *TRIB1* | Tribbles pseudokinase 1 | -1.364 | 0.00413 |
| *TRIM2* | Tripartite motif containing 2 | -1.472 | 0.00165 |
| *TRIM44* | Tripartite motif containing 44 | 1.566 | 0.00011 |
| *TRIM65* | Tripartite motif containing 65 | 1.33 | 0.00809 |
| *TRRAP* | Transformation/transcription domain-associated protein | 1.267 | 0.00527 |
| *TSC22D3* | TSC22 domain family, member 3 | 1.394 | 0.00242 |
| *TSEN34* | TSEN34 tRNA splicing endonuclease subunit | 1.488 | 0.00393 |
| *TSPAN31* | Tetraspanin 31 | -1.261 | 0.00087 |
| *TSPO* | Translocator protein (18kDa) | 1.445 | 0.00498 |
| *TTC19* | Tetratricopeptide repeat domain 19 | -1.445 | 6.95E-06 |
| *TTC28* | Tetratricopeptide repeat domain 28 | 1.306 | 0.00313 |
| *TTC9* | Tetratricopeptide repeat domain 9 | 1.606 | 4.51E-05 |
| *TTF1* | Transcription termination factor, RNA polymerase I | 1.281 | 0.00903 |
| *TUBA1B* | Tubulin, alpha 1b | -1.461 | 4.08E-06 |
| *TUSC5* | Tumor suppressor candidate 5 | 3.188 | 0.00225 |
| *UBC* | Ubiquitin C | 1.415 | 0.00049 |
| *Ube2n* | Ubiquitin-conjugating enzyme E2N | -1.39 | 0.0006 |
| *UBE4A* | Ubiquitination factor E4A | 1.32 | 0.00153 |
| *UBR4* | Ubiquitin protein ligase E3 component n-recognin 4 | 1.26 | 0.00133 |
| *ULK1* | Unc-51 like autophagy activating kinase 1 | 1.577 | 7.56E-07 |
| *UPF1* | UPF1 regulator of nonsense transcripts homolog (yeast) | 1.27 | 0.00333 |
| *UQCR10* | Ubiquinol-cytochrome c reductase, complex III subunit X | -1.32 | 0.00074 |
| *UQCRB* | Ubiquinol-cytochrome c reductase binding protein | -1.4 | 0.00023 |
| *UQCRC2* | Ubiquinol-cytochrome c reductase core protein II | -1.387 | 0.00311 |
| *UQCRFS1* | Ubiquinol-cytochrome c reductase, Rieske iron-sulfur polypeptide 1 | -1.435 | 0.00023 |
| *USMG5* | Up-regulated during skeletal muscle growth 5 homolog (mouse) | -1.457 | 0.00859 |
| *USP19* | Ubiquitin specific peptidase 19 | 1.485 | 4.53E-05 |
| *USP20* | Ubiquitin specific peptidase 20 | 1.325 | 0.00558 |
| *USP36* | Ubiquitin specific peptidase 36 | 1.298 | 0.00792 |
| *VDAC2* | Voltage-dependent anion channel 2 | -1.269 | 0.00079 |
| *VGLL4* | Vestigial like 4 (Drosophila) | 1.458 | 0.00082 |
| *VIPR1* | Vasoactive intestinal peptide receptor 1 | 1.501 | 0.00037 |
| *VOPP1* | Vesicular, overexpressed in cancer, prosurvival protein 1 | 1.347 | 0.00628 |
| *VWA5A* | Von Willebrand factor A domain containing 5A | 2.829 | 0.00625 |
| *WARS* | Tryptophanyl-tRNA synthetase | -1.599 | 5.06E-05 |
| *WBSCR17* | Williams-Beuren syndrome chromosome region 17 | 1.626 | 0.00035 |
| *WDFY1* | WD repeat and FYVE domain containing 1 | 1.291 | 0.00795 |
| *WDR34* | WD repeat domain 34 | 1.374 | 0.008 |
| *WDR45* | WD repeat domain 45 | 1.319 | 0.0006 |
| *WDR6* | WD repeat domain 6 | 1.263 | 0.00391 |
| *WDR62* | WD repeat domain 62 | 1.689 | 0.00085 |
| *WDR77* | WD repeat domain 77 | -1.253 | 0.00227 |
| *WDR81* | WD repeat domain 81 | 1.317 | 0.00306 |
| *WDR91* | WD repeat domain 91 | 1.463 | 7.04E-05 |
| *WHSC1* | Wolf-Hirschhorn syndrome candidate 1 | 1.433 | 3.62E-05 |
| *WIPI2* | WD repeat domain, phosphoinositide interacting 2 | 1.278 | 0.00225 |
| *Wiz* | Widely-interspaced zinc finger motifs | 1.333 | 0.00319 |
| *WWP2* | WW domain containing E3 ubiquitin protein ligase 2 | 1.263 | 0.00314 |
| *XAB2* | XPA binding protein 2 | 1.28 | 0.00876 |
| *XBP1* | X-box binding protein 1 | -1.268 | 0.00169 |
| *XPC* | Xeroderma pigmentosum, complementation group C | 1.449 | 0.00073 |
| *XRCC1* | X-ray repair complementing defective repair in Chinese hamster cells 1 | 1.395 | 0.00227 |
| *YARS* | Tyrosyl-tRNA synthetase | -1.366 | 0.00049 |
| *YARS2* | Tyrosyl-tRNA synthetase 2, mitochondrial | -1.342 | 0.00549 |
| *YBX3* | Y box binding protein 3 | 1.408 | 0.00052 |
| *YEATS4* | YEATS domain containing 4 | -1.502 | 0.00219 |
| *YPEL3* | Yippee-like 3 (Drosophila) | 1.751 | 2.43E-05 |
| *ZC3H12A* | Zinc finger CCCH-type containing 12A | 1.439 | 0.0046 |
| *ZCCHC24* | Zinc finger, CCHC domain containing 24 | 1.306 | 0.00823 |
| *ZFYVE1* | Zinc finger, FYVE domain containing 1 | 1.396 | 1.43E-05 |
| *ZFYVE27* | Zinc finger, FYVE domain containing 27 | 1.349 | 0.00308 |
| *ZMIZ1* | Zinc finger, MIZ-type containing 1 | 1.531 | 3.22E-06 |
| *ZNF217* | Zinc finger protein 217 | 1.361 | 0.00869 |
| *ZNF219* | Zinc finger protein 219 | 1.32 | 0.00482 |
| *ZNF274* | Zinc finger protein 274 | 1.532 | 0.00027 |
| *ZNF335* | Zinc finger protein 335 | 1.281 | 0.00826 |
| *ZNF395* | Zinc finger protein 395 | 1.42 | 0.00245 |
| *ZNF512B* | Zinc finger protein 512B | 1.295 | 0.00715 |
| *ZNF532* | Zinc finger protein 532 | 1.31 | 0.00055 |
| *ZNF672* | Zinc finger protein 672 | -1.29 | 0.00335 |
| *ZNF703* | Zinc finger protein 703 | 1.534 | 0.00236 |
| *ZSWIM8* | Zinc finger, SWIM-type containing 8 | 1.284 | 0.00225 |

^1^ Fold changes are up or down in restricted fed animals compared to *ad libitum* control animals
