# Supplementary material for: Effect of Dietary Restriction and Subsequent Re-Alimentation on the Transcriptional Profile of Bovine Skeletal Muscle
Source: PLoS One. 2016 Feb 12;11(2):e0149373. doi: 10.1371/journal.pone.0149373 (PMC4752344; doi:10.1371/journal.pone.0149373)
Supplement: S3 Table — (DOCX) [file pone.0149373.s003.docx]

**S3 Table.** Genes commonly differentially expressed in *M. longissimus dorsi* of Holstein Friesian bulls following a 120-day period of dietary restriction at the end of Period 1 and during a subsequent re-alimentation and compensatory growth period of 15-days in Period 2 relative to *ad libitum*-fed controls

| Gene symbol | Gene name | Period 1^1^ | Period 2^1^ |
| --- | --- | --- | --- |
| *ABCA1* | ATP-binding cassette, sub-family A (ABC1), member 1 | 1.705 | 2.501 |
| *ABCC8* | ATP-binding cassette, sub-family C (CFTR/MRP), member 8 | -1.669 | 1.537 |
| *ABR* | Active BCR-related | -1.266 | 1.303 |
| *ACAD10* | Acyl-CoA dehydrogenase family, member 10 | 1.365 | 1.296 |
| *ACADM* | Acyl-CoA dehydrogenase, C-4 to C-12 straight chain | 1.335 | -1.278 |
| *ACLY* | ATP citrate lyase | -3.676 | 1.846 |
| *ACSM1* | Acyl-CoA synthetase medium-chain family member 1 | -3.262 | 3.229 |
| *ADAMTS9* | ADAM metallopeptidase with thrombospondin type 1 motif, 9 | -1.338 | -1.512 |
| *ADCY6* | Adenylate cyclase 6 | -1.383 | 1.316 |
| *AEBP1* | AE binding protein 1 | -2.192 | 2.545 |
| *AGRN* | Agrin | -1.52 | 1.718 |
| *AMPD2* | Adenosine monophosphate deaminase 2 | -1.494 | 1.531 |
| *ANGPT1* | Angiopoietin 1 | -1.699 | -1.61 |
| *ANKRD13B* | Ankyrin repeat domain 13B | -1.749 | 1.444 |
| *AQP4* | Aquaporin 4 | 1.409 | -1.655 |
| *ARID5B* | AT rich interactive domain 5B (MRF1-like) | 1.446 | 1.952 |
| *ARRDC2* | Arrestin domain containing 2 | 1.836 | 2.512 |
| *ATP2B2* | ATPase, Ca++ transporting, plasma membrane 2 | -2.109 | 1.719 |
| *ATP5B* | ATP synthase, H+ transporting, mitochondrial F1 complex, beta polypeptide | 1.359 | -1.322 |
| *ATP5C1* | ATP synthase, H+ transporting, mitochondrial F1 complex, gamma polypeptide 1 | 1.366 | -1.273 |
| *BGN* | Biglycan | -1.403 | 2.097 |
| *C10orf10* | Chromosome 10 open reading frame 10 | 1.616 | 1.566 |
| *CALCOCO1* | Calcium binding and coiled-coil domain 1 | 1.301 | 1.767 |
| *CARD11* | Caspase recruitment domain family, member 11 | -1.983 | 2.068 |
| *CBR4* | Carbonyl reductase 4 | 1.341 | -1.696 |
| *CBX7* | Chromobox homolog 7 | 1.406 | 1.566 |
| *CCDC3* | Coiled-coil domain containing 3 | -2.53 | 2.823 |
| *CCDC43* | Coiled-coil domain containing 43 | 1.259 | -1.26 |
| *CCDC86* | Coiled-coil domain containing 86 | -1.513 | -1.32 |
| *CEP250* | Centrosomal protein 250kDa | -1.394 | 1.396 |
| *CHCHD3* | Coiled-coil-helix-coiled-coil-helix domain containing 3 | 1.297 | -1.314 |
| *CHD3* | Chromodomain helicase DNA binding protein 3 | -1.978 | 1.552 |
| *CIDEC* | Cell death-inducing DFFA-like effector c | -3.852 | 2.637 |
| *CLIC4* | Chloride intracellular channel 4 | -1.343 | -1.483 |
| *COL15A1* | Collagen, type XV, alpha 1 | -1.267 | -1.354 |
| *COX6B1* | Cytochrome c oxidase subunit VIb polypeptide 1 (ubiquitous) | 1.294 | -1.412 |
| *COX7A1* | Cytochrome c oxidase subunit VIIa polypeptide 1 (muscle) | 1.383 | -1.253 |
| *CPT1B* | Carnitine palmitoyltransferase 1B (muscle) | 1.465 | -1.358 |
| *CS* | Citrate synthase | 1.315 | -1.657 |
| *CYB5R1* | Cytochrome b5 reductase 1 | 1.424 | 1.305 |
| *CYB5R4* | Cytochrome b5 reductase 4 | -1.332 | -1.363 |
| *DARS2* | Aspartyl-tRNA synthetase 2, mitochondrial | -1.332 | -1.341 |
| *DBI* | Diazepam binding inhibitor (GABA receptor modulator, acyl-CoA binding protein) | -1.506 | -1.327 |
| *DDO* | D-aspartate oxidase | 1.44 | -1.27 |
| *DDX25* | DEAD (Asp-Glu-Ala-Asp) box helicase 25 | -2.588 | 1.575 |
| *DHRS3* | Dehydrogenase/reductase (SDR family) member 3 | -1.389 | 1.485 |
| *DOK4* | Docking protein 4 | 1.702 | -1.673 |
| *DUSP26* | Dual specificity phosphatase 26 (putative) | 1.679 | 1.422 |
| *EGLN3* | Egl-9 family hypoxia-inducible factor 3 | -1.665 | 1.512 |
| *EGR1* | Early growth response 1 | -2.135 | 1.861 |
| *ETS2* | V-ets avian erythroblastosis virus E26 oncogene homolog 2 | -1.501 | 1.743 |
| *EXTL1* | Exostosin-like glycosyltransferase 1 | -2.28 | -1.609 |
| *FABP3* | Fatty acid binding protein 3, muscle and heart (mammary-derived growth inhibitor) | -1.457 | -1.307 |
| *FAM173A* | Family with sequence similarity 173, member A | -1.667 | 1.429 |
| *FDFT1* | Farnesyl-diphosphate farnesyltransferase 1 | 1.252 | 1.367 |
| *FGFR4* | Fibroblast growth factor receptor 4 | -1.644 | 1.458 |
| *FRMD4A* | FERM domain containing 4A | -1.376 | 1.338 |
| *G6PD* | Glucose-6-phosphate dehydrogenase | -2.284 | 1.735 |
| *GAB2* | GRB2-associated binding protein 2 | 1.33 | 1.484 |
| *GATSL2* | GATS protein-like 2 | 1.318 | 1.581 |
| *GBAS* | Glioblastoma amplified sequence | 1.315 | -1.276 |
| *GOT1* | Glutamic-oxaloacetic transaminase 1, soluble | 1.292 | -1.284 |
| *GPR146* | G protein-coupled receptor 146 | -1.391 | 1.463 |
| *GRB10* | Growth factor receptor-bound protein 10 | 1.322 | 1.456 |
| *GRK4* | G protein-coupled receptor kinase 4 | -1.73 | 1.67 |
| *HECTD4* | HECT domain containing E3 ubiquitin protein ligase 4 | -1.376 | 1.272 |
| *HMGCR* | 3-hydroxy-3-methylglutaryl-CoA reductase | -1.461 | -1.642 |
| *HPCAL4* | Hippocalcin like 4 | 1.531 | -2.606 |
| *IARS* | Isoleucyl-tRNA synthetase | -1.26 | -1.444 |
| *IFT122* | Intraflagellar transport 122 homolog (Chlamydomonas) | -1.32 | 1.492 |
| *IFT140* | Intraflagellar transport 140 homolog (Chlamydomonas) | -1.884 | 1.738 |
| *IMPA2* | Inositol(myo)-1(or 4)-monophosphatase 2 | 1.54 | 1.807 |
| *IRAK1* | Interleukin-1 receptor-associated kinase 1 | -1.588 | 1.35 |
| *ITGB5* | Integrin, beta 5 | -1.301 | 1.46 |
| *ITPRIP* | Inositol 1,4,5-trisphosphate receptor interacting protein | 1.374 | 1.276 |
| *JADE2* | Jade family PHD finger 2 | 1.725 | 1.957 |
| *JUNB* | Jun B proto-oncogene | -1.874 | 2.041 |
| *KCNMB1* | Potassium large conductance calcium-activated channel, subfamily M, beta member 1 | -2.026 | 1.628 |
| *KCP* | Kielin/chordin-like protein | -1.75 | 1.469 |
| *KDM5C* | Lysine (K)-specific demethylase 5C | -1.437 | 1.26 |
| *KLF15* | Kruppel-like factor 15 | 1.413 | 1.533 |
| *KLHL22* | Kelch-like family member 22 | 1.334 | 1.426 |
| *LAMA3* | Laminin, alpha 3 | -2.087 | 1.906 |
| *LIN37* | Lin-37 homolog (C. elegans) | 1.353 | 1.334 |
| *LMNB1* | Lamin B1 | -1.494 | -2.17 |
| *LMOD3* | Leiomodin 3 (fetal) | -1.393 | 1.37 |
| *LTBP2* | Latent transforming growth factor beta binding protein 2 | 1.532 | 1.755 |
| *MAOA* | Monoamine oxidase A | 1.616 | -1.57 |
| *MAOB* | Monoamine oxidase B | -1.574 | 1.593 |
| *MARC2* | Mitochondrial amidoxime reducing component 2 | -1.366 | 1.33 |
| *ME3* | Malic enzyme 3, NADP(+)-dependent, mitochondrial | 1.742 | 1.769 |
| *MED12* | Mediator complex subunit 12 | -1.533 | 1.295 |
| *MFSD4* | Major facilitator superfamily domain containing 4 | -1.956 | 1.874 |
| *MLLT6* | Myeloid/lymphoid or mixed-lineage leukemia (trithorax homolog, Drosophila); 6 | 1.334 | 1.645 |
| *MSMO1* | Methylsterol monooxygenase 1 | -1.423 | -1.971 |
| *MTHFR* | Methylenetetrahydrofolate reductase (NAD(P)H) | 1.306 | 1.313 |
| *MYF5* | Myogenic factor 5 | -1.575 | 1.503 |
| *MYH14* | Myosin, heavy chain 14, non-muscle | 1.497 | 1.477 |
| *MYLIP* | Myosin regulatory light chain interacting protein | 1.32 | 1.28 |
| *MYO10* | Myosin X | -1.526 | 1.698 |
| *NDRG2* | NDRG family member 2 | -1.921 | 1.314 |
| *NME3* | NME/NM23 nucleoside diphosphate kinase 3 | 1.327 | 1.315 |
| *NPNT* | Nephronectin | -4.839 | 2.188 |
| *NQO1* | NAD(P)H dehydrogenase, quinone 1 | -1.309 | -1.271 |
| *NREP* | Neuronal regeneration related protein | -1.326 | -1.395 |
| *NSUN4* | NOP2/Sun domain family, member 4 | 1.322 | -1.321 |
| *OSBP2* | Oxysterol binding protein 2 | -2.018 | 1.961 |
| *OTUD3* | OTU domain containing 3 | -1.257 | 1.396 |
| *PARM1* | Prostate androgen-regulated mucin-like protein 1 | -1.573 | 2.437 |
| *PCBD1* | Pterin-4 alpha-carbinolamine dehydratase/dimerization cofactor of hepatocyte nuclear factor 1 alpha | 3.309 | -4.002 |
| *PCDH12* | Protocadherin 12 | -1.825 | -1.756 |
| *PDHB* | Pyruvate dehydrogenase (lipoamide) beta | 1.312 | -1.283 |
| *PGK1* | Phosphoglycerate kinase 1 | -1.342 | -1.351 |
| *PGPEP1L* | Pyroglutamyl-peptidase I-like | 1.924 | 1.818 |
| *PIK3IP1* | Phosphoinositide-3-kinase interacting protein 1 | 1.352 | 1.945 |
| *PLA2R1* | Phospholipase A2 receptor 1, 180kDa | -2.577 | 1.678 |
| *PLIN2* | Perilipin 2 | 1.805 | 1.731 |
| *PMEPA1* | Prostate transmembrane protein, androgen induced 1 | -1.63 | 1.711 |
| *PNPLA2* | Patatin-like phospholipase domain containing 2 | 1.767 | 1.394 |
| *PNPO* | Pyridoxamine 5'-phosphate oxidase | -1.317 | -1.286 |
| *POLD4* | Polymerase (DNA-directed), delta 4, accessory subunit | 1.297 | 1.308 |
| *PPA1* | Pyrophosphatase (inorganic) 1 | -1.548 | -1.368 |
| *PRKAG3* | Protein kinase, AMP-activated, gamma 3 non-catalytic subunit | -1.32 | 1.35 |
| *PSPH* | Phosphoserine phosphatase | -2.462 | -4.508 |
| *PTPLA* | Protein tyrosine phosphatase-like (proline instead of catalytic arginine), member A | -1.311 | -1.375 |
| *PXMP2* | Peroxisomal membrane protein 2, 22kDa | 1.273 | 1.444 |
| *RAD1* | RAD1 homolog (S. pombe) | -1.523 | -1.516 |
| *RASA4* | RAS p21 protein activator 4 | 1.556 | 1.44 |
| *RFXANK* | Regulatory factor X-associated ankyrin-containing protein | -1.869 | 1.479 |
| *RPL3* | Ribosomal protein L3 | -1.309 | -1.621 |
| *SELT* | Selenoprotein T | -1.44 | -1.372 |
| *SEPT5* | Septin 5 | -2.201 | 1.714 |
| *SERINC5* | Serine incorporator 5 | -1.581 | 1.549 |
| *SIPA1L2* | Signal-induced proliferation-associated 1 like 2 | -1.414 | 1.421 |
| *SLC25A12* | Solute carrier family 25 (aspartate/glutamate carrier), member 12 | 1.329 | -1.304 |
| *SLC2A4RG* | SLC2A4 regulator | 1.399 | 1.464 |
| *SLC7A1* | Solute carrier family 7 (cationic amino acid transporter, y+ system), member 1 | 1.418 | -1.574 |
| *SLC7A4* | Solute carrier family 7, member 4 | -2.098 | 2.214 |
| *SLC7A8* | Solute carrier family 7 (amino acid transporter light chain, L system), member 8 | -2.007 | 1.738 |
| *SMTNL1* | Smoothelin-like 1 | 1.673 | 1.48 |
| *SOCS2* | Suppressor of cytokine signaling 2 | 2.746 | -2.033 |
| *SOGA2* | SOGA family member 2 | -1.584 | 1.648 |
| *SORL1* | Sortilin-related receptor, L(DLR class) A repeats containing | -1.564 | 1.914 |
| *SPSB3* | SplA/ryanodine receptor domain and SOCS box containing 3 | 1.367 | 1.435 |
| *SREBF1* | Sterol regulatory element binding transcription factor 1 | 1.343 | 1.44 |
| *STAT5A* | Signal transducer and activator of transcription 5A | 1.276 | 1.505 |
| *STAT5B* | Signal transducer and activator of transcription 5B | 1.543 | 1.395 |
| *SZT2* | Seizure threshold 2 homolog (mouse) | -1.293 | 1.296 |
| *TBC1D17* | TBC1 domain family, member 17 | 1.295 | 1.389 |
| *TEAD4* | TEA domain family member 4 | -1.316 | 1.434 |
| *THBS1* | Thrombospondin 1 | -1.987 | 1.85 |
| *TM2D1* | TM2 domain containing 1 | -1.314 | -1.253 |
| *TMEM120A* | Transmembrane protein 120A | 1.447 | -1.425 |
| *TMEM182* | Transmembrane protein 182 | 1.266 | -1.295 |
| *TMEM51* | Transmembrane protein 51 | -1.994 | 1.74 |
| *TNS1* | Tensin 1 | 1.452 | 1.398 |
| *TOMM40L* | Translocase of outer mitochondrial membrane 40 homolog (yeast)-like | 1.304 | -1.424 |
| *TPPP2* | Tubulin polymerization-promoting protein family member 2 | -3.331 | 1.959 |
| *TRIM44* | Tripartite motif containing 44 | -1.607 | 1.566 |
| *TTC9* | Tetratricopeptide repeat domain 9 | 1.533 | 1.606 |
| *UBC* | Ubiquitin C | 1.276 | 1.415 |
| *UQCRC2* | Ubiquinol-cytochrome c reductase core protein II | 1.285 | -1.387 |
| *VDAC2* | Voltage-dependent anion channel 2 | 1.331 | -1.269 |
| *VOPP1* | Vesicular, overexpressed in cancer, prosurvival protein 1 | 1.542 | 1.347 |
| *WBSCR17* | Williams-Beuren syndrome chromosome region 17 | -2.107 | 1.626 |
| *WDR62* | WD repeat domain 62 | -1.53 | 1.689 |
| *YBX3* | Y box binding protein 3 | 1.539 | 1.408 |
| *YPEL3* | Yippee-like 3 (Drosophila) | 1.322 | 1.751 |
| *ZMIZ1* | Zinc finger, MIZ-type containing 1 | -1.355 | 1.531 |
| *ZNF274* | Zinc finger protein 274 | 1.336 | 1.532 |
| *ZNF672* | Zinc finger protein 672 | 1.359 | -1.29 |

^1^ Fold changes are up or down in restricted fed animals compared to *ad libitum* control animals
