# Supplementary material for: Effect of Dietary Restriction and Subsequent Re-Alimentation on the Transcriptional Profile of Bovine Skeletal Muscle
Source: PLoS One. 2016 Feb 12;11(2):e0149373. doi: 10.1371/journal.pone.0149373 (PMC4752344; doi:10.1371/journal.pone.0149373)
Supplement: S4 Table — (DOCX) [file pone.0149373.s004.docx]

**S4 Table.** Genes differentially expressed in *M. longissimus dorsi* of Holstein Friesian bulls (n = 10) following a 15-day period of re-alimentation and compensatory growth in Period 2 relative to animals fed a restricted diet for 120 days at the end of Period 1(n = 10)

| Gene  Symbol | Gene Name | Fold change^1^ | P-value |
| --- | --- | --- | --- |
| MARCH3 | Membrane-associated ring finger (C3HC4) 3, E3 ubiquitin protein ligase | -1.813 | 1.81E-04 |
| SEPT5 | Septin 5 | 2.071 | 9.67E-07 |
| MARCH6 | Membrane-associated ring finger (C3HC4) 6, E3 ubiquitin protein ligase | -1.382 | 1.50E-04 |
| SEPT6 | Septin 6 | 1.544 | 6.35E-04 |
| SEPT8 | Septin 8 | 1.3 | 9.64E-03 |
| SEPT11 | Septin 11 | -1.404 | 5.25E-04 |
| AAAS | Achalasia, adrenocortical insufficiency, alacrimia | 1.848 | 4.16E-09 |
| AASS | Aminoadipate-semialdehyde synthase | -1.981 | 1.95E-10 |
| ABAT | 4-aminobutyrate aminotransferase | -1.34 | 1.08E-03 |
| ABCA1 | ATP-binding cassette, sub-family A (ABC1), member 1 | -4.549 | 3.99E-25 |
| ABCA3 | ATP-binding cassette, sub-family A (ABC1), member 3 | 1.535 | 6.72E-03 |
| ABCB10 | ATP-binding cassette, sub-family B (MDR/TAP), member 10 | -1.279 | 5.85E-04 |
| ABCB8 | ATP-binding cassette, sub-family B (MDR/TAP), member 8 | 2.096 | 2.97E-12 |
| ABCC1 | ATP-binding cassette, sub-family C (CFTR/MRP), member 1 | -1.26 | 6.61E-03 |
| ABCC9 | ATP-binding cassette, sub-family C (CFTR/MRP), member 9 | -1.347 | 7.87E-03 |
| ABCF3 | ATP-binding cassette, sub-family F (GCN20), member 3 | 1.34 | 1.75E-05 |
| ABCG1 | ATP-binding cassette, sub-family G (WHITE), member 1 | -2.525 | 7.03E-08 |
| ABHD11 | Abhydrolase domain containing 11 | 1.88 | 7.15E-09 |
| ABHD14B | Abhydrolase domain containing 14B | 1.302 | 1.31E-02 |
| ABHD3 | Abhydrolase domain containing 3 | 1.502 | 1.59E-03 |
| ABHD8 | Abhydrolase domain containing 8 | 1.716 | 2.71E-09 |
| ABI2 | Abl-interactor 2 | -1.638 | 1.44E-03 |
| ABI3 | ABI family, member 3 | 1.456 | 7.73E-03 |
| ABI3BP | ABI family, member 3 (NESH) binding protein | 1.393 | 1.27E-03 |
| ABL2 | ABL proto-oncogene 2, non-receptor tyrosine kinase | -1.432 | 2.48E-02 |
| ABRA | Actin binding Rho activating protein | 1.646 | 4.63E-05 |
| ABT1 | Activator of basal transcription 1 | 1.262 | 8.56E-03 |
| ACAA1 | Acetyl-CoA acyltransferase 1 | -1.267 | 1.52E-03 |
| ACAD10 | Acyl-CoA dehydrogenase family, member 10 | -1.771 | 1.62E-11 |
| ACADL | Acyl-CoA dehydrogenase, long chain | 1.336 | 1.44E-03 |
| ACADM | Acyl-CoA dehydrogenase, C-4 to C-12 straight chain | -1.252 | 7.85E-03 |
| ACADS | Acyl-CoA dehydrogenase, C-2 to C-3 short chain | 1.272 | 6.22E-03 |
| ACAT2 | Acetyl-CoA acetyltransferase 2 | 1.853 | 1.00E-03 |
| ACCS | 1-aminocyclopropane-1-carboxylate synthase homolog (Arabidopsis)(non-functional) | 1.576 | 4.28E-04 |
| ACE | Angiotensin I converting enzyme | -1.311 | 1.22E-02 |
| ACKR3 | Atypical chemokine receptor 3 | 1.457 | 3.31E-05 |
| ACLY | ATP citrate lyase | 1.387 | 2.05E-02 |
| ACO2 | Aconitase 2, mitochondrial | -1.527 | 9.44E-07 |
| ACOX1 | Acyl-CoA oxidase 1, palmitoyl | -1.628 | 3.19E-05 |
| ACSF2 | Acyl-CoA synthetase family member 2 | 1.426 | 2.80E-03 |
| ACSL1 | Acyl-CoA synthetase long-chain family member 1 | 1.293 | 4.78E-04 |
| ACSL5 | Acyl-CoA synthetase long-chain family member 5 | 1.494 | 4.77E-03 |
| ACSM5 | Acyl-CoA synthetase medium-chain family member 5 | -1.796 | 5.53E-12 |
| ACSS3 | Acyl-CoA synthetase short-chain family member 3 | 1.274 | 1.72E-02 |
| ACTA2 | Actin, alpha 2, smooth muscle, aorta | 1.254 | 1.43E-02 |
| ACTB | Actin, beta | 1.251 | 1.03E-02 |
| ACTC1 | Actin, alpha, cardiac muscle 1 | 9.798 | 3.83E-21 |
| ACTG1 | Actin gamma 1 | 1.35 | 6.19E-05 |
| ACTN1 | Actinin, alpha 1 | 1.257 | 2.84E-02 |
| Actn3 | Actinin alpha 3 | -1.319 | 3.98E-03 |
| ACTR1A | ARP1 actin-related protein 1 homolog A, centractin alpha (yeast) | 1.253 | 2.12E-03 |
| ACTR3 | ARP3 actin-related protein 3 homolog (yeast) | 1.474 | 2.69E-06 |
| ACVR1B | Activin A receptor, type IB | -1.313 | 1.70E-03 |
| ACVR2B | Activin A receptor, type IIB | -1.429 | 2.35E-03 |
| ACYP1 | Acylphosphatase 1, erythrocyte (common) type | 1.317 | 1.62E-02 |
| ADA | Adenosine deaminase | 1.662 | 3.61E-06 |
| ADAL | Adenosine deaminase-like | 1.435 | 2.28E-04 |
| ADAM17 | ADAM metallopeptidase domain 17 | 1.289 | 1.10E-02 |
| ADAM19 | ADAM metallopeptidase domain 19 | 1.734 | 1.19E-06 |
| ADAMTS2 | ADAM metallopeptidase with thrombospondin type 1 motif, 2 | 1.624 | 9.33E-06 |
| ADAMTS20 | ADAM metallopeptidase with thrombospondin type 1 motif, 20 | 1.334 | 1.18E-02 |
| ADAMTS9 | ADAM metallopeptidase with thrombospondin type 1 motif, 9 | 2.011 | 2.11E-11 |
| ADAMTSL2 | ADAMTS-like 2 | 1.637 | 6.65E-04 |
| ADAMTSL4 | ADAMTS-like 4 | -1.365 | 2.99E-03 |
| ADGRE5 | Adhesion G protein-coupled receptor E5 | 1.27 | 6.38E-03 |
| ADGRF5 | Adhesion G protein-coupled receptor F5 | -1.373 | 2.15E-04 |
| ADGRL1 | Adhesion G protein-coupled receptor L1 | 1.319 | 1.69E-02 |
| ADH5 | Alcohol dehydrogenase 5 (class III), chi polypeptide | 1.279 | 1.94E-03 |
| ADHFE1 | Alcohol dehydrogenase, iron containing, 1 | -1.73 | 2.25E-10 |
| ADIPOR2 | Adiponectin receptor 2 | -1.454 | 2.04E-06 |
| ADNP2 | ADNP homeobox 2 | -1.42 | 3.47E-05 |
| ADRB2 | Adrenoceptor beta 2, surface | 1.58 | 6.50E-06 |
| ADSS | Adenylosuccinate synthase | 1.341 | 3.91E-03 |
| AFAP1L1 | Actin filament associated protein 1-like 1 | 1.459 | 2.05E-03 |
| AFF1 | AF4/FMR2 family, member 1 | -1.552 | 8.69E-06 |
| AFF4 | AF4/FMR2 family, member 4 | -1.846 | 1.68E-02 |
| AGFG1 | ArfGAP with FG repeats 1 | 1.406 | 2.42E-03 |
| AGL | Amylo-alpha-1, 6-glucosidase, 4-alpha-glucanotransferase | -1.4 | 1.07E-02 |
| AGMAT | Agmatine ureohydrolase (agmatinase) | -1.721 | 8.63E-05 |
| AGO4 | Argonaute RISC catalytic component 4 | -1.365 | 4.50E-03 |
| AGRN | Agrin | 1.384 | 1.17E-03 |
| AGTRAP | Angiotensin II receptor-associated protein | -1.266 | 1.35E-02 |
| AHCY | Adenosylhomocysteinase | 1.368 | 2.57E-04 |
| AHDC1 | AT hook, DNA binding motif, containing 1 | 1.312 | 1.72E-03 |
| AHNAK | AHNAK nucleoprotein | -1.755 | 2.62E-10 |
| AHSA1 | AHA1, activator of heat shock 90kDa protein ATPase homolog 1 (yeast) | 1.251 | 4.62E-03 |
| AIP | Aryl hydrocarbon receptor interacting protein | 1.46 | 1.92E-04 |
| AKAP12 | A kinase (PRKA) anchor protein 12 | -1.467 | 9.67E-05 |
| AKAP8 | A kinase (PRKA) anchor protein 8 | -1.454 | 2.92E-07 |
| AKIRIN2 | Akirin 2 | 1.275 | 3.83E-03 |
| AKR1B1 | Aldo-keto reductase family 1, member B1 (aldose reductase) | 1.409 | 3.32E-06 |
| AKR1C3 | Aldo-keto reductase family 1, member C3 | -1.563 | 2.42E-02 |
| AKR7A2 | Aldo-keto reductase family 7, member A2 | 1.299 | 4.39E-03 |
| AKTIP | AKT interacting protein | -1.358 | 4.85E-07 |
| ALCAM | Activated leukocyte cell adhesion molecule | -2.433 | 6.03E-07 |
| ALDH18A1 | Aldehyde dehydrogenase 18 family, member A1 | 1.287 | 2.15E-04 |
| ALDH1A1 | Aldehyde dehydrogenase 1 family, member A1 | 1.259 | 1.23E-02 |
| ALDH6A1 | Aldehyde dehydrogenase 6 family, member A1 | -1.29 | 2.30E-04 |
| ALDOA | Aldolase A, fructose-bisphosphate | -1.306 | 1.60E-03 |
| ALG3 | ALG3, alpha-1,3- mannosyltransferase | 1.677 | 5.81E-07 |
| ALG9 | ALG9, alpha-1,2-mannosyltransferase | 1.346 | 2.19E-02 |
| ALKBH3 | AlkB homolog 3, alpha-ketoglutarate-dependent dioxygenase | -1.367 | 6.00E-07 |
| ALS2 | Amyotrophic lateral sclerosis 2 (juvenile) | -1.345 | 1.35E-05 |
| ALS2CL | ALS2 C-terminal like | -1.63 | 4.45E-05 |
| AMD1 | Adenosylmethionine decarboxylase 1 | 1.347 | 1.40E-02 |
| AMER1 | APC membrane recruitment protein 1 | -1.49 | 6.97E-05 |
| AMIGO2 | Adhesion molecule with Ig-like domain 2 | 1.834 | 4.00E-05 |
| AMMECR1L | AMMECR1-like | 1.251 | 1.65E-02 |
| AMOT | Angiomotin | -1.392 | 6.03E-06 |
| AMOTL1 | Angiomotin like 1 | -1.303 | 2.38E-04 |
| AMPD1 | Adenosine monophosphate deaminase 1 | -1.512 | 5.11E-06 |
| AMY2A | Amylase, alpha 2A (pancreatic) | -1.503 | 1.53E-04 |
| ANAPC10 | Anaphase promoting complex subunit 10 | -1.335 | 5.42E-04 |
| ANAPC4 | Anaphase promoting complex subunit 4 | 1.396 | 3.71E-04 |
| ANAPC7 | Anaphase promoting complex subunit 7 | 1.311 | 1.91E-02 |
| ANGPT1 | Angiopoietin 1 | 2.265 | 5.28E-07 |
| ANGPT2 | Angiopoietin 2 | 1.546 | 9.22E-03 |
| ANGPT4 | Angiopoietin 4 | -1.445 | 2.03E-03 |
| ANGPTL1 | Angiopoietin-like 1 | 1.588 | 9.90E-04 |
| ANGPTL4 | Angiopoietin-like 4 | 2.98 | 5.89E-06 |
| ANK1 | Ankyrin 1, erythrocytic | 1.3 | 8.63E-04 |
| ANKH | ANKH inorganic pyrophosphate transport regulator | -1.386 | 1.79E-05 |
| ANKLE2 | Ankyrin repeat and LEM domain containing 2 | -1.319 | 9.85E-06 |
| ANKRA2 | Ankyrin repeat, family A (RFXANK-like), 2 | -1.555 | 9.05E-07 |
| ANKRD1 | Ankyrin repeat domain 1 (cardiac muscle) | 1.646 | 6.82E-04 |
| ANKRD13B | Ankyrin repeat domain 13B | 1.511 | 3.49E-04 |
| ANKRD2 | Ankyrin repeat domain 2 (stretch responsive muscle) | 2.375 | 5.59E-06 |
| ANKRD28 | Ankyrin repeat domain 28 | -1.313 | 2.43E-03 |
| ANKRD37 | Ankyrin repeat domain 37 | -1.928 | 3.80E-06 |
| ANKRD40 | Ankyrin repeat domain 40 | 1.471 | 1.56E-06 |
| ANKRD54 | Ankyrin repeat domain 54 | 1.597 | 7.13E-04 |
| ANKRD9 | Ankyrin repeat domain 9 | -1.543 | 5.16E-05 |
| ANKS1A | Ankyrin repeat and sterile alpha motif domain containing 1A | -1.296 | 1.33E-02 |
| ANO6 | Anoctamin 6 | -1.357 | 4.23E-03 |
| ANTXR2 | Anthrax toxin receptor 2 | 1.349 | 5.50E-04 |
| ANXA2 | Annexin A2 | 1.6 | 2.23E-05 |
| ANXA7 | Annexin A7 | -1.482 | 1.23E-05 |
| ANXA9 | Annexin A9 | 1.824 | 2.32E-05 |
| AP1G2 | Adaptor-related protein complex 1, gamma 2 subunit | 1.253 | 1.22E-02 |
| AP1S1 | Adaptor-related protein complex 1, sigma 1 subunit | 1.335 | 6.70E-03 |
| AP1S2 | Adaptor-related protein complex 1, sigma 2 subunit | 1.316 | 9.42E-03 |
| AP2A1 | Adaptor-related protein complex 2, alpha 1 subunit | -1.276 | 3.72E-03 |
| AP2B1 | Adaptor-related protein complex 2, beta 1 subunit | -1.321 | 8.14E-05 |
| AP3M2 | Adaptor-related protein complex 3, mu 2 subunit | -1.277 | 2.79E-03 |
| AP4B1 | Adaptor-related protein complex 4, beta 1 subunit | 1.271 | 3.91E-03 |
| AP4S1 | Adaptor-related protein complex 4, sigma 1 subunit | -1.283 | 3.12E-03 |
| AP5S1 | Adaptor-related protein complex 5, sigma 1 subunit | 1.396 | 1.40E-03 |
| APBB1 | Amyloid beta (A4) precursor protein-binding, family B, member 1 (Fe65) | -1.479 | 2.83E-03 |
| APBB2 | Amyloid beta (A4) precursor protein-binding, family B, member 2 | -1.471 | 5.33E-04 |
| APCDD1 | Adenomatosis polyposis coli down-regulated 1 | -2.013 | 1.98E-07 |
| APMAP | Adipocyte plasma membrane associated protein | -1.411 | 9.92E-05 |
| APOD | Apolipoprotein D | -1.496 | 1.44E-03 |
| APPL1 | Adaptor protein, phosphotyrosine interaction, PH domain and leucine zipper containing 1 | -1.31 | 4.54E-04 |
| APPL2 | Adaptor protein, phosphotyrosine interaction, PH domain and leucine zipper containing 2 | -1.368 | 8.13E-05 |
| APRT | Adenine phosphoribosyltransferase | 1.447 | 3.06E-05 |
| AQP3 | Aquaporin 3 (Gill blood group) | -1.834 | 1.34E-04 |
| ARAP3 | ArfGAP with RhoGAP domain, ankyrin repeat and PH domain 3 | 1.381 | 1.15E-02 |
| Arf2 | ADP-ribosylation factor 2 | 1.468 | 2.56E-06 |
| ARFGAP2 | ADP-ribosylation factor GTPase activating protein 2 | -1.374 | 2.40E-04 |
| ARFGEF2 | ADP-ribosylation factor guanine nucleotide-exchange factor 2 (brefeldin A-inhibited) | -1.288 | 4.80E-04 |
| ARG2 | Arginase 2 | 2.732 | 3.31E-06 |
| ARHGAP12 | Rho GTPase activating protein 12 | -1.286 | 2.32E-02 |
| ARHGAP18 | Rho GTPase activating protein 18 | 1.493 | 1.76E-03 |
| ARHGAP19 | Rho GTPase activating protein 19 | -1.381 | 3.98E-03 |
| ARHGAP44 | Rho GTPase activating protein 44 | 1.848 | 1.40E-04 |
| ARHGAP6 | Rho GTPase activating protein 6 | -1.367 | 3.01E-03 |
| ARHGDIB | Rho GDP dissociation inhibitor (GDI) beta | 1.332 | 3.92E-03 |
| ARHGEF15 | Rho guanine nucleotide exchange factor (GEF) 15 | 1.371 | 2.20E-02 |
| ARHGEF2 | Rho/Rac guanine nucleotide exchange factor (GEF) 2 | 1.291 | 1.32E-03 |
| ARHGEF40 | Rho guanine nucleotide exchange factor (GEF) 40 | 1.636 | 2.82E-06 |
| ARID1B | AT rich interactive domain 1B (SWI1-like) | -1.267 | 3.09E-04 |
| ARID5B | AT rich interactive domain 5B (MRF1-like) | -3.252 | 4.07E-15 |
| ARL4A | ADP-ribosylation factor-like 4A | -1.262 | 6.22E-03 |
| ARL6 | ADP-ribosylation factor-like 6 | 1.671 | 3.28E-04 |
| ARL6IP5 | ADP-ribosylation factor-like 6 interacting protein 5 | 1.261 | 7.29E-03 |
| ARMC5 | Armadillo repeat containing 5 | 1.346 | 1.86E-02 |
| ARMC6 | Armadillo repeat containing 6 | 1.266 | 1.26E-02 |
| ARMCX2 | Armadillo repeat containing, X-linked 2 | 1.575 | 1.15E-03 |
| ARNTL | Aryl hydrocarbon receptor nuclear translocator-like | -1.907 | 1.38E-04 |
| ARPC3 | Actin related protein 2/3 complex, subunit 3, 21kDa | 1.366 | 2.25E-03 |
| ARPC5 | Actin related protein 2/3 complex, subunit 5, 16kDa | 1.31 | 1.45E-03 |
| ARRDC2 | Arrestin domain containing 2 | -3.809 | 5.77E-11 |
| ARSB | Arylsulfatase B | 2.058 | 6.02E-07 |
| ARSK | Arylsulfatase family, member K | 1.572 | 8.30E-03 |
| ART3 | ADP-ribosyltransferase 3 | -2.27 | 8.60E-09 |
| ART5 | ADP-ribosyltransferase 5 | -3.136 | 1.21E-04 |
| ASB18 | Ankyrin repeat and SOCS box containing 18 | -1.344 | 1.21E-03 |
| ASCC1 | Activating signal cointegrator 1 complex subunit 1 | -2.245 | 6.24E-10 |
| ASF1B | Anti-silencing function 1B histone chaperone | 3.372 | 1.01E-19 |
| ASH2L | Ash2 (absent, small, or homeotic)-like (Drosophila) | -1.336 | 1.32E-04 |
| ASNS | Asparagine synthetase (glutamine-hydrolyzing) | 6.681 | 1.21E-10 |
| ASPN | Asporin | 2.594 | 5.81E-06 |
| ASS1 | Argininosuccinate synthase 1 | -2.542 | 9.84E-12 |
| ATAD2B | ATPase family, AAA domain containing 2B | -1.45 | 1.55E-02 |
| ATE1 | Arginyltransferase 1 | -1.377 | 4.44E-04 |
| ATF5 | Activating transcription factor 5 | 1.812 | 6.97E-03 |
| ATF7IP | Activating transcription factor 7 interacting protein | -1.326 | 5.07E-03 |
| ATG13 | Autophagy related 13 | -1.306 | 9.83E-04 |
| ATG2B | Autophagy related 2B | -1.475 | 2.13E-03 |
| ATG9A | Autophagy related 9A | -1.379 | 6.94E-06 |
| ATM | ATM serine/threonine kinase | -1.461 | 2.11E-02 |
| ATOH8 | Atonal bHLH transcription factor 8 | 1.788 | 9.91E-04 |
| ATP11A | ATPase, class VI, type 11A | -1.322 | 1.09E-02 |
| ATP13A3 | ATPase type 13A3 | -1.668 | 3.89E-03 |
| ATP1A4 | ATPase, Na+/K+ transporting, alpha 4 polypeptide | -1.672 | 2.04E-03 |
| ATP1B2 | ATPase, Na+/K+ transporting, beta 2 polypeptide | -1.426 | 7.67E-03 |
| ATP1B3 | ATPase, Na+/K+ transporting, beta 3 polypeptide | 1.52 | 2.43E-04 |
| ATP1B4 | ATPase, Na+/K+ transporting, beta 4 polypeptide | -1.399 | 4.48E-04 |
| ATP2A2 | ATPase, Ca++ transporting, cardiac muscle, slow twitch 2 | -1.485 | 1.56E-06 |
| ATP2C2 | ATPase, Ca++ transporting, type 2C, member 2 | -1.524 | 9.98E-03 |
| ATP5S | ATP synthase, H+ transporting, mitochondrial Fo complex, subunit s (factor B) | -1.343 | 3.49E-04 |
| ATP6V0A1 | ATPase, H+ transporting, lysosomal V0 subunit a1 | -1.252 | 3.98E-04 |
| ATP6V1A | ATPase, H+ transporting, lysosomal 70kDa, V1 subunit A | 1.33 | 1.08E-02 |
| ATP7A | ATPase, Cu++ transporting, alpha polypeptide | -4.446 | 3.64E-08 |
| ATP8A1 | ATPase, aminophospholipid transporter (APLT), class I, type 8A, member 1 | -1.824 | 1.23E-03 |
| ATXN1 | Ataxin 1 | -1.529 | 1.46E-03 |
| ATXN10 | Ataxin 10 | 1.269 | 3.96E-03 |
| ATXN7 | Ataxin 7 | -1.277 | 2.23E-03 |
| AVEN | Apoptosis, caspase activation inhibitor | 1.275 | 3.34E-04 |
| AZIN1 | Antizyme inhibitor 1 | 1.55 | 2.78E-03 |
| B2M | Beta-2-microglobulin | 1.312 | 1.68E-02 |
| B3GALT2 | UDP-Gal:betaGlcNAc beta 1,3-galactosyltransferase, polypeptide 2 | 1.712 | 1.39E-04 |
| B9D1 | B9 protein domain 1 | 1.529 | 9.99E-04 |
| B9D2 | B9 protein domain 2 | 1.688 | 4.12E-06 |
| BAG4 | BCL2-associated athanogene 4 | -1.519 | 1.89E-04 |
| BAK1 | BCL2-antagonist/killer 1 | 1.673 | 4.27E-05 |
| BAX | BCL2-associated X protein | 1.873 | 1.94E-06 |
| BCAR3 | Breast cancer anti-estrogen resistance 3 | -1.436 | 4.87E-03 |
| BCAS1 | Breast carcinoma amplified sequence 1 | 1.529 | 6.17E-03 |
| BCAS3 | Breast carcinoma amplified sequence 3 | -1.511 | 4.54E-07 |
| BCAT2 | Branched chain amino-acid transaminase 2, mitochondrial | 1.375 | 1.70E-02 |
| BCKDHA | Branched chain keto acid dehydrogenase E1, alpha polypeptide | -1.357 | 5.07E-04 |
| BCL2L1 | BCL2-like 1 | -1.278 | 5.17E-03 |
| BCL2L12 | BCL2-like 12 (proline rich) | 1.358 | 4.70E-03 |
| BCL6 | B-cell CLL/lymphoma 6 | 1.95 | 5.39E-04 |
| BCL6B | B-cell CLL/lymphoma 6, member B | 1.427 | 4.77E-03 |
| BCLAF1 | BCL2-associated transcription factor 1 | -1.452 | 1.86E-05 |
| BCORL1 | BCL6 corepressor-like 1 | 1.479 | 2.16E-04 |
| BCS1L | BC1 (ubiquinol-cytochrome c reductase) synthesis-like | 1.268 | 1.18E-03 |
| BEND5 | BEN domain containing 5 | -1.459 | 2.92E-03 |
| BGN | Biglycan | 1.854 | 8.05E-07 |
| BID | BH3 interacting domain death agonist | -1.391 | 7.20E-03 |
| BIRC5 | Baculoviral IAP repeat containing 5 | 2.622 | 2.23E-12 |
| BLCAP | Bladder cancer associated protein | -1.341 | 1.21E-03 |
| BLM | Bloom syndrome, RecQ helicase-like | -1.468 | 8.68E-03 |
| BLOC1S2 | Biogenesis of lysosomal organelles complex-1, subunit 2 | 1.263 | 2.29E-02 |
| BLVRA | Biliverdin reductase A | -1.31 | 1.20E-04 |
| BLVRB | Biliverdin reductase B | 1.409 | 1.24E-04 |
| BLZF1 | Basic leucine zipper nuclear factor 1 | -1.304 | 1.36E-03 |
| BMP1 | Bone morphogenetic protein 1 | 2.622 | 1.11E-12 |
| BMPR1B | Bone morphogenetic protein receptor, type IB | -1.324 | 1.85E-02 |
| BNIP2 | BCL2/adenovirus E1B 19kDa interacting protein 2 | -1.444 | 1.96E-03 |
| BNIP3 | BCL2/adenovirus E1B 19kDa interacting protein 3 | -1.34 | 1.67E-03 |
| BOC | BOC cell adhesion associated, oncogene regulated | -1.64 | 3.35E-04 |
| BOK | BCL2-related ovarian killer | 1.459 | 2.30E-03 |
| BRD1 | Bromodomain containing 1 | -1.292 | 7.94E-03 |
| BRD3 | Bromodomain containing 3 | -1.429 | 1.08E-04 |
| BROX | BRO1 domain and CAAX motif containing | -1.392 | 6.70E-03 |
| BTBD11 | BTB (POZ) domain containing 11 | 1.573 | 1.81E-02 |
| BTBD19 | BTB (POZ) domain containing 19 | -1.411 | 4.27E-03 |
| BTBD6 | BTB (POZ) domain containing 6 | 1.26 | 3.44E-03 |
| BTBD7 | BTB (POZ) domain containing 7 | -1.683 | 3.24E-04 |
| BTD | Biotinidase | -1.408 | 1.37E-02 |
| BTG1 | B-cell translocation gene 1, anti-proliferative | -1.73 | 7.12E-05 |
| BTG2 | BTG family, member 2 | 2.143 | 4.49E-07 |
| BYSL | Bystin-like | 1.356 | 3.15E-03 |
| C10orf10 | Chromosome 10 open reading frame 10 | -2.099 | 4.59E-09 |
| C10orf107 | Chromosome 10 open reading frame 107 | -2.421 | 4.14E-12 |
| C10orf71 | Chromosome 10 open reading frame 71 | -1.423 | 7.39E-06 |
| C11orf49 | Chromosome 11 open reading frame 49 | 1.387 | 5.29E-03 |
| C11orf54 | Chromosome 11 open reading frame 54 | 1.48 | 2.12E-03 |
| C14orf142 | Chromosome 14 open reading frame 142 | -1.281 | 5.80E-03 |
| C14orf93 | Chromosome 14 open reading frame 93 | -1.821 | 4.85E-07 |
| C15orf27 | Chromosome 15 open reading frame 27 | -1.366 | 5.91E-04 |
| C15orf39 | Chromosome 15 open reading frame 39 | 1.663 | 9.24E-04 |
| C17orf59 | Chromosome 17 open reading frame 59 | -1.359 | 1.39E-04 |
| C1orf110 | Chromosome 1 open reading frame 110 | -1.823 | 1.36E-04 |
| C1orf50 | Chromosome 1 open reading frame 50 | 1.254 | 1.82E-03 |
| C1orf54 | Chromosome 1 open reading frame 54 | 1.457 | 2.00E-03 |
| C1QB | Complement component 1, q subcomponent, B chain | 1.826 | 3.25E-04 |
| C1QC | Complement component 1, q subcomponent, C chain | 1.723 | 2.46E-03 |
| C1QTNF1 | C1q and tumor necrosis factor related protein 1 | 1.331 | 3.40E-03 |
| C1QTNF3 | C1q and tumor necrosis factor related protein 3 | 1.825 | 8.06E-08 |
| C1QTNF5 | C1q and tumor necrosis factor related protein 5 | 1.978 | 5.00E-06 |
| C1QTNF6 | C1q and tumor necrosis factor related protein 6 | 2.779 | 2.58E-09 |
| C1QTNF9B | C1q and tumor necrosis factor related protein 9B | -1.362 | 4.90E-04 |
| C2CD2 | C2 calcium-dependent domain containing 2 | -1.352 | 1.85E-02 |
| C2orf76 | Chromosome 2 open reading frame 76 | 1.413 | 9.63E-03 |
| C3orf18 | Chromosome 3 open reading frame 18 | -1.342 | 2.45E-03 |
| C3orf62 | Chromosome 3 open reading frame 62 | -1.337 | 1.26E-02 |
| C4orf46 | Chromosome 4 open reading frame 46 | 1.31 | 1.26E-02 |
| C5AR2 | Complement component 5a receptor 2 | 1.737 | 8.02E-05 |
| C5orf28 | Chromosome 5 open reading frame 28 | 1.448 | 2.00E-02 |
| C7orf60 | Chromosome 7 open reading frame 60 | -1.317 | 2.79E-02 |
| C8orf33 | Chromosome 8 open reading frame 33 | 1.505 | 5.82E-06 |
| C9orf16 | Chromosome 9 open reading frame 16 | 1.319 | 1.84E-03 |
| C9orf40 | Chromosome 9 open reading frame 40 | 1.3 | 9.54E-04 |
| C9orf72 | Chromosome 9 open reading frame 72 | 1.373 | 1.29E-02 |
| CA11 | Carbonic anhydrase XI | 1.89 | 2.48E-05 |
| CA14 | Carbonic anhydrase XIV | -1.51 | 5.67E-05 |
| CA3 | Carbonic anhydrase III | 1.398 | 2.13E-03 |
| CA4 | Carbonic anhydrase IV | -1.42 | 7.00E-03 |
| CABIN1 | Calcineurin binding protein 1 | -1.263 | 6.20E-03 |
| CACNA1S | Calcium channel, voltage-dependent, L type, alpha 1S subunit | -1.372 | 5.21E-04 |
| CADM1 | Cell adhesion molecule 1 | -1.274 | 4.33E-03 |
| CALCOCO1 | Calcium binding and coiled-coil domain 1 | -1.798 | 3.29E-10 |
| CALR | Calreticulin | 1.381 | 1.34E-06 |
| CALU | Calumenin | 1.351 | 9.27E-04 |
| CAMTA1 | Calmodulin binding transcription activator 1 | -1.789 | 4.31E-05 |
| CANX | Calnexin | 1.254 | 1.77E-03 |
| CAP1 | CAP, adenylate cyclase-associated protein 1 (yeast) | 1.379 | 5.80E-05 |
| CAPN6 | Calpain 6 | 1.794 | 2.19E-07 |
| CAPRIN2 | Caprin family member 2 | -1.442 | 7.02E-03 |
| CARF | Calcium responsive transcription factor | -1.409 | 1.11E-02 |
| CARNMT1 | Carnosine N-methyltransferase 1 | 1.353 | 8.51E-03 |
| CARNS1 | Carnosine synthase 1 | 1.637 | 2.11E-10 |
| CARS | Cysteinyl-tRNA synthetase | 1.379 | 1.55E-02 |
| CASP3 | Caspase 3, apoptosis-related cysteine peptidase | 1.577 | 2.05E-03 |
| CASP4 | Caspase 4, apoptosis-related cysteine peptidase | 1.632 | 3.89E-04 |
| CASP8 | Caspase 8, apoptosis-related cysteine peptidase | 1.708 | 7.36E-05 |
| CASQ2 | Calsequestrin 2 (cardiac muscle) | 2.197 | 9.25E-07 |
| CAST | Calpastatin | 1.258 | 1.26E-02 |
| CBWD1 | COBW domain containing 1 | 1.281 | 9.09E-04 |
| CBX7 | Chromobox homolog 7 | -1.721 | 1.15E-06 |
| CCDC107 | Coiled-coil domain containing 107 | -1.323 | 4.72E-03 |
| CCDC117 | Coiled-coil domain containing 117 | 1.452 | 4.32E-05 |
| CCDC13 | Coiled-coil domain containing 13 | 1.371 | 2.47E-02 |
| CCDC130 | Coiled-coil domain containing 130 | -1.297 | 2.81E-03 |
| CCDC14 | Coiled-coil domain containing 14 | -1.453 | 1.36E-02 |
| CCDC28A | Coiled-coil domain containing 28A | -1.314 | 5.06E-03 |
| CCDC51 | Coiled-coil domain containing 51 | 1.285 | 1.59E-02 |
| CCDC57 | Coiled-coil domain containing 57 | -1.666 | 1.06E-03 |
| CCDC77 | Coiled-coil domain containing 77 | -1.612 | 3.59E-04 |
| CCDC80 | Coiled-coil domain containing 80 | 1.776 | 5.38E-11 |
| CCDC86 | Coiled-coil domain containing 86 | 2.148 | 1.01E-16 |
| CCDC88A | Coiled-coil domain containing 88A | 1.481 | 6.36E-03 |
| CCHCR1 | Coiled-coil alpha-helical rod protein 1 | -1.316 | 2.01E-02 |
| CCL2 | Chemokine (C-C motif) ligand 2 | 2.465 | 3.09E-04 |
| CCND1 | Cyclin D1 | 3.758 | 2.33E-11 |
| CCNDBP1 | Cyclin D-type binding-protein 1 | -1.393 | 5.17E-05 |
| CCNE2 | Cyclin E2 | 1.627 | 1.67E-02 |
| CCNG2 | Cyclin G2 | -1.316 | 2.38E-02 |
| CCNYL1 | Cyclin Y-like 1 | -1.671 | 4.84E-09 |
| CCRL2 | Chemokine (C-C motif) receptor-like 2 | -2.417 | 8.45E-11 |
| CCSER2 | Coiled-coil serine-rich protein 2 | -1.308 | 1.09E-04 |
| CCT2 | Chaperonin containing TCP1, subunit 2 (beta) | 1.277 | 1.09E-02 |
| CCT3 | Chaperonin containing TCP1, subunit 3 (gamma) | 1.435 | 9.21E-09 |
| CCT4 | Chaperonin containing TCP1, subunit 4 (delta) | 1.292 | 7.83E-03 |
| CCT5 | Chaperonin containing TCP1, subunit 5 (epsilon) | 1.434 | 2.78E-09 |
| CCT6A | Chaperonin containing TCP1, subunit 6A (zeta 1) | 1.538 | 3.75E-08 |
| CCT7 | Chaperonin containing TCP1, subunit 7 (eta) | 1.289 | 8.20E-05 |
| CCT8 | Chaperonin containing TCP1, subunit 8 (theta) | 1.277 | 1.91E-03 |
| CD109 | CD109 molecule | -1.515 | 1.80E-02 |
| CD248 | CD248 molecule, endosialin | 1.452 | 6.16E-03 |
| CD44 | CD44 molecule (Indian blood group) | 2.146 | 6.04E-08 |
| CD93 | CD93 molecule | 1.866 | 3.42E-09 |
| CDC14A | Cell division cycle 14A | 1.451 | 1.19E-02 |
| CDC26 | Cell division cycle 26 | 2.054 | 3.63E-12 |
| CDC34 | Cell division cycle 34 | 1.293 | 5.74E-03 |
| CDC42EP1 | CDC42 effector protein (Rho GTPase binding) 1 | 1.311 | 7.19E-03 |
| CDC42EP2 | CDC42 effector protein (Rho GTPase binding) 2 | 2.091 | 7.26E-08 |
| CDC42EP3 | CDC42 effector protein (Rho GTPase binding) 3 | 1.395 | 2.39E-04 |
| CDC42SE1 | CDC42 small effector 1 | 1.323 | 1.13E-04 |
| CDC7 | Cell division cycle 7 | 1.648 | 8.62E-04 |
| CDC73 | Cell division cycle 73 | -1.336 | 2.42E-06 |
| CDH11 | Cadherin 11, type 2, OB-cadherin (osteoblast) | 1.307 | 1.96E-02 |
| CDH13 | Cadherin 13 | 1.289 | 3.40E-04 |
| CDH22 | Cadherin 22, type 2 | -3.359 | 2.78E-15 |
| CDH23 | Cadherin-related 23 | 1.334 | 1.88E-02 |
| CDH4 | Cadherin 4, type 1, R-cadherin (retinal) | -1.448 | 4.65E-03 |
| CDIP1 | Cell death-inducing p53 target 1 | -1.436 | 4.18E-05 |
| CDK16 | Cyclin-dependent kinase 16 | 1.273 | 2.77E-04 |
| CDK19 | Cyclin-dependent kinase 19 | -1.603 | 2.23E-04 |
| CDK20 | Cyclin-dependent kinase 20 | -1.507 | 5.21E-05 |
| CDK2AP2 | Cyclin-dependent kinase 2 associated protein 2 | 1.253 | 2.73E-02 |
| CDK5RAP2 | CDK5 regulatory subunit associated protein 2 | -1.284 | 1.10E-03 |
| CDK7 | Cyclin-dependent kinase 7 | 1.273 | 1.31E-02 |
| CDKN1A | Cyclin-dependent kinase inhibitor 1A (p21, Cip1) | 1.843 | 5.14E-04 |
| CDKN1B | Cyclin-dependent kinase inhibitor 1B (p27, Kip1) | -1.324 | 8.84E-05 |
| CDKN2AIPNL | CDKN2A interacting protein N-terminal like | -1.325 | 1.78E-04 |
| CDNF | Cerebral dopamine neurotrophic factor | 1.382 | 2.90E-03 |
| CDON | Cell adhesion associated, oncogene regulated | -1.54 | 1.39E-05 |
| CDR2L | Cerebellar degeneration-related protein 2-like | 1.463 | 2.24E-02 |
| CEBPG | CCAAT/enhancer binding protein (C/EBP), gamma | 1.35 | 2.19E-03 |
| CENPU | Centromere protein U | -1.405 | 2.57E-02 |
| CEP131 | Centrosomal protein 131kDa | -1.369 | 1.12E-02 |
| CEP350 | Centrosomal protein 350kDa | -1.398 | 1.86E-02 |
| CEP83 | Centrosomal protein 83kDa | 1.702 | 2.09E-04 |
| CEP85 | Centrosomal protein 85kDa | -1.281 | 3.25E-04 |
| CEP85L | Centrosomal protein 85kDa-like | -2.047 | 1.49E-03 |
| CEP97 | Centrosomal protein 97kDa | -1.791 | 8.18E-04 |
| CERCAM | Cerebral endothelial cell adhesion molecule | 1.406 | 5.54E-03 |
| CERS1 | Ceramide synthase 1 | -1.398 | 1.11E-04 |
| CES1 | Carboxylesterase 1 | -1.86 | 4.48E-04 |
| CFL1 | Cofilin 1 (non-muscle) | 1.578 | 7.13E-09 |
| CFL2 | Cofilin 2 (muscle) | 1.423 | 6.54E-03 |
| CHCHD4 | Coiled-coil-helix-coiled-coil-helix domain containing 4 | 1.321 | 1.79E-03 |
| CHD3 | Chromodomain helicase DNA binding protein 3 | 1.374 | 6.36E-05 |
| CHD9 | Chromodomain helicase DNA binding protein 9 | -1.285 | 9.05E-03 |
| CHODL | Chondrolectin | 1.641 | 1.83E-04 |
| CHORDC1 | Cysteine and histidine-rich domain (CHORD) containing 1 | 1.941 | 4.66E-16 |
| CHP1 | Calcineurin-like EF-hand protein 1 | 1.639 | 9.17E-10 |
| CHRDL2 | Chordin-like 2 | 1.561 | 2.72E-02 |
| CHRNB1 | Cholinergic receptor, nicotinic, beta 1 (muscle) | 1.319 | 3.76E-05 |
| CHRNE | Cholinergic receptor, nicotinic, epsilon (muscle) | -1.685 | 3.65E-04 |
| CHST15 | Carbohydrate (N-acetylgalactosamine 4-sulfate 6-O) sulfotransferase 15 | 1.901 | 6.51E-05 |
| CHST2 | Carbohydrate (N-acetylglucosamine-6-O) sulfotransferase 2 | 1.425 | 4.00E-03 |
| CHURC1 | Churchill domain containing 1 | 1.332 | 5.00E-03 |
| CIB1 | Calcium and integrin binding 1 (calmyrin) | 1.341 | 4.11E-03 |
| CIB2 | Calcium and integrin binding family member 2 | 1.429 | 1.05E-02 |
| CIITA | Class II, major histocompatibility complex, transactivator | -1.777 | 2.04E-05 |
| CILP | Cartilage intermediate layer protein, nucleotide pyrophosphohydrolase | 2.917 | 7.41E-14 |
| CIPC | CLOCK-interacting pacemaker | -1.294 | 6.32E-03 |
| CIRBP | Cold inducible RNA binding protein | -3.095 | 1.08E-27 |
| CISD1 | CDGSH iron sulfur domain 1 | 1.343 | 8.12E-03 |
| CISD3 | CDGSH iron sulfur domain 3 | 1.348 | 1.10E-02 |
| CITED1 | Cbp/p300-interacting transactivator, with Glu/Asp-rich carboxy-terminal domain, 1 | -1.753 | 1.77E-04 |
| CKAP4 | Cytoskeleton-associated protein 4 | 1.337 | 3.99E-03 |
| CKB | Creatine kinase, brain | 1.658 | 2.35E-02 |
| CLASP1 | Cytoplasmic linker associated protein 1 | -1.266 | 2.86E-04 |
| CLCN6 | Chloride channel, voltage-sensitive 6 | -1.338 | 4.10E-03 |
| CLCN7 | Chloride channel, voltage-sensitive 7 | -1.251 | 1.70E-02 |
| CLEC1A | C-type lectin domain family 1, member A | -1.496 | 1.08E-02 |
| CLEC3B | C-type lectin domain family 3, member B | -1.346 | 3.05E-05 |
| CLIC4 | Chloride intracellular channel 4 | 2.096 | 9.99E-13 |
| CLIP2 | CAP-GLY domain containing linker protein 2 | 1.524 | 6.35E-03 |
| CLPTM1 | Cleft lip and palate associated transmembrane protein 1 | -1.376 | 1.85E-05 |
| CMBL | Carboxymethylenebutenolidase homolog (Pseudomonas) | -1.487 | 8.68E-06 |
| CMC4 | C-x(9)-C motif containing 4 | -1.551 | 1.20E-05 |
| CMPK1 | Cytidine monophosphate (UMP-CMP) kinase 1, cytosolic | 1.322 | 9.69E-03 |
| CMYA5 | Cardiomyopathy associated 5 | -1.708 | 4.83E-06 |
| CNGA3 | Cyclic nucleotide gated channel alpha 3 | -1.511 | 1.13E-02 |
| CNKSR1 | Connector enhancer of kinase suppressor of Ras 1 | 1.471 | 1.35E-04 |
| CNKSR3 | CNKSR family member 3 | 1.282 | 1.63E-02 |
| CNN1 | Calponin 1, basic, smooth muscle | 2.032 | 2.23E-07 |
| CNN2 | Calponin 2 | 1.376 | 2.17E-03 |
| CNN3 | Calponin 3, acidic | 1.461 | 2.77E-05 |
| CNNM4 | Cyclin and CBS domain divalent metal cation transport mediator 4 | 1.47 | 2.63E-03 |
| CNOT10 | CCR4-NOT transcription complex, subunit 10 | -1.349 | 6.88E-04 |
| CNP | 2',3'-cyclic nucleotide 3' phosphodiesterase | 1.609 | 1.02E-06 |
| CNPPD1 | Cyclin Pas1/PHO80 domain containing 1 | -1.343 | 3.09E-03 |
| CNPY4 | Canopy FGF signaling regulator 4 | 1.292 | 1.66E-03 |
| CNST | Consortin, connexin sorting protein | -1.409 | 1.87E-04 |
| COA3 | Cytochrome c oxidase assembly factor 3 | 1.366 | 4.16E-05 |
| COBL | Cordon-bleu WH2 repeat protein | -1.372 | 3.86E-07 |
| COG1 | Component of oligomeric golgi complex 1 | -1.312 | 3.65E-04 |
| COL15A1 | Collagen, type XV, alpha 1 | 1.607 | 4.01E-08 |
| COL1A1 | Collagen, type I, alpha 1 | 1.445 | 5.49E-03 |
| COL1A2 | Collagen, type I, alpha 2 | 1.639 | 2.52E-06 |
| COL4A1 | Collagen, type IV, alpha 1 | 2.363 | 3.93E-11 |
| COL4A2 | Collagen, type IV, alpha 2 | 2.034 | 4.22E-08 |
| COL4A3BP | Collagen, type IV, alpha 3 (Goodpasture antigen) binding protein | -1.825 | 5.09E-09 |
| COL5A3 | Collagen, type V, alpha 3 | 1.683 | 1.88E-06 |
| COL6A1 | Collagen, type VI, alpha 1 | 1.32 | 3.13E-03 |
| COL6A3 | Collagen, type VI, alpha 3 | 1.638 | 6.89E-10 |
| COL6A6 | Collagen, type VI, alpha 6 | 1.846 | 1.35E-04 |
| COL8A1 | Collagen, type VIII, alpha 1 | 1.787 | 8.37E-04 |
| COL8A2 | Collagen, type VIII, alpha 2 | 1.547 | 2.07E-02 |
| COLGALT1 | Collagen beta(1-O)galactosyltransferase 1 | 1.512 | 6.63E-06 |
| COMMD7 | COMM domain containing 7 | 1.367 | 6.35E-05 |
| COMT | Catechol-O-methyltransferase | -1.312 | 2.82E-02 |
| COPG2 | Coatomer protein complex, subunit gamma 2 | -1.381 | 1.46E-04 |
| COPRS | Coordinator of PRMT5, differentiation stimulator | -1.265 | 6.38E-03 |
| Cops2 | COP9 (constitutive photomorphogenic) homolog, subunit 2 (Arabidopsis thaliana) | 1.436 | 1.05E-03 |
| COQ6 | Coenzyme Q6 monooxygenase | 1.477 | 1.62E-04 |
| COQ7 | Coenzyme Q7 homolog, ubiquinone (yeast) | 1.287 | 2.55E-03 |
| CORO1A | Coronin, actin binding protein, 1A | 1.655 | 8.65E-04 |
| CORO1B | Coronin, actin binding protein, 1B | 1.344 | 1.16E-03 |
| COX15 | Cytochrome c oxidase assembly homolog 15 (yeast) | 1.321 | 6.30E-04 |
| COX5B | Cytochrome c oxidase subunit Vb | -1.298 | 4.37E-04 |
| CPE | Carboxypeptidase E | 1.432 | 2.68E-02 |
| CPEB2 | Cytoplasmic polyadenylation element binding protein 2 | -2.144 | 3.92E-09 |
| CPEB4 | Cytoplasmic polyadenylation element binding protein 4 | -1.294 | 6.44E-03 |
| CPED1 | Cadherin-like and PC-esterase domain containing 1 | 1.515 | 9.39E-05 |
| CPNE8 | Copine VIII | -1.467 | 2.48E-02 |
| CPSF6 | Cleavage and polyadenylation specific factor 6, 68kDa | -1.444 | 2.72E-07 |
| CPT1A | Carnitine palmitoyltransferase 1A (liver) | 2.355 | 7.04E-07 |
| CPT2 | Carnitine palmitoyltransferase 2 | 1.327 | 4.22E-04 |
| CPXM1 | Carboxypeptidase X (M14 family), member 1 | 1.511 | 2.03E-03 |
| CRABP2 | Cellular retinoic acid binding protein 2 | 1.582 | 8.87E-03 |
| CREB3L1 | cAMP responsive element binding protein 3-like 1 | 1.335 | 1.75E-02 |
| CREBBP | CREB binding protein | -1.258 | 1.07E-03 |
| CREBRF | CREB3 regulatory factor | -2.774 | 7.20E-07 |
| CRELD1 | Cysteine-rich with EGF-like domains 1 | 1.346 | 2.30E-05 |
| CRELD2 | Cysteine-rich with EGF-like domains 2 | 1.531 | 3.58E-03 |
| CRIM1 | Cysteine rich transmembrane BMP regulator 1 (chordin-like) | -1.375 | 1.25E-03 |
| CRIP1 | Cysteine-rich protein 1 (intestinal) | 1.407 | 1.21E-02 |
| CRISPLD2 | Cysteine-rich secretory protein LCCL domain containing 2 | 1.357 | 1.28E-04 |
| CRY2 | Cryptochrome circadian clock 2 | -1.274 | 1.70E-02 |
| CRYAB | Crystallin, alpha B | 2.539 | 1.14E-11 |
| CRYZL1 | Crystallin, zeta (quinone reductase)-like 1 | -1.438 | 2.20E-04 |
| CSAD | Cysteine sulfinic acid decarboxylase | -1.319 | 1.78E-03 |
| CSTF2 | Cleavage stimulation factor, 3' pre-RNA, subunit 2, 64kDa | 1.437 | 1.09E-03 |
| CSTF3 | Cleavage stimulation factor, 3' pre-RNA, subunit 3, 77kDa | -1.324 | 2.28E-03 |
| CTCF | CCCTC-binding factor (zinc finger protein) | -1.331 | 1.25E-05 |
| CTDSP2 | CTD (carboxy-terminal domain, RNA polymerase II, polypeptide A) small phosphatase 2 | -1.347 | 8.50E-06 |
| CTGF | Connective tissue growth factor | 1.568 | 3.77E-03 |
| CTNNA3 | Catenin (cadherin-associated protein), alpha 3 | -1.555 | 1.76E-04 |
| CTNNBIP1 | Catenin, beta interacting protein 1 | -1.554 | 7.05E-04 |
| CTNNBL1 | Catenin, beta like 1 | -1.438 | 4.88E-07 |
| CTPS1 | CTP synthase 1 | 2.675 | 2.22E-17 |
| CTSD | Cathepsin D | -1.266 | 2.00E-02 |
| CTSF | Cathepsin F | -1.743 | 3.91E-11 |
| CTSH | Cathepsin H | 1.367 | 3.79E-03 |
| CTSK | Cathepsin K | 1.643 | 2.09E-08 |
| CTSS | Cathepsin S | 1.375 | 1.17E-02 |
| CXCR4 | Chemokine (C-X-C motif) receptor 4 | 1.755 | 1.01E-05 |
| CXorf36 | Chromosome X open reading frame 36 | 1.266 | 6.69E-03 |
| CYB561D2 | Cytochrome b561 family, member D2 | 1.418 | 1.87E-03 |
| CYB5B | Cytochrome b5 type B (outer mitochondrial membrane) | 1.333 | 1.70E-04 |
| CYB5R1 | Cytochrome b5 reductase 1 | -1.329 | 1.87E-04 |
| CYB5R4 | Cytochrome b5 reductase 4 | 1.515 | 1.30E-05 |
| CYP1A1 | Cytochrome P450, family 1, subfamily A, polypeptide 1 | -1.908 | 2.95E-05 |
| CYP20A1 | Cytochrome P450, family 20, subfamily A, polypeptide 1 | -1.387 | 6.06E-04 |
| CYP26B1 | Cytochrome P450, family 26, subfamily B, polypeptide 1 | -1.39 | 2.68E-02 |
| CYP51A1 | Cytochrome P450, family 51, subfamily A, polypeptide 1 | 1.859 | 3.69E-05 |
| CYTH3 | Cytohesin 3 | -1.456 | 1.59E-04 |
| D2HGDH | D-2-hydroxyglutarate dehydrogenase | -1.31 | 4.79E-04 |
| DAAM1 | Dishevelled associated activator of morphogenesis 1 | -1.251 | 1.86E-02 |
| DAB2 | Dab, mitogen-responsive phosphoprotein, homolog 2 (Drosophila) | 1.478 | 1.57E-04 |
| DAGLA | Diacylglycerol lipase, alpha | 1.313 | 1.29E-02 |
| DAP3 | Death associated protein 3 | 1.264 | 1.18E-03 |
| DARS2 | Aspartyl-tRNA synthetase 2, mitochondrial | 1.558 | 2.44E-05 |
| DBI | Diazepam binding inhibitor (GABA receptor modulator, acyl-CoA binding protein) | 1.677 | 6.04E-09 |
| DCAF11 | DDB1 and CUL4 associated factor 11 | -1.38 | 1.12E-07 |
| DCAF13 | DDB1 and CUL4 associated factor 13 | 1.289 | 2.21E-03 |
| DCAF17 | DDB1 and CUL4 associated factor 17 | -1.312 | 3.63E-03 |
| DCAKD | Dephospho-CoA kinase domain containing | 1.428 | 1.84E-03 |
| DCHS1 | Dachsous cadherin-related 1 | 1.331 | 5.98E-03 |
| DCLK3 | Doublecortin-like kinase 3 | -1.623 | 2.40E-02 |
| DCLRE1A | DNA cross-link repair 1A | -1.345 | 1.82E-02 |
| DCN | Decorin | 1.317 | 1.62E-02 |
| DCTN1 | Dynactin 1 | -1.286 | 4.87E-04 |
| DDC | Dopa decarboxylase (aromatic L-amino acid decarboxylase) | 1.77 | 6.84E-04 |
| DDIT3 | DNA-damage-inducible transcript 3 | 1.58 | 4.57E-05 |
| DDIT4L | DNA-damage-inducible transcript 4-like | -1.766 | 9.40E-06 |
| DDO | D-aspartate oxidase | -1.478 | 3.28E-04 |
| DDX10 | DEAD (Asp-Glu-Ala-Asp) box polypeptide 10 | 1.355 | 2.87E-02 |
| DDX11 | DEAD/H (Asp-Glu-Ala-Asp/His) box helicase 11 | 1.409 | 1.74E-02 |
| DDX18 | DEAD (Asp-Glu-Ala-Asp) box polypeptide 18 | -1.333 | 6.26E-03 |
| DDX20 | DEAD (Asp-Glu-Ala-Asp) box polypeptide 20 | -1.355 | 6.77E-04 |
| DDX28 | DEAD (Asp-Glu-Ala-Asp) box polypeptide 28 | -1.394 | 4.51E-05 |
| DDX3Y | DEAD (Asp-Glu-Ala-Asp) box helicase 3, Y-linked | -1.353 | 3.98E-04 |
| DDX42 | DEAD (Asp-Glu-Ala-Asp) box helicase 42 | -1.331 | 5.04E-06 |
| DDX51 | DEAD (Asp-Glu-Ala-Asp) box polypeptide 51 | 1.291 | 2.03E-02 |
| DDX54 | DEAD (Asp-Glu-Ala-Asp) box polypeptide 54 | 1.276 | 1.77E-02 |
| DEGS1 | Delta(4)-desaturase, sphingolipid 1 | 1.449 | 4.97E-03 |
| DENND5B | DENN/MADD domain containing 5B | -2.603 | 3.78E-04 |
| DERL1 | Derlin 1 | 1.253 | 9.77E-03 |
| DES | Desmin | -1.363 | 1.90E-04 |
| DESI1 | Desumoylating isopeptidase 1 | 1.331 | 4.95E-03 |
| DEXI | Dexi homolog (mouse) | 1.279 | 4.63E-03 |
| DFFA | DNA fragmentation factor, 45kDa, alpha polypeptide | 1.464 | 4.23E-05 |
| DGKQ | Diacylglycerol kinase, theta 110kDa | -1.301 | 1.77E-02 |
| DHCR24 | 24-dehydrocholesterol reductase | 1.475 | 3.66E-03 |
| DHDH | Dihydrodiol dehydrogenase (dimeric) | 1.434 | 1.84E-04 |
| DHTKD1 | Dehydrogenase E1 and transketolase domain containing 1 | 1.277 | 5.38E-04 |
| DHX38 | DEAH (Asp-Glu-Ala-His) box polypeptide 38 | -1.365 | 9.84E-04 |
| DHX57 | DEAH (Asp-Glu-Ala-Asp/His) box polypeptide 57 | -1.287 | 5.31E-03 |
| DIMT1 | DIM1 dimethyladenosine transferase 1 homolog | 1.318 | 1.15E-03 |
| DIRAS1 | DIRAS family, GTP-binding RAS-like 1 | -1.449 | 5.68E-03 |
| DKK2 | Dickkopf WNT signaling pathway inhibitor 2 | 1.94 | 2.36E-05 |
| DLG4 | Discs, large homolog 4 (Drosophila) | 1.832 | 6.37E-04 |
| DMPK | Dystrophia myotonica-protein kinase | 1.49 | 1.74E-03 |
| DNAJA1 | DnaJ (Hsp40) homolog, subfamily A, member 1 | 2.022 | 7.13E-11 |
| DNAJA4 | DnaJ (Hsp40) homolog, subfamily A, member 4 | 1.889 | 2.87E-09 |
| DNAJB1 | DnaJ (Hsp40) homolog, subfamily B, member 1 | 1.324 | 3.13E-04 |
| DNAJB11 | DnaJ (Hsp40) homolog, subfamily B, member 11 | 1.332 | 1.70E-04 |
| DNAJB12 | DnaJ (Hsp40) homolog, subfamily B, member 12 | -1.269 | 3.11E-03 |
| DNAJB4 | DnaJ (Hsp40) homolog, subfamily B, member 4 | 1.634 | 2.86E-06 |
| DNAJC12 | DnaJ (Hsp40) homolog, subfamily C, member 12 | 1.394 | 2.13E-02 |
| DNAJC21 | DnaJ (Hsp40) homolog, subfamily C, member 21 | 1.316 | 4.56E-05 |
| DNAJC24 | DnaJ (Hsp40) homolog, subfamily C, member 24 | 1.486 | 1.78E-03 |
| DNAJC3 | DnaJ (Hsp40) homolog, subfamily C, member 3 | -1.273 | 1.46E-02 |
| DNAJC4 | DnaJ (Hsp40) homolog, subfamily C, member 4 | -1.297 | 2.89E-03 |
| DNASE2 | Deoxyribonuclease II, lysosomal | 1.309 | 2.01E-02 |
| DNM1L | Dynamin 1-like | 1.349 | 5.56E-04 |
| DOCK4 | Dedicator of cytokinesis 4 | -1.346 | 7.71E-03 |
| DOCK9 | Dedicator of cytokinesis 9 | -1.468 | 1.69E-05 |
| DOPEY2 | Dopey family member 2 | -1.314 | 3.31E-03 |
| DPAGT1 | Dolichyl-phosphate (UDP-N-acetylglucosamine) N-acetylglucosaminephosphotransferase 1 (GlcNAc-1-P transferase) | 1.347 | 2.80E-04 |
| DPF3 | D4, zinc and double PHD fingers, family 3 | 1.451 | 6.27E-04 |
| DPM2 | Dolichyl-phosphate mannosyltransferase polypeptide 2, regulatory subunit | 1.306 | 3.11E-03 |
| DPP8 | Dipeptidyl-peptidase 8 | -1.38 | 2.07E-03 |
| DPT | Dermatopontin | 1.804 | 2.65E-06 |
| DPY19L1 | Dpy-19-like 1 (C. elegans) | 1.522 | 8.31E-03 |
| DPYSL3 | Dihydropyrimidinase-like 3 | 3.097 | 1.29E-11 |
| DSE | Dermatan sulfate epimerase | 1.327 | 2.62E-02 |
| DSG4 | Desmoglein 4 | -2.18 | 3.03E-05 |
| DST | Dystonin | -1.26 | 3.35E-03 |
| DTD1 | D-tyrosyl-tRNA deacylase 1 | -1.393 | 6.04E-05 |
| DTNBP1 | Dystrobrevin binding protein 1 | -1.259 | 3.97E-03 |
| DUS1L | Dihydrouridine synthase 1-like | 1.317 | 1.34E-03 |
| DUSP1 | Dual specificity phosphatase 1 | 1.522 | 5.80E-05 |
| DUSP13 | Dual specificity phosphatase 13 | 1.404 | 1.62E-04 |
| DUSP14 | Dual specificity phosphatase 14 | -1.373 | 1.22E-03 |
| DUSP16 | Dual specificity phosphatase 16 | 1.515 | 2.16E-04 |
| DUSP26 | Dual specificity phosphatase 26 (putative) | -1.926 | 3.14E-09 |
| DUSP6 | Dual specificity phosphatase 6 | 1.326 | 1.11E-02 |
| DYNC1LI2 | Dynein, cytoplasmic 1, light intermediate chain 2 | 1.42 | 1.19E-06 |
| DYNLL1 | Dynein, light chain, LC8-type 1 | 2.302 | 4.31E-10 |
| DYRK1A | Dual-specificity tyrosine-(Y)-phosphorylation regulated kinase 1A | -1.25 | 4.54E-03 |
| DYRK1B | Dual-specificity tyrosine-(Y)-phosphorylation regulated kinase 1B | 1.354 | 7.75E-04 |
| DYRK2 | Dual-specificity tyrosine-(Y)-phosphorylation regulated kinase 2 | -1.649 | 1.45E-09 |
| DYRK3 | Dual-specificity tyrosine-(Y)-phosphorylation regulated kinase 3 | 1.815 | 2.74E-04 |
| E2F3 | E2F transcription factor 3 | 1.372 | 1.52E-02 |
| EAPP | E2F-associated phosphoprotein | -1.405 | 9.13E-04 |
| EBAG9 | Estrogen receptor binding site associated, antigen, 9 | 1.297 | 1.41E-02 |
| EBNA1BP2 | EBNA1 binding protein 2 | 1.45 | 2.23E-05 |
| ECE2 | Endothelin converting enzyme 2 | 1.442 | 1.86E-04 |
| ECHDC3 | Enoyl CoA hydratase domain containing 3 | -1.478 | 4.95E-03 |
| ECHS1 | Enoyl CoA hydratase, short chain, 1, mitochondrial | -1.289 | 4.78E-04 |
| ECI2 | Enoyl-CoA delta isomerase 2 | -1.257 | 3.26E-03 |
| ECM1 | Extracellular matrix protein 1 | 1.451 | 2.67E-03 |
| ECM2 | Extracellular matrix protein 2, female organ and adipocyte specific | 1.612 | 1.06E-05 |
| EDC4 | Enhancer of mRNA decapping 4 | -1.266 | 2.15E-04 |
| EDEM3 | ER degradation enhancer, mannosidase alpha-like 3 | -2.275 | 8.01E-05 |
| EEF1B2 | Eukaryotic translation elongation factor 1 beta 2 | 1.312 | 4.83E-04 |
| EFHD2 | EF-hand domain family, member D2 | 1.394 | 4.36E-03 |
| EHBP1L1 | EH domain binding protein 1-like 1 | 1.377 | 3.65E-05 |
| EHD3 | EH-domain containing 3 | 1.598 | 7.23E-05 |
| EIF2B1 | Eukaryotic translation initiation factor 2B, subunit 1 alpha, 26kDa | 1.403 | 3.12E-05 |
| EIF2D | Eukaryotic translation initiation factor 2D | 1.487 | 3.54E-04 |
| EIF3B | Eukaryotic translation initiation factor 3, subunit B | -1.253 | 1.47E-03 |
| EIF3C | Eukaryotic translation initiation factor 3, subunit C | -1.287 | 6.89E-05 |
| EIF3M | Eukaryotic translation initiation factor 3, subunit M | -1.332 | 1.62E-02 |
| EIF4A1 | Eukaryotic translation initiation factor 4A1 | 1.314 | 5.07E-05 |
| EIF4B | Eukaryotic translation initiation factor 4B | -1.636 | 3.48E-10 |
| EIF4E | Eukaryotic translation initiation factor 4E | 1.28 | 1.32E-02 |
| EIF4E3 | Eukaryotic translation initiation factor 4E family member 3 | -1.698 | 4.17E-08 |
| EIF4EBP1 | Eukaryotic translation initiation factor 4E binding protein 1 | -2.755 | 2.19E-15 |
| ELL2 | Elongation factor, RNA polymerase II, 2 | 1.376 | 2.01E-03 |
| ELMSAN1 | ELM2 and Myb/SANT-like domain containing 1 | -1.354 | 6.37E-04 |
| ELOVL6 | ELOVL fatty acid elongase 6 | 2.976 | 2.41E-04 |
| EMD | Emerin | 1.258 | 1.38E-02 |
| EMILIN1 | Elastin microfibril interfacer 1 | 1.418 | 4.36E-04 |
| EMP3 | Epithelial membrane protein 3 | 1.301 | 7.61E-03 |
| ENC1 | Ectodermal-neural cortex 1 (with BTB domain) | 1.363 | 4.31E-03 |
| ENO1 | Enolase 1, (alpha) | 1.517 | 1.68E-07 |
| ENO3 | Enolase 3 (beta, muscle) | -1.262 | 4.80E-03 |
| ENSA | Endosulfine alpha | 1.321 | 6.02E-06 |
| ENTPD5 | Ectonucleoside triphosphate diphosphohydrolase 5 | 1.327 | 1.38E-02 |
| EPB41L3 | Erythrocyte membrane protein band 4.1-like 3 | 1.438 | 1.32E-03 |
| EPC1 | Enhancer of polycomb homolog 1 (Drosophila) | -1.314 | 4.18E-05 |
| EPG5 | Ectopic P-granules autophagy protein 5 homolog (C. elegans) | -1.321 | 1.86E-02 |
| EPHX2 | Epoxide hydrolase 2, cytoplasmic | -1.599 | 4.12E-04 |
| EPS8 | Epidermal growth factor receptor pathway substrate 8 | -1.29 | 4.82E-03 |
| ERCC1 | Excision repair cross-complementation group 1 | 1.309 | 1.16E-02 |
| ERCC6 | Excision repair cross-complementation group 6 | -1.438 | 1.45E-03 |
| ERMP1 | Endoplasmic reticulum metallopeptidase 1 | 1.359 | 2.58E-02 |
| ESCO1 | Establishment of sister chromatid cohesion N-acetyltransferase 1 | 1.565 | 3.34E-03 |
| ESR1 | Estrogen receptor 1 | -1.315 | 2.09E-03 |
| ETS1 | V-ets avian erythroblastosis virus E26 oncogene homolog 1 | 1.469 | 4.56E-04 |
| EXOC2 | Exocyst complex component 2 | 1.274 | 1.32E-02 |
| EXOSC10 | Exosome component 10 | -1.307 | 2.94E-05 |
| EXTL1 | Exostosin-like glycosyltransferase 1 | 3.669 | 1.48E-16 |
| EYA3 | EYA transcriptional coactivator and phosphatase 3 | 1.329 | 1.58E-03 |
| EYA4 | EYA transcriptional coactivator and phosphatase 4 | -1.533 | 9.99E-05 |
| EZH1 | Enhancer of zeste 1 polycomb repressive complex 2 subunit | -1.477 | 6.99E-09 |
| EZR | Ezrin | -1.302 | 5.81E-03 |
| F13A1 | Coagulation factor XIII, A1 polypeptide | 1.61 | 7.25E-03 |
| F2R | Coagulation factor II (thrombin) receptor | 1.582 | 7.95E-04 |
| FABP3 | Fatty acid binding protein 3, muscle and heart | 1.889 | 7.36E-10 |
| FADS3 | Fatty acid desaturase 3 | 2.349 | 5.96E-11 |
| FAH | Fumarylacetoacetate hydrolase (fumarylacetoacetase) | -3.353 | 2.01E-15 |
| FAM101B | Family with sequence similarity 101, member B | 2.69 | 4.50E-12 |
| FAM109A | Family with sequence similarity 109, member A | 1.822 | 2.60E-05 |
| FAM120A | Family with sequence similarity 120A | 1.401 | 1.09E-06 |
| FAM124B | Family with sequence similarity 124B | 1.935 | 1.82E-04 |
| FAM131A | Family with sequence similarity 131, member A | 1.273 | 1.91E-03 |
| FAM134B | Family with sequence similarity 134, member B | -1.738 | 1.35E-04 |
| FAM135A | Family with sequence similarity 135, member A | -1.413 | 5.72E-03 |
| Fam13a | Family with sequence similarity 13, member A | -1.648 | 2.21E-05 |
| FAM13B | Family with sequence similarity 13, member B | -1.77 | 1.94E-03 |
| FAM160B1 | Family with sequence similarity 160, member B1 | -1.332 | 2.73E-02 |
| FAM171A2 | Family with sequence similarity 171, member A2 | -1.475 | 6.57E-04 |
| FAM173A | Family with sequence similarity 173, member A | 1.819 | 4.59E-06 |
| FAM177A1 | Family with sequence similarity 177, member A1 | 1.405 | 5.04E-03 |
| FAM184A | Family with sequence similarity 184, member A | -1.477 | 1.93E-02 |
| Fam184b | Family with sequence similarity 184, member B | -1.788 | 1.01E-07 |
| FAM188A | Family with sequence similarity 188, member A | 1.364 | 3.59E-03 |
| FAM189A2 | Family with sequence similarity 189, member A2 | -1.266 | 1.04E-02 |
| FAM46C | Family with sequence similarity 46, member C | 1.706 | 6.97E-05 |
| FAM53C | Family with sequence similarity 53, member C | -1.443 | 1.69E-07 |
| FAM57A | Family with sequence similarity 57, member A | 1.485 | 4.03E-03 |
| FAM63A | Family with sequence similarity 63, member A | -1.304 | 8.50E-04 |
| FAM69A | Family with sequence similarity 69, member A | -1.296 | 2.28E-02 |
| FAM71E1 | Family with sequence similarity 71, member E1 | 1.443 | 7.66E-03 |
| FAM78A | Family with sequence similarity 78, member A | -1.29 | 4.03E-03 |
| FAM83H | Family with sequence similarity 83, member H | -1.382 | 2.56E-02 |
| FAM8A1 | Family with sequence similarity 8, member A1 | -1.253 | 2.91E-03 |
| FAM92A1 | Family with sequence similarity 92, member A1 | 1.42 | 4.11E-03 |
| FANCC | Fanconi anemia, complementation group C | 1.293 | 2.03E-02 |
| FANCM | Fanconi anemia, complementation group M | 1.455 | 1.64E-02 |
| FAR2 | Fatty acyl CoA reductase 2 | 2.202 | 4.01E-06 |
| FARSA | Phenylalanyl-tRNA synthetase, alpha subunit | 1.291 | 1.73E-02 |
| FBL | Fibrillarin | 1.29 | 4.16E-03 |
| FBLN1 | Fibulin 1 | 1.431 | 8.78E-04 |
| FBN1 | Fibrillin 1 | 1.38 | 3.06E-03 |
| FBXL14 | F-box and leucine-rich repeat protein 14 | 1.313 | 1.15E-02 |
| Fbxl22 | F-box and leucine-rich repeat protein 22 | 1.489 | 7.89E-05 |
| FBXL4 | F-box and leucine-rich repeat protein 4 | -1.475 | 1.65E-04 |
| FBXO10 | F-box protein 10 | -2.423 | 4.07E-08 |
| FBXO15 | F-box protein 15 | 1.404 | 7.61E-03 |
| FBXO25 | F-box protein 25 | -1.391 | 1.01E-04 |
| FBXO32 | F-box protein 32 | -2.101 | 4.88E-07 |
| FBXO45 | F-box protein 45 | -1.275 | 2.31E-02 |
| FBXO6 | F-box protein 6 | -1.294 | 3.12E-03 |
| FBXW8 | F-box and WD repeat domain containing 8 | -1.271 | 1.40E-03 |
| FCGRT | Fc fragment of IgG, receptor, transporter, alpha | 1.791 | 2.83E-10 |
| FDFT1 | Farnesyl-diphosphate farnesyltransferase 1 | -1.697 | 1.60E-08 |
| FECH | Ferrochelatase | -1.377 | 1.70E-06 |
| FES | FES proto-oncogene, tyrosine kinase | 1.502 | 4.69E-03 |
| FGFR4 | Fibroblast growth factor receptor 4 | 1.468 | 1.96E-03 |
| FGL2 | Fibrinogen-like 2 | 1.482 | 1.14E-04 |
| FHL3 | Four and a half LIM domains 3 | -1.309 | 1.20E-04 |
| FHOD3 | Formin homology 2 domain containing 3 | 1.534 | 8.02E-04 |
| FILIP1 | Filamin A interacting protein 1 | -1.46 | 2.64E-03 |
| FILIP1L | Filamin A interacting protein 1-like | -1.274 | 8.78E-03 |
| FKBP10 | FK506 binding protein 10, 65 kDa | 2.556 | 8.30E-14 |
| FKBP14 | FK506 binding protein 14, 22 kDa | 1.909 | 9.14E-08 |
| FKBP3 | FK506 binding protein 3, 25kDa | 1.27 | 2.42E-02 |
| FKBP5 | FK506 binding protein 5 | -1.465 | 7.22E-03 |
| FKBP7 | FK506 binding protein 7 | 1.891 | 2.89E-07 |
| FKBP9 | FK506 binding protein 9, 63 kDa | 1.279 | 1.76E-02 |
| FKRP | Fukutin related protein | 1.273 | 4.76E-03 |
| FLAD1 | Flavin adenine dinucleotide synthetase 1 | 1.285 | 8.38E-03 |
| FLNA | Filamin A, alpha | 1.276 | 4.04E-03 |
| FLOT2 | Flotillin 2 | -1.286 | 6.78E-04 |
| FLT1 | Fms-related tyrosine kinase 1 | -1.388 | 1.08E-04 |
| FMOD | Fibromodulin | 1.314 | 9.16E-04 |
| FMR1 | Fragile X mental retardation 1 | -1.459 | 9.26E-03 |
| Fnbp1l | Formin binding protein 1-like | -1.327 | 1.39E-02 |
| FNDC3A | Fibronectin type III domain containing 3A | -1.283 | 2.01E-02 |
| FNIP1 | Folliculin interacting protein 1 | -1.87 | 3.33E-06 |
| FNTB | Farnesyltransferase, CAAX box, beta | -1.97 | 2.00E-07 |
| FOS | FBJ murine osteosarcoma viral oncogene homolog | 3.122 | 2.16E-03 |
| FOXJ2 | Forkhead box J2 | -1.273 | 8.20E-05 |
| FOXN2 | Forkhead box N2 | -1.464 | 5.43E-04 |
| Foxn3 | Forkhead box N3 | -1.433 | 9.19E-06 |
| FOXO1 | Forkhead box O1 | -1.937 | 1.47E-03 |
| FOXO3 | Forkhead box O3 | -1.674 | 1.32E-06 |
| FOXO4 | Forkhead box O4 | -1.554 | 1.43E-12 |
| FOXS1 | Forkhead box S1 | -2.044 | 4.74E-09 |
| FRS3 | Fibroblast growth factor receptor substrate 3 | -1.352 | 1.95E-02 |
| FRY | Furry homolog (Drosophila) | -1.297 | 3.49E-04 |
| FRYL | FRY-like | -1.294 | 2.33E-02 |
| FSCN1 | Fascin actin-bundling protein 1 | 2.164 | 4.51E-09 |
| FSD1L | Fibronectin type III and SPRY domain containing 1-like | -1.936 | 2.81E-03 |
| FST | Follistatin | 1.412 | 2.24E-02 |
| FSTL1 | Follistatin-like 1 | 1.366 | 3.85E-04 |
| FTSJ3 | FtsJ homolog 3 (E. coli) | 1.368 | 3.00E-04 |
| FUBP1 | Far upstream element (FUSE) binding protein 1 | 1.388 | 2.80E-03 |
| FUBP3 | Far upstream element (FUSE) binding protein 3 | -1.298 | 5.01E-04 |
| FUNDC2 | FUN14 domain containing 2 | -1.293 | 1.67E-04 |
| FURIN | Furin (paired basic amino acid cleaving enzyme) | -1.278 | 5.50E-03 |
| FXYD5 | FXYD domain containing ion transport regulator 5 | 1.299 | 1.96E-02 |
| FZD7 | Frizzled class receptor 7 | -1.296 | 1.19E-03 |
| GAB2 | GRB2-associated binding protein 2 | -1.588 | 3.54E-05 |
| GABARAP | GABA(A) receptor-associated protein | -1.269 | 1.05E-03 |
| GABBR1 | Gamma-aminobutyric acid (GABA) B receptor, 1 | -1.36 | 5.26E-03 |
| GABPA | GA binding protein transcription factor, alpha subunit 60kDa | -1.291 | 9.48E-03 |
| GABPB2 | GA binding protein transcription factor, beta subunit 2 | -1.569 | 8.02E-04 |
| GADD45B | Growth arrest and DNA-damage-inducible, beta | -1.611 | 6.59E-04 |
| GADL1 | Glutamate decarboxylase-like 1 | 1.301 | 2.76E-02 |
| GAK | Cyclin G associated kinase | -1.357 | 1.28E-03 |
| GALC | Galactosylceramidase | -1.319 | 1.41E-02 |
| GALK1 | Galactokinase 1 | -1.559 | 6.55E-04 |
| GALM | Galactose mutarotase (aldose 1-epimerase) | 1.395 | 9.17E-04 |
| GALNT10 | Polypeptide N-acetylgalactosaminyltransferase 10 | 1.368 | 1.83E-02 |
| GALNT16 | Polypeptide N-acetylgalactosaminyltransferase 16 | 1.496 | 3.47E-04 |
| GALNT18 | Polypeptide N-acetylgalactosaminyltransferase 18 | 1.35 | 2.22E-02 |
| GAMT | Guanidinoacetate N-methyltransferase | 1.284 | 7.21E-03 |
| GAPDH | Glyceraldehyde-3-phosphate dehydrogenase | -1.275 | 1.54E-03 |
| GARS | Glycyl-tRNA synthetase | 1.784 | 2.44E-06 |
| GAS1 | Growth arrest-specific 1 | -1.783 | 1.91E-04 |
| GAS2L1 | Growth arrest-specific 2 like 1 | 1.741 | 3.75E-04 |
| GAS7 | Growth arrest-specific 7 | 2.15 | 1.26E-04 |
| GATA2 | GATA binding protein 2 | -1.611 | 1.13E-03 |
| GATAD1 | GATA zinc finger domain containing 1 | -1.289 | 2.21E-02 |
| GATAD2B | GATA zinc finger domain containing 2B | -1.272 | 2.21E-03 |
| GATSL2 | GATS protein-like 2 | -1.826 | 1.09E-09 |
| GBE1 | Glucan (1,4-alpha-), branching enzyme 1 | 1.274 | 2.48E-02 |
| GCNT1 | Glucosaminyl (N-acetyl) transferase 1, core 2 | -1.768 | 3.28E-05 |
| GDAP2 | Ganglioside induced differentiation associated protein 2 | 1.56 | 5.29E-04 |
| GDF11 | Growth differentiation factor 11 | 1.685 | 2.61E-04 |
| GEM | GTP binding protein overexpressed in skeletal muscle | 1.703 | 2.84E-03 |
| GEMIN7 | Gem (nuclear organelle) associated protein 7 | 1.292 | 2.04E-03 |
| GFPT2 | Glutamine-fructose-6-phosphate transaminase 2 | -1.43 | 1.77E-02 |
| GID4 | GID complex subunit 4 homolog | 1.279 | 4.32E-04 |
| GJA1 | Gap junction protein, alpha 1, 43kDa | 1.728 | 1.35E-04 |
| GKAP1 | G kinase anchoring protein 1 | 1.261 | 2.48E-02 |
| GLB1 | Galactosidase, beta 1 | -1.3 | 1.52E-02 |
| GLCE | Glucuronic acid epimerase | -1.361 | 2.42E-02 |
| GLIPR2 | GLI pathogenesis-related 2 | 1.82 | 7.45E-05 |
| GLRX | Glutaredoxin (thioltransferase) | 1.48 | 4.61E-06 |
| GLYCTK | Glycerate kinase | 1.293 | 2.33E-02 |
| GM2A | GM2 ganglioside activator | 1.44 | 3.28E-03 |
| GMEB2 | Glucocorticoid modulatory element binding protein 2 | -1.262 | 1.49E-02 |
| GMNN | Geminin, DNA replication inhibitor | 1.551 | 1.27E-03 |
| GMPPA | GDP-mannose pyrophosphorylase A | 1.325 | 2.36E-04 |
| GMPPB | GDP-mannose pyrophosphorylase B | 1.403 | 4.09E-03 |
| GNAI2 | Guanine nucleotide binding protein (G protein), alpha inhibiting activity polypeptide 2 | 1.391 | 5.48E-05 |
| GNAI3 | Guanine nucleotide binding protein (G protein), alpha inhibiting activity polypeptide 3 | 1.486 | 1.56E-04 |
| GNAQ | Guanine nucleotide binding protein (G protein), q polypeptide | -1.756 | 9.02E-06 |
| GNE | Glucosamine (UDP-N-acetyl)-2-epimerase/N-acetylmannosamine kinase | -1.424 | 1.69E-04 |
| GNG2 | Guanine nucleotide binding protein (G protein), gamma 2 | 1.777 | 3.78E-04 |
| GNL2 | Guanine nucleotide binding protein-like 2 (nucleolar) | -1.488 | 1.23E-06 |
| GNL3 | Guanine nucleotide binding protein-like 3 (nucleolar) | 1.43 | 7.33E-06 |
| GNPDA1 | Glucosamine-6-phosphate deaminase 1 | 1.317 | 1.11E-03 |
| GPAM | Glycerol-3-phosphate acyltransferase, mitochondrial | 2.079 | 7.94E-09 |
| GPATCH3 | G patch domain containing 3 | -1.27 | 8.80E-03 |
| GPATCH8 | G patch domain containing 8 | -1.329 | 2.26E-03 |
| GPC1 | Glypican 1 | 1.712 | 1.90E-09 |
| GPD2 | Glycerol-3-phosphate dehydrogenase 2 (mitochondrial) | -1.798 | 1.12E-07 |
| GPN3 | GPN-loop GTPase 3 | 1.339 | 3.76E-03 |
| GPR137 | G protein-coupled receptor 137 | 1.386 | 1.41E-03 |
| GPT | Glutamic-pyruvate transaminase (alanine aminotransferase) | 1.408 | 3.78E-04 |
| GPT2 | Glutamic pyruvate transaminase (alanine aminotransferase) 2 | -1.351 | 2.87E-03 |
| GPX7 | Glutathione peroxidase 7 | 1.323 | 9.77E-03 |
| GRAMD1B | GRAM domain containing 1B | -1.729 | 2.44E-04 |
| GRB10 | Growth factor receptor-bound protein 10 | -1.875 | 1.94E-16 |
| GRIP2 | Glutamate receptor interacting protein 2 | 1.3 | 1.18E-02 |
| GRWD1 | Glutamate-rich WD repeat containing 1 | 1.427 | 3.69E-05 |
| GSR | Glutathione reductase | 1.546 | 2.05E-05 |
| GSS | Glutathione synthetase | 1.284 | 1.90E-02 |
| GSTK1 | Glutathione S-transferase kappa 1 | 1.604 | 5.55E-08 |
| GTDC1 | Glycosyltransferase-like domain containing 1 | 1.283 | 8.65E-03 |
| GTF2H3 | General transcription factor IIH, polypeptide 3, 34kDa | 1.327 | 4.88E-03 |
| GTF2IRD1 | GTF2I repeat domain containing 1 | 1.334 | 1.28E-02 |
| Gtf2ird2 | GTF2I repeat domain containing 2 | -1.409 | 2.38E-04 |
| GUK1 | Guanylate kinase 1 | 1.384 | 4.63E-04 |
| GUSB | Glucuronidase, beta | 1.423 | 8.55E-04 |
| H1FX | H1 histone family, member X | -1.442 | 1.14E-05 |
| H2AFJ | H2A histone family, member J | -1.56 | 2.80E-06 |
| H2AFX | H2A histone family, member X | 1.663 | 2.14E-04 |
| H2AFZ | H2A histone family, member Z | 1.441 | 9.82E-04 |
| HABP4 | Hyaluronan binding protein 4 | 1.743 | 5.72E-08 |
| HACD1 | 3-hydroxyacyl-CoA dehydratase 1 | 1.473 | 3.75E-06 |
| HACD3 | 3-hydroxyacyl-CoA dehydratase 3 | 1.261 | 1.06E-02 |
| HAGHL | Hydroxyacylglutathione hydrolase-like | 1.461 | 2.83E-05 |
| HAPLN3 | Hyaluronan and proteoglycan link protein 3 | 1.437 | 4.64E-03 |
| HBD | Hemoglobin, delta | 159.75 | 1.31E-15 |
| HBP1 | HMG-box transcription factor 1 | -1.543 | 1.06E-06 |
| HCFC1R1 | Host cell factor C1 regulator 1 (XPO1 dependent) | 1.371 | 1.59E-02 |
| HCLS1 | Hematopoietic cell-specific Lyn substrate 1 | 1.446 | 1.38E-02 |
| HCN2 | Hyperpolarization activated cyclic nucleotide gated potassium channel 2 | -1.345 | 2.09E-03 |
| HDAC10 | Histone deacetylase 10 | 1.667 | 1.76E-03 |
| HDAC4 | Histone deacetylase 4 | -1.64 | 3.53E-06 |
| HDAC6 | Histone deacetylase 6 | -1.253 | 6.67E-04 |
| HDAC9 | Histone deacetylase 9 | -1.395 | 2.35E-04 |
| HDGFRP2 | Hepatoma-derived growth factor-related protein 2 | -1.266 | 1.17E-03 |
| HECTD1 | HECT domain containing E3 ubiquitin protein ligase 1 | -1.25 | 5.56E-03 |
| HERC2 | HECT and RLD domain containing E3 ubiquitin protein ligase 2 | 1.251 | 4.78E-03 |
| HERC3 | HECT and RLD domain containing E3 ubiquitin protein ligase 3 | -1.314 | 2.22E-03 |
| HERC4 | HECT and RLD domain containing E3 ubiquitin protein ligase 4 | 1.407 | 2.27E-03 |
| HES2 | Hes family bHLH transcription factor 2 | 1.555 | 5.51E-04 |
| HEXDC | Hexosaminidase (glycosyl hydrolase family 20, catalytic domain) containing | 1.55 | 3.98E-06 |
| HEY2 | Hes-related family bHLH transcription factor with YRPW motif 2 | -1.356 | 1.87E-02 |
| HEYL | Hes-related family bHLH transcription factor with YRPW motif-like | 1.467 | 6.76E-04 |
| HFE2 | Hemochromatosis type 2 (juvenile) | -1.376 | 9.71E-06 |
| HGH1 | HGH1 homolog | 1.748 | 3.60E-07 |
| HIGD1A | HIG1 hypoxia inducible domain family, member 1A | 1.354 | 4.99E-03 |
| HIPK3 | Homeodomain interacting protein kinase 3 | -1.586 | 2.79E-03 |
| HIST1H2AC | Histone cluster 1, H2ac | 2.036 | 3.14E-04 |
| HIST1H2BD | Histone cluster 1, H2bd | 1.336 | 3.61E-04 |
| HK2 | Hexokinase 2 | -1.494 | 3.72E-04 |
| HMGCR | 3-hydroxy-3-methylglutaryl-CoA reductase | 1.523 | 8.09E-04 |
| HMGCS1 | 3-hydroxy-3-methylglutaryl-CoA synthase 1 (soluble) | 1.252 | 2.77E-02 |
| HNRNPDL | Heterogeneous nuclear ribonucleoprotein D-like | -1.358 | 6.17E-04 |
| HNRNPH1 | Heterogeneous nuclear ribonucleoprotein H1 (H) | -1.278 | 1.06E-03 |
| HNRNPH2 | Heterogeneous nuclear ribonucleoprotein H2 (H') | -1.276 | 1.21E-04 |
| HNRNPUL2 | Heterogeneous nuclear ribonucleoprotein U-like 2 | -1.252 | 6.55E-04 |
| HOMER1 | Homer scaffolding protein 1 | 1.251 | 2.41E-02 |
| HOMER3 | Homer scaffolding protein 3 | -1.391 | 5.13E-05 |
| HOOK3 | Hook microtubule-tethering protein 3 | -2.286 | 9.31E-04 |
| HOXA3 | Homeobox A3 | 1.271 | 2.80E-02 |
| HOXA4 | Homeobox A4 | 1.724 | 9.83E-05 |
| HOXB4 | Homeobox B4 | 1.639 | 4.68E-05 |
| HOXC5 | Homeobox C5 | 1.542 | 3.63E-04 |
| HOXC8 | Homeobox C8 | -1.261 | 2.91E-03 |
| HPCAL1 | Hippocalcin-like 1 | 1.466 | 2.09E-04 |
| HPDL | 4-hydroxyphenylpyruvate dioxygenase-like | 2.197 | 4.24E-06 |
| HR | Hair growth associated | 2.241 | 7.49E-06 |
| HRASLS | HRAS-like suppressor | -1.347 | 1.73E-04 |
| HS1BP3 | HCLS1 binding protein 3 | 1.575 | 1.42E-03 |
| HSD17B12 | Hydroxysteroid (17-beta) dehydrogenase 12 | 1.586 | 5.22E-05 |
| HSD17B7 | Hydroxysteroid (17-beta) dehydrogenase 7 | 1.531 | 1.53E-03 |
| HSD17B8 | Hydroxysteroid (17-beta) dehydrogenase 8 | 1.528 | 3.58E-08 |
| HSF4 | Heat shock transcription factor 4 | -1.332 | 9.75E-03 |
| HSP90AA1 | Heat shock protein 90kDa alpha (cytosolic), class A member 1 | 1.282 | 6.91E-04 |
| HSP90AB1 | Heat shock protein 90kDa alpha (cytosolic), class B member 1 | 1.292 | 7.82E-03 |
| HSP90B1 | Heat shock protein 90kDa beta (Grp94), member 1 | 1.307 | 3.24E-04 |
| HSPA12A | Heat shock 70kDa protein 12A | -1.386 | 9.06E-03 |
| HSPA4 | Heat shock 70kDa protein 4 | 1.364 | 2.53E-03 |
| HSPA5 | Heat shock 70kDa protein 5 (glucose-regulated protein, 78kDa) | 1.426 | 7.39E-06 |
| HSPA8 | Heat shock 70kDa protein 8 | 1.529 | 2.58E-08 |
| HSPB1 | Heat shock 27kDa protein 1 | 1.808 | 1.51E-04 |
| HSPB8 | Heat shock 22kDa protein 8 | 1.56 | 7.20E-12 |
| HSPBP1 | HSPA (heat shock 70kDa) binding protein, cytoplasmic cochaperone 1 | 1.312 | 1.74E-02 |
| HSPD1 | Heat shock 60kDa protein 1 (chaperonin) | 1.342 | 1.39E-03 |
| HSPE1 | Heat shock 10kDa protein 1 | 1.301 | 4.77E-04 |
| HSPG2 | Heparan sulfate proteoglycan 2 | 1.329 | 1.10E-03 |
| HSPH1 | Heat shock 105kDa/110kDa protein 1 | 2.413 | 4.42E-13 |
| HYAL2 | Hyaluronoglucosaminidase 2 | 1.622 | 1.59E-06 |
| HYOU1 | Hypoxia up-regulated 1 | 1.322 | 2.61E-03 |
| IARS | Isoleucyl-tRNA synthetase | 1.418 | 1.41E-03 |
| IBA57 | IBA57 homolog, iron-sulfur cluster assembly | 1.698 | 6.85E-07 |
| ICA1 | Islet cell autoantigen 1, 69kDa | 1.613 | 2.71E-04 |
| ICAM3 | Intercellular adhesion molecule 3 | 1.604 | 1.74E-04 |
| ID2 | Inhibitor of DNA binding 2, dominant negative helix-loop-helix protein | 1.392 | 9.28E-03 |
| IDH3B | Isocitrate dehydrogenase 3 (NAD+) beta | -1.308 | 3.90E-05 |
| IDI1 | Isopentenyl-diphosphate delta isomerase 1 | 1.255 | 4.47E-03 |
| IER2 | Immediate early response 2 | 1.417 | 1.37E-03 |
| IFRD1 | Interferon-related developmental regulator 1 | 1.72 | 1.01E-05 |
| IFRD2 | Interferon-related developmental regulator 2 | 1.321 | 3.04E-03 |
| IFT140 | Intraflagellar transport 140 | 1.413 | 5.93E-03 |
| IFT172 | Intraflagellar transport 172 | 1.342 | 1.03E-02 |
| IFT20 | Intraflagellar transport 20 | 1.353 | 7.34E-03 |
| IGDCC4 | Immunoglobulin superfamily, DCC subclass, member 4 | 1.517 | 4.15E-05 |
| IGF1R | Insulin-like growth factor 1 receptor | -1.567 | 8.52E-06 |
| IGF2R | Insulin-like growth factor 2 receptor | -1.438 | 2.63E-06 |
| IGFBP4 | Insulin-like growth factor binding protein 4 | 1.377 | 4.00E-05 |
| IGFBP5 | Insulin-like growth factor binding protein 5 | 1.489 | 1.71E-04 |
| IGFBP7 | Insulin-like growth factor binding protein 7 | 1.93 | 9.66E-12 |
| IGSF3 | Immunoglobulin superfamily, member 3 | 1.383 | 1.69E-02 |
| IGSF8 | Immunoglobulin superfamily, member 8 | 1.854 | 1.40E-06 |
| IKBKAP | Inhibitor of kappa light polypeptide gene enhancer in B-cells, kinase complex-associated protein | -1.264 | 2.97E-03 |
| IKBKG | Inhibitor of kappa light polypeptide gene enhancer in B-cells, kinase gamma | 1.441 | 1.97E-04 |
| IL17RC | Interleukin 17 receptor C | -1.282 | 2.78E-02 |
| IL1B | Interleukin 1, beta | -1.525 | 5.53E-03 |
| IL27RA | Interleukin 27 receptor, alpha | 1.505 | 4.18E-03 |
| IL2RG | Interleukin 2 receptor, gamma | 1.66 | 7.65E-04 |
| IL34 | Interleukin 34 | 1.343 | 2.79E-02 |
| IL4R | Interleukin 4 receptor | -1.291 | 1.43E-03 |
| IL6ST | Interleukin 6 signal transducer | -1.853 | 1.11E-03 |
| IMPA2 | Inositol(myo)-1(or 4)-monophosphatase 2 | -1.671 | 6.45E-04 |
| IMPDH1 | IMP (inosine 5'-monophosphate) dehydrogenase 1 | 1.698 | 1.24E-05 |
| INADL | InaD-like (Drosophila) | -1.384 | 4.74E-03 |
| INCA1 | Inhibitor of CDK, cyclin A1 interacting protein 1 | -1.399 | 5.53E-04 |
| ING2 | Inhibitor of growth family, member 2 | 1.479 | 1.17E-04 |
| ING5 | Inhibitor of growth family, member 5 | -1.464 | 3.22E-04 |
| INHA | Inhibin, alpha | 2.979 | 6.31E-10 |
| INO80B | INO80 complex subunit B | -1.522 | 1.91E-05 |
| INPP4B | Inositol polyphosphate-4-phosphatase, type II, 105kDa | 1.439 | 1.22E-02 |
| INPP5K | Inositol polyphosphate-5-phosphatase K | -1.385 | 8.51E-07 |
| INSR | Insulin receptor | -1.403 | 1.46E-05 |
| INTS2 | Integrator complex subunit 2 | 1.412 | 2.45E-02 |
| INTS6 | Integrator complex subunit 6 | 1.257 | 9.78E-03 |
| INTS7 | Integrator complex subunit 7 | 1.372 | 9.63E-04 |
| IRAK1 | Interleukin-1 receptor-associated kinase 1 | 1.446 | 1.51E-03 |
| IRF2BPL | Interferon regulatory factor 2 binding protein-like | 1.38 | 3.67E-03 |
| IRF3 | Interferon regulatory factor 3 | 1.286 | 2.60E-02 |
| IRF9 | Interferon regulatory factor 9 | -1.321 | 2.67E-03 |
| IRS2 | Insulin receptor substrate 2 | -2.32 | 4.56E-09 |
| ITGA5 | Integrin, alpha 5 (fibronectin receptor, alpha polypeptide) | 1.786 | 1.08E-07 |
| ITGA6 | Integrin, alpha 6 | 1.298 | 6.51E-03 |
| ITGA7 | Integrin, alpha 7 | 1.36 | 1.15E-03 |
| ITGAV | Integrin, alpha V | -2.043 | 5.88E-04 |
| ITGB2 | Integrin, beta 2 (complement component 3 receptor 3 and 4 subunit) | 1.498 | 2.61E-02 |
| ITM2B | Integral membrane protein 2B | -1.255 | 1.20E-03 |
| ITPRIP | Inositol 1,4,5-trisphosphate receptor interacting protein | -1.603 | 4.24E-08 |
| JADE2 | Jade family PHD finger 2 | -2.862 | 4.20E-13 |
| JCHAIN | Joining chain of multimeric IgA and IgM | 1.604 | 1.45E-04 |
| JDP2 | Jun dimerization protein 2 | 1.417 | 7.16E-03 |
| JKAMP | JNK1/MAPK8-associated membrane protein | 1.404 | 1.78E-02 |
| JUN | Jun proto-oncogene | 1.309 | 3.52E-03 |
| KANK4 | KN motif and ankyrin repeat domains 4 | -1.565 | 2.17E-04 |
| KANSL1L | KAT8 regulatory NSL complex subunit 1-like | -1.384 | 3.93E-03 |
| KAT2A | K(lysine) acetyltransferase 2A | 1.457 | 4.75E-04 |
| KAT2B | K(lysine) acetyltransferase 2B | -2.011 | 2.51E-23 |
| KAT6A | K(lysine) acetyltransferase 6A | -1.368 | 4.23E-05 |
| KAT8 | K(lysine) acetyltransferase 8 | -1.399 | 8.87E-06 |
| KATNAL1 | Katanin p60 subunit A-like 1 | -1.761 | 3.54E-08 |
| KBTBD13 | Kelch repeat and BTB (POZ) domain containing 13 | -1.517 | 1.69E-03 |
| KBTBD2 | Kelch repeat and BTB (POZ) domain containing 2 | 1.487 | 1.20E-06 |
| KCNA5 | Potassium channel, voltage gated shaker related subfamily A, member 5 | 1.563 | 1.05E-02 |
| KCNB1 | Potassium channel, voltage gated Shab related subfamily B, member 1 | 1.814 | 1.31E-05 |
| KCNG2 | Potassium channel, voltage gated modifier subfamily G, member 2 | -2.636 | 2.96E-08 |
| KCNJ8 | Potassium channel, inwardly rectifying subfamily J, member 8 | 1.647 | 3.31E-06 |
| KCNMA1 | Potassium channel, calcium activated large conductance subfamily M alpha, member 1 | 1.6 | 1.99E-06 |
| KCNMB4 | Potassium channel subfamily M regulatory beta subunit 4 | -1.467 | 1.90E-03 |
| KCNQ4 | Potassium channel, voltage gated KQT-like subfamily Q, member 4 | 1.529 | 1.98E-03 |
| KCNQ5 | Potassium channel, voltage gated KQT-like subfamily Q, member 5 | 1.604 | 1.70E-06 |
| KCNS3 | Potassium voltage-gated channel, modifier subfamily S, member 3 | -2.567 | 2.58E-19 |
| KCP | Kielin/chordin-like protein | 1.524 | 7.48E-03 |
| KCTD11 | Potassium channel tetramerization domain containing 11 | 1.454 | 6.78E-06 |
| KCTD15 | Potassium channel tetramerization domain containing 15 | 1.419 | 1.88E-02 |
| KCTD2 | Potassium channel tetramerization domain containing 2 | -1.288 | 1.15E-04 |
| KCTD21 | Potassium channel tetramerization domain containing 21 | 1.433 | 7.93E-03 |
| KCTD3 | Potassium channel tetramerization domain containing 3 | 1.253 | 1.32E-02 |
| KCTD5 | Potassium channel tetramerization domain containing 5 | -1.265 | 1.53E-03 |
| KDELC1 | KDEL (Lys-Asp-Glu-Leu) containing 1 | 1.748 | 2.16E-06 |
| KDELC2 | KDEL (Lys-Asp-Glu-Leu) containing 2 | 1.263 | 2.13E-02 |
| KDELR3 | KDEL (Lys-Asp-Glu-Leu) endoplasmic reticulum protein retention receptor 3 | 1.546 | 2.14E-04 |
| KDM3B | Lysine (K)-specific demethylase 3B | -1.413 | 1.11E-07 |
| KDM4B | Lysine (K)-specific demethylase 4B | -1.345 | 1.38E-04 |
| KDM5A | Lysine (K)-specific demethylase 5A | -1.629 | 1.20E-05 |
| KDM5C | Lysine (K)-specific demethylase 5C | 1.255 | 3.65E-03 |
| KDM7A | Lysine (K)-specific demethylase 7A | -1.906 | 1.24E-02 |
| KHNYN | KH and NYN domain containing | -1.253 | 5.12E-03 |
| KHSRP | KH-type splicing regulatory protein | -1.329 | 9.34E-03 |
| KIAA0020 | KIAA0020 | 1.437 | 2.25E-03 |
| KIAA0408 | KIAA0408 | -1.732 | 5.62E-06 |
| KIAA0430 | KIAA0430 | -1.456 | 2.02E-08 |
| KIAA0754 | KIAA0754 | 1.375 | 8.57E-03 |
| KIAA0922 | KIAA0922 | -1.31 | 4.36E-04 |
| KIAA1191 | KIAA1191 | 1.278 | 8.25E-03 |
| KIAA1211 | KIAA1211 | -1.68 | 4.84E-06 |
| KIAA1217 | KIAA1217 | -1.506 | 6.83E-07 |
| KIAA1671 | KIAA1671 | 1.478 | 3.77E-03 |
| KIAA2018 | KIAA2018 | -1.45 | 2.65E-04 |
| KIF13A | Kinesin family member 13A | -1.251 | 4.17E-04 |
| KIF1B | Kinesin family member 1B | -1.566 | 3.35E-04 |
| KIF21A | Kinesin family member 21A | 1.295 | 1.32E-02 |
| KIF3A | Kinesin family member 3A | -1.577 | 1.20E-04 |
| KIT | V-kit Hardy-Zuckerman 4 feline sarcoma viral oncogene homolog | 1.47 | 2.12E-02 |
| KITLG | KIT ligand | -1.691 | 1.71E-03 |
| KIZ | Kizuna centrosomal protein | -1.597 | 1.16E-06 |
| KLC4 | Kinesin light chain 4 | -1.25 | 2.75E-02 |
| KLF10 | Kruppel-like factor 10 | 1.506 | 1.05E-02 |
| KLF11 | Kruppel-like factor 11 | 1.612 | 1.22E-02 |
| KLF15 | Kruppel-like factor 15 | -1.985 | 1.36E-09 |
| KLF3 | Kruppel-like factor 3 (basic) | -1.355 | 4.57E-03 |
| KLF6 | Kruppel-like factor 6 | -1.288 | 4.21E-03 |
| KLF9 | Kruppel-like factor 9 | -1.352 | 2.82E-04 |
| KLHL17 | Kelch-like family member 17 | 2.287 | 9.46E-09 |
| KLHL2 | Kelch-like family member 2 | 1.307 | 2.12E-02 |
| KLHL22 | Kelch-like family member 22 | -1.507 | 7.01E-05 |
| KLHL24 | Kelch-like family member 24 | -2.189 | 2.00E-05 |
| KLHL25 | Kelch-like family member 25 | -1.347 | 5.22E-03 |
| KLHL26 | Kelch-like family member 26 | -1.292 | 3.59E-03 |
| KLHL28 | Kelch-like family member 28 | -1.994 | 1.71E-03 |
| KLHL33 | Kelch-like family member 33 | -1.568 | 3.19E-07 |
| KLHL38 | Kelch-like family member 38 | -1.331 | 1.87E-02 |
| KLHL41 | Kelch-like family member 41 | -1.341 | 3.04E-03 |
| KMT2A | Lysine (K)-specific methyltransferase 2A | -1.422 | 5.77E-03 |
| KPNA2 | Karyopherin alpha 2 (RAG cohort 1, importin alpha 1) | 1.726 | 5.18E-09 |
| KPTN | Kaptin (actin binding protein) | 1.513 | 9.74E-06 |
| KRBA1 | KRAB-A domain containing 1 | 1.741 | 7.21E-05 |
| KREMEN1 | Kringle containing transmembrane protein 1 | -1.467 | 1.33E-05 |
| KXD1 | KxDL motif containing 1 | -1.259 | 1.73E-03 |
| LACC1 | Laccase (multicopper oxidoreductase) domain containing 1 | 1.484 | 4.71E-03 |
| LAMA4 | Laminin, alpha 4 | 1.531 | 2.47E-06 |
| LAMB1 | Laminin, beta 1 | 1.376 | 2.28E-04 |
| LARP1B | La ribonucleoprotein domain family, member 1B | 1.72 | 3.52E-07 |
| LASP1 | LIM and SH3 protein 1 | 1.744 | 8.92E-11 |
| LBR | Lamin B receptor | -1.314 | 4.50E-03 |
| LDB1 | LIM domain binding 1 | -1.305 | 1.45E-02 |
| LDLRAD4 | Low density lipoprotein receptor class A domain containing 4 | 1.586 | 2.72E-04 |
| LFNG | LFNG O-fucosylpeptide 3-beta-N-acetylglucosaminyltransferase | 1.577 | 7.86E-05 |
| LGALS1 | Lectin, galactoside-binding, soluble, 1 | 1.521 | 5.86E-10 |
| LHFPL2 | Lipoma HMGIC fusion partner-like 2 | 1.486 | 4.98E-03 |
| LHPP | Phospholysine phosphohistidine inorganic pyrophosphate phosphatase | 1.349 | 9.99E-04 |
| LIFR | Leukemia inhibitory factor receptor alpha | -2.351 | 1.24E-04 |
| LIMA1 | LIM domain and actin binding 1 | 1.559 | 1.10E-04 |
| LIMD1 | LIM domains containing 1 | -1.325 | 1.56E-03 |
| LIMK2 | LIM domain kinase 2 | -1.369 | 1.07E-03 |
| LIMS1 | LIM and senescent cell antigen-like domains 1 | -1.392 | 1.30E-05 |
| LIMS2 | LIM and senescent cell antigen-like domains 2 | 1.583 | 1.23E-05 |
| LIN37 | Lin-37 DREAM MuvB core complex component | -1.46 | 1.37E-04 |
| LIN9 | Lin-9 DREAM MuvB core complex component | 1.598 | 2.55E-03 |
| LINS | Lines homolog (Drosophila) | 1.4 | 2.65E-02 |
| LIPE | Lipase, hormone-sensitive | -1.758 | 7.59E-03 |
| LIX1 | Limb and CNS expressed 1 | 1.795 | 9.02E-07 |
| LLGL2 | Lethal giant larvae homolog 2 (Drosophila) | -1.955 | 1.35E-04 |
| LMNB1 | Lamin B1 | 2.186 | 2.93E-07 |
| LMOD1 | Leiomodin 1 (smooth muscle) | -1.712 | 9.47E-07 |
| LMOD2 | Leiomodin 2 (cardiac) | -1.473 | 5.93E-04 |
| LNP1 | Leukemia NUP98 fusion partner 1 | -1.44 | 8.53E-03 |
| LNX1 | Ligand of numb-protein X 1, E3 ubiquitin protein ligase | -1.57 | 1.23E-04 |
| LOC81691 | Exonuclease NEF-sp | 1.279 | 2.72E-02 |
| LONP2 | lon peptidase 2, peroxisomal | -1.363 | 1.70E-06 |
| LOXL1 | Lysyl oxidase-like 1 | 1.728 | 2.26E-04 |
| LOXL2 | Lysyl oxidase-like 2 | 2.106 | 1.89E-10 |
| LPIN1 | Lipin 1 | -1.898 | 2.04E-11 |
| LPIN2 | Lipin 2 | 1.584 | 1.58E-03 |
| LPPR2 | Lipid phosphate phosphatase-related protein type 2 | 2.238 | 3.29E-06 |
| LRIG1 | Leucine-rich repeats and immunoglobulin-like domains 1 | -1.347 | 1.29E-04 |
| LRP10 | Low density lipoprotein receptor-related protein 10 | -1.283 | 3.11E-04 |
| LRP11 | Low density lipoprotein receptor-related protein 11 | 1.253 | 1.92E-02 |
| LRP3 | Low density lipoprotein receptor-related protein 3 | -1.285 | 1.84E-02 |
| LRP6 | Low density lipoprotein receptor-related protein 6 | -1.653 | 1.99E-04 |
| LRRC20 | Leucine rich repeat containing 20 | -1.387 | 7.43E-04 |
| LRRC39 | Leucine rich repeat containing 39 | -1.462 | 1.11E-03 |
| LRRC47 | Leucine rich repeat containing 47 | -1.252 | 1.65E-03 |
| LRRC61 | Leucine rich repeat containing 61 | -1.72 | 5.67E-03 |
| LRRC8D | Leucine rich repeat containing 8 family, member D | 1.526 | 4.82E-03 |
| LRRN1 | Leucine rich repeat neuronal 1 | 1.688 | 3.32E-05 |
| LSM6 | LSM6 homolog, U6 small nuclear RNA and mRNA degradation associated | -1.355 | 3.93E-04 |
| LTA4H | Leukotriene A4 hydrolase | -1.369 | 1.08E-03 |
| LTBP2 | Latent transforming growth factor beta binding protein 2 | -1.505 | 2.19E-03 |
| LTBP3 | Latent transforming growth factor beta binding protein 3 | 1.307 | 2.19E-03 |
| LTV1 | LTV1 ribosome biogenesis factor | 1.431 | 3.85E-04 |
| LUM | Lumican | 3.106 | 7.72E-15 |
| LY6G5B | Lymphocyte antigen 6 complex, locus G5B | -1.615 | 7.32E-04 |
| LYN | LYN proto-oncogene, Src family tyrosine kinase | 1.478 | 1.62E-04 |
| LYPLA2 | Lysophospholipase II | 1.308 | 5.05E-03 |
| LYRM9 | LYR motif containing 9 | 1.262 | 1.26E-03 |
| LYZ | Lysozyme | 2.057 | 9.51E-03 |
| LZTS3 | Leucine zipper, putative tumor suppressor family member 3 | -1.319 | 7.91E-04 |
| MACROD1 | MACRO domain containing 1 | -1.313 | 4.87E-03 |
| MAGEH1 | Melanoma antigen family H1 | -1.291 | 1.00E-02 |
| MAGIX | MAGI family member, X-linked | 2.318 | 4.57E-06 |
| MAK16 | MAK16 homolog | 1.372 | 6.11E-05 |
| MAL | Mal, T-cell differentiation protein | -2.355 | 5.92E-06 |
| MALL | Mal, T-cell differentiation protein-like | 1.46 | 3.99E-03 |
| MAMSTR | MEF2 activating motif and SAP domain containing transcriptional regulator | 2.058 | 1.05E-10 |
| MAN1C1 | Mannosidase, alpha, class 1C, member 1 | -1.331 | 7.14E-03 |
| MAN2B1 | Mannosidase, alpha, class 2B, member 1 | -1.261 | 3.30E-03 |
| MANBA | Mannosidase, beta A, lysosomal | -1.534 | 2.40E-03 |
| MANEAL | Mannosidase, endo-alpha-like | -1.953 | 9.02E-07 |
| MANF | Mesencephalic astrocyte-derived neurotrophic factor | 1.266 | 1.12E-03 |
| MAP1LC3B | Microtubule-associated protein 1 light chain 3 beta | -1.555 | 1.39E-03 |
| MAP3K1 | Mitogen-activated protein kinase kinase kinase 1, E3 ubiquitin protein ligase | -1.395 | 7.43E-03 |
| MAP3K14 | Mitogen-activated protein kinase kinase kinase 14 | 1.436 | 6.45E-03 |
| MAP4 | Microtubule-associated protein 4 | -1.292 | 6.60E-04 |
| MAPK12 | Mitogen-activated protein kinase 12 | -1.294 | 5.37E-03 |
| MAPK8 | Mitogen-activated protein kinase 8 | -1.363 | 1.21E-02 |
| MAPK8IP3 | Mitogen-activated protein kinase 8 interacting protein 3 | -1.339 | 9.16E-03 |
| MAPT | Microtubule-associated protein tau | 1.345 | 1.46E-03 |
| MARCKS | Myristoylated alanine-rich protein kinase C substrate | 1.352 | 2.07E-03 |
| MARCKSL1 | MARCKS-like 1 | 1.933 | 2.13E-06 |
| MARS | Methionyl-tRNA synthetase | 1.787 | 4.02E-06 |
| MARS2 | Methionyl-tRNA synthetase 2, mitochondrial | 1.572 | 1.79E-04 |
| MARVELD1 | MARVEL domain containing 1 | 1.292 | 1.25E-03 |
| MASP1 | Mannan-binding lectin serine peptidase 1 (C4/C2 activating component of Ra-reactive factor) | -1.658 | 7.52E-06 |
| MAST3 | Microtubule associated serine/threonine kinase 3 | 1.493 | 6.55E-03 |
| MAST4 | Microtubule associated serine/threonine kinase family member 4 | -1.351 | 4.49E-04 |
| MAT2A | Methionine adenosyltransferase II, alpha | 1.363 | 1.03E-03 |
| MAX | MYC associated factor X | -1.673 | 1.57E-07 |
| MBD3 | Methyl-CpG binding domain protein 3 | 1.685 | 7.94E-10 |
| MBNL1 | Muscleblind-like splicing regulator 1 | -1.669 | 2.99E-08 |
| MBP | Myelin basic protein | -1.916 | 7.42E-08 |
| MCCC1 | Methylcrotonoyl-CoA carboxylase 1 (alpha) | -1.476 | 9.88E-06 |
| MCCC2 | Methylcrotonoyl-CoA carboxylase 2 (beta) | -1.49 | 8.71E-06 |
| MCF2L | MCF.2 cell line derived transforming sequence-like | -1.778 | 3.12E-05 |
| MCHR1 | Melanin-concentrating hormone receptor 1 | 2.576 | 4.30E-07 |
| MCL1 | Myeloid cell leukemia 1 | 1.3 | 1.28E-03 |
| MCM2 | Minichromosome maintenance complex component 2 | -1.427 | 1.96E-04 |
| MCM6 | Minichromosome maintenance complex component 6 | 1.26 | 1.53E-02 |
| MCOLN1 | Mucolipin 1 | -1.335 | 2.49E-04 |
| MDM2 | MDM2 proto-oncogene, E3 ubiquitin protein ligase | 1.424 | 7.69E-05 |
| ME1 | Malic enzyme 1, NADP(+)-dependent, cytosolic | 1.315 | 5.23E-03 |
| ME3 | Malic enzyme 3, NADP(+)-dependent, mitochondrial | -2.269 | 1.98E-10 |
| MED1 | Mediator complex subunit 1 | -1.252 | 2.63E-03 |
| MED12 | Mediator complex subunit 12 | 1.33 | 1.74E-03 |
| MED13L | Mediator complex subunit 13-like | -1.339 | 1.11E-05 |
| MED20 | Mediator complex subunit 20 | 1.624 | 9.07E-06 |
| MEGF9 | Multiple EGF-like-domains 9 | -1.426 | 1.71E-02 |
| MEIS1 | Meis homeobox 1 | -1.3 | 2.19E-03 |
| MEOX1 | Mesenchyme homeobox 1 | 2.135 | 1.60E-07 |
| MEST | Mesoderm specific transcript | 4.259 | 2.00E-23 |
| MET | MET proto-oncogene, receptor tyrosine kinase | -1.546 | 1.55E-04 |
| METAP2 | Methionyl aminopeptidase 2 | -1.262 | 1.12E-02 |
| METTL1 | Methyltransferase like 1 | 1.69 | 2.65E-07 |
| METTL13 | Methyltransferase like 13 | 1.34 | 1.62E-03 |
| METTL15 | Methyltransferase like 15 | 1.276 | 4.08E-03 |
| METTL16 | Methyltransferase like 16 | -1.295 | 1.17E-03 |
| METTL23 | Methyltransferase like 23 | 1.345 | 1.61E-03 |
| METTL3 | Methyltransferase like 3 | 1.259 | 4.39E-03 |
| METTL8 | Methyltransferase like 8 | 1.525 | 2.33E-03 |
| MEX3D | Mex-3 RNA binding family member D | -1.515 | 2.81E-05 |
| MFAP2 | Microfibrillar-associated protein 2 | 1.584 | 2.06E-03 |
| MFAP4 | Microfibrillar-associated protein 4 | -1.375 | 1.12E-02 |
| MFAP5 | Microfibrillar associated protein 5 | 1.444 | 1.21E-04 |
| MFSD1 | Major facilitator superfamily domain containing 1 | -1.257 | 4.12E-03 |
| MFSD8 | Major facilitator superfamily domain containing 8 | 1.33 | 1.90E-02 |
| MGA | MGA, MAX dimerization protein | -1.449 | 6.12E-03 |
| MGEA5 | Meningioma expressed antigen 5 (hyaluronidase) | -1.47 | 2.30E-09 |
| MGP | Matrix Gla protein | 1.304 | 1.44E-02 |
| MICAL1 | Microtubule associated monooxygenase, calponin and LIM domain containing 1 | 1.488 | 1.77E-02 |
| MICAL2 | Microtubule associated monooxygenase, calponin and LIM domain containing 2 | 1.613 | 2.78E-04 |
| MICALL2 | MICAL-like 2 | 1.587 | 2.13E-02 |
| MIEN1 | Migration and invasion enhancer 1 | 1.868 | 3.84E-11 |
| MINA | MYC induced nuclear antigen | -1.397 | 6.23E-05 |
| MITF | Microphthalmia-associated transcription factor | -1.503 | 8.69E-06 |
| MKL2 | MKL/myocardin-like 2 | -1.405 | 2.39E-05 |
| MKRN2 | Makorin ring finger protein 2 | -1.503 | 2.64E-03 |
| MLEC | Malectin | 1.299 | 7.91E-03 |
| MLIP | Muscular LMNA-interacting protein | -1.409 | 1.05E-08 |
| MLLT11 | Myeloid/lymphoid or mixed-lineage leukemia; translocated to, 11 | 2.324 | 7.22E-07 |
| MLLT6 | Myeloid/lymphoid or mixed-lineage leukemia; translocated to, 6 | -1.818 | 3.73E-10 |
| MLX | MLX, MAX dimerization protein | -1.291 | 7.97E-04 |
| MLXIP | MLX interacting protein | -1.319 | 1.47E-03 |
| MLYCD | Malonyl-CoA decarboxylase | 1.781 | 3.59E-06 |
| MMACHC | Methylmalonic aciduria (cobalamin deficiency) cblC type, with homocystinuria | 1.374 | 4.85E-05 |
| MMP15 | Matrix metallopeptidase 15 (membrane-inserted) | 1.56 | 5.77E-05 |
| MMP16 | Matrix metallopeptidase 16 (membrane-inserted) | 1.454 | 7.65E-03 |
| MMP19 | Matrix metallopeptidase 19 | 1.789 | 2.85E-05 |
| MNS1 | Meiosis-specific nuclear structural 1 | -2.162 | 1.87E-06 |
| MOB1B | MOB kinase activator 1B | -1.47 | 1.33E-02 |
| MOB3B | MOB kinase activator 3B | -2.002 | 2.96E-06 |
| MOB3C | MOB kinase activator 3C | 1.627 | 3.51E-05 |
| MOGS | Mannosyl-oligosaccharide glucosidase | 1.584 | 4.86E-07 |
| MON1A | MON1 secretory trafficking family member A | 1.408 | 4.55E-04 |
| MON2 | MON2 homolog, regulator of endosome-to-Golgi trafficking | -1.489 | 6.56E-04 |
| MOSPD1 | Motile sperm domain containing 1 | -1.292 | 2.42E-02 |
| MOV10 | Mov10 RISC complex RNA helicase | 1.337 | 7.50E-03 |
| MPDZ | Multiple PDZ domain protein | -1.406 | 1.27E-04 |
| MPI | Mannose phosphate isomerase | -1.277 | 5.68E-03 |
| MPND | MPN domain containing | 1.811 | 2.68E-08 |
| MPP6 | Membrane protein, palmitoylated 6 (MAGUK p55 subfamily member 6) | -2.439 | 7.90E-11 |
| MPP7 | Membrane protein, palmitoylated 7 (MAGUK p55 subfamily member 7) | -1.446 | 9.66E-04 |
| MPPE1 | Metallophosphoesterase 1 | 1.721 | 3.46E-05 |
| MPZL1 | Myelin protein zero-like 1 | 1.274 | 1.55E-02 |
| MPZL3 | Myelin protein zero-like 3 | -1.449 | 2.19E-02 |
| MRAS | Muscle RAS oncogene homolog | 1.352 | 2.33E-05 |
| MRC2 | Mannose receptor, C type 2 | 1.595 | 3.31E-06 |
| MRO | Maestro | 1.315 | 1.28E-02 |
| MROH1 | Maestro heat-like repeat family member 1 | -1.298 | 1.07E-02 |
| MRPL12 | Mitochondrial ribosomal protein L12 | 1.265 | 1.51E-03 |
| MRPL15 | Mitochondrial ribosomal protein L15 | 1.406 | 8.11E-07 |
| MRPL17 | Mitochondrial ribosomal protein L17 | 1.319 | 1.04E-04 |
| MRPL19 | Mitochondrial ribosomal protein L19 | 1.295 | 1.12E-02 |
| MRPL27 | Mitochondrial ribosomal protein L27 | 1.25 | 1.76E-02 |
| MRPL41 | Mitochondrial ribosomal protein L41 | -1.287 | 2.01E-02 |
| MRPS23 | Mitochondrial ribosomal protein S23 | -1.257 | 2.42E-04 |
| MRPS28 | Mitochondrial ribosomal protein S28 | 1.365 | 1.47E-04 |
| MRPS9 | Mitochondrial ribosomal protein S9 | -1.312 | 1.56E-04 |
| MRTO4 | MRT4 homolog, ribosome maturation factor | 1.268 | 7.79E-04 |
| MSANTD3 | Myb/SANT-like DNA-binding domain containing 3 | -1.33 | 2.31E-03 |
| MSMO1 | Methylsterol monooxygenase 1 | 1.895 | 1.35E-07 |
| MSN | Moesin | 1.344 | 2.05E-04 |
| MSTN | Myostatin | -1.473 | 3.20E-04 |
| MSTO1 | Misato 1, mitochondrial distribution and morphology regulator | -1.354 | 5.65E-04 |
| MTCH2 | Mitochondrial carrier 2 | 1.268 | 2.75E-04 |
| MTF1 | Metal-regulatory transcription factor 1 | -1.387 | 3.13E-05 |
| MTHFD1 | Methylenetetrahydrofolate dehydrogenase (NADP+ dependent) 1, methenyltetrahydrofolate cyclohydrolase, formyltetrahydrofolate synthetase | -1.257 | 5.85E-04 |
| MTHFR | Methylenetetrahydrofolate reductase (NAD(P)H) | -1.596 | 4.35E-07 |
| MTMR1 | Myotubularin related protein 1 | 1.255 | 8.37E-03 |
| MTMR12 | Myotubularin related protein 12 | -1.285 | 2.62E-02 |
| MTMR14 | Myotubularin related protein 14 | -1.425 | 6.22E-07 |
| MTMR3 | Myotubularin related protein 3 | -1.416 | 1.07E-06 |
| MTMR7 | Myotubularin related protein 7 | -1.39 | 2.12E-03 |
| MTURN | Maturin, neural progenitor differentiation regulator homolog (Xenopus) | -1.52 | 1.18E-03 |
| MXD1 | MAX dimerization protein 1 | -1.446 | 1.68E-04 |
| MXRA5 | Matrix-remodelling associated 5 | 2.168 | 2.14E-08 |
| MYADM | Myeloid-associated differentiation marker | 1.804 | 3.85E-11 |
| MYBPC1 | Myosin binding protein C, slow type | -1.257 | 1.87E-02 |
| MYBPH | Myosin binding protein H | 2.149 | 2.43E-03 |
| MYC | V-myc avian myelocytomatosis viral oncogene homolog | 2.514 | 6.84E-13 |
| MYDGF | Myeloid-derived growth factor | 1.308 | 5.55E-03 |
| MYF6 | Myogenic factor 6 (herculin) | 1.516 | 7.17E-07 |
| MYH14 | Myosin, heavy chain 14, non-muscle | -1.802 | 7.22E-09 |
| MYH4 | Myosin, heavy chain 4, skeletal muscle | -1.261 | 1.88E-02 |
| MYL12B | Myosin, light chain 12B, regulatory | 1.322 | 1.23E-04 |
| MYL3 | Myosin, light chain 3, alkali; ventricular, skeletal, slow | 1.764 | 3.68E-04 |
| MYL6 | Myosin, light chain 6, alkali, smooth muscle and non-muscle | 1.658 | 2.90E-08 |
| MYL6B | Myosin, light chain 6B, alkali, smooth muscle and non-muscle | 5.14 | 1.59E-07 |
| MYL9 | Myosin, light chain 9, regulatory | 1.902 | 1.09E-08 |
| MYLIP | Myosin regulatory light chain interacting protein | -1.733 | 3.12E-11 |
| MYLK2 | Myosin light chain kinase 2 | -1.644 | 1.03E-07 |
| MYLK3 | Myosin light chain kinase 3 | -3.419 | 1.37E-16 |
| MYO10 | Myosin X | 1.263 | 9.98E-03 |
| MYO1B | Myosin IB | 1.431 | 6.16E-04 |
| MYO1E | Myosin IE | 1.301 | 7.40E-04 |
| MYO9A | Myosin IXA | -1.567 | 2.13E-03 |
| MYOD1 | Myogenic differentiation 1 | 1.507 | 1.19E-03 |
| MYOM3 | Myomesin 3 | 1.561 | 4.37E-04 |
| MYOZ1 | Myozenin 1 | -1.359 | 1.94E-05 |
| MYOZ2 | Myozenin 2 | 1.676 | 2.39E-05 |
| N4BP1 | NEDD4 binding protein 1 | -1.322 | 5.62E-05 |
| N6AMT1 | N-6 adenine-specific DNA methyltransferase 1 (putative) | 1.557 | 1.61E-04 |
| NAA38 | N(alpha)-acetyltransferase 38, NatC auxiliary subunit | 1.666 | 1.40E-06 |
| NACC1 | Nucleus accumbens associated 1, BEN and BTB (POZ) domain containing | 1.325 | 2.67E-03 |
| NAGK | N-acetylglucosamine kinase | 1.603 | 1.07E-05 |
| NAGLU | N-acetylglucosaminidase, alpha | 1.439 | 5.88E-05 |
| NAMPT | Nicotinamide phosphoribosyltransferase | -1.715 | 9.41E-08 |
| NANS | N-acetylneuraminic acid synthase | 1.437 | 3.76E-06 |
| NAPG | N-ethylmaleimide-sensitive factor attachment protein, gamma | 1.251 | 4.31E-03 |
| NARS2 | Asparaginyl-tRNA synthetase 2, mitochondrial (putative) | 1.343 | 1.15E-03 |
| NAT10 | N-acetyltransferase 10 (GCN5-related) | 1.311 | 2.06E-03 |
| NAT14 | N-acetyltransferase 14 (GCN5-related, putative) | -1.435 | 2.28E-04 |
| NCALD | Neurocalcin delta | 1.685 | 2.01E-07 |
| NCAM1 | Neural cell adhesion molecule 1 | 1.47 | 2.71E-02 |
| NCAPH2 | Non-SMC condensin II complex, subunit H2 | -1.37 | 2.26E-03 |
| NCBP2 | Nuclear cap binding protein subunit 2, 20kDa | 1.494 | 1.40E-06 |
| NCEH1 | Neutral cholesterol ester hydrolase 1 | -1.368 | 1.09E-02 |
| NCK2 | NCK adaptor protein 2 | -1.285 | 2.63E-03 |
| NCOA1 | Nuclear receptor coactivator 1 | -1.275 | 2.08E-02 |
| NCOA2 | Nuclear receptor coactivator 2 | -2.043 | 1.41E-10 |
| NDRG1 | N-myc downstream regulated 1 | -1.373 | 4.78E-04 |
| NDRG2 | NDRG family member 2 | 1.559 | 1.46E-06 |
| NDUFA4L2 | NADH dehydrogenase (ubiquinone) 1 alpha subcomplex, 4-like 2 | 1.476 | 5.42E-03 |
| NDUFAF5 | NADH dehydrogenase (ubiquinone) complex I, assembly factor 5 | 1.38 | 1.68E-03 |
| NDUFB10 | NADH dehydrogenase (ubiquinone) 1 beta subcomplex, 10, 22kDa | -1.333 | 2.12E-04 |
| NDUFC1 | NADH dehydrogenase (ubiquinone) 1, subcomplex unknown, 1, 6kDa | 1.405 | 1.12E-03 |
| NEDD1 | Neural precursor cell expressed, developmentally down-regulated 1 | 1.25 | 1.95E-02 |
| NEDD4 | Neural precursor cell expressed, developmentally down-regulated 4, E3 ubiquitin protein ligase | -2.078 | 3.59E-06 |
| NEDD4L | Neural precursor cell expressed, developmentally down-regulated 4-like, E3 ubiquitin protein ligase | -1.967 | 1.42E-10 |
| NEK7 | NIMA-related kinase 7 | -1.492 | 3.63E-06 |
| NELFCD | Negative elongation factor complex member C/D | -1.323 | 3.08E-04 |
| Nes | Nestin | 1.855 | 8.53E-05 |
| NEXN | Nexilin (F actin binding protein) | 1.61 | 7.47E-03 |
| NFATC2IP | Nuclear factor of activated T-cells, cytoplasmic, calcineurin-dependent 2 interacting protein | 1.276 | 4.55E-03 |
| NFATC3 | Nuclear factor of activated T-cells, cytoplasmic, calcineurin-dependent 3 | -1.527 | 1.33E-04 |
| NFIA | Nuclear factor I/A | -1.349 | 5.13E-04 |
| NFIL3 | Nuclear factor, interleukin 3 regulated | 2.06 | 2.44E-06 |
| NFKB2 | Nuclear factor of kappa light polypeptide gene enhancer in B-cells 2 (p49/p100) | 1.886 | 1.33E-08 |
| NFKBIA | Nuclear factor of kappa light polypeptide gene enhancer in B-cells inhibitor, alpha | -1.869 | 2.32E-06 |
| NIPSNAP3A | Nipsnap homolog 3A (C. elegans) | 2.174 | 5.06E-05 |
| NKTR | Natural killer cell triggering receptor | 1.765 | 3.90E-06 |
| NLGN2 | Neuroligin 2 | 1.389 | 1.51E-02 |
| NLRX1 | NLR family member X1 | -1.272 | 2.49E-03 |
| NME2 | NME/NM23 nucleoside diphosphate kinase 2 | 1.434 | 4.36E-05 |
| NMRK2 | Nicotinamide riboside kinase 2 | 2.38 | 4.98E-07 |
| NOB1 | NIN1/RPN12 binding protein 1 homolog | 1.261 | 4.56E-03 |
| NOC3L | NOC3-like DNA replication regulator | 1.377 | 9.02E-04 |
| NOL6 | Nucleolar protein 6 (RNA-associated) | 1.388 | 2.85E-02 |
| NOL8 | Nucleolar protein 8 | -1.49 | 1.28E-03 |
| NOL9 | Nucleolar protein 9 | 1.274 | 2.52E-02 |
| NOP14 | NOP14 nucleolar protein | 1.277 | 2.11E-02 |
| NOP16 | NOP16 nucleolar protein | 1.573 | 2.02E-09 |
| NOP58 | NOP58 ribonucleoprotein | 1.311 | 1.00E-02 |
| NOS1 | Nitric oxide synthase 1 (neuronal) | -1.677 | 2.88E-05 |
| NOS2 | Nitric oxide synthase 2, inducible | 2.05 | 6.25E-03 |
| NOSTRIN | Nitric oxide synthase trafficking | -1.462 | 1.40E-02 |
| NOV | Nephroblastoma overexpressed | 1.981 | 1.26E-05 |
| NPAT | Nuclear protein, ataxia-telangiectasia locus | -1.438 | 5.94E-03 |
| NPEPPS | Aminopeptidase puromycin sensitive | 1.281 | 1.77E-03 |
| NPM3 | Nucleophosmin/nucleoplasmin 3 | 2.163 | 1.99E-08 |
| NPNT | Nephronectin | 2.219 | 2.84E-14 |
| NPRL3 | NPR3-like, GATOR1 complex subunit | 1.268 | 7.76E-03 |
| NQO1 | NAD(P)H dehydrogenase, quinone 1 | 1.44 | 4.59E-07 |
| NR1D2 | Nuclear receptor subfamily 1, group D, member 2 | -1.255 | 3.51E-04 |
| NR2C2 | Nuclear receptor subfamily 2, group C, member 2 | -1.316 | 1.03E-03 |
| NR3C1 | Nuclear receptor subfamily 3, group C, member 1 (glucocorticoid receptor) | -2.063 | 8.53E-06 |
| NRAP | Nebulin-related anchoring protein | -2.243 | 4.47E-16 |
| NRDE2 | NRDE-2, necessary for RNA interference, domain containing | -1.371 | 5.86E-05 |
| NRF1 | Nuclear respiratory factor 1 | -1.322 | 7.36E-03 |
| NRIP1 | Nuclear receptor interacting protein 1 | -1.827 | 9.43E-04 |
| NRM | Nurim (nuclear envelope membrane protein) | 1.548 | 3.13E-04 |
| NRP2 | Neuropilin 2 | 1.618 | 3.01E-03 |
| NSDHL | NAD(P) dependent steroid dehydrogenase-like | 1.658 | 3.70E-04 |
| NT5M | 5',3'-nucleotidase, mitochondrial | 1.29 | 8.75E-03 |
| NUAK1 | NUAK family, SNF1-like kinase, 1 | -1.599 | 1.96E-06 |
| NUB1 | Negative regulator of ubiquitin-like proteins 1 | -1.484 | 5.05E-09 |
| NUBPL | Nucleotide binding protein-like | 1.5 | 1.42E-03 |
| NUDCD2 | NudC domain containing 2 | 1.252 | 3.18E-03 |
| NUDT12 | Nudix (nucleoside diphosphate linked moiety X)-type motif 12 | 1.323 | 2.18E-02 |
| NUDT14 | Nudix (nucleoside diphosphate linked moiety X)-type motif 14 | 1.504 | 1.11E-02 |
| NUDT16L1 | Nudix (nucleoside diphosphate linked moiety X)-type motif 16-like 1 | -1.313 | 1.65E-02 |
| NUDT2 | Nudix (nucleoside diphosphate linked moiety X)-type motif 2 | -1.293 | 7.29E-04 |
| NUDT21 | Nudix (nucleoside diphosphate linked moiety X)-type motif 21 | -1.262 | 4.12E-03 |
| NUMA1 | Nuclear mitotic apparatus protein 1 | -1.278 | 7.61E-04 |
| NUMBL | Numb homolog (Drosophila)-like | -1.32 | 2.25E-02 |
| NUP133 | Nucleoporin 133kDa | 1.327 | 9.34E-03 |
| NUP54 | Nucleoporin 54kDa | -1.462 | 6.88E-03 |
| NUTF2 | Nuclear transport factor 2 | 1.316 | 4.16E-05 |
| NXN | Nucleoredoxin | 1.256 | 2.56E-04 |
| OAT | Ornithine aminotransferase | 1.566 | 2.34E-05 |
| OAZ2 | Ornithine decarboxylase antizyme 2 | -1.569 | 3.94E-10 |
| OBFC1 | Oligonucleotide/oligosaccharide-binding fold containing 1 | 1.273 | 4.35E-03 |
| OBSL1 | Obscurin-like 1 | 1.274 | 5.50E-03 |
| ODC1 | Ornithine decarboxylase 1 | 1.331 | 2.33E-02 |
| ODF2L | Outer dense fiber of sperm tails 2-like | 1.802 | 2.29E-02 |
| OFD1 | Oral-facial-digital syndrome 1 | -1.57 | 1.14E-03 |
| OGN | Osteoglycin | 1.556 | 6.92E-03 |
| OLA1 | Obg-like ATPase 1 | 1.359 | 6.03E-05 |
| OLFML2B | Olfactomedin-like 2B | 1.516 | 2.94E-04 |
| OR51E2 | Olfactory receptor, family 51, subfamily E, member 2 | 1.394 | 4.41E-03 |
| ORC5 | Origin recognition complex, subunit 5 | 1.606 | 4.71E-03 |
| ORMDL2 | ORMDL sphingolipid biosynthesis regulator 2 | 1.262 | 1.48E-02 |
| ORMDL3 | ORMDL sphingolipid biosynthesis regulator 3 | -1.267 | 2.13E-03 |
| OSBPL10 | Oxysterol binding protein-like 10 | -1.389 | 8.20E-03 |
| OSBPL1A | Oxysterol binding protein-like 1A | -1.266 | 8.60E-04 |
| OSBPL5 | Oxysterol binding protein-like 5 | -1.297 | 1.42E-02 |
| OSBPL7 | Oxysterol binding protein-like 7 | 1.529 | 7.77E-03 |
| OSBPL8 | Oxysterol binding protein-like 8 | -1.825 | 1.09E-04 |
| OSER1 | Oxidative stress responsive serine-rich 1 | -1.438 | 1.68E-06 |
| OSGEPL1 | O-sialoglycoprotein endopeptidase-like 1 | 1.336 | 5.31E-03 |
| OSTF1 | Osteoclast stimulating factor 1 | 1.28 | 3.76E-03 |
| OTUB2 | OTU deubiquitinase, ubiquitin aldehyde binding 2 | 1.415 | 2.56E-02 |
| OTUD1 | OTU deubiquitinase 1 | 3.619 | 1.62E-07 |
| OTUD4 | OTU deubiquitinase 4 | -1.413 | 6.05E-03 |
| OTUD5 | OTU deubiquitinase 5 | -1.354 | 1.75E-03 |
| OTUD7B | OTU deubiquitinase 7B | -1.292 | 1.69E-04 |
| P2RX5 | Purinergic receptor P2X, ligand gated ion channel, 5 | 1.254 | 7.69E-03 |
| P4HA1 | Prolyl 4-hydroxylase, alpha polypeptide I | 1.487 | 2.55E-05 |
| P4HA2 | Prolyl 4-hydroxylase, alpha polypeptide II | 1.694 | 3.20E-08 |
| PAFAH2 | Platelet-activating factor acetylhydrolase 2, 40kDa | 1.673 | 2.08E-07 |
| PAIP2B | Poly(A) binding protein interacting protein 2B | -1.554 | 7.30E-08 |
| PALD1 | Phosphatase domain containing, paladin 1 | 1.742 | 2.95E-03 |
| PAN3 | PAN3 poly(A) specific ribonuclease subunit | -1.39 | 3.12E-03 |
| PAQR3 | Progestin and adipoQ receptor family member III | -1.691 | 4.20E-09 |
| PAQR4 | Progestin and adipoQ receptor family member IV | 1.585 | 8.23E-05 |
| PARD3B | Par-3 family cell polarity regulator beta | -1.543 | 2.89E-03 |
| PARP14 | Poly (ADP-ribose) polymerase family, member 14 | 1.574 | 1.81E-03 |
| PARP2 | Poly (ADP-ribose) polymerase 2 | -1.445 | 1.28E-05 |
| PARS2 | Prolyl-tRNA synthetase 2, mitochondrial (putative) | -1.392 | 4.17E-03 |
| PARVA | Parvin, alpha | 1.599 | 1.91E-08 |
| PATZ1 | POZ (BTB) and AT hook containing zinc finger 1 | -1.711 | 6.65E-08 |
| PBRM1 | Polybromo 1 | -1.573 | 2.62E-04 |
| PCDH12 | Protocadherin 12 | 3.345 | 6.59E-15 |
| PCGF5 | Polycomb group ring finger 5 | -1.867 | 9.06E-04 |
| PCID2 | PCI domain containing 2 | -1.317 | 6.73E-05 |
| PCIF1 | PDX1 C-terminal inhibiting factor 1 | -1.392 | 4.87E-04 |
| PCMTD2 | Protein-L-isoaspartate (D-aspartate) O-methyltransferase domain containing 2 | -1.384 | 1.19E-05 |
| PCNT | Pericentrin | -1.521 | 5.80E-09 |
| PCOLCE | Procollagen C-endopeptidase enhancer | 1.301 | 2.53E-02 |
| PCYT1A | Phosphate cytidylyltransferase 1, choline, alpha | -1.258 | 9.66E-04 |
| PDCL3 | Phosducin-like 3 | 1.329 | 4.78E-04 |
| PDE12 | Phosphodiesterase 12 | 1.486 | 2.65E-04 |
| PDE4B | Phosphodiesterase 4B, cAMP-specific | 1.452 | 2.37E-02 |
| PDE4C | Phosphodiesterase 4C, cAMP-specific | -1.25 | 1.07E-02 |
| PDE7B | Phosphodiesterase 7B | -1.423 | 2.32E-04 |
| PDF | Peptide deformylase (mitochondrial) | 1.372 | 3.05E-04 |
| PDIA4 | Protein disulfide isomerase family A, member 4 | 1.584 | 5.51E-08 |
| PDK1 | Pyruvate dehydrogenase kinase, isozyme 1 | -1.278 | 3.30E-03 |
| PDK2 | Pyruvate dehydrogenase kinase, isozyme 2 | -1.285 | 1.51E-03 |
| PDK3 | Pyruvate dehydrogenase kinase, isozyme 3 | 1.334 | 1.06E-02 |
| PDLIM7 | PDZ and LIM domain 7 (enigma) | 1.303 | 4.75E-03 |
| PDPR | Pyruvate dehydrogenase phosphatase regulatory subunit | -1.715 | 2.40E-16 |
| PDSS2 | Prenyl (decaprenyl) diphosphate synthase, subunit 2 | 1.327 | 7.05E-04 |
| PDXDC1 | Pyridoxal-dependent decarboxylase domain containing 1 | -1.744 | 4.39E-09 |
| PDZD9 | PDZ domain containing 9 | -1.423 | 3.40E-03 |
| PDZRN3 | PDZ domain containing ring finger 3 | -1.372 | 1.70E-04 |
| PEA15 | Phosphoprotein enriched in astrocytes 15 | 1.395 | 1.79E-05 |
| PEAK1 | Pseudopodium-enriched atypical kinase 1 | -1.388 | 1.15E-02 |
| PECAM1 | Platelet/endothelial cell adhesion molecule 1 | 1.536 | 3.58E-05 |
| PELI3 | Pellino E3 ubiquitin protein ligase family member 3 | -1.501 | 4.62E-03 |
| PEMT | Phosphatidylethanolamine N-methyltransferase | 1.547 | 1.84E-03 |
| PER1 | Period circadian clock 1 | -1.484 | 2.56E-03 |
| PER2 | Period circadian clock 2 | -1.414 | 3.10E-03 |
| PEX2 | Peroxisomal biogenesis factor 2 | 1.322 | 7.22E-03 |
| PEX26 | Peroxisomal biogenesis factor 26 | -1.29 | 2.09E-03 |
| PEX7 | Peroxisomal biogenesis factor 7 | 1.325 | 1.51E-03 |
| PFKFB1 | 6-phosphofructo-2-kinase/fructose-2,6-biphosphatase 1 | 1.347 | 2.68E-04 |
| PFKFB2 | 6-phosphofructo-2-kinase/fructose-2,6-biphosphatase 2 | -1.303 | 2.37E-03 |
| PFKFB4 | 6-phosphofructo-2-kinase/fructose-2,6-biphosphatase 4 | -1.849 | 1.69E-11 |
| PFKM | Phosphofructokinase, muscle | -1.37 | 8.46E-07 |
| PFKP | Phosphofructokinase, platelet | 1.441 | 7.51E-03 |
| PFN2 | Profilin 2 | 1.272 | 3.10E-03 |
| PGK1 | Phosphoglycerate kinase 1 | 1.44 | 1.44E-04 |
| PGM1 | Phosphoglucomutase 1 | -1.387 | 4.56E-06 |
| PGM2L1 | Phosphoglucomutase 2-like 1 | -3.478 | 3.27E-10 |
| PGPEP1 | Pyroglutamyl-peptidase I | -1.393 | 2.14E-03 |
| PGPEP1L | Pyroglutamyl-peptidase I-like | -2.767 | 4.79E-18 |
| PGRMC1 | Progesterone receptor membrane component 1 | 1.552 | 2.99E-04 |
| PGRMC2 | Progesterone receptor membrane component 2 | -1.59 | 4.83E-07 |
| PHAX | Phosphorylated adaptor for RNA export | 1.273 | 3.65E-03 |
| PHC1 | Polyhomeotic homolog 1 (Drosophila) | -1.368 | 2.01E-04 |
| PHF14 | PHD finger protein 14 | 1.251 | 1.57E-02 |
| PHF20L1 | PHD finger protein 20-like 1 | -1.416 | 5.82E-04 |
| PHF21A | PHD finger protein 21A | 1.362 | 1.22E-03 |
| PHGDH | Phosphoglycerate dehydrogenase | 12.171 | 2.54E-13 |
| PHIP | Pleckstrin homology domain interacting protein | -2.169 | 1.26E-03 |
| PHTF1 | Putative homeodomain transcription factor 1 | 1.725 | 1.55E-04 |
| PICALM | Phosphatidylinositol binding clathrin assembly protein | 1.253 | 9.82E-04 |
| PIEZO1 | Piezo-type mechanosensitive ion channel component 1 | 1.594 | 3.05E-06 |
| PIGH | Phosphatidylinositol glycan anchor biosynthesis, class H | 1.335 | 3.10E-03 |
| PIGP | Phosphatidylinositol glycan anchor biosynthesis, class P | 1.514 | 1.87E-03 |
| PIGU | Phosphatidylinositol glycan anchor biosynthesis, class U | 1.345 | 1.29E-02 |
| PIK3AP1 | Phosphoinositide-3-kinase adaptor protein 1 | -1.818 | 3.20E-08 |
| PIK3IP1 | Phosphoinositide-3-kinase interacting protein 1 | -1.98 | 7.01E-09 |
| PIK3R1 | Phosphoinositide-3-kinase, regulatory subunit 1 (alpha) | -1.289 | 1.02E-03 |
| PIKFYVE | Phosphoinositide kinase, FYVE finger containing | -1.697 | 6.38E-03 |
| PIM2 | Pim-2 proto-oncogene, serine/threonine kinase | -1.354 | 8.97E-03 |
| PINX1 | PIN2/TERF1 interacting, telomerase inhibitor 1 | 2.42 | 4.36E-09 |
| PITHD1 | PITH (C-terminal proteasome-interacting domain of thioredoxin-like) domain containing 1 | -1.316 | 3.08E-04 |
| PITPNA | Phosphatidylinositol transfer protein, alpha | -1.714 | 8.44E-10 |
| PJA2 | Praja ring finger 2, E3 ubiquitin protein ligase | -1.285 | 1.70E-04 |
| PKD1L3 | Polycystic kidney disease 1-like 3 | -1.73 | 9.23E-05 |
| PKMYT1 | Protein kinase, membrane associated tyrosine/threonine 1 | 1.539 | 4.68E-04 |
| PKNOX2 | PBX/knotted 1 homeobox 2 | -1.31 | 2.45E-04 |
| PLAC9 | Placenta-specific 9 | 1.373 | 4.56E-03 |
| PLAU | Plasminogen activator, urokinase | 1.422 | 3.90E-04 |
| PLBD1 | Phospholipase B domain containing 1 | -2.083 | 1.10E-17 |
| PLCL2 | Phospholipase C-like 2 | -1.55 | 7.90E-08 |
| PLD1 | Phospholipase D1, phosphatidylcholine-specific | -1.579 | 1.80E-03 |
| PLD2 | Phospholipase D2 | 1.521 | 1.50E-02 |
| PLEKHF1 | Pleckstrin homology domain containing, family F (with FYVE domain) member 1 | -1.443 | 3.84E-04 |
| PLEKHO2 | Pleckstrin homology domain containing, family O member 2 | 1.418 | 5.14E-04 |
| PLIN2 | Perilipin 2 | -1.947 | 1.45E-09 |
| PLIN4 | Perilipin 4 | -1.267 | 7.12E-03 |
| PLIN5 | Perilipin 5 | -1.685 | 5.86E-07 |
| PLOD1 | Procollagen-lysine, 2-oxoglutarate 5-dioxygenase 1 | 1.552 | 4.16E-06 |
| PLOD2 | Procollagen-lysine, 2-oxoglutarate 5-dioxygenase 2 | 2.146 | 1.63E-05 |
| PLOD3 | Procollagen-lysine, 2-oxoglutarate 5-dioxygenase 3 | 1.312 | 1.11E-02 |
| PLS3 | Plastin 3 | 1.275 | 1.80E-02 |
| PLVAP | Plasmalemma vesicle associated protein | 1.453 | 1.47E-02 |
| PLXDC1 | Plexin domain containing 1 | 1.736 | 2.95E-07 |
| PLXDC2 | Plexin domain containing 2 | 1.539 | 3.62E-03 |
| PLXNA1 | Plexin A1 | 1.25 | 1.91E-02 |
| PLXNA2 | Plexin A2 | 1.285 | 3.98E-03 |
| PLXND1 | Plexin D1 | 1.487 | 2.48E-03 |
| PM20D2 | Peptidase M20 domain containing 2 | -1.432 | 6.22E-03 |
| PMS1 | PMS1 homolog 1, mismatch repair system component | 1.568 | 2.19E-03 |
| PNMA1 | Paraneoplastic Ma antigen 1 | 1.277 | 2.30E-02 |
| PNPLA2 | Patatin-like phospholipase domain containing 2 | -2.095 | 9.03E-15 |
| PNPO | Pyridoxamine 5'-phosphate oxidase | 1.7 | 3.71E-11 |
| PNRC1 | Proline-rich nuclear receptor coactivator 1 | -1.595 | 1.24E-06 |
| POC1A | POC1 centriolar protein A | -1.39 | 1.13E-02 |
| POLD4 | Polymerase (DNA-directed), delta 4, accessory subunit | -1.369 | 7.54E-05 |
| POLG2 | Polymerase (DNA directed), gamma 2, accessory subunit | -1.342 | 3.31E-03 |
| POLR1A | Polymerase (RNA) I polypeptide A, 194kDa | 1.402 | 2.00E-04 |
| POLR2L | Polymerase (RNA) II (DNA directed) polypeptide L, 7.6kDa | 1.254 | 8.06E-04 |
| POLR3B | Polymerase (RNA) III (DNA directed) polypeptide B | 1.407 | 1.22E-05 |
| POLR3D | Polymerase (RNA) III (DNA directed) polypeptide D, 44kDa | 1.444 | 1.50E-04 |
| POLR3G | Polymerase (RNA) III (DNA directed) polypeptide G (32kD) | 1.573 | 6.97E-04 |
| POMGNT2 | Protein O-linked mannose N-acetylglucosaminyltransferase 2 (beta 1,4-) | 1.251 | 1.87E-02 |
| PON2 | Paraoxonase 2 | 1.276 | 2.65E-02 |
| PPA1 | Pyrophosphatase (inorganic) 1 | 1.793 | 1.65E-09 |
| PPAN | Peter pan homolog (Drosophila) | 1.256 | 2.27E-02 |
| PPARD | Peroxisome proliferator-activated receptor delta | -1.269 | 2.46E-02 |
| PPEF1 | Protein phosphatase, EF-hand calcium binding domain 1 | 1.503 | 1.75E-02 |
| PPFIBP1 | PTPRF interacting protein, binding protein 1 (liprin beta 1) | -1.3 | 3.74E-03 |
| PPIC | Peptidylprolyl isomerase C (cyclophilin C) | 2.169 | 1.72E-09 |
| PPID | Peptidylprolyl isomerase D | 1.324 | 7.81E-05 |
| PPIF | Peptidylprolyl isomerase F | 1.522 | 1.61E-04 |
| PPIL1 | Peptidylprolyl isomerase (cyclophilin)-like 1 | 1.319 | 9.96E-04 |
| PPIL3 | Peptidylprolyl isomerase (cyclophilin)-like 3 | 1.397 | 5.59E-03 |
| PPIP5K1 | Diphosphoinositol pentakisphosphate kinase 1 | 1.448 | 1.24E-06 |
| PPM1J | Protein phosphatase, Mg2+/Mn2+ dependent, 1J | 1.835 | 2.58E-09 |
| PPM1K | Protein phosphatase, Mg2+/Mn2+ dependent, 1K | -1.539 | 1.92E-02 |
| PPM1L | Protein phosphatase, Mg2+/Mn2+ dependent, 1L | -1.439 | 1.19E-05 |
| PPP1R15A | Protein phosphatase 1, regulatory subunit 15A | 1.703 | 6.25E-06 |
| PPP1R18 | Protein phosphatase 1, regulatory subunit 18 | 1.485 | 9.67E-04 |
| PPP1R3B | Protein phosphatase 1, regulatory subunit 3B | -1.282 | 3.06E-03 |
| PPP2CA | Protein phosphatase 2, catalytic subunit, alpha isozyme | 1.264 | 2.40E-02 |
| PPP2R4 | Protein phosphatase 2A activator, regulatory subunit 4 | 1.254 | 3.52E-03 |
| PPP2R5B | Protein phosphatase 2, regulatory subunit B', beta | 1.597 | 1.12E-07 |
| PPWD1 | Peptidylprolyl isomerase domain and WD repeat containing 1 | 1.267 | 2.04E-03 |
| PQLC1 | PQ loop repeat containing 1 | 1.308 | 2.64E-02 |
| PQLC3 | PQ loop repeat containing 3 | 1.532 | 1.18E-02 |
| PRAF2 | PRA1 domain family, member 2 | 1.509 | 5.07E-08 |
| PRC1 | Protein regulator of cytokinesis 1 | 1.702 | 8.73E-06 |
| PRCP | Prolylcarboxypeptidase (angiotensinase C) | 1.86 | 3.45E-07 |
| PRDX4 | Peroxiredoxin 4 | -1.427 | 1.98E-06 |
| PREB | Prolactin regulatory element binding | 1.268 | 1.03E-04 |
| PRELID1 | PRELI domain containing 1 | 1.433 | 2.34E-05 |
| PRELP | Proline/arginine-rich end leucine-rich repeat protein | 1.251 | 1.39E-02 |
| PREX1 | Phosphatidylinositol-3,4,5-trisphosphate-dependent Rac exchange factor 1 | 1.431 | 1.56E-02 |
| PRICKLE1 | Prickle homolog 1 | -1.437 | 5.84E-04 |
| PRKAB2 | Protein kinase, AMP-activated, beta 2 non-catalytic subunit | -1.299 | 1.87E-03 |
| PRKCDBP | Protein kinase C, delta binding protein | 1.465 | 2.65E-04 |
| PRKCZ | Protein kinase C, zeta | -1.263 | 1.81E-02 |
| PRKD2 | Protein kinase D2 | 1.348 | 1.04E-02 |
| PRKG1 | Protein kinase, cGMP-dependent, type I | -1.789 | 2.68E-06 |
| PRKRIR | Protein-kinase, interferon-inducible double stranded RNA dependent inhibitor, repressor of (P58 repressor) | -1.322 | 1.24E-02 |
| PRMT3 | Protein arginine methyltransferase 3 | 1.325 | 1.37E-02 |
| PRNP | Prion protein | 1.252 | 6.19E-03 |
| PROX1 | Prospero homeobox 1 | -1.547 | 2.43E-02 |
| PRPF18 | Pre-mRNA processing factor 18 | -1.265 | 2.32E-03 |
| PRPF19 | Pre-mRNA processing factor 19 | 1.295 | 7.61E-05 |
| PRPSAP1 | Phosphoribosyl pyrophosphate synthetase-associated protein 1 | -1.481 | 9.17E-09 |
| PRPSAP2 | Phosphoribosyl pyrophosphate synthetase-associated protein 2 | -1.379 | 2.29E-04 |
| PRUNE2 | Prune homolog 2 (Drosophila) | 2.1 | 1.82E-06 |
| PSAT1 | Phosphoserine aminotransferase 1 | 4.325 | 1.84E-10 |
| PSMC1 | Proteasome (prosome, macropain) 26S subunit, ATPase, 1 | -1.342 | 2.29E-04 |
| PSMC3 | Proteasome (prosome, macropain) 26S subunit, ATPase, 3 | -1.315 | 1.65E-03 |
| PSME4 | Proteasome (prosome, macropain) activator subunit 4 | -1.503 | 3.41E-04 |
| PSMF1 | Proteasome (prosome, macropain) inhibitor subunit 1 (PI31) | -1.276 | 1.68E-03 |
| PSPH | Phosphoserine phosphatase | 7.189 | 7.21E-12 |
| PSTPIP2 | Proline-serine-threonine phosphatase interacting protein 2 | -1.456 | 3.89E-04 |
| PTGER4 | Prostaglandin E receptor 4 (subtype EP4) | 1.297 | 9.74E-03 |
| PTGES2 | Prostaglandin E synthase 2 | 1.29 | 1.01E-02 |
| PTGS1 | Prostaglandin-endoperoxide synthase 1 (prostaglandin G/H synthase and cyclooxygenase) | -1.465 | 5.64E-03 |
| PTP4A3 | Protein tyrosine phosphatase type IVA, member 3 | -1.751 | 1.96E-10 |
| PTPN1 | Protein tyrosine phosphatase, non-receptor type 1 | -1.278 | 1.14E-03 |
| PTPN4 | Protein tyrosine phosphatase, non-receptor type 4 (megakaryocyte) | -1.808 | 2.33E-04 |
| PTPN9 | Protein tyrosine phosphatase, non-receptor type 9 | 1.39 | 1.55E-03 |
| PTPRB | Protein tyrosine phosphatase, receptor type, B | -1.518 | 2.79E-07 |
| PTPRG | Protein tyrosine phosphatase, receptor type, G | -1.462 | 4.65E-04 |
| PTPRK | Protein tyrosine phosphatase, receptor type, K | -1.472 | 1.79E-03 |
| PTPRU | Protein tyrosine phosphatase, receptor type, U | -1.934 | 9.63E-10 |
| PTRHD1 | Peptidyl-tRNA hydrolase domain containing 1 | 1.784 | 2.18E-06 |
| PURB | Purine-rich element binding protein B | -1.517 | 5.27E-04 |
| PXMP2 | Peroxisomal membrane protein 2, 22kDa | -1.64 | 2.36E-05 |
| PYCR1 | Pyrroline-5-carboxylate reductase 1 | 3.417 | 3.56E-07 |
| PYGL | Phosphorylase, glycogen, liver | 1.283 | 1.03E-02 |
| PYGM | Phosphorylase, glycogen, muscle | -1.321 | 1.39E-04 |
| QPCT | Glutaminyl-peptide cyclotransferase | 1.542 | 8.77E-04 |
| QPCTL | Glutaminyl-peptide cyclotransferase-like | 1.371 | 2.46E-02 |
| QSOX1 | Quiescin Q6 sulfhydryl oxidase 1 | 1.465 | 3.67E-05 |
| RAB11FIP2 | RAB11 family interacting protein 2 (class I) | -1.305 | 1.09E-02 |
| RAB13 | RAB13, member RAS oncogene family | 1.311 | 5.74E-03 |
| Rab15 | RAB15, member RAS oncogene family | 2.124 | 2.84E-04 |
| RAB32 | RAB32, member RAS oncogene family | -1.299 | 2.80E-02 |
| RAB4A | RAB4A, member RAS oncogene family | 1.328 | 6.16E-03 |
| RAB7B | RAB7B, member RAS oncogene family | 1.426 | 7.51E-04 |
| RABGGTA | Rab geranylgeranyltransferase, alpha subunit | -1.341 | 3.21E-05 |
| RACGAP1 | Rac GTPase activating protein 1 | 1.561 | 2.90E-06 |
| RAD1 | RAD1 checkpoint DNA exonuclease | 1.827 | 4.78E-06 |
| RADIL | Ras association and DIL domains | -1.635 | 7.93E-05 |
| RALA | V-ral simian leukemia viral oncogene homolog A (ras related) | 1.387 | 4.95E-05 |
| RAMP2 | Receptor (G protein-coupled) activity modifying protein 2 | 1.262 | 4.91E-03 |
| RANGAP1 | Ran GTPase activating protein 1 | 1.372 | 4.34E-04 |
| RANGRF | RAN guanine nucleotide release factor | -1.622 | 1.10E-04 |
| RAPGEF3 | Rap guanine nucleotide exchange factor (GEF) 3 | -1.256 | 1.42E-02 |
| RAPGEF6 | Rap guanine nucleotide exchange factor (GEF) 6 | -1.808 | 6.97E-04 |
| RASA4 | RAS p21 protein activator 4 | -1.587 | 5.87E-06 |
| RASAL1 | RAS protein activator like 1 (GAP1 like) | -1.726 | 6.38E-05 |
| RASD1 | RAS, dexamethasone-induced 1 | -1.367 | 1.02E-02 |
| RASGRF2 | Ras protein-specific guanine nucleotide-releasing factor 2 | -1.388 | 1.42E-02 |
| RASIP1 | Ras interacting protein 1 | 1.471 | 2.12E-06 |
| RBBP8 | Retinoblastoma binding protein 8 | 1.654 | 1.45E-04 |
| RBCK1 | RanBP-type and C3HC4-type zinc finger containing 1 | -1.303 | 2.65E-03 |
| RBM15 | RNA binding motif protein 15 | -1.337 | 4.33E-03 |
| RBM20 | RNA binding motif protein 20 | -1.746 | 1.56E-12 |
| RBM3 | RNA binding motif (RNP1, RRM) protein 3 | -4.225 | 3.65E-39 |
| RBM38 | RNA binding motif protein 38 | -1.41 | 1.66E-02 |
| RBMS1 | RNA binding motif, single stranded interacting protein 1 | 1.285 | 7.86E-03 |
| RC3H2 | Ring finger and CCCH-type domains 2 | -1.791 | 1.61E-02 |
| Rcan1 | Regulator of calcineurin 1 | 2.614 | 2.00E-07 |
| RCAN2 | Regulator of calcineurin 2 | 2.316 | 1.02E-04 |
| RCC2 | Regulator of chromosome condensation 2 | 1.938 | 9.23E-10 |
| RCN1 | Reticulocalbin 1, EF-hand calcium binding domain | 1.622 | 2.22E-07 |
| RCN3 | Reticulocalbin 3, EF-hand calcium binding domain | 1.749 | 5.21E-04 |
| RDH11 | Retinol dehydrogenase 11 (all-trans/9-cis/11-cis) | 1.412 | 4.28E-03 |
| RDH13 | Retinol dehydrogenase 13 (all-trans/9-cis) | 1.424 | 7.77E-03 |
| RECK | Reversion-inducing-cysteine-rich protein with kazal motifs | 1.397 | 1.43E-02 |
| REEP1 | Receptor accessory protein 1 | 1.622 | 9.08E-11 |
| RELB | V-rel avian reticuloendotheliosis viral oncogene homolog B | 1.66 | 2.89E-05 |
| RELL1 | RELT-like 1 | -1.315 | 2.16E-02 |
| REPS1 | RALBP1 associated Eps domain containing 1 | -1.471 | 1.89E-05 |
| RERE | Arginine-glutamic acid dipeptide (RE) repeats | -1.276 | 1.34E-02 |
| RET | Ret proto-oncogene | 1.596 | 1.66E-03 |
| REV3L | REV3-like, polymerase (DNA directed), zeta, catalytic subunit | -1.628 | 3.56E-06 |
| REXO4 | REX4 homolog, 3'-5' exonuclease | 1.679 | 2.16E-06 |
| RFC3 | Replication factor C (activator 1) 3, 38kDa | -1.374 | 1.67E-03 |
| RFX2 | Regulatory factor X, 2 (influences HLA class II expression) | 2.083 | 1.70E-03 |
| RFX5 | Regulatory factor X, 5 (influences HLA class II expression) | 1.449 | 1.41E-03 |
| RFXANK | Regulatory factor X-associated ankyrin-containing protein | 1.577 | 3.97E-04 |
| RGAG4 | Retrotransposon gag domain containing 4 | -1.403 | 1.32E-03 |
| RGCC | Regulator of cell cycle | -1.64 | 6.19E-04 |
| RGL2 | Ral guanine nucleotide dissociation stimulator-like 2 | -1.382 | 4.63E-06 |
| RGS14 | Regulator of G-protein signaling 14 | -1.265 | 9.50E-03 |
| RGS16 | Regulator of G-protein signaling 16 | 1.693 | 3.38E-03 |
| RGS3 | Regulator of G-protein signaling 3 | 1.295 | 1.45E-03 |
| RHBDD2 | Rhomboid domain containing 2 | -1.429 | 7.12E-04 |
| RHBDF2 | Rhomboid 5 homolog 2 (Drosophila) | 2.302 | 4.32E-10 |
| RHOB | Ras homolog family member B | 1.304 | 1.92E-03 |
| RHOD | Ras homolog family member D | 1.633 | 3.04E-04 |
| RHOG | Ras homolog family member G | 1.331 | 1.66E-03 |
| RIBC1 | RIB43A domain with coiled-coils 1 | -1.671 | 8.50E-05 |
| RIC1 | RIC1 homolog, RAB6A GEF complex partner 1 | -1.347 | 1.92E-03 |
| RIOK1 | RIO kinase 1 | 1.39 | 3.44E-03 |
| RIPK2 | Receptor-interacting serine-threonine kinase 2 | 1.635 | 1.06E-03 |
| RMND5A | Required for meiotic nuclear division 5 homolog A | -2.133 | 8.07E-10 |
| RNASE1 | Ribonuclease, RNase A family, 1 (pancreatic) | 1.851 | 8.63E-05 |
| RNASE6 | Ribonuclease, RNase A family, k6 | 1.685 | 1.97E-03 |
| RNASEH2B | Ribonuclease H2, subunit B | -1.311 | 9.22E-04 |
| RND2 | Rho family GTPase 2 | 1.589 | 9.15E-03 |
| RNF113A | Ring finger protein 113A | -1.258 | 3.91E-03 |
| RNF123 | Ring finger protein 123 | -1.342 | 6.83E-04 |
| RNF130 | Ring finger protein 130 | 1.371 | 3.67E-05 |
| RNF144B | Ring finger protein 144B | -1.357 | 1.22E-03 |
| RNF145 | Ring finger protein 145 | -1.523 | 9.05E-06 |
| RNF207 | Ring finger protein 207 | 1.329 | 7.95E-03 |
| RNF208 | Ring finger protein 208 | -1.638 | 4.67E-04 |
| RNF41 | Ring finger protein 41, E3 ubiquitin protein ligase | 1.261 | 1.52E-02 |
| RNF7 | Ring finger protein 7 | -1.32 | 1.17E-04 |
| RNFT2 | Ring finger protein, transmembrane 2 | 1.613 | 5.31E-03 |
| RNH1 | Ribonuclease/angiogenin inhibitor 1 | -1.294 | 7.73E-04 |
| RNPC3 | RNA-binding region (RNP1, RRM) containing 3 | 1.722 | 4.68E-04 |
| ROBO1 | Roundabout guidance receptor 1 | 1.35 | 8.55E-03 |
| ROBO4 | Roundabout guidance receptor 4 | 1.322 | 9.19E-03 |
| RORA | RAR-related orphan receptor A | -1.737 | 3.58E-06 |
| RORC | RAR-related orphan receptor C | -1.633 | 1.66E-02 |
| RPE | Ribulose-5-phosphate-3-epimerase | 1.674 | 4.51E-05 |
| RPF2 | Ribosome production factor 2 homolog | 1.488 | 2.53E-05 |
| RPL22 | Ribosomal protein L22 | 1.259 | 7.31E-03 |
| RPL3 | Ribosomal protein L3 | 2.178 | 1.86E-16 |
| RPL32 | Ribosomal protein L32 | 1.254 | 2.43E-02 |
| RPL3L | Ribosomal protein L3-like | -1.647 | 9.30E-10 |
| RPL6 | Ribosomal protein L6 | 1.257 | 1.06E-02 |
| RPL7 | Ribosomal protein L7 | 1.254 | 1.88E-02 |
| RPL8 | Ribosomal protein L8 | 1.264 | 7.60E-03 |
| RPP40 | Ribonuclease P/MRP 40kDa subunit | 1.524 | 1.99E-04 |
| RPS4Y2 | Ribosomal protein S4, Y-linked 2 | 1.347 | 2.91E-05 |
| RPS6KA1 | Ribosomal protein S6 kinase, 90kDa, polypeptide 1 | 1.696 | 2.50E-06 |
| RPS6KA5 | Ribosomal protein S6 kinase, 90kDa, polypeptide 5 | -1.57 | 2.95E-05 |
| RPUSD2 | RNA pseudouridylate synthase domain containing 2 | -1.295 | 1.81E-02 |
| RRAGB | Ras-related GTP binding B | -1.578 | 9.51E-04 |
| RRAGD | Ras-related GTP binding D | -1.25 | 4.30E-04 |
| RRAS2 | Related RAS viral (r-ras) oncogene homolog 2 | 1.359 | 1.96E-03 |
| Rrbp1 | Ribosome binding protein 1 | 1.701 | 2.18E-09 |
| RRP15 | Ribosomal RNA processing 15 homolog | 1.3 | 7.58E-04 |
| RRP1B | Ribosomal RNA processing 1B | -1.261 | 6.88E-03 |
| RRS1 | Ribosome biogenesis regulator homolog | 1.28 | 3.60E-03 |
| RSAD1 | Radical S-adenosyl methionine domain containing 1 | 1.258 | 1.34E-02 |
| RSBN1 | Round spermatid basic protein 1 | -1.464 | 1.51E-03 |
| RSL1D1 | Ribosomal L1 domain containing 1 | 1.346 | 1.12E-03 |
| RSPO3 | R-spondin 3 | 2.097 | 2.07E-04 |
| RSRC1 | Arginine/serine-rich coiled-coil 1 | 1.436 | 1.78E-04 |
| RSU1 | Ras suppressor protein 1 | 1.468 | 1.61E-06 |
| RTEL1 | Regulator of telomere elongation helicase 1 | 1.398 | 1.19E-02 |
| RTF1 | RTF1 homolog, Paf1/RNA polymerase II complex component | -1.257 | 1.36E-03 |
| RTN4IP1 | Reticulon 4 interacting protein 1 | 1.442 | 2.13E-04 |
| RUFY2 | RUN and FYVE domain containing 2 | -1.372 | 1.14E-02 |
| RUNDC1 | RUN domain containing 1 | 1.357 | 6.15E-04 |
| RUSC2 | RUN and SH3 domain containing 2 | -1.679 | 7.61E-10 |
| RYR1 | Ryanodine receptor 1 (skeletal) | -1.551 | 3.84E-09 |
| S100A10 | S100 calcium binding protein A10 | 1.409 | 7.43E-03 |
| S100A11 | S100 calcium binding protein A11 | 1.904 | 1.08E-06 |
| S100A14 | S100 calcium binding protein A14 | 2.057 | 6.96E-09 |
| S100A16 | S100 calcium binding protein A16 | 1.502 | 1.07E-07 |
| S100A2 | S100 calcium binding protein A2 | 1.438 | 1.14E-03 |
| S100B | S100 calcium binding protein B | 1.355 | 1.27E-03 |
| S1PR1 | Sphingosine-1-phosphate receptor 1 | -1.27 | 1.09E-03 |
| S1PR3 | Sphingosine-1-phosphate receptor 3 | 1.271 | 2.37E-02 |
| SAAL1 | Serum amyloid A-like 1 | -1.772 | 1.28E-05 |
| SAMD4A | Sterile alpha motif domain containing 4A | -1.607 | 4.22E-09 |
| SARS | Seryl-tRNA synthetase | 1.892 | 3.41E-05 |
| SART1 | Squamous cell carcinoma antigen recognized by T cells 1 | -1.266 | 9.66E-03 |
| SASH1 | SAM and SH3 domain containing 1 | -1.482 | 9.46E-05 |
| SAT2 | Spermidine/spermine N1-acetyltransferase family member 2 | -1.292 | 8.03E-04 |
| SATB1 | SATB homeobox 1 | -1.529 | 1.84E-08 |
| SBF2 | SET binding factor 2 | -1.442 | 9.04E-05 |
| SBK1 | SH3 domain binding kinase 1 | 1.691 | 1.10E-04 |
| SBNO1 | Strawberry notch homolog 1 (Drosophila) | -1.402 | 4.40E-03 |
| SCAPER | S-phase cyclin A-associated protein in the ER | -1.413 | 4.76E-05 |
| SCARA3 | Scavenger receptor class A, member 3 | 1.696 | 1.48E-04 |
| SCARA5 | Scavenger receptor class A, member 5 | 1.303 | 1.93E-03 |
| SCARB1 | Scavenger receptor class B, member 1 | 1.413 | 4.84E-04 |
| SCIN | Scinderin | 1.696 | 5.49E-05 |
| SCMH1 | Sex comb on midleg homolog 1 (Drosophila) | -1.301 | 1.07E-04 |
| SCN4A | Sodium channel, voltage gated, type IV alpha subunit | -1.258 | 1.29E-03 |
| SCUBE2 | Signal peptide, CUB domain, EGF-like 2 | -1.675 | 4.93E-04 |
| SDC4 | Syndecan 4 | 1.255 | 2.76E-02 |
| SDHAF3 | Succinate dehydrogenase complex assembly factor 3 | -1.284 | 2.23E-02 |
| SDPR | Serum deprivation response | 1.37 | 4.90E-03 |
| SDS | Serine dehydratase | 2.407 | 2.76E-02 |
| SEC14L1 | SEC14-like lipid binding 1 | 1.268 | 8.58E-03 |
| SEC22C | SEC22 vesicle trafficking protein homolog C (S. cerevisiae) | -1.404 | 3.97E-04 |
| SEC24B | SEC24 family member B | -1.297 | 3.37E-03 |
| SEC24D | SEC24 family member D | 1.651 | 2.25E-05 |
| SEC31A | SEC31 homolog A, COPII coating complex component | 1.264 | 5.75E-04 |
| SEC61A2 | Sec61 alpha 2 subunit (S. cerevisiae) | 1.346 | 9.05E-06 |
| SEL1L3 | Sel-1 suppressor of lin-12-like 3 (C. elegans) | 1.271 | 3.18E-03 |
| SELENBP1 | Selenium binding protein 1 | -1.386 | 4.82E-04 |
| SELK | Selenoprotein K | -1.386 | 2.16E-03 |
| SELT | Selenoprotein T | 1.566 | 5.44E-05 |
| SEMA3G | Sema domain, immunoglobulin domain (Ig), short basic domain, secreted, (semaphorin) 3G | -1.349 | 7.28E-03 |
| SEMA6B | Sema domain, transmembrane domain (TM), and cytoplasmic domain, (semaphorin) 6B | 1.611 | 2.09E-04 |
| SEMA6C | Sema domain, transmembrane domain (TM), and cytoplasmic domain, (semaphorin) 6C | 1.306 | 1.54E-03 |
| SEMA6D | Sema domain, transmembrane domain (TM), and cytoplasmic domain, (semaphorin) 6D | -1.623 | 1.71E-05 |
| SEMA7A | Semaphorin 7A, GPI membrane anchor (John Milton Hagen blood group) | -1.369 | 3.59E-04 |
| SENP6 | SUMO1/sentrin specific peptidase 6 | 1.324 | 2.43E-02 |
| SEPSECS | Sep (O-phosphoserine) tRNA:Sec (selenocysteine) tRNA synthase | -1.54 | 1.85E-02 |
| SERF2 | Small EDRK-rich factor 2 | 1.393 | 1.80E-05 |
| SERINC2 | Serine incorporator 2 | -1.425 | 3.09E-04 |
| SERPINA5 | Serpin peptidase inhibitor, clade A (alpha-1 antiproteinase, antitrypsin), member 5 | -1.364 | 1.86E-02 |
| SERPINE1 | Serpin peptidase inhibitor, clade E (nexin, plasminogen activator inhibitor type 1), member 1 | 1.901 | 4.69E-03 |
| SERPINE2 | Serpin peptidase inhibitor, clade E (nexin, plasminogen activator inhibitor type 1), member 2 | 1.735 | 1.12E-05 |
| SERPINF1 | Serpin peptidase inhibitor, clade F (alpha-2 antiplasmin, pigment epithelium derived factor), member 1 | 1.682 | 1.86E-08 |
| SERPINH1 | Serpin peptidase inhibitor, clade H (heat shock protein 47), member 1, (collagen binding protein 1) | 2.417 | 1.80E-17 |
| SERTAD3 | SERTA domain containing 3 | 1.511 | 3.00E-05 |
| SESN1 | Sestrin 1 | -1.591 | 1.85E-03 |
| SETBP1 | SET binding protein 1 | -1.718 | 1.96E-04 |
| SETD3 | SET domain containing 3 | -1.289 | 2.79E-04 |
| SETD4 | SET domain containing 4 | 1.782 | 2.24E-08 |
| SF3B5 | Splicing factor 3b, subunit 5, 10kDa | 1.514 | 8.65E-06 |
| SFXN3 | Sideroflexin 3 | 1.593 | 5.28E-04 |
| SGCG | Sarcoglycan, gamma (35kDa dystrophin-associated glycoprotein) | 1.286 | 2.92E-04 |
| SGMS1 | Sphingomyelin synthase 1 | -1.358 | 3.42E-04 |
| SGSM3 | Small G protein signaling modulator 3 | -1.268 | 5.99E-03 |
| SH2B3 | SH2B adaptor protein 3 | 1.553 | 9.80E-05 |
| SH2D3C | SH2 domain containing 3C | 1.446 | 1.41E-03 |
| SH3BGRL | SH3 domain binding glutamate-rich protein like | 1.339 | 2.86E-02 |
| SH3BP5 | SH3-domain binding protein 5 (BTK-associated) | -1.257 | 2.59E-02 |
| SH3GL1 | SH3-domain GRB2-like 1 | 1.269 | 2.27E-02 |
| SH3PXD2B | SH3 and PX domains 2B | 1.366 | 2.61E-02 |
| SH3RF3 | SH3 domain containing ring finger 3 | -1.377 | 1.18E-03 |
| SHB | Src homology 2 domain containing adaptor protein B | 1.42 | 2.59E-02 |
| SHISA2 | Shisa family member 2 | -1.525 | 3.32E-04 |
| SHKBP1 | SH3KBP1 binding protein 1 | 1.265 | 1.65E-02 |
| SHMT1 | Serine hydroxymethyltransferase 1 (soluble) | -1.433 | 1.93E-03 |
| SHMT2 | Serine hydroxymethyltransferase 2 (mitochondrial) | 1.269 | 4.67E-03 |
| SHPRH | SNF2 histone linker PHD RING helicase, E3 ubiquitin protein ligase | -1.264 | 2.67E-02 |
| SHQ1 | SHQ1, H/ACA ribonucleoprotein assembly factor | 1.457 | 2.92E-03 |
| SIGMAR1 | Sigma non-opioid intracellular receptor 1 | 1.385 | 8.49E-04 |
| SIM2 | Single-minded family bHLH transcription factor 2 | 1.878 | 5.13E-08 |
| SIPA1 | Signal-induced proliferation-associated 1 | 1.258 | 1.10E-02 |
| SIRPA | Signal-regulatory protein alpha | 1.519 | 7.54E-03 |
| SIX1 | SIX homeobox 1 | 1.273 | 5.49E-05 |
| SIX2 | SIX homeobox 2 | 2.243 | 1.35E-06 |
| SKAP1 | Src kinase associated phosphoprotein 1 | 1.672 | 3.10E-03 |
| SKAP2 | Src kinase associated phosphoprotein 2 | 1.323 | 3.39E-03 |
| SKI | SKI proto-oncogene | -1.368 | 6.82E-04 |
| SKIV2L | Superkiller viralicidic activity 2-like (S. cerevisiae) | -1.276 | 9.33E-04 |
| SLC10A3 | Solute carrier family 10, member 3 | 1.266 | 3.26E-03 |
| SLC12A2 | Solute carrier family 12 (sodium/potassium/chloride transporter), member 2 | -1.368 | 2.21E-02 |
| SLC15A4 | Solute carrier family 15 (oligopeptide transporter), member 4 | 1.261 | 3.70E-03 |
| SLC16A13 | Solute carrier family 16, member 13 | 1.696 | 3.95E-03 |
| SLC16A6 | Solute carrier family 16, member 6 | 1.811 | 8.09E-03 |
| SLC1A4 | Solute carrier family 1 (glutamate/neutral amino acid transporter), member 4 | 1.638 | 8.13E-06 |
| SLC22A17 | Solute carrier family 22, member 17 | 1.429 | 2.19E-02 |
| SLC22A23 | Solute carrier family 22, member 23 | 1.52 | 2.04E-04 |
| SLC22A4 | Solute carrier family 22 (organic cation/zwitterion transporter), member 4 | -1.871 | 3.52E-05 |
| SLC22A5 | Solute carrier family 22 (organic cation/carnitine transporter), member 5 | 1.254 | 9.36E-03 |
| SLC25A11 | Solute carrier family 25 (mitochondrial carrier; oxoglutarate carrier), member 11 | -1.256 | 1.61E-02 |
| SLC25A12 | Solute carrier family 25 (aspartate/glutamate carrier), member 12 | -1.283 | 1.80E-03 |
| SLC25A15 | Solute carrier family 25 (mitochondrial carrier; ornithine transporter) member 15 | 1.406 | 2.14E-04 |
| SLC25A20 | Solute carrier family 25 (carnitine/acylcarnitine translocase), member 20 | 1.273 | 1.12E-02 |
| SLC25A28 | Solute carrier family 25 (mitochondrial iron transporter), member 28 | -1.357 | 2.60E-04 |
| SLC25A30 | Solute carrier family 25, member 30 | 1.2523 | 2.38E-03 |
| SLC25A39 | Solute carrier family 25, member 39 | 1.289 | 1.51E-03 |
| SLC25A5 | Solute carrier family 25 (mitochondrial carrier; adenine nucleotide translocator), member 5 | 2.059 | 2.03E-08 |
| SLC26A6 | Solute carrier family 26 (anion exchanger), member 6 | -1.322 | 2.18E-03 |
| SLC27A6 | Solute carrier family 27 (fatty acid transporter), member 6 | -7.33 | 4.26E-16 |
| SLC2A4 | Solute carrier family 2 (facilitated glucose transporter), member 4 | -1.427 | 3.32E-07 |
| SLC2A4RG | SLC2A4 regulator | -1.649 | 5.89E-06 |
| SLC2A8 | Solute carrier family 2 (facilitated glucose transporter), member 8 | -1.402 | 8.49E-03 |
| SLC30A1 | Solute carrier family 30 (zinc transporter), member 1 | -1.388 | 7.31E-03 |
| SLC31A1 | Solute carrier family 31 (copper transporter), member 1 | 1.251 | 1.53E-02 |
| SLC35E3 | Solute carrier family 35, member E3 | 1.398 | 2.70E-03 |
| SLC35E4 | Solute carrier family 35, member E4 | 1.379 | 1.77E-02 |
| SLC37A4 | Solute carrier family 37 (glucose-6-phosphate transporter), member 4 | 1.301 | 6.75E-04 |
| SLC38A2 | Solute carrier family 38, member 2 | 1.535 | 2.60E-03 |
| SLC38A4 | Solute carrier family 38, member 4 | 1.758 | 1.64E-02 |
| SLC39A1 | Solute carrier family 39 (zinc transporter), member 1 | 1.291 | 2.19E-04 |
| SLC39A14 | Solute carrier family 39 (zinc transporter), member 14 | 1.325 | 2.65E-03 |
| SLC39A6 | Solute carrier family 39 (zinc transporter), member 6 | 1.266 | 9.98E-03 |
| SLC3A2 | Solute carrier family 3 (amino acid transporter heavy chain), member 2 | 1.446 | 5.85E-04 |
| SLC40A1 | Solute carrier family 40 (iron-regulated transporter), member 1 | -1.335 | 2.50E-03 |
| SLC6A8 | Solute carrier family 6 (neurotransmitter transporter), member 8 | -1.261 | 4.10E-03 |
| SLC8A3 | Solute carrier family 8 (sodium/calcium exchanger), member 3 | -1.431 | 1.43E-05 |
| SLC9A2 | Solute carrier family 9, subfamily A (NHE2, cation proton antiporter 2), member 2 | 1.325 | 7.52E-03 |
| SLC9A5 | Solute carrier family 9, subfamily A (NHE5, cation proton antiporter 5), member 5 | 1.587 | 2.47E-04 |
| SLC9A9 | Solute carrier family 9, subfamily A (NHE9, cation proton antiporter 9), member 9 | 1.399 | 1.07E-02 |
| SLCO2A1 | Solute carrier organic anion transporter family, member 2A1 | 1.508 | 2.42E-03 |
| SLCO3A1 | Solute carrier organic anion transporter family, member 3A1 | -1.342 | 9.47E-05 |
| SLF1 | SMC5-SMC6 complex localization factor 1 | -1.421 | 2.38E-02 |
| SLIT3 | Slit guidance ligand 3 | 1.609 | 1.22E-03 |
| SLITRK5 | SLIT and NTRK-like family, member 5 | -1.568 | 4.14E-03 |
| SLK | STE20-like kinase | -1.523 | 4.87E-03 |
| SMAP2 | Small ArfGAP2 | 1.56 | 7.70E-05 |
| SMARCA2 | SWI/SNF related, matrix associated, actin dependent regulator of chromatin, subfamily a, member 2 | -1.28 | 7.01E-05 |
| SMARCC2 | SWI/SNF related, matrix associated, actin dependent regulator of chromatin, subfamily c, member 2 | -1.341 | 1.06E-05 |
| SMCHD1 | Structural maintenance of chromosomes flexible hinge domain containing 1 | -1.372 | 2.09E-03 |
| SMCO1 | Single-pass membrane protein with coiled-coil domains 1 | -1.54 | 5.37E-06 |
| SMG1 | SMG1 phosphatidylinositol 3-kinase-related kinase | -1.875 | 2.39E-04 |
| SMPDL3A | Sphingomyelin phosphodiesterase, acid-like 3A | 1.838 | 5.27E-05 |
| SMPX | Small muscle protein, X-linked | 1.405 | 5.69E-03 |
| SMTNL1 | Smoothelin-like 1 | -1.722 | 2.12E-05 |
| SMTNL2 | Smoothelin-like 2 | -1.364 | 8.65E-05 |
| SMYD1 | SET and MYND domain containing 1 | 1.251 | 1.11E-02 |
| SMYD3 | SET and MYND domain containing 3 | -1.317 | 7.49E-04 |
| SNAI2 | Snail family zinc finger 2 | 1.853 | 1.15E-06 |
| SNAI3 | Snail family zinc finger 3 | -1.514 | 2.11E-03 |
| SNAPC2 | Small nuclear RNA activating complex, polypeptide 2, 45kDa | 1.304 | 6.17E-03 |
| SNAPC5 | Small nuclear RNA activating complex, polypeptide 5, 19kDa | 1.343 | 2.22E-02 |
| SNAPIN | SNAP-associated protein | -1.282 | 1.05E-04 |
| SNN | Stannin | -1.578 | 1.96E-06 |
| SNRNP200 | Small nuclear ribonucleoprotein 200kDa (U5) | -1.258 | 6.72E-04 |
| SNRPD1 | Small nuclear ribonucleoprotein D1 polypeptide 16kDa | 1.336 | 1.98E-03 |
| SNTA1 | Syntrophin, alpha 1 | -1.254 | 1.21E-02 |
| SNTB1 | Syntrophin, beta 1 (dystrophin-associated protein A1, 59kDa, basic component 1) | -1.537 | 1.19E-04 |
| SNX1 | Sorting nexin 1 | -1.484 | 1.31E-07 |
| SNX27 | Sorting nexin family member 27 | -1.309 | 6.22E-05 |
| SNX3 | Sorting nexin 3 | 1.394 | 2.62E-05 |
| SNX8 | Sorting nexin 8 | -1.626 | 2.93E-05 |
| SOCS2 | Suppressor of cytokine signaling 2 | -1.649 | 2.96E-03 |
| SOD3 | Superoxide dismutase 3, extracellular | -1.473 | 6.52E-04 |
| SON | SON DNA binding protein | -1.291 | 1.42E-02 |
| SORBS1 | Sorbin and SH3 domain containing 1 | -1.783 | 1.11E-10 |
| SORBS3 | Sorbin and SH3 domain containing 3 | -1.274 | 2.12E-02 |
| SORT1 | Sortilin 1 | -1.322 | 5.61E-04 |
| SOX13 | SRY (sex determining region Y)-box 13 | -1.286 | 2.61E-02 |
| SOX6 | SRY (sex determining region Y)-box 6 | -1.667 | 8.62E-04 |
| SP4 | Sp4 transcription factor | -2.432 | 2.07E-10 |
| SPARC | Secreted protein, acidic, cysteine-rich (osteonectin) | 1.558 | 9.05E-05 |
| SPATA7 | Spermatogenesis associated 7 | -1.47 | 2.89E-03 |
| SPATS2 | Spermatogenesis associated, serine-rich 2 | 2.05 | 6.15E-07 |
| SPEG | SPEG complex locus | -1.31 | 4.15E-03 |
| SPG21 | Spastic paraplegia 21 (autosomal recessive, Mast syndrome) | 1.476 | 3.94E-04 |
| SPINT2 | Serine peptidase inhibitor, Kunitz type, 2 | -1.545 | 1.56E-06 |
| SPON1 | Spondin 1, extracellular matrix protein | 1.625 | 8.76E-05 |
| SPON2 | Spondin 2, extracellular matrix protein | 1.58 | 5.96E-05 |
| SPRTN | SprT-like N-terminal domain | -1.252 | 2.58E-03 |
| SPRY4 | Sprouty RTK signaling antagonist 4 | 2.284 | 1.92E-08 |
| SPSB1 | SplA/ryanodine receptor domain and SOCS box containing 1 | -1.531 | 3.18E-05 |
| SPSB3 | SplA/ryanodine receptor domain and SOCS box containing 3 | -1.502 | 9.71E-05 |
| SQLE | Squalene epoxidase | 2.521 | 4.21E-06 |
| SREBF1 | Sterol regulatory element binding transcription factor 1 | -1.546 | 3.31E-05 |
| SRI | Sorcin | 1.396 | 1.06E-03 |
| SRPK3 | SRSF protein kinase 3 | 1.258 | 5.99E-03 |
| SRPR | Signal recognition particle receptor (docking protein) | 1.266 | 2.13E-02 |
| SRXN1 | Sulfiredoxin 1 | 2.008 | 2.37E-05 |
| SSH2 | Slingshot protein phosphatase 2 | -1.262 | 2.13E-03 |
| SSX2IP | Synovial sarcoma, X breakpoint 2 interacting protein | -1.289 | 1.54E-02 |
| ST6GALNAC4 | ST6 (alpha-N-acetyl-neuraminyl-2,3-beta-galactosyl-1,3)-N-acetylgalactosaminide alpha-2,6-sialyltransferase 4 | 2.039 | 9.89E-12 |
| ST8SIA2 | ST8 alpha-N-acetyl-neuraminide alpha-2,8-sialyltransferase 2 | 1.531 | 1.22E-02 |
| ST8SIA5 | ST8 alpha-N-acetyl-neuraminide alpha-2,8-sialyltransferase 5 | 1.553 | 3.47E-03 |
| STAB1 | Stabilin 1 | 1.758 | 1.38E-06 |
| STAG1 | Stromal antigen 1 | -1.391 | 3.27E-03 |
| STAG2 | Stromal antigen 2 | -1.312 | 2.18E-02 |
| STAMBPL1 | STAM binding protein-like 1 | 1.262 | 1.36E-03 |
| STAP2 | Signal transducing adaptor family member 2 | 1.49 | 6.75E-03 |
| STARD4 | StAR-related lipid transfer (START) domain containing 4 | 1.443 | 8.58E-03 |
| STAT5A | Signal transducer and activator of transcription 5A | -1.426 | 3.36E-06 |
| STAT5B | Signal transducer and activator of transcription 5B | -2.052 | 3.71E-18 |
| STEAP4 | STEAP family member 4 | -1.673 | 1.13E-02 |
| STIP1 | Stress-induced phosphoprotein 1 | 1.279 | 5.18E-04 |
| STK10 | Serine/threonine kinase 10 | 1.254 | 2.49E-02 |
| STK38L | Serine/threonine kinase 38 like | -1.292 | 2.57E-02 |
| STMN1 | Stathmin 1 | 1.956 | 2.77E-06 |
| STMN2 | Stathmin 2 | -2.954 | 6.66E-09 |
| STRA8 | Stimulated by retinoic acid 8 | -1.44 | 2.45E-02 |
| STRN | Striatin, calmodulin binding protein | -1.454 | 2.61E-03 |
| STX6 | Syntaxin 6 | 1.28 | 1.69E-02 |
| STXBP1 | Syntaxin binding protein 1 | 1.281 | 2.65E-02 |
| SUPT3H | Suppressor of Ty 3 homolog (S. cerevisiae) | 1.73 | 3.39E-05 |
| SUV39H1 | Suppressor of variegation 3-9 homolog 1 (Drosophila) | -2.023 | 7.70E-10 |
| SVBP | Small vasohibin binding protein | -1.283 | 2.23E-02 |
| SWI5 | SWI5 homologous recombination repair protein | -1.421 | 1.72E-06 |
| SWT1 | SWT1 RNA endoribonuclease homolog (S. cerevisiae) | -1.336 | 2.12E-02 |
| SYCP3 | Synaptonemal complex protein 3 | 1.583 | 7.55E-04 |
| SYMPK | Symplekin | -1.27 | 1.37E-03 |
| SYN2 | Synapsin II | -1.369 | 1.52E-02 |
| SYNE3 | Spectrin repeat containing, nuclear envelope family member 3 | -1.276 | 2.58E-02 |
| SYNM | Synemin, intermediate filament protein | -1.631 | 3.25E-11 |
| SYPL2 | Synaptophysin-like 2 | 1.259 | 1.61E-03 |
| SYS1 | Sys1 golgi trafficking protein | 1.254 | 2.26E-02 |
| SYT7 | Synaptotagmin VII | 1.717 | 1.23E-03 |
| TACC1 | Transforming, acidic coiled-coil containing protein 1 | -1.413 | 5.32E-06 |
| TACO1 | Translational activator of mitochondrially encoded cytochrome c oxidase I | -1.333 | 1.19E-03 |
| TAF1 | TAF1 RNA polymerase II, TATA box binding protein (TBP)-associated factor, 250kDa | 1.561 | 2.32E-07 |
| TAF13 | TAF13 RNA polymerase II, TATA box binding protein (TBP)-associated factor, 18kDa | 1.346 | 1.37E-02 |
| TAF4 | TAF4 RNA polymerase II, TATA box binding protein (TBP)-associated factor, 135kDa | -1.268 | 1.24E-03 |
| TAF7 | TAF7 RNA polymerase II, TATA box binding protein (TBP)-associated factor, 55kDa | -1.282 | 1.66E-02 |
| TAF8 | TAF8 RNA polymerase II, TATA box binding protein (TBP)-associated factor, 43kDa | 1.269 | 4.28E-03 |
| TAF9B | TAF9B RNA polymerase II, TATA box binding protein (TBP)-associated factor, 31kDa | -1.308 | 5.93E-03 |
| TAGLN2 | Transgelin 2 | 1.685 | 4.79E-07 |
| TAGLN3 | Transgelin 3 | 1.409 | 2.14E-02 |
| TANC1 | Tetratricopeptide repeat, ankyrin repeat and coiled-coil containing 1 | -1.337 | 1.17E-02 |
| TANGO2 | Transport and golgi organization 2 homolog | 1.49 | 4.78E-03 |
| TAP1 | Transporter 1, ATP-binding cassette, sub-family B (MDR/TAP) | 1.469 | 7.06E-03 |
| TAPT1 | Transmembrane anterior posterior transformation 1 | -1.256 | 1.37E-03 |
| TARDBP | TAR DNA binding protein | -1.302 | 4.37E-03 |
| TARS | Threonyl-tRNA synthetase | 1.384 | 5.83E-05 |
| TATDN1 | TatD DNase domain containing 1 | -1.262 | 1.18E-02 |
| TAX1BP3 | Tax1 (human T-cell leukemia virus type I) binding protein 3 | 1.415 | 3.94E-04 |
| TBC1D17 | TBC1 domain family, member 17 | -1.537 | 1.75E-06 |
| TBC1D7 | TBC1 domain family, member 7 | 1.263 | 1.28E-02 |
| TCAP | Titin-cap | -1.364 | 2.09E-04 |
| TCEA2 | Transcription elongation factor A (SII), 2 | -1.437 | 1.16E-04 |
| TCEA3 | Transcription elongation factor A (SII), 3 | -1.714 | 7.46E-13 |
| TCOF1 | Treacher Collins-Franceschetti syndrome 1 | -1.254 | 5.07E-03 |
| TCP11L2 | T-complex 11, testis-specific-like 2 | -1.281 | 1.69E-02 |
| TCTN1 | Tectonic family member 1 | 1.272 | 1.43E-02 |
| TDRD7 | Tudor domain containing 7 | -1.306 | 5.30E-03 |
| TEAD1 | TEA domain family member 1 (SV40 transcriptional enhancer factor) | -1.497 | 3.13E-03 |
| TENM3 | Teneurin transmembrane protein 3 | -1.407 | 7.82E-03 |
| TET2 | Tet methylcytosine dioxygenase 2 | -1.561 | 1.10E-03 |
| TFAM | Transcription factor A, mitochondrial | -1.284 | 3.25E-03 |
| TFAP4 | Transcription factor AP-4 (activating enhancer binding protein 4) | -1.833 | 1.57E-04 |
| TFB2M | Transcription factor B2, mitochondrial | 1.252 | 1.13E-02 |
| TGFB1 | Transforming growth factor, beta 1 | 1.371 | 4.00E-03 |
| TGFB1I1 | Transforming growth factor beta 1 induced transcript 1 | 1.275 | 2.02E-02 |
| TGFB3 | Transforming growth factor, beta 3 | 1.541 | 3.44E-03 |
| TGFBR1 | Transforming growth factor, beta receptor 1 | -1.755 | 1.96E-03 |
| TGFBR3 | Transforming growth factor, beta receptor III | -1.463 | 1.14E-04 |
| TGIF1 | TGFB-induced factor homeobox 1 | 1.358 | 2.53E-02 |
| TGM2 | Transglutaminase 2 | 1.3 | 7.56E-03 |
| THADA | Thyroid adenoma associated | 1.544 | 9.26E-06 |
| THAP2 | THAP domain containing, apoptosis associated protein 2 | 1.505 | 2.51E-02 |
| THAP8 | THAP domain containing 8 | 1.609 | 2.37E-04 |
| THBS1 | Thrombospondin 1 | 1.599 | 7.20E-04 |
| THNSL2 | Threonine synthase-like 2 (S. cerevisiae) | -1.652 | 5.92E-03 |
| THOC3 | THO complex 3 | 1.466 | 2.56E-05 |
| THOC6 | THO complex 6 | 1.341 | 1.68E-02 |
| THUMPD2 | THUMP domain containing 2 | -1.337 | 1.35E-02 |
| THY1 | Thy-1 cell surface antigen | 1.698 | 6.27E-07 |
| TIA1 | TIA1 cytotoxic granule-associated RNA binding protein | -1.32 | 2.23E-02 |
| TICAM1 | Toll-like receptor adaptor molecule 1 | 1.481 | 3.09E-03 |
| TIGD2 | Tigger transposable element derived 2 | 1.331 | 2.19E-02 |
| TIMELESS | Timeless circadian clock | 1.553 | 1.86E-05 |
| TIMM8B | Translocase of inner mitochondrial membrane 8 homolog B (yeast) | -1.302 | 1.93E-03 |
| TIMP1 | TIMP metallopeptidase inhibitor 1 | 1.479 | 3.79E-05 |
| TIPARP | TCDD-inducible poly(ADP-ribose) polymerase | 1.496 | 7.75E-04 |
| TIPIN | TIMELESS interacting protein | 1.382 | 1.17E-02 |
| TLE2 | Transducin-like enhancer of split 2 | 1.71 | 3.55E-05 |
| TLE4 | Transducin-like enhancer of split 4 | 1.282 | 1.45E-02 |
| TLK1 | Tousled-like kinase 1 | 1.327 | 4.80E-03 |
| TLN2 | Talin 2 | -1.439 | 3.48E-07 |
| TM2D1 | TM2 domain containing 1 | 1.583 | 8.69E-09 |
| TM4SF18 | Transmembrane 4 L six family member 18 | 4.665 | 6.85E-15 |
| TMBIM1 | Transmembrane BAX inhibitor motif containing 1 | -1.292 | 1.40E-03 |
| TMED1 | Transmembrane emp24 protein transport domain containing 1 | 1.645 | 1.17E-07 |
| TMED3 | Transmembrane emp24 protein transport domain containing 3 | 2.128 | 9.71E-08 |
| TMED4 | Transmembrane emp24 protein transport domain containing 4 | 1.254 | 2.44E-02 |
| TMED5 | Transmembrane emp24 protein transport domain containing 5 | 1.427 | 2.19E-02 |
| TMED6 | Transmembrane emp24 protein transport domain containing 6 | 1.549 | 3.87E-03 |
| TMEM101 | Transmembrane protein 101 | 1.348 | 7.20E-05 |
| TMEM126B | Transmembrane protein 126B | 1.283 | 2.28E-02 |
| TMEM135 | Transmembrane protein 135 | 1.514 | 2.89E-04 |
| TMEM140 | Transmembrane protein 140 | -1.698 | 2.48E-06 |
| TMEM145 | Transmembrane protein 145 | -1.39 | 1.28E-03 |
| TMEM150A | Transmembrane protein 150A | 1.362 | 7.02E-03 |
| TMEM176B | Transmembrane protein 176B | 1.782 | 1.11E-05 |
| TMEM178A | Transmembrane protein 178A | -1.33 | 1.16E-02 |
| TMEM185A | Transmembrane protein 185A | -1.299 | 2.00E-04 |
| TMEM189 | Transmembrane protein 189 | 1.375 | 1.07E-04 |
| TMEM208 | Transmembrane protein 208 | 1.272 | 6.65E-03 |
| TMEM231 | Transmembrane protein 231 | -1.417 | 6.24E-03 |
| TMEM243 | Transmembrane protein 243, mitochondrial | -1.33 | 1.31E-03 |
| TMEM245 | Transmembrane protein 245 | -2.087 | 3.14E-06 |
| TMEM255A | Transmembrane protein 255A | -3.612 | 4.94E-10 |
| TMEM260 | Transmembrane protein 260 | 1.815 | 4.14E-08 |
| TMEM33 | Transmembrane protein 33 | 1.28 | 2.03E-02 |
| TMEM35 | Transmembrane protein 35 | 1.47 | 6.49E-03 |
| TMEM51 | Transmembrane protein 51 | 1.306 | 1.36E-02 |
| TMEM60 | Transmembrane protein 60 | -1.401 | 1.30E-02 |
| TMEM63A | Transmembrane protein 63A | -1.264 | 1.89E-03 |
| TMEM87B | Transmembrane protein 87B | -1.412 | 2.52E-02 |
| TMOD1 | Tropomodulin 1 | 1.273 | 5.85E-03 |
| TNFAIP1 | Tumor necrosis factor, alpha-induced protein 1 (endothelial) | 1.341 | 2.56E-05 |
| TNFAIP8L1 | Tumor necrosis factor, alpha-induced protein 8-like 1 | 1.576 | 5.64E-04 |
| TNFRSF12A | Tumor necrosis factor receptor superfamily, member 12A | 5.74 | 2.45E-13 |
| TNFRSF21 | Tumor necrosis factor receptor superfamily, member 21 | -1.638 | 2.60E-06 |
| TNKS | Tankyrase, TRF1-interacting ankyrin-related ADP-ribose polymerase | -1.78 | 1.35E-04 |
| TNKS1BP1 | Tankyrase 1 binding protein 1, 182kDa | -1.388 | 2.36E-04 |
| TNNC1 | Troponin C type 1 (slow) | -1.306 | 2.53E-03 |
| TNS1 | Tensin 1 | -1.952 | 1.21E-17 |
| TP53INP1 | Tumor protein p53 inducible nuclear protein 1 | -1.809 | 1.68E-04 |
| TP53INP2 | Tumor protein p53 inducible nuclear protein 2 | 1.834 | 8.13E-05 |
| TP63 | Tumor protein p63 | -1.503 | 4.93E-04 |
| TPCN1 | Two pore segment channel 1 | 1.687 | 7.89E-07 |
| TPD52 | Tumor protein D52 | 1.286 | 2.00E-02 |
| TPGS2 | Tubulin polyglutamylase complex subunit 2 | 1.363 | 3.80E-04 |
| TPK1 | Thiamin pyrophosphokinase 1 | 1.317 | 1.91E-02 |
| TPM4 | Tropomyosin 4 | 2.004 | 2.79E-10 |
| TPMT | Thiopurine S-methyltransferase | 1.299 | 1.29E-02 |
| TPP1 | Tripeptidyl peptidase I | 1.504 | 1.47E-08 |
| TPPP3 | Tubulin polymerization-promoting protein family member 3 | 1.756 | 6.77E-09 |
| TPST2 | Tyrosylprotein sulfotransferase 2 | -1.487 | 1.63E-06 |
| TRAF3 | TNF receptor-associated factor 3 | 2.157 | 1.29E-08 |
| TRAF3IP1 | TNF receptor-associated factor 3 interacting protein 1 | -1.425 | 1.36E-05 |
| TRAPPC6A | Trafficking protein particle complex 6A | 1.404 | 2.62E-02 |
| TREX1 | Three prime repair exonuclease 1 | 1.629 | 5.40E-04 |
| TRIB1 | Tribbles pseudokinase 1 | 1.503 | 2.52E-05 |
| TRIM21 | Tripartite motif containing 21 | 1.311 | 2.73E-02 |
| TRIM23 | Tripartite motif containing 23 | -1.273 | 2.51E-02 |
| TRIM33 | Tripartite motif containing 33 | -2.144 | 2.00E-05 |
| TRIM45 | Tripartite motif containing 45 | -1.338 | 1.27E-03 |
| TRIT1 | tRNA isopentenyltransferase 1 | 1.291 | 1.49E-03 |
| TRMT61A | tRNA methyltransferase 61A | 1.532 | 7.26E-05 |
| TRRAP | Transformation/transcription domain-associated protein | -1.28 | 6.46E-03 |
| TSC22D3 | TSC22 domain family, member 3 | -1.321 | 3.56E-04 |
| TSPAN14 | Tetraspanin 14 | -1.369 | 2.51E-05 |
| TSPAN15 | Tetraspanin 15 | 1.349 | 1.90E-02 |
| TSPAN4 | Tetraspanin 4 | -1.296 | 1.03E-02 |
| TSPAN6 | Tetraspanin 6 | 1.326 | 1.92E-02 |
| TSPO | Translocator protein (18kDa) | -1.282 | 2.40E-02 |
| TSPYL1 | TSPY-like 1 | -1.412 | 3.45E-06 |
| TSPYL2 | TSPY-like 2 | -1.843 | 5.59E-09 |
| TSPYL4 | TSPY-like 4 | -1.611 | 2.45E-04 |
| TTC1 | Tetratricopeptide repeat domain 1 | 1.268 | 6.86E-03 |
| TTC33 | Tetratricopeptide repeat domain 33 | -1.41 | 1.14E-03 |
| TTC7B | Tetratricopeptide repeat domain 7B | -1.272 | 2.31E-03 |
| TTC9 | Tetratricopeptide repeat domain 9 | -1.757 | 8.75E-08 |
| TTF2 | Transcription termination factor, RNA polymerase II | 1.409 | 4.57E-03 |
| TTLL1 | Tubulin tyrosine ligase-like family member 1 | 1.802 | 5.52E-07 |
| TTN | Titin | -1.5 | 3.20E-06 |
| TTYH2 | Tweety family member 2 | 1.404 | 1.37E-03 |
| TUBA1A | Tubulin, alpha 1a | 1.635 | 7.02E-09 |
| TUBA1B | Tubulin, alpha 1b | 1.481 | 3.69E-07 |
| TUBA1C | Tubulin, alpha 1c | 1.549 | 1.41E-07 |
| TUBB | Tubulin, beta class I | 1.496 | 5.15E-05 |
| TUBB6 | Tubulin, beta 6 class V | 1.978 | 9.57E-09 |
| TXLNB | Taxilin beta | -1.69 | 1.48E-08 |
| TXN | Thioredoxin | 1.318 | 5.23E-05 |
| TXNDC5 | Thioredoxin domain containing 5 (endoplasmic reticulum) | 1.544 | 4.42E-04 |
| TXNIP | Thioredoxin interacting protein | -1.356 | 1.57E-02 |
| TYRP1 | Tyrosinase-related protein 1 | 1.574 | 1.41E-02 |
| UAP1L1 | UDP-N-acetylglucosamine pyrophosphorylase 1 like 1 | -1.339 | 1.82E-02 |
| UBA52 | Ubiquitin A-52 residue ribosomal protein fusion product 1 | -1.314 | 1.97E-04 |
| UBC | Ubiquitin C | -1.711 | 1.79E-09 |
| UBE2L6 | Ubiquitin-conjugating enzyme E2L 6 | -1.258 | 1.71E-02 |
| UBE2O | Ubiquitin-conjugating enzyme E2O | -1.264 | 2.59E-03 |
| UBE2Q2 | Ubiquitin-conjugating enzyme E2Q family member 2 | 1.381 | 3.21E-04 |
| UBE2T | Ubiquitin-conjugating enzyme E2T | -1.255 | 4.35E-03 |
| UBE4A | Ubiquitination factor E4A | -1.542 | 2.41E-07 |
| UBR5 | Ubiquitin protein ligase E3 component n-recognin 5 | -1.251 | 2.11E-02 |
| UBTD2 | Ubiquitin domain containing 2 | 1.318 | 2.55E-02 |
| UBXN1 | UBX domain protein 1 | -1.468 | 1.27E-07 |
| UCK1 | Uridine-cytidine kinase 1 | 1.349 | 3.02E-03 |
| UCK2 | Uridine-cytidine kinase 2 | 2.116 | 3.75E-06 |
| UCP2 | Uncoupling protein 2 (mitochondrial, proton carrier) | -1.313 | 5.97E-03 |
| UEVLD | UEV and lactate/malate dehyrogenase domains | 1.389 | 1.04E-02 |
| UGP2 | UDP-glucose pyrophosphorylase 2 | -1.302 | 3.23E-03 |
| UHMK1 | U2AF homology motif (UHM) kinase 1 | -4.417 | 4.21E-06 |
| UIMC1 | Ubiquitin interaction motif containing 1 | -1.333 | 6.61E-03 |
| ULBP3 | UL16 binding protein 3 | 2.937 | 3.43E-10 |
| UNC13B | unc-13 homolog B (C. elegans) | 1.32 | 8.10E-03 |
| UPF3A | UPF3 regulator of nonsense transcripts homolog A (yeast) | 1.792 | 1.25E-12 |
| URM1 | Ubiquitin related modifier 1 | 1.305 | 4.35E-04 |
| USP18 | Ubiquitin specific peptidase 18 | -1.588 | 8.19E-04 |
| USP19 | Ubiquitin specific peptidase 19 | -1.333 | 5.69E-04 |
| USP25 | Ubiquitin specific peptidase 25 | -1.407 | 2.17E-05 |
| USP48 | Ubiquitin specific peptidase 48 | 1.404 | 4.21E-05 |
| UVSSA | UV-stimulated scaffold protein A | -1.45 | 5.48E-04 |
| VANGL1 | VANGL planar cell polarity protein 1 | -1.305 | 2.39E-02 |
| VARS | Valyl-tRNA synthetase | 1.329 | 3.68E-03 |
| VASH1 | Vasohibin 1 | 2.613 | 2.76E-07 |
| VASP | Vasodilator-stimulated phosphoprotein | 1.458 | 8.14E-05 |
| VCAM1 | Vascular cell adhesion molecule 1 | 1.846 | 8.89E-03 |
| VCAN | Versican | 1.408 | 2.61E-03 |
| VEGFA | Vascular endothelial growth factor A | 1.464 | 2.28E-03 |
| VEGFB | Vascular endothelial growth factor B | -1.351 | 7.86E-05 |
| VEGFC | Vascular endothelial growth factor C | 1.449 | 1.95E-03 |
| VIM | Vimentin | 1.491 | 4.06E-05 |
| VIPR1 | Vasoactive intestinal peptide receptor 1 | -1.823 | 7.27E-08 |
| VMP1 | Vacuole membrane protein 1 | 1.409 | 4.37E-04 |
| VNN1 | Vanin 1 | -1.893 | 5.66E-08 |
| VOPP1 | Vesicular, overexpressed in cancer, prosurvival protein 1 | -2.012 | 1.14E-12 |
| VPS13C | Vacuolar protein sorting 13 homolog C (S. cerevisiae) | -1.275 | 1.62E-02 |
| VPS13D | Vacuolar protein sorting 13 homolog D (S. cerevisiae) | -1.277 | 5.49E-03 |
| VPS33A | Vacuolar protein sorting 33 homolog A (S. cerevisiae) | -1.33 | 1.07E-04 |
| VPS45 | Vacuolar protein sorting 45 homolog (S. cerevisiae) | 1.256 | 1.76E-02 |
| VPS51 | Vacuolar protein sorting 51 homolog (S. cerevisiae) | 1.318 | 3.02E-03 |
| VPS52 | Vacuolar protein sorting 52 homolog (S. cerevisiae) | 1.384 | 7.74E-04 |
| VRK2 | Vaccinia related kinase 2 | 1.426 | 2.67E-03 |
| VSTM2L | V-set and transmembrane domain containing 2 like | -1.513 | 6.88E-04 |
| VWF | Von Willebrand factor | -1.27 | 1.31E-02 |
| WARS | Tryptophanyl-tRNA synthetase | 1.621 | 1.61E-05 |
| WASF3 | WAS protein family, member 3 | -1.417 | 3.05E-05 |
| WDFY1 | WD repeat and FYVE domain containing 1 | -1.265 | 3.02E-03 |
| WDR1 | WD repeat domain 1 | 1.499 | 1.01E-06 |
| WDR12 | WD repeat domain 12 | 1.33 | 1.54E-03 |
| WDR17 | WD repeat domain 17 | -1.44 | 2.26E-03 |
| WDR24 | WD repeat domain 24 | 1.407 | 3.83E-05 |
| WDR4 | WD repeat domain 4 | 1.743 | 1.83E-04 |
| WDR46 | WD repeat domain 46 | 1.32 | 5.67E-04 |
| WDR59 | WD repeat domain 59 | -1.276 | 9.70E-04 |
| WDR62 | WD repeat domain 62 | 1.458 | 1.77E-02 |
| WDR77 | WD repeat domain 77 | 1.271 | 2.77E-04 |
| WDR89 | WD repeat domain 89 | 1.301 | 4.18E-03 |
| WDR92 | WD repeat domain 92 | -1.336 | 1.26E-03 |
| WHSC1L1 | Wolf-Hirschhorn syndrome candidate 1-like 1 | -1.363 | 4.47E-05 |
| WIPF1 | WAS/WASL interacting protein family, member 1 | 1.344 | 3.95E-03 |
| WIPI2 | WD repeat domain, phosphoinositide interacting 2 | -1.295 | 5.01E-04 |
| WNK2 | WNK lysine deficient protein kinase 2 | -1.471 | 3.11E-06 |
| WNT11 | Wingless-type MMTV integration site family, member 11 | 1.421 | 1.33E-04 |
| WRN | Werner syndrome, RecQ helicase-like | -1.369 | 1.31E-02 |
| WWP1 | WW domain containing E3 ubiquitin protein ligase 1 | -1.727 | 5.48E-11 |
| XBP1 | X-box binding protein 1 | 1.352 | 1.60E-05 |
| XIAP | X-linked inhibitor of apoptosis, E3 ubiquitin protein ligase | -1.772 | 1.85E-03 |
| XIRP2 | Xin actin binding repeat containing 2 | 1.908 | 6.29E-06 |
| XPNPEP3 | X-prolyl aminopeptidase 3, mitochondrial | 1.419 | 5.80E-04 |
| XPO1 | Exportin 1 | 1.469 | 1.58E-02 |
| XRN1 | 5'-3' exoribonuclease 1 | -1.957 | 3.39E-05 |
| YARS | Tyrosyl-tRNA synthetase | 1.407 | 2.01E-05 |
| YARS2 | Tyrosyl-tRNA synthetase 2, mitochondrial | 1.44 | 3.51E-04 |
| YBEY | YbeY metallopeptidase (putative) | -1.384 | 2.25E-04 |
| YBX2 | Y box binding protein 2 | 1.489 | 2.09E-03 |
| YBX3 | Y box binding protein 3 | -1.846 | 4.07E-15 |
| YEATS2 | YEATS domain containing 2 | 1.669 | 6.27E-09 |
| YPEL1 | Yippee-like 1 | -1.539 | 1.06E-03 |
| YPEL2 | Yippee-like 2 | -1.334 | 4.78E-04 |
| YPEL3 | Yippee-like 3 | -1.649 | 1.69E-05 |
| YWHAB | Tyrosine 3-monooxygenase/tryptophan 5-monooxygenase activation protein, beta | 1.264 | 8.27E-04 |
| YWHAQ | Tyrosine 3-monooxygenase/tryptophan 5-monooxygenase activation protein, theta | 1.399 | 2.88E-04 |
| YWHAZ | Tyrosine 3-monooxygenase/tryptophan 5-monooxygenase activation protein, zeta | 1.633 | 8.42E-04 |
| ZACN | Zinc activated ligand-gated ion channel | 1.5 | 2.88E-03 |
| ZAK | Sterile alpha motif and leucine zipper containing kinase AZK | -1.622 | 2.35E-02 |
| ZBED1 | Zinc finger, BED-type containing 1 | -1.408 | 1.10E-04 |
| ZBED5 | Zinc finger, BED-type containing 5 | -1.389 | 2.49E-03 |
| ZBED6 | Zinc finger, BED-type containing 6 | -3.593 | 1.11E-03 |
| ZBTB16 | Zinc finger and BTB domain containing 16 | -1.604 | 3.02E-05 |
| ZBTB24 | Zinc finger and BTB domain containing 24 | -1.264 | 1.10E-02 |
| ZBTB26 | Zinc finger and BTB domain containing 26 | -1.617 | 9.39E-04 |
| ZBTB38 | Zinc finger and BTB domain containing 38 | -1.567 | 5.46E-05 |
| ZBTB40 | Zinc finger and BTB domain containing 40 | 1.294 | 1.38E-02 |
| ZBTB43 | Zinc finger and BTB domain containing 43 | -1.308 | 8.90E-03 |
| ZC3H11A | Zinc finger CCCH-type containing 11A | -1.288 | 6.67E-04 |
| ZC3H12C | Zinc finger CCCH-type containing 12C | -2.14 | 1.88E-05 |
| ZC3H15 | Zinc finger CCCH-type containing 15 | 1.297 | 5.04E-04 |
| ZC3H4 | Zinc finger CCCH-type containing 4 | -1.378 | 5.37E-03 |
| ZC3H6 | Zinc finger CCCH-type containing 6 | -2.504 | 9.08E-20 |
| ZCCHC12 | Zinc finger, CCHC domain containing 12 | 1.787 | 6.13E-04 |
| ZCCHC14 | Zinc finger, CCHC domain containing 14 | -1.539 | 2.11E-04 |
| ZCCHC17 | Zinc finger, CCHC domain containing 17 | -1.459 | 2.72E-09 |
| ZCCHC24 | Zinc finger, CCHC domain containing 24 | -1.258 | 2.76E-03 |
| ZCCHC6 | Zinc finger, CCHC domain containing 6 | -1.494 | 1.27E-05 |
| ZDHHC16 | Zinc finger, DHHC-type containing 16 | 1.274 | 5.26E-03 |
| ZDHHC23 | Zinc finger, DHHC-type containing 23 | -1.514 | 5.87E-03 |
| ZDHHC9 | Zinc finger, DHHC-type containing 9 | 1.395 | 3.68E-04 |
| ZFAND2B | Zinc finger, AN1-type domain 2B | 1.386 | 1.37E-04 |
| ZFAND3 | Zinc finger, AN1-type domain 3 | 1.27 | 9.46E-04 |
| ZFAND5 | Zinc finger, AN1-type domain 5 | -1.516 | 3.39E-04 |
| ZFAND6 | Zinc finger, AN1-type domain 6 | -1.312 | 1.77E-04 |
| ZFHX2 | Zinc finger homeobox 2 | 2.217 | 5.91E-09 |
| ZFHX4 | Zinc finger homeobox 4 | -1.421 | 1.26E-02 |
| ZFP62 | ZFP62 zinc finger protein | -1.474 | 1.20E-02 |
| ZFP90 | ZFP90 zinc finger protein | 1.31 | 9.13E-03 |
| ZFYVE19 | Zinc finger, FYVE domain containing 19 | -1.951 | 4.80E-08 |
| ZKSCAN1 | Zinc finger with KRAB and SCAN domains 1 | -1.363 | 8.25E-03 |
| ZMYM4 | Zinc finger, MYM-type 4 | -1.328 | 9.39E-03 |
| ZMYM6 | Zinc finger, MYM-type 6 | -1.471 | 2.70E-03 |
| ZNF106 | Zinc finger protein 106 | 1.845 | 7.54E-05 |
| ZNF112 | Zinc finger protein 112 | -1.519 | 1.84E-03 |
| ZNF134 | Zinc finger protein 134 | -1.386 | 1.32E-02 |
| ZNF135 | Zinc finger protein 135 | -1.408 | 6.31E-03 |
| ZNF140 | Zinc finger protein 140 | -1.272 | 6.83E-03 |
| ZNF19 | Zinc finger protein 19 | -1.256 | 1.98E-02 |
| ZNF212 | Zinc finger protein 212 | -1.436 | 3.83E-03 |
| ZNF24 | Zinc finger protein 24 | -1.576 | 5.35E-05 |
| ZNF256 | Zinc finger protein 256 | -1.575 | 3.30E-04 |
| ZNF274 | Zinc finger protein 274 | -1.84 | 5.48E-09 |
| ZNF304 | Zinc finger protein 304 | -1.257 | 1.40E-02 |
| ZNF32 | Zinc finger protein 32 | -1.26 | 7.60E-03 |
| ZNF331 | Zinc finger protein 331 | -1.349 | 1.00E-02 |
| ZNF35 | Zinc finger protein 35 | -1.383 | 2.44E-04 |
| ZNF362 | Zinc finger protein 362 | -1.401 | 1.97E-04 |
| ZNF395 | Zinc finger protein 395 | -1.47 | 2.76E-05 |
| ZNF419 | Zinc finger protein 419 | -1.71 | 4.90E-09 |
| ZNF423 | Zinc finger protein 423 | -1.25 | 9.44E-03 |
| ZNF428 | Zinc finger protein 428 | 1.324 | 2.63E-02 |
| ZNF445 | Zinc finger protein 445 | -1.292 | 7.30E-04 |
| ZNF502 | Zinc finger protein 502 | -1.476 | 6.45E-03 |
| ZNF518A | Zinc finger protein 518A | -1.565 | 1.39E-03 |
| ZNF518B | Zinc finger protein 518B | -1.36 | 1.24E-03 |
| ZNF555 | Zinc finger protein 555 | -1.512 | 1.21E-02 |
| ZNF593 | Zinc finger protein 593 | 1.472 | 1.30E-04 |
| ZNF608 | Zinc finger protein 608 | 1.644 | 5.80E-05 |
| ZNF613 | Zinc finger protein 613 | -1.483 | 3.36E-03 |
| ZNF614 | Zinc finger protein 614 | -1.371 | 1.86E-02 |
| ZNF70 | Zinc finger protein 70 | -1.29 | 8.64E-03 |
| ZNF710 | Zinc finger protein 710 | -1.849 | 1.16E-05 |
| ZNF740 | Zinc finger protein 740 | -1.276 | 1.88E-02 |
| ZNF75A | Zinc finger protein 75a | -1.421 | 7.41E-03 |
| ZNF777 | Zinc finger protein 777 | -1.297 | 1.35E-02 |
| ZNF784 | Zinc finger protein 784 | -1.359 | 2.75E-03 |
| ZNF800 | Zinc finger protein 800 | -1.334 | 1.56E-02 |
| ZNF829 | Zinc finger protein 829 | -1.375 | 1.62E-02 |
| ZNHIT3 | Zinc finger, HIT-type containing 3 | 1.449 | 1.61E-04 |
| ZSCAN12 | Zinc finger and SCAN domain containing 12 | -1.464 | 1.06E-02 |
| ZUFSP | Zinc finger with UFM1-specific peptidase domain | 1.344 | 1.35E-02 |
| ZXDB | Zinc finger, X-linked, duplicated B | -1.332 | 1.29E-02 |
| ZXDC | ZXD family zinc finger C | -1.284 | 2.38E-02 |
| ZYX | Zyxin | 1.376 | 1.03E-04 |

^1^ Fold changes are up or down in compensating animals compared to restricted animals
